# Photoredox Imino Functionalizations of Olefins

**DOI:** 10.1002/anie.201708497

**Published:** 2017-09-22

**Authors:** Jacob Davies, Nadeem S. Sheikh, Daniele Leonori

**Affiliations:** ^1^ School of Chemistry University of Manchester Oxford Road Manchester M13 9PL UK; ^2^ Department of Chemistry Faculty of Science King Faisal University P.O. Box 380 Al-Ahsa 31982 Saudi Arabia

**Keywords:** cyclization, nitrogen heterocycles, photochemistry, radicals, reaction mechanisms

## Abstract

Shown herein is that polyfunctionalized nitrogen heterocycles can be easily prepared by a visible‐light‐mediated radical cascade process. This divergent strategy features the oxidative generation of iminyl radicals and subsequent cyclization/radical trapping, which allows the effective construction of highly functionalized heterocycles. The reactions proceed efficiently at room temperature, utilize an organic photocatalyst, use simple and readily available materials, and generate, in a single step, valuable building blocks that would be difficult to prepare by other methods.

Small‐molecule nitrogen heterocycles constitute the core of many drugs and agrochemicals and are one of the key epitopes evaluated in biological screenings.^1^ As a result, the invention of methods enabling their rapid construction is a topic of continuous scientific endeavour. An overarching goal in the design of these methodologies is the identification of divergent approaches whereby simple starting materials are converted into a broad array of products which contain different functional groups and efficiently tap into chemical space.

Nitrogen radicals are a class of versatile synthetic intermediates, and their reactivity encompasses a series of powerful bond‐forming reactions, like intramolecular cyclization and 1,5‐H abstraction.^2^ In recent years, owing to the power of visible‐light photoredox catalysis^3^ in realizing single‐electron‐transfer (SET) processes,^4^ the chemistry of nitrogen‐radicals has witnessed a remarkable resurgence in scientific interest.^5^

We have developed a class of electron‐poor O‐aryl oximes and aryloxy amides which allows, upon reductive SET, access to iminyl^6^ and amidyl^7^ radicals to use in radical hydroaminations (Scheme 1 A). However, despite all our efforts, we have not been able to harness this reactivity mode for the development of much sought after, but also more challenging, amino‐functionalization processes.2i, 8 Herein, we describe our work towards this goal, which has led to the development of a powerful platform for the divergent assembly of polyfunctionalized nitrogen heterocycles (Scheme 1 B).^9^

**Scheme 1:**
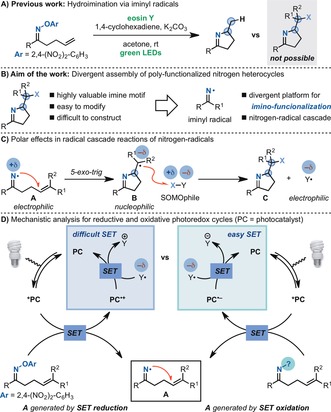
Nitrogen heterocycles and mechanistic analysis of the divergent functionalization processes developed in this work.

While facing the difficulties of developing cascade photoredox imino functionalizations of electron‐poor O‐aryl oximes,^6^ we realized that an inherent chemical reactivity issue was associated with the envisaged catalytic cycle. The nitrogen radicals **A** are electrophilic species and undergo facile *exo‐trig* cyclizations (Scheme 1 C).^10^ However, upon ring closure, they generate the nucleophilic carbon radical **B**, which benefits from polar effects^11^ in the reaction with SOMOphiles X−Y. This process leads to the formation of the imino‐functionalized product **C** and the radical Y^.^. This radical, even though it is not involved in any bond‐forming event, controls the fate of our photoredox manifold. In fact, owing to the required polarization of X−Y for efficient reaction with **B**, Y^.^ is expected to be electrophilic. This species generates an electronic mismatch in our first‐generation photoredox cycle, whereby [PC^.+^ + Y^.^ → PC + Y^+^] is a endothermic process (Scheme 1 D, left). Therefore, we reasoned that should the iminyl **A** be generated by oxidative SET from the visible‐light‐excited photocatalyst (*PC), a facile SET between Y^.^ and PC^⋅−^ ought to take place, thus ensuring catalytic activity (Scheme 1 D, right). An additional challenge associated with the realization of this approach concerns the nature of the SOMOphile X−Y.^12^ In fact, many of these species are excellent SET quenchers for *PC, and they have been exploited in many powerful atom/group‐transfer reactions and they can lead to the formation of onium ions.12b As a result, we would require the oxidative generation of the iminyl radical, its intramolecular cyclization, and the following intermolecular radical reaction to supersede any other possible photoredox and ionic pathway.

Inspired by the pioneering work of Forrester^13^ and Zard,2h we speculated that the oxime **I** might serve as a traceless (n+σ+σ) electrophore and undergo a sequence of two β‐scissions upon in situ deprotonation (**II**) and SET oxidation (Scheme 2 A). This step would deliver an iminyl radical in conjunction with the release of CO_2_ and HCOH (**III**→**IV**).^14^ As α‐hydroxy acids (e.g., **1**) have been employed in many photoredox decarboxylations,12c, 15 we were confident that such a substrate design would be feasible. Surprisingly, electrochemical analysis of the model substrate **2 a** revealed its oxidation potential to be almost outside the range for photoredox oxidation (*E*_1/2_^ox^=2.10 V vs. SCE; Scheme 2 B). In fact, previous activation methods required the use of strong oxidants either under forcing conditions or with the formation of the corresponding Barton ester.2h,^13^

**Scheme 2:**
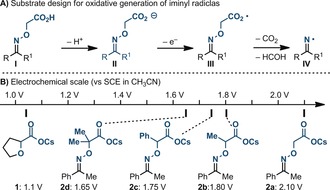
Substrate design and electrochemical studies.

Hence, we speculated that the addition of organyl groups at the methylenic position might inductively increase the electron density at the carboxylate, and lower its *E*_1/2_^ox^. Pleasingly, the addition of a methyl (**2 b**) and a phenyl (**2 c**) group lowered the *E*_1/2_^ox^ by 0.25 and 0.3 V, respectively. More importantly, the substrate **2 d**, which was prepared by condensation of acetophenone with a commercially available hydroxylamine, displayed *E*_1/2_^ox^=1.65 V (vs. SCE), a value which can be reached by some organic photoredox catalysts.^16^

Having identified a suitable electrophore, we prepared the substrate **3 a** and begun our investigations by looking at developing an oxidative hydroimination (Scheme 3). To accomplish this goal, we selected the methyl acridinium perchlorate **4** (E*_1/2_=+2.06 V vs. SCE)3c, 17 as the photoredox catalyst and evaluated a range of solvents and bases under blue LED irradiation. Pleasingly, in the presence of an aryl‐disulfide as H‐atom relay catalyst,^18^ and 2,6‐lutidine as the base, **5 a** was obtained in 81 % yield, which, to the best of our knowledge, represents the first fully catalytic radical hydroimination of olefins. Control experiments established the requirement for **4** and continuous irradiation and the calculated quantum yield^19^ *Φ*=0.09 supports the photoredox nature of the process.^16^

**Scheme 3:**
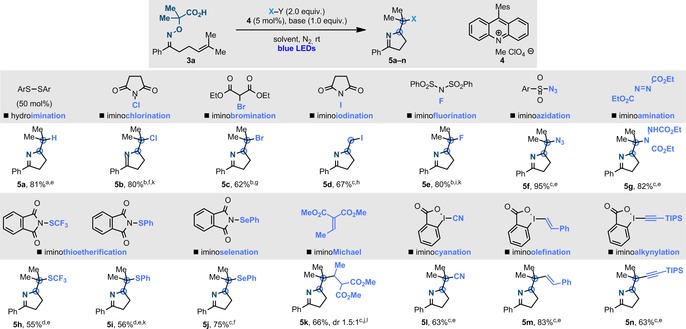
Scope of the imino functionalization strategy. Base used: [a] 2,6‐lutidine, [b] K_2_CO_3_, [c] Cs_2_CO_3_, [d] CsOBz. Solvent used: [e] CH_2_Cl_2_, [f] Toluene, [g] *i*PrOH, [h] MeOH, [i] Hexafluoroisopropanol, [j] CH_3_CN/H_2_O (2:1). Equivalents of X‐Y: [k] 3.0 equiv, [n] 4.0 equiv. Bn=benzyl, TIPS=triisopropylsilyl.

We next turned our attention to defining the capacity for imino functionalization in the presence of external coupling partners. By employing NCS, diethyl‐bromo‐malonate, NIS, and NFSI, we developed olefin imino halogenations in excellent to good yield (**5 b**–**e**). Interestingly, the use of Selectfluor as a radical fluorinating agent^20^ provided **5 e** in considerably lower yield. Imino iodinations and imino fluorinations have not been reported previously. The use of arylsulfonyl azide and DEAD as coupling partners enabled the preparation of substrates with two nitrogen‐based functional groups in different oxidation states [imine and azide (**5 f**) or hydrazine (**5 g**)] and with orthogonal reactivity. This aspect opens the potential for further functionalization and application in click chemistry ligation. As thioether motifs are frequently found in pharmaceutical and agrochemical compounds,^21^ we evaluated their introduction as part of our strategy. Pleasingly, we were able to form the imines **5 h**,**i** which incorporated S−CF_3_ and S−Ph groups. In this case, CsOBz12h proved to be uniquely effective in promoting good conversions. The formation of **5 h** is particularly relevant because the introduction of the S−CF_3_ group in organic molecules is a significant but challenging task in medicinal chemistry.^22^ This reactivity was also successfully extended to the introduction of a Se functionality (**5 j**). Finally, we evaluated this approach for the installation of organic groups based on the cascade formation of C−N and C−C bonds. By using a Michael acceptor, we obtained the product **5 k**, which represents a radical imino Michael cascade. Furthermore, by using hypervalent IBX reagents,^23^ we have developed unprecedented radical imino cyanation (**5 l**), olefination (**5 m**), and alkynylation (**5 n**) processes. The successful formation of **5 l** is interesting, as recent literature reports described the cyanating reagent to be able to react with only C(sp^3^)‐radicals α to a heteroatom.12i The reagent used for the formation of **5 m** 24 has not been used before in radical manifolds, and makes this example the first use of IBX reagents as vinylation partners in free‐radical processes.

Regarding the optimization of these 14 different imino functionalizations, we were able to use **4** as the photocatalyst across all the systems, but the base, solvent, and reaction concentration had to be adjusted to maximize the reaction efficiency. In general, we have found that the inorganic bases Cs_2_CO_3_ and K_2_CO_3_, and the solvents CH_2_Cl_2_ and toluene, provided the best results.^16^ We have also conducted additional electrochemical Stern–Volmer and quantum yield (*Φ*) studies, which corroborate our working hypothesis for the reaction mechanism.^16^ According to these studies we believe that, in general, the iminofunctionalizations proceed by a single photoredox catalytic cycle starting with the SET oxidation of **3 a**. However, in the case of the IBX reagents (imino‐cyanation **5 l** and alkynylation **5 m**), the quantum yields 2<*Φ*<5 suggest that very short‐lived productive radical‐chain processes might be operating together with the main photoredox manifold. It is worth mentioning, that none of these processes provided the desired products using our first generation iminyl radical precursors. This outcome clearly highlights the importance of polar effects and redox balance in the development of cascade reactions of nitrogen radicals.

Having developed a platform for the divergent assembly of poly‐functionalized nitrogen heterocycles, we expanded the reaction scope. As shown in Scheme 4, we were able to implement substrates containing terminal olefins (**3 b**), which lead, via the intermediacy of a primary radical, to the imines **6 a**–**h**. Pyridine substituents were tolerated, thus providing the nicotine analogues **6 i**–**k**. Substrates containing disubstituted olefins (**3 d**,**e**) were also successfully engaged, thus proving that the strategy is amenable to the formation and functionalization of secondary benzylic (**7 a**–**e**) and α‐ester (**7 f**–**j**) radicals. By embedding the olefin into a cycle (**3 f**), we generated polyfunctionalized bicyclic molecules in good yield and good to moderate diastereoselectivity, thus favoring the all‐*syn* isomer (**7 j**–**q**). We also expanded the scope of coupling partners in terms of Michael acceptors (**8 a**,**b**) and IBX reagents for imino olefination (**8 c**) and alkynylation (**8 d**,**e**). Substrates prepared from α‐ketoacids could also be employed leading to the formation of proline‐like products (**8 f**–**i**). Lastly, this radical platform was evaluated as an enabling tool for the late‐stage imino functionalization of biologically active molecules. Therefore, we tested the structurally complex and densely functionalized morphane derivative thevinone (**9**),^25^ which was successfully engaged in the formation of products derived from imino azidation (**10 a**), imino amination (**10 b**), and imino selenation (**10 c**) of the olefin moiety.

**Scheme 4:**
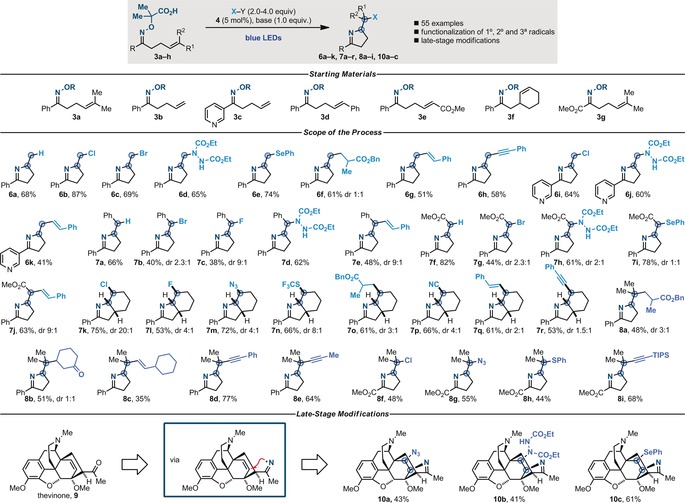
Reaction scope. See Scheme 3 for solvent and base used in each imino functionalization reaction.^17^

In conclusion, we have developed a general method for the fast and divergent assembly of polyfunctionalized nitrogen heterocycles. The reaction involves the organo‐photoredox generation of iminyl radicals by oxidative SET of a traceless electrophore and a subsequent cyclization/functionalization cascade with a broad range of SOMOphiles. This strategy generates useful building blocks in a single step, and its application to a number of more complex examples highlights its broad applicability.

## Conflict of interest

The authors declare no conflict of interest.

## Supporting information

As a service to our authors and readers, this journal provides supporting information supplied by the authors. Such materials are peer reviewed and may be re‐organized for online delivery, but are not copy‐edited or typeset. Technical support issues arising from supporting information (other than missing files) should be addressed to the authors.

Supplementary
