# Supplementary material for: Photoredox Imino Functionalizations of Olefins
Source: Angew Chem Int Ed Engl. 2017 Sep 22;56(43):13361–5. doi: 10.1002/anie.201708497 (PMC5656829; doi:10.1002/anie.201708497)
Supplement: Supplementary file 1 — Supplementary [file ANIE-56-13361-s001.pdf]

Angewandte Chemie
Eine Zeitschrift der Gesellschaft Deutscher Chemiker

Supporting Information

# Photoredox Imino Functionalizations of Olefins

*Jacob Davies, Nadeem S. Sheikh, and Daniele Leonori**

anie_201708497_sm_miscellaneous_information.pdf

## Table of Contents


1 General Experimental Details ........................................ 3

2 Abbreviations ........................................ 4

Photoredox catalysts: ........................................ 4

3 Starting Material Synthesis ........................................ 5

3.1 Synthesis of Hypervalent Iodine Reagents ........................................ 5

3.2 Synthesis of Ketones ........................................ 8

3.3 Synthesis of Oximes 3a–h ........................................ 15

3.4 General procedure for the synthesis of oximes – GP3 ........................................ 15

4 Electrochemical studies ........................................ 19

4.1 Synthesis of Substrates ........................................ 19

4.2 General Experimental Detail ........................................ 21

4.3 Electrochemical studies ........................................ 22

4.4 Electrochemical Potentials ........................................ 23

4.5 Cyclic Voltammograms ........................................ 26

5 Reaction Optimizations ........................................ 36

5.1 Hydro-imination ........................................ 36

5.2 Imino-chlorination ........................................ 43

5.3 Imino-bromination ........................................ 46

5.4 Imino-iodination ........................................ 49

5.5 Imino-fluorination ........................................ 51

5.6 Imino-azidation ........................................ 55

5.7 Imino-amination ........ 57
5.8 Imino-thioetherificaiton ........ 59
5.9 Imino-selenation ........ 63
5.10 Imino-Michael ........ 65
5.11 Imino-cyanation ........ 67
5.12 Imino-olefination ........ 71
5.13 Imino-alkynylation ........ 73
6 Iminofunctionalization Reaction Products ........ 75
6.1 General Procedure for the Purification of the Iminofunctionalization Products ........ 75
6.2 Picture of Reaction Set-Up ........ 75
6.3 Product Structures ........ 76
6.4 Product characterizations ........ 77
7 Mechanistic Considerations ........ 103
7.1 Emission Quenching Experiments – Stern-Volmer Studies ........ 103
7.2 Quantum Yield Determination ........ 104
7.3 Substrate Oxidation and Fragmentation ........ 107
7.4 Nucleophilicity of the Carbon-Radicals ........ 108
8 Computational Studies ........ 110
8.1 Computational Methods ........ 110
8.2 Carbon-Centered Radicals ........ 111
8.3 Bond Dissociation Enthalpies ........ 120
8.4 Structural Analysis for Carboxylate Anions ........ 143
9 NMR Spectra ........ 162
10 References ........ 227

## 1 General Experimental Details

All required fine chemicals were used directly without purification unless stated otherwise. All air and moisture sensitive reactions were carried out under nitrogen atmosphere using standard Schlenk manifold technique. 1-carboxy-1-methylethoxyammonium chloride was purchased by Fluorochem and used without further purification. $^{1}H$ and $^{13}C$ Nuclear Magnetic Resonance (NMR) spectra were acquired at various field strengths as indicated and were referenced to $CHCl_3$ (7.27 and 77.0 ppm for $^{1}H$ and $^{13}C$ respectively). $^{1}H$ NMR coupling constants are reported in Hertz and refer to apparent multiplicities and not true coupling constants. Data are reported as follows: chemical shift, integration, multiplicity (s = singlet, br s = broad singlet, d = doublet, t = triplet, q = quartet, qi = quintet, sx = sextet, sp = septet, m = multiplet, dd = doublet of doublets, etc.), proton assignment (determined by 2D NMR experiments: COSY, HSQC and HMBC) where possible. High-resolution mass spectra were obtained using a JEOL JMS-700 spectrometer or a Fissions VG Trio 2000 quadrupole mass spectrometer. Spectra were obtained using electron impact ionization (EI) and chemical ionization (CI) techniques, or positive electrospray (ES). Infra-red spectra were recorded using a JASCO FT/IR 410 spectrometer or using an ATI Mattson Genesis Seris FTIR spectrometer as evaporated films or liquid films. Analytical TLC: aluminum backed plates pre-coated (0.25 mm) with Merck Silica Gel 60 F254. Compounds were visualized by exposure to UV-light or by dipping the plates in permanganate ($KMnO_4$) stain followed by heating. Flash column chromatography was performed using Merck Silica Gel 60 (40–63 μm). All mixed solvent eluents are reported as v/v solutions. UV/Vis spectra were obtained using an Agilent 6453 spectrometer and 1 mm High Precision Cell made of quartz from Hellma Analytics.

The LEDs were bought from LEDLightZone.

All the reactions were conducted in CEM 10 mL glass microwave tubes.

## 2 Abbreviations

**Photoredox catalysts:**

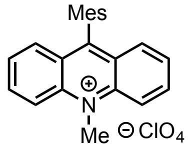


**PC1**

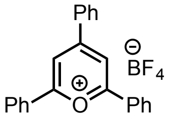


**PC2**

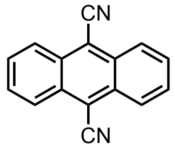


**PC3**

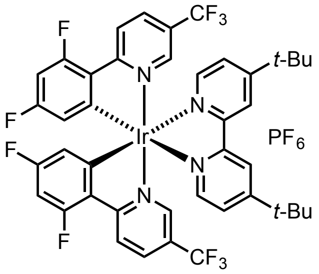


**PC4**

## 3 Starting Material Synthesis

### 3.1 Synthesis of Hypervalent Iodine Reagents

The following IBX reagents **S1–4** are not commercially available and were prepared according to literature procedures.

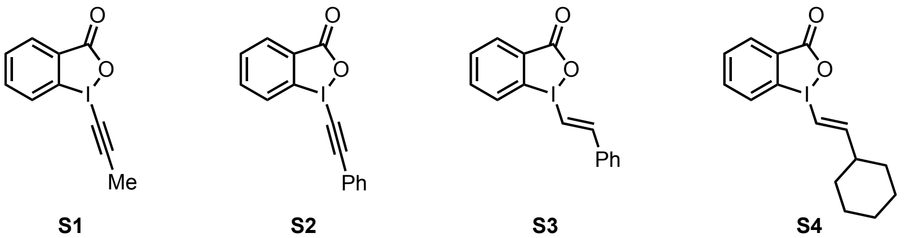


**1-Hydroxy-1 λ³-Benzo[d][1,2]iodaoxol-3(1H)-one (S5)**

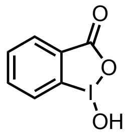

2-Iodobenzoic acid (5.7 g, 23.0 mmol, 1.0 equiv.) and $NaIO_4$ (5.2 g, 24.3 mmol, 1.05 equiv.) were stirred under reflux in aqueous acetic acid (35 mL, 30% v/v) for 4 h. The reaction mixture was diluted with $H_2O$ (100 mL) and allowed to cool to r.t (whilst being protected from light). After 1 h, the crude product was collected by filtration, washed with cold water and dried under vacuum in the dark to give **S5** (5.7 g, 94%). $^1$H NMR (400 MHz, MeOD) δ 8.82 (1H, dd, *J* = 7.5, 1.3 Hz), 8.69 (1H, dd, *J* = 8.2, 6.8, 1.4 Hz), 8.64 (1H, dd, *J* = 8.1, 1.2 Hz), 8.44 (1H, ddd, *J* = 8.0, 7.0, 1.4 Hz). Data in accordance with the literature.[1]

**1-(Prop-1-yn-1-yl)-1 λ³-Benzo[d][1,2]iodaoxol-3(1H)-one (S1)**

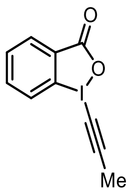

Trimethylsilyl triflate (0.65 mL, 3.6 mmol, 1.1 equiv.) was added dropwise to a solution of **S5** (0.86 g, 3.3 mmol, 1.0 equiv.) in $CH_2Cl_2$ (9 mL). The reaction mixture was stirred for 1 h and then 1-methyl-2-trimethylsilylacetylene (0.54 mL, 3.6 mmol, 1.1 equiv.) was added. The reaction mixture was stirred for 5 h at r.t. Saturated $NaHCO_3$ (20 ml) was added and the mixture was stirred vigorously. The layers of were separated and the aqueous layer was washed with $CH_2Cl_2$ (x3). The combined organic layers were dried ($Mg_2SO_4$), filtered and the solvent removed under reduced pressure. Purification by column chromatography (EtOAc)

gave **S1** (0.36 g, 38 %). $^1$H NMR (500 MHz, MeOD) δ 8.35 (1H, d, *J* = 8.2 Hz), 8.25 (1H, d, *J* = 7.4 Hz), 7.87 (1H, t, *J* = 7.7 Hz), 7.81 (1H, t, *J* = 7.3 Hz), 3.31 (3H, s, *J* = 2.34). Data in accordance to the literature.[2]

**1-(Phenylethynyl)-1 λ$^3$-Benzo[d][1,2]iodaoxol-3(1H)-one (S2)**

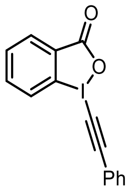


Trimethylsilyl triflate (1.5 mL, 8.3 mmol, 1.1 equiv.) was added dropwise to a solution of **S5** (2.0 g, 7.6 mmol, 1.0 equiv.) in $CH_2Cl_2$ (20 ml). The reaction mixture was stirred for 1 h and then 1-phenyl-2-trimethylsilylacetylene (1.6 mL, 8.3 mmol, 1.1 equiv.) was added. The reaction mixture was stirred for 6 h at r.t. Saturated $NaHCO_3$ (20 mL) was added and the mixture was stirred vigorously. The precipitate was filtered and the 2 layers of the filtrate were separated. The organic layer was washed with saturated $NaHCO_3$, dried ($Mg_2SO_4$), filtered and the solvent removed under reduced pressure. The solid was combined with the collected precipitate and recrystallized from hot MeCN to give **S2** (1.1 g, 42%). $^1$H NMR (400 MHz, MeOD) δ 12.36 (1H, d, *J* = 8.2 Hz), 12.24 (1H, dd, *J* = 7.4, 1.6 Hz), 11.91–11.84 (1H, m), 11.80 (1H, t, *J* = 7.3 Hz), 11.67 (2H, dd, *J* = 8.0, 1.3 Hz), 11.54–11.43 (3H, m). Data in accordance with the literature.[1]

**(*E*)-1-Styryl-1λ$^3$-Benzo[d][1,2]iodaoxol-3(1H)-one (S3)**

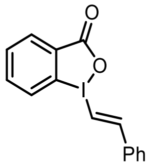


*m*-CPBA (1.1 g, 5.5 mmol, 1.1 equiv.) was added to a solution of 2-iodobenzoic acid (1.2 g, 5.0 mmol, 1.0 equiv.) in $CHCl_2$ (24 mL). The reaction mixture was cooled to 0 ˚C and triflic acid (0.7 ml, 7.5 mmol, 1.5 equiv.) was added at this temperature before being stirred at r.t for 15 mins. The reaction mixture was again cooled to 0 ºC over 10 mins and *trans*-2-phenylvinylboronic acid (1.0 g, 7.0 mmol, 1.4 equiv.) and the mixture was stirred at r.t for 1 h. A saturated $NaHCO_3$ solution (25 mL) was added and the mixture was stirred for 1 h. The mixture was diluted with $CH_2Cl_2$ (25 mL) and $H_2O$ (25 mL) and the layers were separated. The aqueous layer was extracted with $CH_2Cl_2$ (3 x 20 mL) and the combined organic layers

were washed with brine, dried ($Mg_2SO_4$) and filtered. The solvent was removed under reduced pressure and recrystallization from $Et_2O$ gave **S3** (1.1 g, 64%). $^1$H NMR (400 MHz, MeOD) δ 8.30–8.26 (1H, m), 7.97 (1H, d, *J* = 15.5 Hz), 7.76–7.72 (1H, m), 7.72–7.67 (5H, m), 7.52–7.46 (3H, m). Data in accordance with the literature.[3]

**(*E*)-1-(2-cyclohexylvinyl)-1 λ$^3$-benzo[d][1,2]iodaoxol-3(1H)-one (S4)**

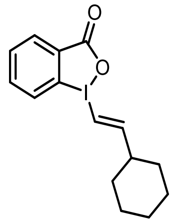


*m*-CPBA (0.8 g, 3.6 mmol, 1.1 equiv.) was added to a solution of 2-iodobenzoic acid (0.8 g, 3.3 mmol, 1.0 equiv.) in $CHCl_2$ (20 mL). The reaction mixture was cooled to 0 ºC and triflic acid (0.3 mL, 3.3 mmol, 1.0 equiv.) was added at this temperature before being stirred at r.t for 15 mins. The reaction mixture was again cooled to 0 ºC (over 10 mins) and *trans*-2-cyclohexylvinylboronic acid (1.0 g, 6.5 mmol, 2.0 equiv.) was added before being stirred at r.t for 1 h. The solvent was removed under reduced pressure and $Et_2O$ (80 mL) was added. After stirring for 30 min, the supernatant was removed and the resultant precipitate was washed with $Et_2O$ (4 x 80 mL). The precipitate was dried under reduced pressure, $CH_2Cl_2$ (16 mL) and a saturated $NaHCO_3$ solution (16 mL) were added and the mixture stirred for 1 h. The mixture was diluted with $CH_2Cl_2$ (20 mL) and $H_2O$ (20 mL) and the layers were separated. The aqueous layer was extracted with $CH_2Cl_2$ (3 x 20 mL) and the combined organic layers were washed with brine, dried ($Mg_2SO_4$) and filtered to give **S4** (0.36 g, 31%). $^1$H NMR (500 MHz, MeOD) δ 8.28 (1H, dd, *J* = 7.3, 5.1 Hz), 7.72–7.66 (3H, m), 7.12 (1H, dd, *J* = 15.1, 7.0 Hz), 6.83 (1H, d, *J* = 15.1 Hz), 2.47 (1H, dtd, *J* = 14.5, 7.1, 3.6 Hz), 1.94 (2H, d, *J* = 12.7 Hz), 1.87–1.81 (2H, m), 1.74 (1H, d, *J* = 12.8 Hz), 1.48–1.23 (5H, m). Data in accordance with the literature.[3]

## 3.2 Synthesis of Ketones

**1-Phenylpent-4-en-1-one (S6)**

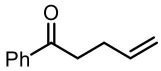

A dry Schlenk tube equipped with a stirring bar was charged with CuI (0.15 equiv.) and then the flask was evacuated and refilled with $N_2$ (x 3). The benzoyl chloride (5.0 ml, 43 mmol, 1.0 equiv.) and THF (0.1M) were added and the mixture was stirred at room temperature for 10 min and then cooled to –78 ºC. A freshly prepared solution of but-3-en-1-ylmagnesium bromide (1.1 equiv.) was added by dropwise [Grignard preparation: a dry Schlenck tube equipped with a stirring bar was charged with Mg turnings (1.1 equiv.) and then the flask was evacuated and refilled with $N_2$ (x 3). The minimum amount of THF was added and then 4-bromobut-1-ene (0.3 equiv.) was added neat. Once the Grignard reaction started the remaining 4-bromobut-1-ene (0.7 equiv.) were added as a solution in THF (0.7M). The corresponding mixture was stirred for 1 additional hour]. The mixture was allowed to warm to room temperature overnight. $NH_4Cl$ was added and the mixture was diluted with $Et_2O$. The layers were separated and the organic layer was dried ($MgSO_4$), filtered and evaporated. Purification by column chromatography on silica gel, eluting with petrol–$Et_2O$ 99:1, gave **S6** (5.5 g, 80%) as an oil. $^1$H NMR (400 MHz, $CDCl_3$) δ 7.97 (2H, d, $J$ = 7.2 Hz), 7.59–7.43 (3H, m), 5.98–5.84 (1H, m), 5.13–4.99 (2H, m), 3.11–3.06 (2H, m), 2.54–2.46 (2H, m); $^{13}$C NMR (101 MHz, $CDCl_3$) δ 199.1, 137.1, 136.8, 132.9, 128.4, 127.9, 115.2, 37.8, 28.2. Data in accordance with the literature.[4]

**General procedure for the synthesis of ketones S7–12**

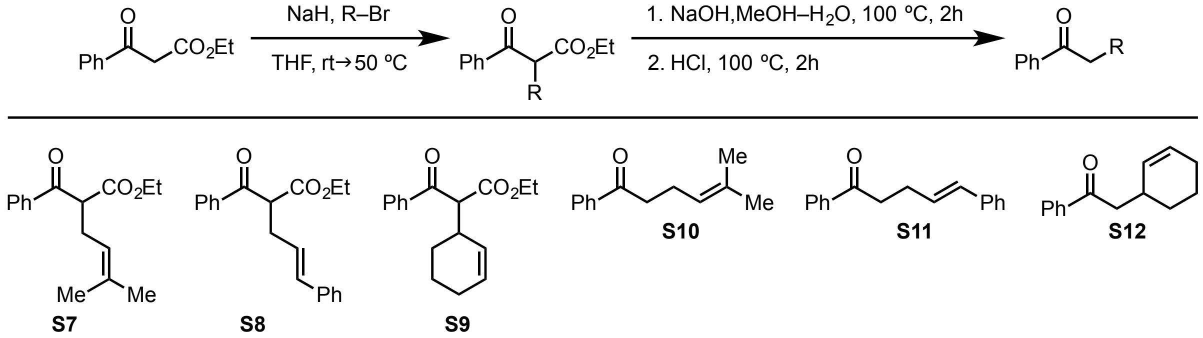

**GP1**

A solution of ethyl 3-oxo-3-phenylpropanoate (1.0 equiv.) in THF (0.17M) was treated with NaH (1.0 equiv., 60% in mineral oil), stirred for 1h and treated with the allylic bromide (1.1 equiv.). The mixture was warmed to 40 ºC and stirred overnight. The mixture was cooled to room temperature and MeOH was added. The crude product was absorbed on silica and purified by column chromatography on silica gel eluting with petrol–$Et_2O$ (95:5) to give the product.

**GP2**

A solution of the β-ketoester (1.0 equiv.) in MeOH–$H_2O$ (0.01M, 2:1) was treated with NaOH (4.0 equiv.) and heated under reflux for 20 h (at this point GCMS analysis revealed complete hydrolysis and subsequent decarboxylation of the ester). The mixture was cooled to room temperature and the MeOH was removed *in vaco*. EtOAc was added and the layers were separated. The aqueous layer was then washed with EtOAc (x3) and the combined organic fractions were dried ($MgSO_4$), filtered and evaporated. Purification by column chromatography on silica gel, eluting with petrol-$Et_2O$ 99:1, gave the ketone.

**Ethyl 2-Benzoyl-5-methylhex-4-enoate (S7)**

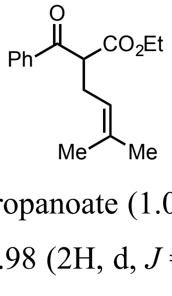

Following **GP1**, ethyl 3-oxo-3-phenylpropanoate (1.0 g, 5.2 mmol) gave **S7** (1.34 g, 99%) as an oil. $^1$H NMR (400 MHz, $CDCl_3$) δ 7.98 (2H, d, $J$ = 7.8 Hz), 7.58 (1H, t, $J$ = 7.4 Hz), 7.47 (2H, t, $J$ = 7.5 Hz), 5.10 (1H, t, $J$ = 7.2 Hz), 4.29 (1H, t, $J$ = 7.3 Hz), 4.13 (2H, q, $J$ = 7.1 Hz), 2.77–2.60 (2H, m), 1.65 (3H, s, $J$ = 7.4 Hz), 1.62 (3H, s), 1.16 (3H, t, $J$ = 7.1 Hz). Data in accordance with the literature.[5]

**Ethyl (*E*)-2-benzoyl-5-phenylpent-4-enoate (S8)**

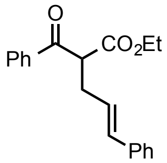


Following **GP1**, ethyl 3-oxo-3-phenylpropanoate (1.0 g, 5.2 mmol) gave **S8** (1.31 g, 81%) as an oil. $^{1}$H NMR (400 MHz, $CDCl_3$) δ 7.96 (2H, d, *J* = 12 Hz), 7.12–7.56 (8H, m), 6.42 (1H, d, *J* = 15.5 Hz), 6.18 (1H, ddd, *J* = 15.5, 8.0, 8.0 Hz), 4.41 (1H, t, *J* = 8.0 Hz), 4.11 (2H, q, *J* = 7.5 Hz), 2.86 (2H, m), 1.12 (3H, t, *J* = 7.5 Hz); $^{13}$C NMR (101 MHz, $CDCl_3$) δ 194.1, 169. 1, 136.8, 136.0, 133.3, 132.4, 128.5, 128.4, 128.4, 128.3, 128.0, 127.1, 125.9, 125.9, 61.33, 54.2, 32.3, 14.0. Data in accordance with the literature.[6]

**Ethyl 2-(Cyclohex-2-en-1-yl)-3-oxo-3-phenylpropanoate (S9)**

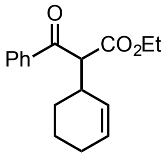


Following **GP1**, ethyl 3-oxo-3-phenylpropanoate (1.0 g, 5.2 mmol) gave **S9** (1.34 g, 99%) as an oil. $^{1}$H NMR (400 MHz, $CDCl_3$) δ 7.96 (2H, d, *J* = 12 Hz), 7.12–7.56 (8H, m), 6.42 (1H, d, *J* = 15.5 Hz), 6.18 (1H, ddd, *J* = 15.5, 8.0, 8.0 Hz), 4.41 (1H, t, *J* = 8.0 Hz), 4.11 (2H, q, *J* = 7.5 Hz), 2.86 (2H, m), 1.12 (3H, t, *J* = 7.5 Hz); $^{13}$C NMR (101 MHz, $CDCl_3$) δ 194.1, 169. 1, 136.8, 136.0, 133.3, 132.4, 128.5, 128.4, 128.4, 128.3, 128.0, 127.1, 125.9, 125.9, 61.33, 54.2, 32.3, 14.0. Data in accordance with the literature.[7]

**5-Methyl-1-phenylhex-4-en-1-one (S10)**

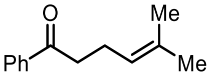


Following **GP2**, **S7** (1.34 g, 5.1 mmol) gave **S10** (1.0 g, 99%) as an oil. $^{1}$H NMR (400 MHz, $CDCl_3$) δ 7.96 (2H, d, *J* = 7.8 Hz), 7.55 (1H, t, *J* = 7.3 Hz), 7.46 (2H, t, *J* = 7.4 Hz), 5.17 (1H, t, *J* = 7.1 Hz), 3.00 (2H, t, *J* = 7.5 Hz), 2.42 (2H, q, *J* = 7.4 Hz), 1.69 (3H, s), 1.63 (3H, s). Data in accordance with the literature.[5]

**(*E*)-1,5-Diphenylpent-4-en-1-one (S11)**

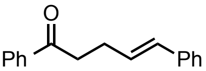


Following **GP2**, **S8** (1.31 g, 4.3 mmol) gave **S11** (1.0 g, 97%) as an oil. $^{1}$H NMR (400 MHz,

$CDCl_3$) δ 7.98 (2H, dt, *J* = 7.8, 1.3 Hz), 7.57 (1H, tt, *J* = 7.8, 1.5 Hz), 7.47 (2H, t, *J* = 7.9 Hz), 7.34 (2H, d, *J* = 7.1 Hz), 7.29 (2H, t, *J* = 7.5 Hz), 7.22 (1H, tt, *J* = 7.5, 1.3 Hz), 6.50 (1H, d, *J* = 16.3 Hz), 6.33 (1H, dt, *J* = 16.3, 7.4 Hz), 3.17 (2H, t, *J* = 7.4 Hz), 2.64 (2H, qd, *J* = 7.3, 1.3 Hz); $^{13}$C NMR (101 MHz, $CDCl_3$) δ 199.3, 137.5, 137.2, 133.1, 130.5, 129.2, 128.4, 128.5, 128.1, 127.3, 126.1, 38.5, 27.5. Data in accordance with the literature.[8]

**2-(Cyclohex-2-en-1-yl)-1-phenylethan-1-one (S12)**

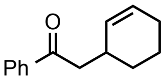


Following **GP2**, **S9** (442 mg, 1.61 mmol) gave **S12** (266 mg, 82%) as an oil. $^1$H NMR (400 MHz, $CDCl_3$) δ 7.96 (2H, d, *J* = 7.2 Hz), 7.59–7.42 (3H, m), 5.75–5.70 (1H, m), 5.62–5.57 (1H, m), 2.97–2.74 (3H, m), 2.04–1.50 (5H, m), 1.37–1.24 (1H, m); $^{13}$C NMR (101 MHz, $CDCl_3$) δ 191.3, 137.2, 133.0, 130.8, 128.6, 128.1, 128.0, 44.8, 31.6, 29.1, 25.1, 21.1. Data in accordance with the literature.[9]

**Methyl 6-Methyl-2-oxohept-5-enoate (S13)**

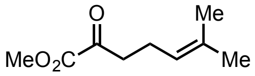


A dry Schlenk tube equipped with a stirring bar was charged with dimethyl oxalate (0.5 g, 4.2 mmol, 1.0 equiv.) and then the flask was evacuated and refilled with $N_2$ (x 3). Dry THF (0.7 M) was added and the mixture was cooled to –78 ºC. A freshly prepared solution of 4-methylpent-3-en-1-yl)magnesium bromide (1.05 equiv.) was added by dropwise [Grignard preparation: a dry Schlenck tube equipped with a stirring bar was charged with Mg turnings (1.1 equiv.) and then the flask was evacuated and refilled with $N_2$ (x 3). The minimum amount of THF was added and then 5-bromo-2-methylpent-2-ene (0.3 equiv.) was added neat. Once the Grignard reaction started the remaining 5-bromo-2-methylpent-2-ene (0.75 equiv.) were added as a solution in THF (2 M). The corresponding mixture was stirred for 1 additional hour]. The mixture was allowed to warm to room temperature overnight. $NH_4Cl$ was added and the mixture was diluted with $Et_2O$. The layers were separated and the organic layer was dried ($MgSO_4$), filtered and evaporated. Purification by column chromatography on silica gel, eluting with petrol–$Et_2O$ 95:5, gave **S13** (0.2 g, 25%) as an oil, $^1$H NMR (400 MHz, $CDCl_3$) δ 5.07 (1H, t, $J$ = 7.2 Hz), 3.86 (3H, s), 2.87 (2H, t, $J$ = 7.3 Hz), 2.32 (2H, q, $J$ = 7.3 Hz), 1.67 (3H, s), 1.62 (3H, s); $^{13}$C NMR (101 MHz, $CDCl_3$) δ 194.1, 161.6, 133.7, 121.9, 53.0, 39.7, 25.8, 21.9, 17.8; HRMS (ESI): Found $MNH_4^+$ 188.1280 $C_9H_{18}O_3N$ requires 188.1281.

**1-(Pyridin-3-yl)pent-4-en-1-one (S15)**

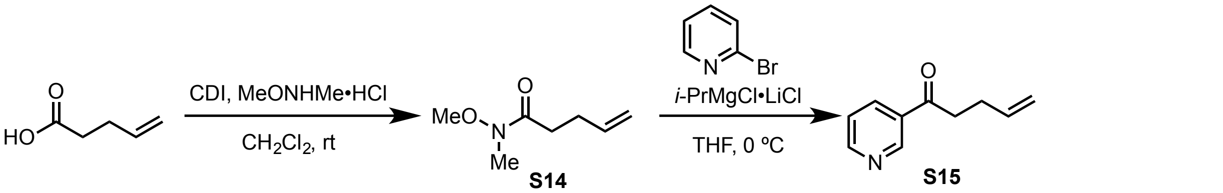


A solution of 4-pentanoic acid (1.8 mL, 18 mmol, 1.0 equiv.) in $CH_2Cl_2$ (180 mL) was cooled to 0 °C, treated with carbonyl diimidazole (3.9 g, 22 mmol, 1.2 equiv.) and stirred at the same temperature for 30 min. *N*-*O*-Dimethylhydroxylamine hydrogenchloride (4.9 g, 45 mmol, 2.5 equiv.) was added and the mixture was allowed to warm to room temperature overnight. $H_2O$ (50 mL) was added and the layers separated. The aqueous layer was extracted with $CH_2Cl_2$ (3 x 20 mL). The organic layers were collected, dried ($MgSO_4$), filtered and evaporated. Purification by column chromatography column chromatography on silica gel gave **S14** as an oil (2.6 g, 99%). $^1$H NMR (400 MHz, $CDCl_3$) δ 5.86 (1H, ddt, $J$ = 16.7, 10.2, 6.5 Hz), 5.09

(1H, dd, $J$ = 17.1, 1.6 Hz), 4.99 (1H, dd, $J$ = 10.3, 1.4 Hz), 3.68 (3H, s), 3.18 (3H, s), 2.53 (2H, t, $J$ = 7.4 Hz), 2.41 (2H, q, $J$ = 7.4 Hz). Data in accordance with literature.[10]

A dry Schlenk tube equipped with a stirring bar was charged with 2-bromopyridine (0.60 mL, 6.3 mmol, 1 equiv.) and dry THF (6.2 mL). The solution was cooled to 0 °C and *i*-PrMgCl•LiCl (4.9 mL, 6.3 mmol, 1 equiv., 1.3 M in THF) was added dropwise. The mixture was stirred at 0 °C for 4 h and then **S14** (0.9 g, 6.3 mmol, 1 equiv.) was added. The mixture was allowed to warm to room temperature overnight and then $H_2O$ (10 mL) and $Et_2O$ (20 mL) were added. The layers were separated and the aqueous layer was extracted with $Et_2O$ (3 x 20 mL). The combined organic layers were dried ($MgSO_4$), filtered and evaporated. Purification by column chromatography on silica gel, gave **S15** as an oil. $^1$H NMR (400 MHz, $CDCl_3$) δ 9.18 (1H, d, $J$ = 1.5 Hz), 8.78 (1H, dd, $J$ = 4.8, 1.6 Hz), 8.24 (1H, dt, $J$ = 8.0, 1.9 Hz), 7.43 (1H, dd, $J$ = 7.9, 4.8 Hz), 5.90 (1H, ddt, $J$ = 16.8, 10.2, 6.5 Hz), 5.10 (1H, dd, $J$ = 17.1, 1.5 Hz), 5.03 (1H, dd, $J$ = 10.2, 1.2 Hz), 3.10 (2H, t, $J$ = 7.3 Hz), 2.52 (2H, q, $J$ = 6.9 Hz); $^{13}$C NMR (101 MHz, $CDCl_3$) δ 198.4, 153.6, 149.7, 136.9, 135.5, 132.2, 123.8, 115.9, 38.2, 27.9. Data in accordance with the literature.[11]

**Methyl (*E*)-6-Oxo-6-phenylhex-2-enoate (S16)**

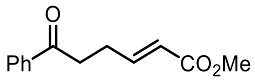


**S6** (1.00 g, 6.3 mmol, 1.0 equiv) was added to an oven dried multi-necked flask and put under $N_2$. Dry $CH_2Cl_2$ (100 ml, degassed by x3 freeze-pump-thaw cycles) was added followed by Grubbs Catalyst 2$^{nd}$ Generation (0.27 g, 0.3 mmol, 0.05 equiv.) and freshly distilled methyl acrylate (2.8 ml, 31.3 mmol, 5.0 equiv.). The solution was then stirred until complete consumption of **S6** as determined by TLC analysis. The crude mixture was then absorbed onto silica and purified by column chromatography (petrol:EtOAc 95:5→90:10) to give **S16** (1.1 g, 83 %). $^1$H NMR (500 MHz, $CDCl_3$) δ 7.96 (2H, d, $J$ = 7.9 Hz), 7.58 (1H, t, $J$ = 7.3 Hz), 7.47 (2H, t, $J$ = 7.7 Hz), 7.04 (1H, dt, $J$ = 15.5, 6.8 Hz), 5.90 (1H, d, $J$ = 15.7 Hz), 3.72 (3H, s), 3.15 (2H, t, $J$ = 7.2 Hz), 2.69–2.63 (2H, m). Data in accordance with the literature.[12]

**1-((4*R*,4a*R*,7*R*,12b*S*,14*S*)-7,9-Dimethoxy-3-methyl-1,2,3,4,7,7a-hexahydro-7,4a-ethano-4,12-methanobenzofuro[3,2-e]isoquinolin-14-yl)ethan-1-one – Thevinone (9)**

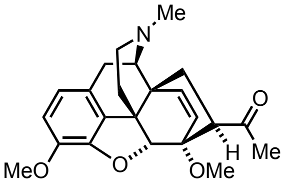


Thebaine (0.5 g, 1.61 mmol, 1.0 equiv.) and freshly distilled methyl vinyl ketone (1.5 mL, 30.6 mmol, 19.0 equiv.) were refluxed for 16 h. The reaction mixture was cooled to r.t. and the excess methyl vinyl ketone was removed under high vacuum. Purification by column chromatography on silica gel eluting with $CH_2Cl_2 \rightarrow CH_2Cl_2$:MeOH (98:2) gave thevinone **9** (0.60 g, 98%) as a solid. $^1$H NMR (400 MHz, $CDCl_3$) δ 6.62 (1H, d, *J* = 8.1 Hz), 6.53 (1H, d, *J* = 8.1 Hz), 5.90 (1H, d, *J* = 8.8 Hz), 5.57 (1H, d, *J* = 8.8 Hz), 4.57 (1H, s), 3.81 (3H, s), 3.59 (3H, s), 3.21 (2H, t, *J* = 11.2 Hz), 2.96–2.88 (2H, m), 2.51 (1H, dd, *J* = 11.9, 5.2 Hz), 2.44 (1H, d, *J* = 6.4 Hz), 2.39 (1H, d, *J* = 6.9 Hz), 2.36 (3H, s), 2.13 (3H, s), 1.96 (1H, td, *J* = 12.6, 5.5 Hz), 1.84 (1H, d, *J* = 11.7 Hz), 1.36 (1H, dd, *J* = 15.7, 11.5 Hz); $^{13}$C NMR (101 MHz, $CDCl_3$) δ 209.2, 148.1, 142.0, 136.0, 134.1, 128.3, 126.2, 119.5, 113.6, 95.3, 81.4, 60.1, 56.7, 53.6, 50.8, 47.6, 45.6, 43.6, 43.3, 33.6, 30.7, 30.1, 22.5. Data in accordance with the literature.[13]

## 3.3 Synthesis of Oximes 3a–h

## 3.4 General procedure for the synthesis of oximes – GP3

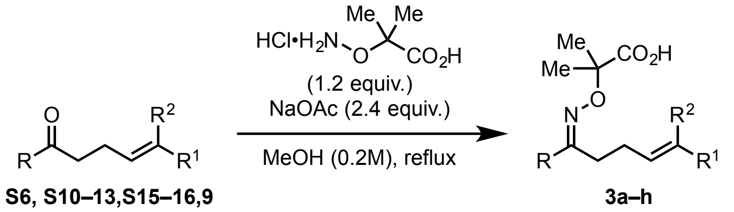

A solution of ketone (1.0 equiv.) in MeOH (0.2 M) was treated with 1-carboxy-1-methylethoxyammonium chloride (1.2 equiv.), anhydrous NaOAc (2.4 equiv.) and heated to reflux until complete by TLC analysis (3-6 h). The mixture was allowed to cool to room temperature and an aqueous $K_2CO_3$ solution was added. This solution was extracted with $Et_2O$ and the organic layer washed with aqueous $K_2CO_3$ solution (x 2). The combined aqueous extractions were then acidified with conc. HCl solution (30% $H_2O$) and extracted with $CH_2Cl_2$ (x 3). The combined organic fractions were dried ($MgSO_4$), filtered and evaporated.

**2-Methyl-2-(((5-methyl-1-phenylhex-4-en-1-ylidene)amino)oxy)propanoic acid (3a)**

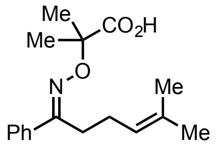

Following **GP3**, **S10** (1.0 g, 5.32 mmol) gave **3a** (1.53 g, 99%) as a solid. FT-IR $\nu_{max}$ (film)/cm$^{-1}$ 2987, 1704, 1471, 1449, 1419, 1363, 1301, 1172; $^1$H NMR (400 MHz, $CDCl_3$) δ 7.61 (2H, d, *J* = 6.9 Hz), 7.43–7.35 (3H, m), 5.16 (1H, t, *J* = 7.3 Hz), 2.83 (2H, t, *J* = 7.8 Hz), 2.25 (2H, q, *J* = 7.6 Hz), 1.67 (3H, s), 1.61 (6H, s), 1.55 (3H, s); $^{13}$C NMR (101 MHz, $CDCl_3$) δ 176.6, 161.1, 134.9, 133.4, 130.0, 128.8, 126.6, 122.9, 81.9, 27.2, 25.8, 25.2, 24.5, 17.7; HRMS (ESI): Found MH+ 290.1748 $C_{17}H_{24}O_3N$ requires 290.1751.

**2-Methyl-2-(((1-phenylpent-4-en-1-ylidene)amino)oxy)propanoic acid (3b)**

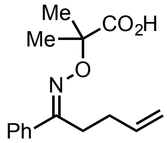

Following **GP3**, **S6** (0.75 g, 4.69 mmol) gave **3b** (1.2 g, 98%) as a solid. FT-IR $\nu_{max}$ (film)/cm$^{-1}$ 2941, 1706, 1453, 1441, 1381, 1366, 1310, 1221, 1178, 1016; $^1$H NMR (400 MHz, $CDCl_3$) δ 7.60 (2H, d, *J* = 7.8 Hz), 7.42–7.35 (3H, m), 5.90–5.78 (1H, m), 5.04 (1H, d,

$J$ = 17.4 Hz), 5.00 (1H, d, $J$ = 11.2 Hz), 2.91 (2H, t, $J$ = 7.7 Hz), 2.33 (2H, q, $J$ = 7.3 Hz), 1.61 (6H, s); $^{13}$C NMR (101 MHz, $CDCl_3$) δ 176.7, 160.6, 137.3, 134.9, 130.0, 128.8, 126.7, 115.7, 82.0, 30.6, 26.4, 24.5; HRMS (ESI): Found $MH^+$ 262.1435 $C_{15}H_{20}O_3N$ requires 262.1438.

**2-Methyl-2-(((1-(pyridin-3-yl)pent-4-en-1-ylidene)amino)oxy)propanoic acid (3c)**

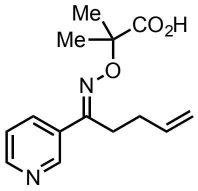


Following **GP3**, **S15** (0.16 g, 1.00 mmol) gave **3c** (0.24 g, 90%) as an oil. FT-IR $\nu_{max}$ (film)/cm$^{-1}$ 2985, 1720, 1641, 1583, 1565, 1468, 1434, 1362, 1286, 1152; $^1$H NMR (500 MHz, $CDCl_3$) δ 8.81 (1H, s, br), 8.64 (1H, s), 7.83 (1H, d, $J$ = 7.9 Hz), 7.69 (1H, t, $J$ = 7.3 Hz), 7.30 (1H, d, $J$ = 4.5 Hz), 5.86 (1H, ddt, $J$ = 16.8, 10.1, 6.6 Hz), 5.01 (1H, d, $J$ = 17.1 Hz), 4.93 (1H, d, $J$ = 10.1 Hz), 3.09 (2H, t, $J$ = 7.6 Hz), 2.35 (2H, q, $J$ = 7.1 Hz), 1.63 (6H, s). $^{13}$C NMR (126 MHz, $CDCl_3$) δ 177.8, 159.5, 152.9, 148.4, 137.9, 137.3, 124.2, 121.7, 115.2, 82.3, 30.5, 24.8, 24.3. HRMS (ESI): Found $MH^+$ 263.1384 $C_{14}H_{19}O_3N_2$ requires 263.1390.

**2-((((*E*)-1,5-diphenylpent-4-en-1-ylidene)amino)oxy)-2-methylpropanoic Acid (3d)**

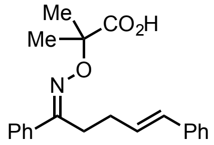


Following **GP3**, **S11** (0.71 g, 6.0 mmol) gave **3d** (0.97 g, 96%) as a solid. FT-IR $\nu_{max}$ (film)/cm$^{-1}$ 2985, 1712, 1496, 1469, 1449, 1362, 1300, 1169; $^1$H NMR (400 MHz, $CDCl_3$) δ 7.69–7.63 (2H, m), 7.41 (3H, m), 7.36–7.28 (4H, m), 7.22 (1H, t, $J$ = 6.6 Hz), 6.43 (1H, d, $J$ = 15.8 Hz), 6.31–6.21 (1H, dt, $J$ = 15.8, 6.8 Hz), 3.01 (2H, t, $J$ = 7.7 Hz), 2.52 (2H, q, $J$ = 7.3 Hz), 1.63 (6H, s); $^{13}$C NMR (126 MHz, $CDCl_3$) δ 177.6, 160.0, 137.5, 135.1, 130.9, 129.9, 129.2, 128.8, 128.7, 127.3, 126.7, 126.2, 81.9, 30.0, 26.9, 24.5. HRMS (ESI): Found $MNa^+$ 360.1566 $C_{21}H_{23}O_3NNa$ requires 360.1570.

**2-((((*E*)-6-Methoxy-6-oxo-1-phenylhex-4-en-1-ylidene)amino)oxy)-2-methylpropanoic Acid (3e)**

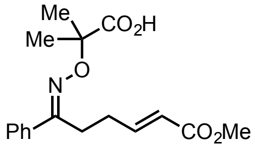

Following **GP3**, **S16** (0.70 g, 3.2 mmol) gave **3e** (1.00 g, 99%) as a solid. FT-IR $\nu_{max}$ (film)/cm$^{-1}$ 2951, 1704, 1642, 1451, 1438, 1306, 1276, 1240, 1172, 1041, 1017 $^1$H NMR (400 MHz, $CDCl_3$) δ 7.59 (2H, d, *J* = 7.0 Hz), 7.43–7.34 (3H, m), 6.98 (1H, dt, *J* = 15.6, 7.5 Hz), 5.84 (1H, d, *J* = 15.6 Hz), 3.72 (3H, s), 2.96 (2H, t, *J* = 7.6 Hz), 2.47 (2H, q, *J* = 7.2 Hz), 1.61 (6H, s). $^{13}$C NMR (101 MHz, $CDCl_3$) δ 177.2, 167.1, 158.8, 147.7, 134.6, 130.0, 128.8, 126.5, 121.7, 82.0, 51.7, 29.1, 25.2, 24.3; HRMS (ESI): Found $MH^+$ 320.1487 $C_{17}H_{22}O_5N$ requires 320.1492.

**2-(((2-(Cyclohex-2-en-1-yl)-1-phenylethylidene)amino)oxy)-2-methylpropanoic Acid (3f)**

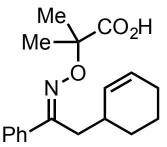

Following **GP3**, **S12** (0.40 g, 2.0 mmol) gave **3f** (0.52 g, 86%) as a solid. FT-IR $\nu_{max}$ (film)/cm$^{-1}$ 2922, 1719, 1697, 1471, 1444, 1295, 1211, 1168; $^1$H NMR (500 MHz, $CDCl_3$) δ 7.65–7.59 (2H, m), 7.42–7.34 (3H, m), 5.73–5.66 (1H, m), 5.54 (1H, dd, *J* = 10.0, 1.6 Hz), 2.89 (1H, dd, *J* = 13.0, 8.9 Hz), 2.79 (1H, dd, *J* = 13.0, 6.7 Hz), 2.44 (1H, br s), 1.97 (2H, s, br), 1.75–1.65 (2H, m), 1.60 (3H, s), 1.59 (3H, s), 1.52–1.44 (1H, m), 1.37–1.29 (1H, m); $^{13}$C NMR (126 MHz, $CDCl_3$) δ 177.0, 160.0, 135.3, 130.6, 129.9, 128.7, 128.1, 126.8, 82.0, 33.3, 32.7, 28.8, 25.2, 24.5, 24.5, 21.0; HRMS (ESI): Found $MH^+$ 302.1740 $C_{18}H_{24}O_3N$ requires 302.1751.

**2-(((1-Methoxy-6-methyl-1-oxohept-5-en-2-ylidene)amino)oxy)-2-methylpropanoic Acid (3g)**

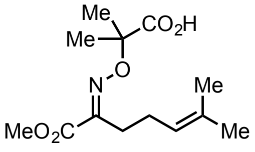

Following **GP3**, **S13** (0.2 g, 1.2 mmol) gave **3g** (0.32 g, 100%) as an oil. FT-IR $\nu_{max}$ (film)/cm$^{-1}$ 2930, 1720, 1440, 1365, 1323, 1285, 1167, 1120; $^1$H NMR (400 MHz, $CDCl_3$) δ

5.13 (1H, t, $J$ = 7.1 Hz), 3.81 (3H, s), 2.61 (2H, t, $J$ = 7.5 Hz), 2.20 (2H, q, $J$ = 7.5 Hz), 1.67 (3H, s), 1.60 (9H, m); $^{13}$C NMR (101 MHz, $CDCl_3$) δ 178.5, 164.2, 153.3, 133.2, 122.9, 82.6, 52.7, 26.0, 25.8, 24.7, 24.2, 17.6; HRMS (ESI): Found $MH^+$ 272.1488 $C_{13}H_{22}O_5N$ requires 272.1492.

**2-(((1-((4*R*,4a*R*,7*R*,12b*S*,14*R*)-7,9-Dimethoxy-3-methyl-1,2,3,4,7,7a-hexahydro-7,4a-ethano-4,12-methanobenzofuro[3,2-e]isoquinolin-14-yl)ethylidene)amino)oxy)-2-methylpropanoic Acid (3h)**

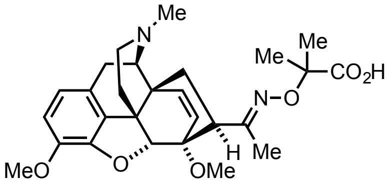


1-Carboxy-1-methylethoxyammonium chloride (270 mg, 1.7 mmol, 1.2 equiv.) and pyridine (0.3 mL, 3.4 mmol, 2.4 equiv.) were added to **9** (0.60 g, 1.60 mmol, 1.0 equiv.) in MeOH (0.2 M) and stirred overnight at r.t. The solvent was then removed under reduced pressure and purification by column chromatography on silica gel, eluting with $CH_2Cl_2$→ $CH_2Cl_2$:MeOH (98:2) gave **3h** (0.53 g, 69%) as a solid. FT-IR $\nu_{max}$ (film)/cm$^{-1}$ 2936, 1716, 1599, 1501, 1441, 1379, 1266, 1208, 1167, 1108; $^1$H NMR (500 MHz, $CDCl_3$) δ 6.63 (1H, d, $J$ = 8.1 Hz), 6.54 (1H, d, $J$ = 8.1 Hz), 5.78 (1H, d, $J$ = 8.7 Hz), 5.49 (1H, d, $J$ = 8.7 Hz), 4.67 (1H, s), 3.81 (3H, s), 3.51 (3H, s), 3.28 (1H, d, $J$ = 5.7 Hz), 3.24 (1H, d, $J$ = 18.7 Hz), 3.03 (1H, dd, $J$ = 12.5, 10.0 Hz), 2.93–2.86 (1H, m), 2.68 (1H, dd, $J$ = 10.9, 3.4 Hz), 2.52–2.44 (2H, m), 2.42 (3H, s), 2.08 (1H, td, $J$ = 12.8, 5.2 Hz), 1.88 (1H, dd, $J$ = 13.1, 1.9 Hz), 1.77 (3H, s), 1.48 (3H, s, $J$ = 6.6 Hz), 1.47 (3H, s), 1.25 (1H, dd, $J$ = 12.9, 6.8 Hz); $^{13}$C NMR (126 MHz, $CDCl_3$) δ 177.2, 160.5, 148.4, 142.2, 135.1, 133.9, 128.5, 127.7, 119.5, 113.6, 92.9, 81.4, 80.3, 60.2, 56.7, 51.9, 47.5, 45.7, 43.4, 43.1, 42.9, 33.4, 30.7, 24.6, 24.4, 22.8, 12.8; HRMS (APCI): Found $MH^+$ 483.2487 $C_{27}H_{35}O_6N_2$ requires 483.2490.

# 4 Electrochemical studies

## 4.1 Synthesis of Substrates

### (*E*)-2-(((1-Phenylethylidene)amino)oxy)acetic Acid (2a)

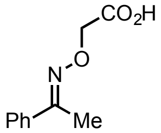


A solution of acetophenone (0.10 g, 0.83 mmol, 1.0 equiv.) in MeOH (0.2 M) was treated with O-(Carboxymethyl)hydroxylamine hemihydrochloride (0.18 g, 1.66 mmol, 2.0 equiv.), anhydrous NaOAc (0.10 g, 1.25 mmol, 1.5 equiv.) and heated to reflux for 90 mins. The mixture was allowed to cool to room temperature and an aqueous $K_2CO_3$ solution was added. This solution was extracted with $Et_2O$ and the organic layer washed with aqueous $K_2CO_3$ solution (x 2). The combined aqueous extractions were then acidified with conc. HCl solution (30% $H_2O$) and extracted with $CH_2Cl_2$ (x 3). The combined organic fractions were dried ($MgSO_4$), filtered and evaporated to give **2a** (0.1 g, 0.83 mmol, 1.0 equiv.) as a solid. $^1$H NMR (500 MHz, $CDCl_3$) δ 7.58–7.55 (2H, m), 7.33–7.29 (3H, m), 4.71 (2H, s), 2.26 (3H, s); $^{13}$C NMR (101 MHz, $CDCl_3$) δ 174.0, 157.8, 135.8, 129.8, 128.6, 126.5, 70.4, 13.3. This compound has been reported in the literature[14] but spectroscopic data was not provided.

### (*E*)-2-(((1-Phenylethylidene)amino)oxy)propanoic Acid (2b)

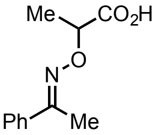


A solution of acetophenone oxime (135 mg, 1.0 mmol, 1.0 equiv.) in dry DMF (0.2M) under nitrogen was cooled to 0 °C and treated with NaH (80 mg, 2.0 mmol, 2.0 equiv., 60% dispersion in mineral oil). The mixture was stirred at this temperature for 30 minutes before 2-bromopropanoic acid (98 µL, 1.1 mmol, 1.1 equiv.) was added and stirred overnight at r.t. The mixture was diluted with $H_2O$ and $Et_2O$. The layers were separated and the aqueous layer washed with $Et_2O$ (x 2). The combined organic extractions were then were dried ($MgSO_4$), filtered and evaporated. Purification by column chromatography gave **2b** as a solid (70 mg, 34%). FT-IR $\nu_{max}$ (film)/cm$^{-1}$ 2922, 2851, 1739, 1462, 1447, 1369, 1210, 1151, 1098, 1045; $^1$H NMR (400 MHz, $CDCl_3$) δ 7.65–7.61 (2H, m), 7.40–7.35 (3H, m), 4.86 (1H, q, *J* = 7.1 Hz), 2.32 (3H, s), 1.60 (3H, d, *J* = 7.1 Hz); $^{13}$C NMR (101 MHz, $CDCl_3$) δ 176.8, 157.3, 135.9, 129.8, 128.6, 126.4, 77.3, 17.0, 13.3; HRMS (ESI): Found $MH^+$ 208.0964 $C_{11}H_{14}O_3N$ requires 208.0968.

**(*E*)-2-Phenyl-2-(((1-phenylethylidene)amino)oxy)acetic Acid (2c)**

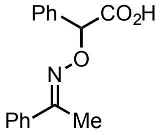


A solution of acetophenone oxime (135 mg, 1.0 mmol, 1.0 equiv.) in dry DMF (0.2M) under nitrogen was cooled to 0 °C and treated with NaH (80 mg, 2.0 mmol, 2.0 equiv., 60% dispersion in mineral oil). The mixture was stirred at this temperature for 30 minutes before 2 2-bromo-2-phenylacetic acid (237 mg, 1.1 mmol, 1.1 equiv.) was added and stirred overnight at r.t. The mixture was diluted with $H_2O$ and $Et_2O$. The layers were separated and the aqueous layer washed with $Et_2O$ (x 2). The combined organic extractions were then were dried ($MgSO_4$), filtered and evaporated. Purification by column chromatography gave **2c** as a solid (75 mg, 28%). FT-IR $\nu_{max}$ (film)/cm$^{-1}$ 2926, 1720, 1701, 1495, 1455, 1442, 1412, 1367, 1311, 1265, 1235, 1186, 1115, 1065; $^1$H NMR (400 MHz, $CDCl_3$) δ 7.66–7.61 (2H, m), 7.57–7.53 (2H, m), 7.41–7.34 (6H, m), 5.75 (1H, s), 2.36 (3H, s); $^{13}$C NMR (101 MHz, $CDCl_3$) δ 175.1, 157.7, 135.9, 134.7, 129.7, 129.3, 128.9, 128.6, 127.8, 126.5, 83.3, 13.5; HRMS (ESI): Found $MH^+$ 270.1119 $C_{16}H_{16}O_3N$ requires 270.1125.

**(*E*)-2-Methyl-2-(((1-phenylethylidene)amino)oxy)propanoic Acid (2d)**

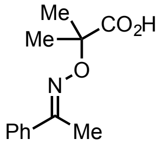


Following **GP3**, acetophenone (50 mg, 0.42 mmol) gave **2d** (92 mg, quantitative) as a solid. FT-IR $\nu_{max}$ (film)/cm$^{-1}$ 2989, 1713, 1470, 1445, 1367, 1306, 1261, 1168, 1088; $^1$H NMR (400 MHz, $CDCl_3$) δ 7.65–7.61 (2H, m), 7.41–7.36 (3H, m), 2.31 (3H, s), 1.61 (6H, s); $^{13}$C NMR (101 MHz, $CDCl_3$) δ 177.3, 157.2, 135.9, 129.9, 128.7, 126.4, 81.9, 24.5, 13.1. HRMS (ASAP): Found $MH^+$ 222.1117 $C_{12}H_{16}O_3N$ requires 222.1125.

## 4.2 General Experimental Detail

Cyclic voltammetry was conducted on an Autolab PGSTAT100 (Metrohm) using a 3-electrode cell configuration. A glassy carbon working electrode was employed alongside a platinum flag counter electrode and a silver pseudo-reference electrode. Ferrocene solution was added as an internal standard to determine the precise potential scale.[15] These values were converted to a saturated calomel electrode (SCE) scale. 5 mM oxime solutions were freshly prepared in acetonitrile along with 0.1 M supporting electrolyte (tetrabutylammonium tetrafluoroborate). Nitrogen was passed through the sample between measurements to avoid the deleterious influence of oxygen reduction, either directly or through indirect reaction with the oxime-derived species. Samples were examined at 8 different scan rates 0.05 V $s^{-1}$–1.00 V $s^{-1}$. The fragmented species reacted too rapidly to produce an oxidation peak on reversal of the potential sweep direction for all except oxime **c** in Scheme 3. Without the presence of an oxidation peak, or other kinetic information about the species formed, the formal reduction potential can only be estimated to within ~50 mV. As a result, we have used the $E_p$max (potential corresponding to the maximum reductive current in the voltammogram from the fastest scan-rate, 1 V $s^{-1}$).[16]

## 4.3 Electrochemical studies

We have performed electrochemical studies to identify the oxidation potential of our oxime starting materials as well as the impact of other factors like counter ion and solvent.

Using oxime **2d**, we have evaluated different solvents and found that in $CH_2Cl_2$ the oxidation potential (vs SCE) is much higher.

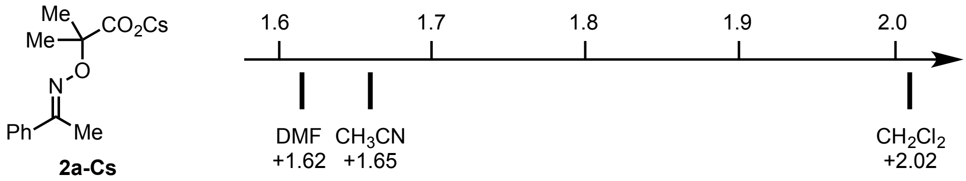


In the photoredox decarboxylation, the use of Cs counter ion has frequently a beneficial effect. We did not observe significant variation in oxidation potential using other inorganic counter ions.

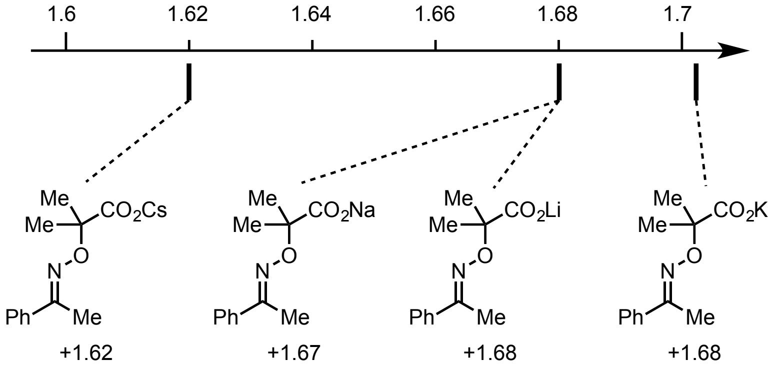


We have tried to determine the $E_{1/2}^{ox}$ of the free carboxylic acids **2a** and **2d** but their oxidation potentials were not observed before solvent oxidation.

We have determined the $E_{1/2}^{red}$ for all the reagents X–Y used in this manuscript. In this case the solvent was sometimes changed from $CH_3CN$ to DMF in order to ensure full solubility of the sample.

## 4.4 Electrochemical Potentials

| Substrate | Solvent | $E_{1/2}^{ox}$ (V) vs SCE |
|---|---|---|
| | *Oximes* | |
| 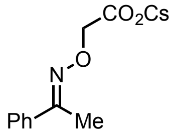 **2a**-Cs | DMF | +2.10 |
| 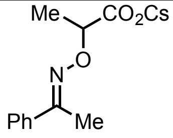 **2b**-Cs | DMF | +1.85 |
| 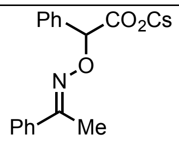 **2c**-Cs | DMF | +1.71 |
| 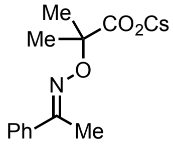 **2d**-Cs | DMF | +1.62 |
| **2d**-Cs | MeCN | +1.60 |
| **2d**-Cs | $CH_2Cl_2$ | +2.02 |
| 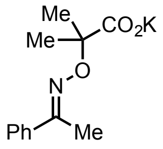 **2d**-K | DMF | +1.68 |
| 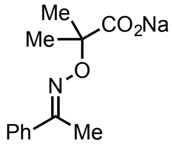 **2d**-Na | DMF | +1.67 |
| 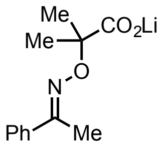 **2d**-Li | DMF | +1.67 |

| Substrate | Solvent | $E_{1/2}^{ox}$ (V) vs SCE |
|---|---|---|
| X–Y reagents | | |
| 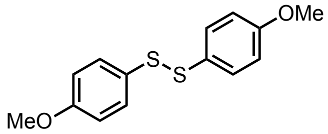 | MeCN | –1.28 |
| 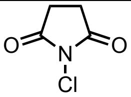 | MeCN | –0.88[1] |
| 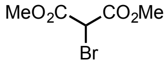 | MeCN | –0.62[17] |
| 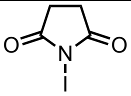 | MeCN | –0.04 |
| 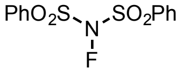 | MeCN | –0.63 |
| 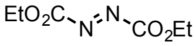 | MeCN | –[2] |
| 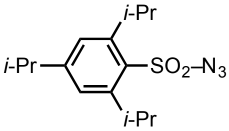 | MeCN | –1.25 |
| 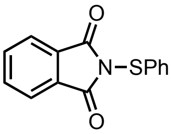 | MeCN | –1.11 |
| 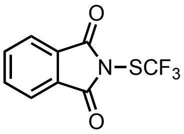 | MeCN | –1.09 |
| 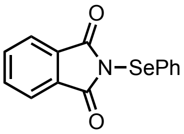 | MeCN | –1.01 |
| 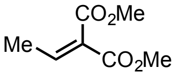 | MeCN | –1.49 |
| 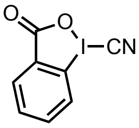 | DMF | –0.70 |

[1] In this case we were not able to determine the E1/2ox at a scan rate of 1.0 V $s^{-1}$. As a result, this potential was obtained at a scan rate of 0.5 V $s^{-1}$.

[2] We have not been able to obtain accurate and reproducible electrochemical data for 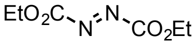.

| Substrate | Solvent | $E_{1/2}^{ox}$ (V) vs SCE |
|---|---|---|
| 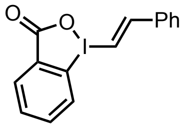 | DMF | –0.98 |
| 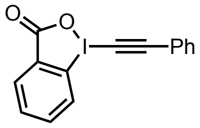 | DMF | –1.11 |

## 4.5 Cyclic Voltammograms

### Oximes

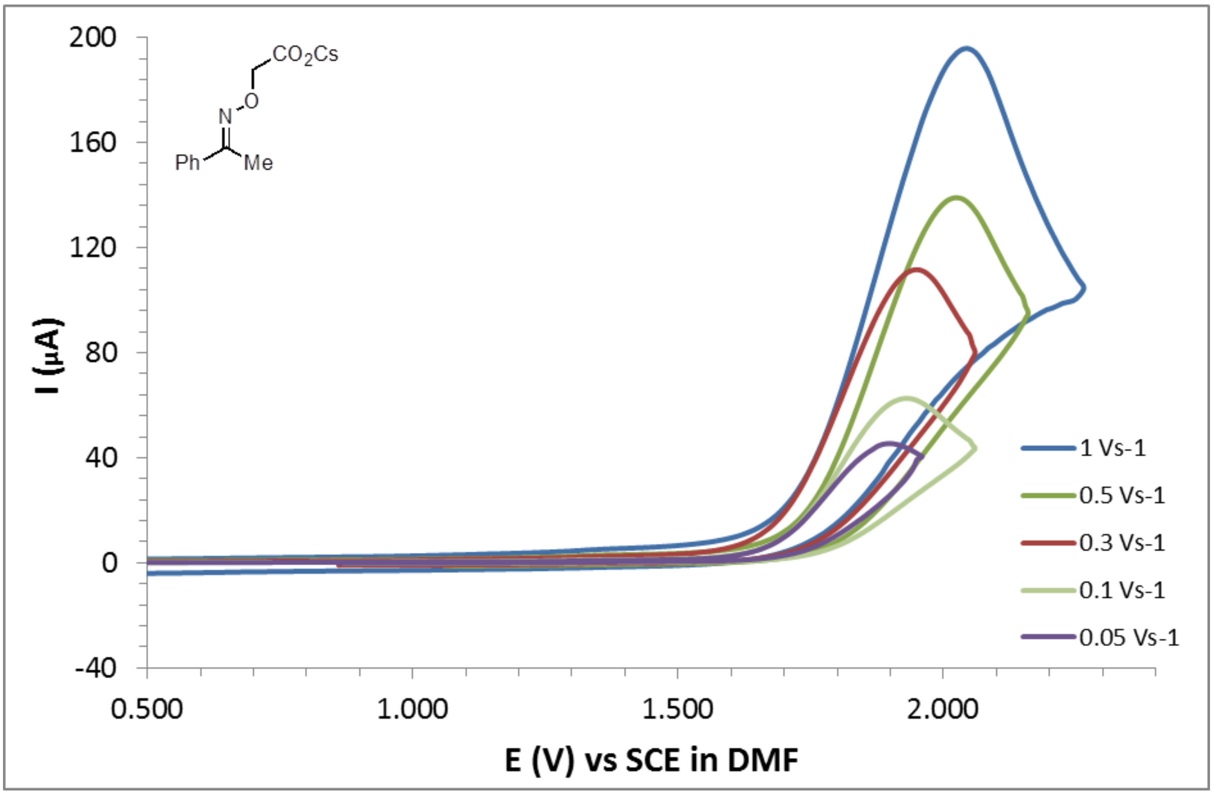


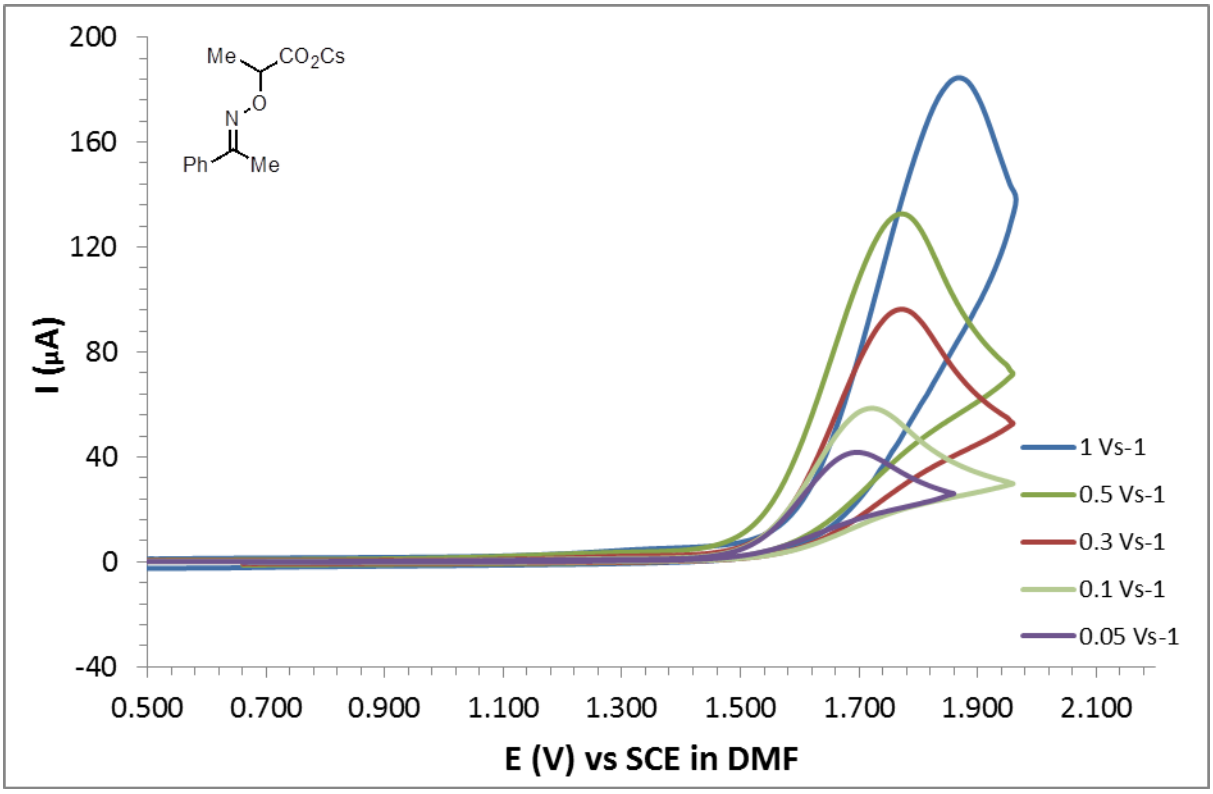

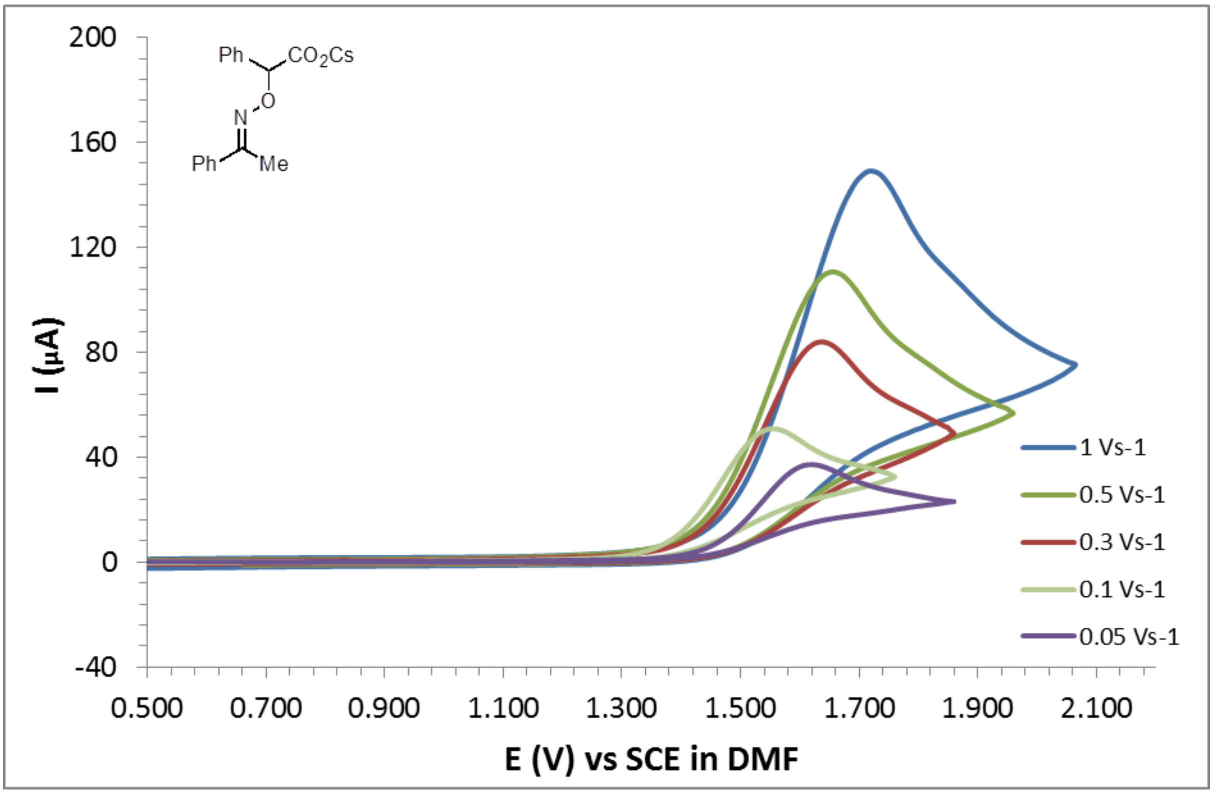

Ph
$CO_2Cs$
O
N
Ph
Me
I (μA)
200
160
120
80
40
0
-40
0.500
0.700
0.900
1.100
1.300
1.500
1.700
1.900
2.100
E (V) vs SCE in DMF
1 Vs-1
0.5 Vs-1
0.3 Vs-1
0.1 Vs-1
0.05 Vs-1


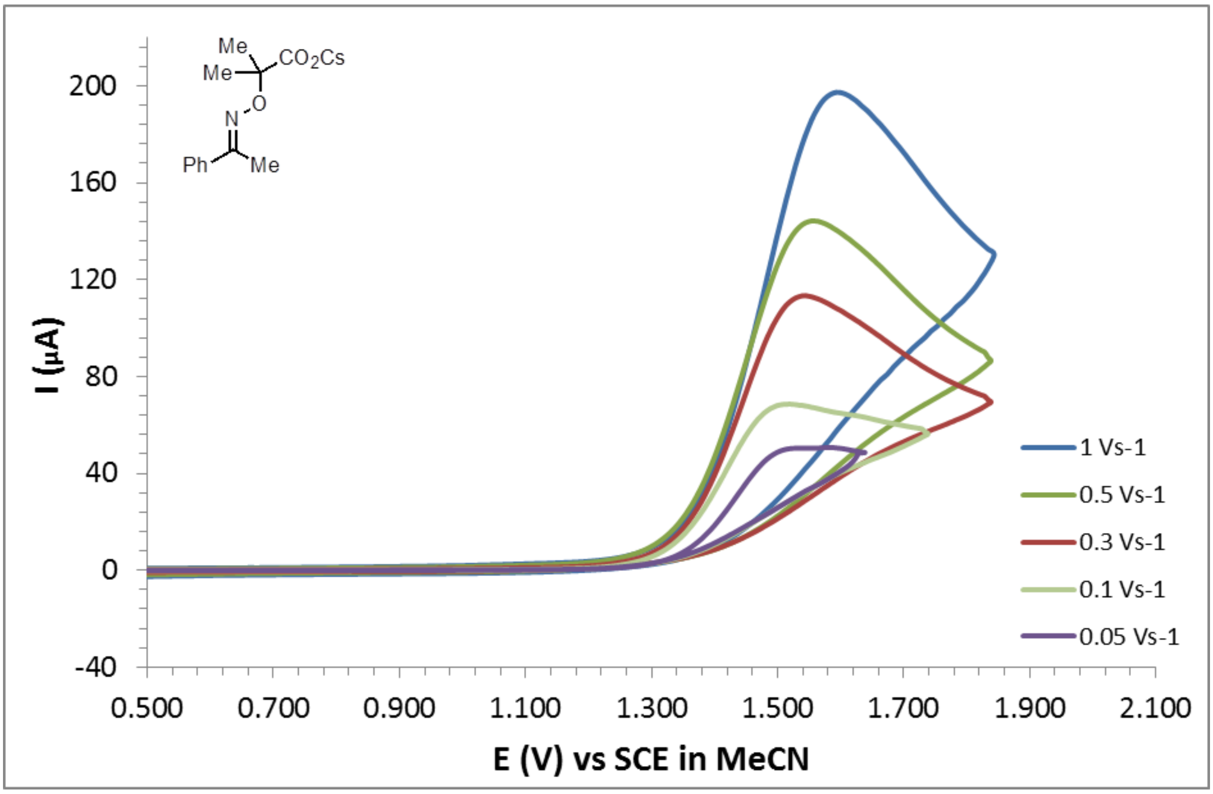

Me
Me
$CO_2Cs$
O
N
Ph
Me
I (μA)
200
160
120
80
40
0
-40
0.500
0.700
0.900
1.100
1.300
1.500
1.700
1.900
2.100
E (V) vs SCE in MeCN
1 Vs-1
0.5 Vs-1
0.3 Vs-1
0.1 Vs-1
0.05 Vs-1

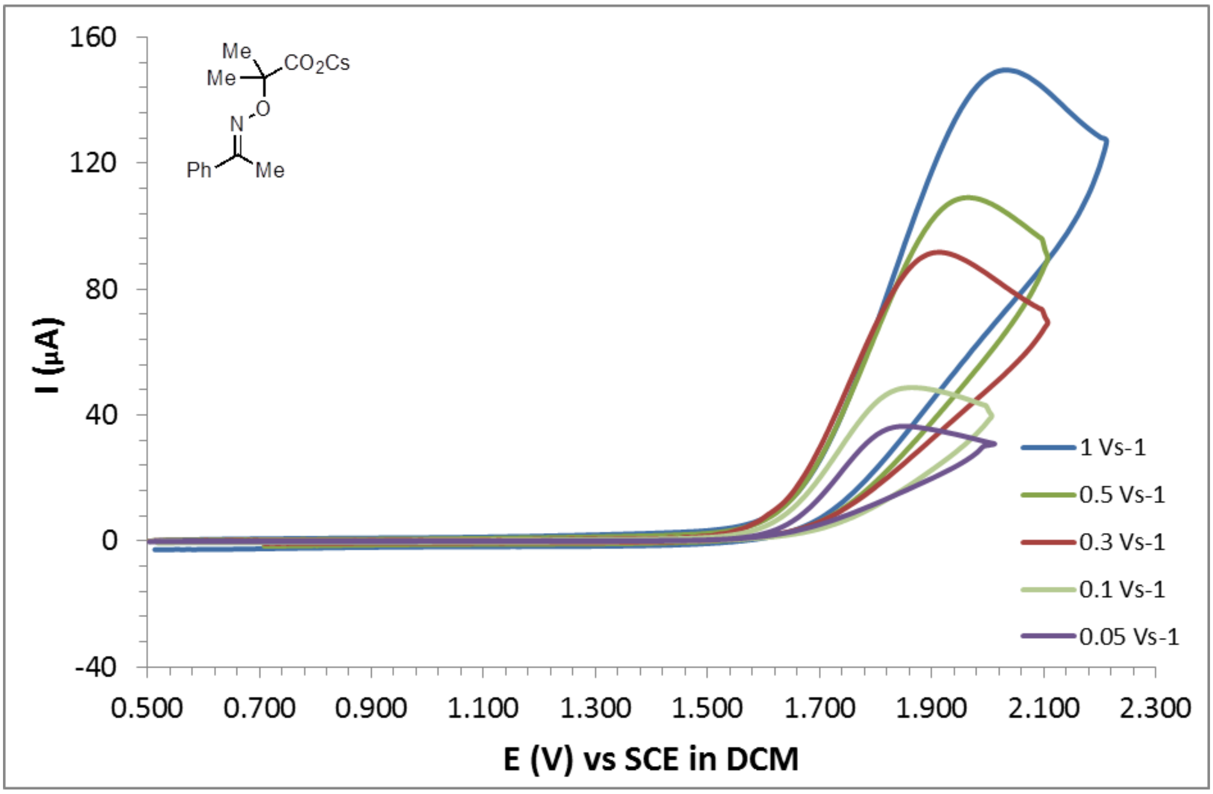
160
120
80
40
0
-40
I (μA)
Me
Me
CO2Cs
O
N
Ph
Me
0.500
0.700
0.900
1.100
1.300
1.500
1.700
1.900
2.100
2.300
E (V) vs SCE in DCM
1 Vs-1
0.5 Vs-1
0.3 Vs-1
0.1 Vs-1
0.05 Vs-1

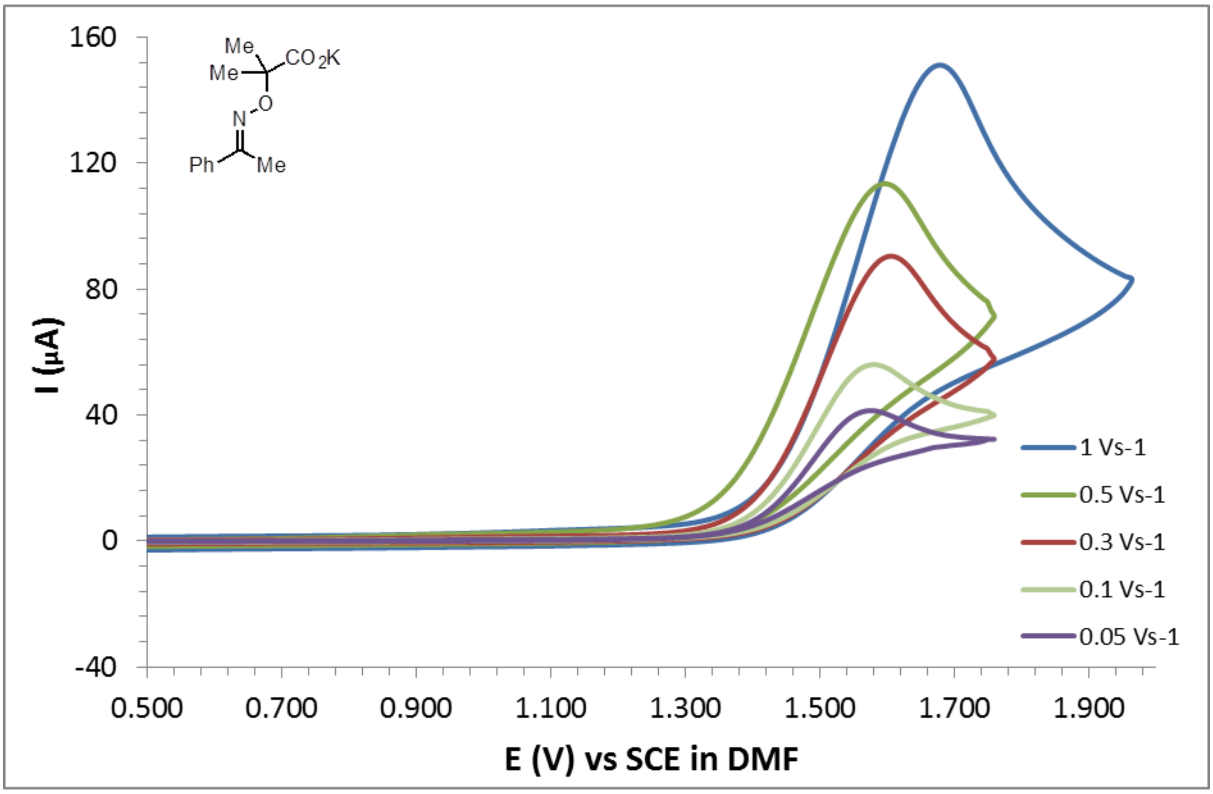
160
120
80
40
0
-40
I (μA)
Me
Me
CO2K
O
N
Ph
Me
0.500
0.700
0.900
1.100
1.300
1.500
1.700
1.900
E (V) vs SCE in DMF
1 Vs-1
0.5 Vs-1
0.3 Vs-1
0.1 Vs-1
0.05 Vs-1

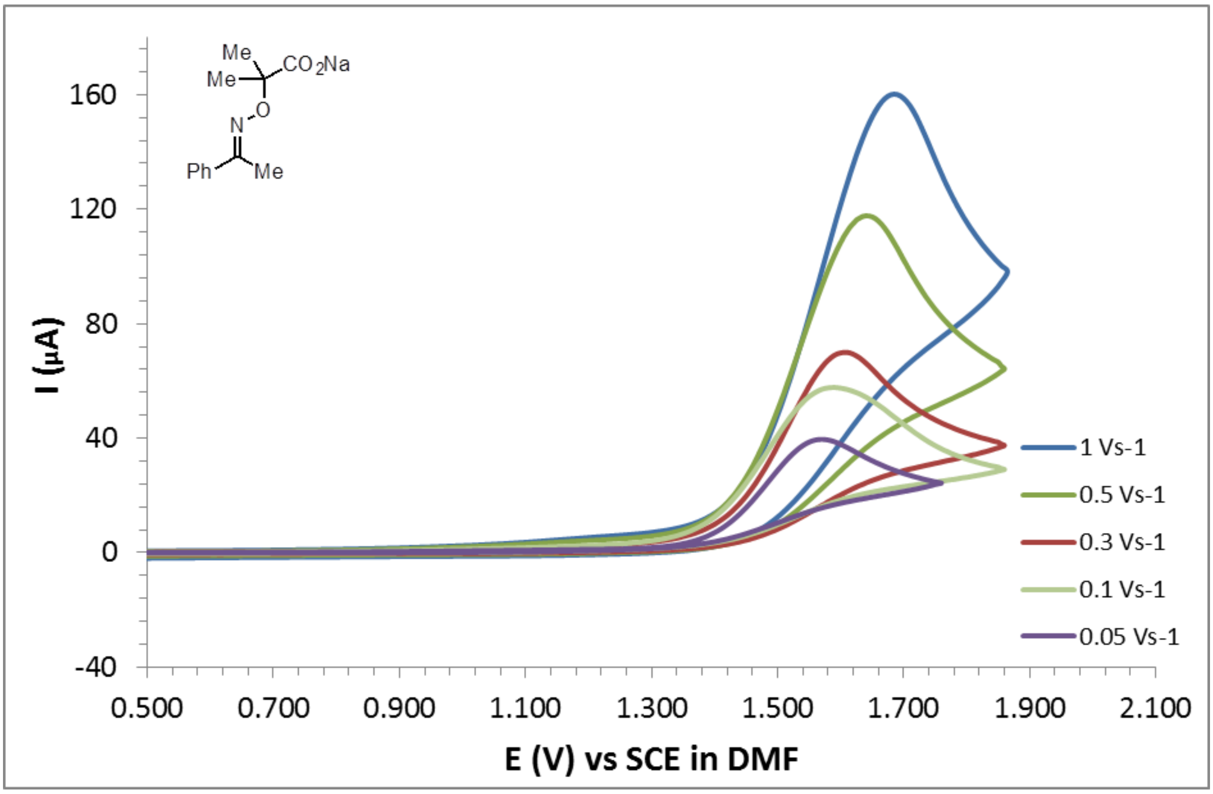

Me
Me
CO2Na
O
N
Ph
Me
160
120
80
40
0
-40
I (μA)
0.500
0.700
0.900
1.100
1.300
1.500
1.700
1.900
2.100
E (V) vs SCE in DMF
1 Vs-1
0.5 Vs-1
0.3 Vs-1
0.1 Vs-1
0.05 Vs-1


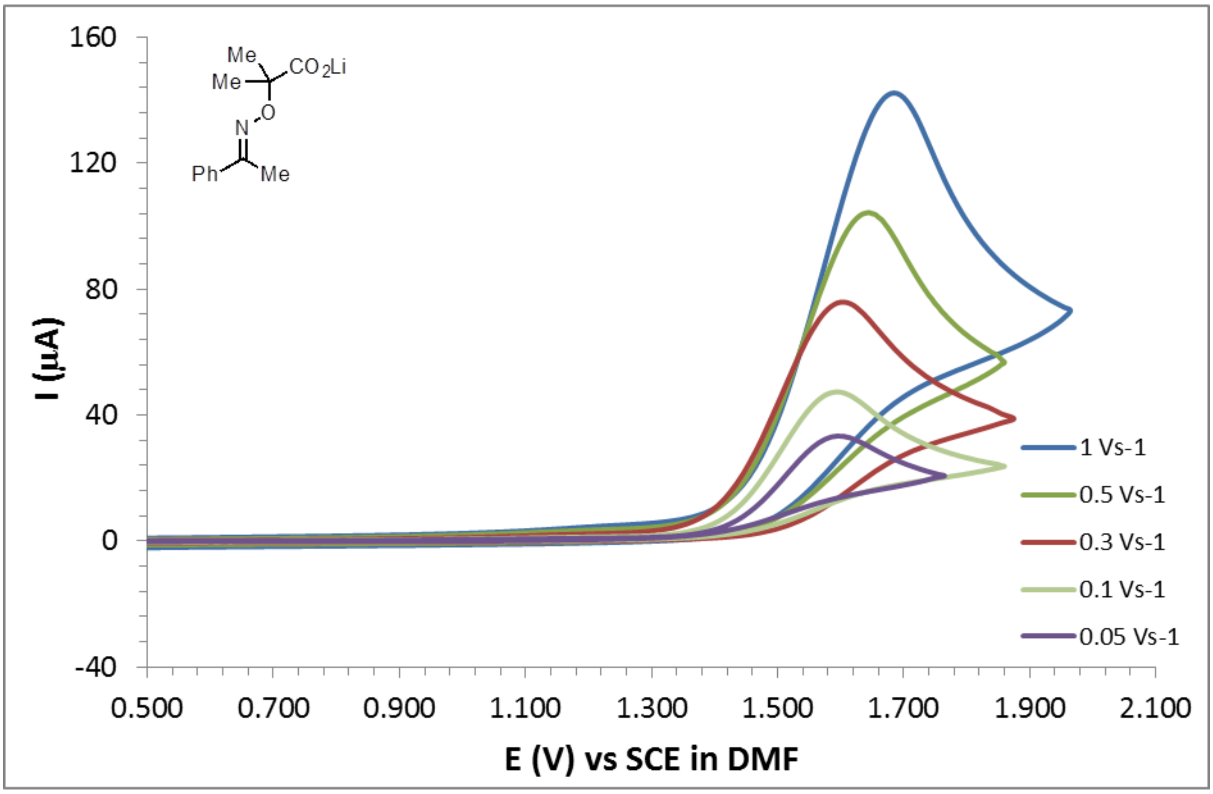

Me
Me
CO2Li
O
N
Ph
Me
160
120
80
40
0
-40
I (μA)
0.500
0.700
0.900
1.100
1.300
1.500
1.700
1.900
2.100
E (V) vs SCE in DMF
1 Vs-1
0.5 Vs-1
0.3 Vs-1
0.1 Vs-1
0.05 Vs-1

## X–Y Reagents

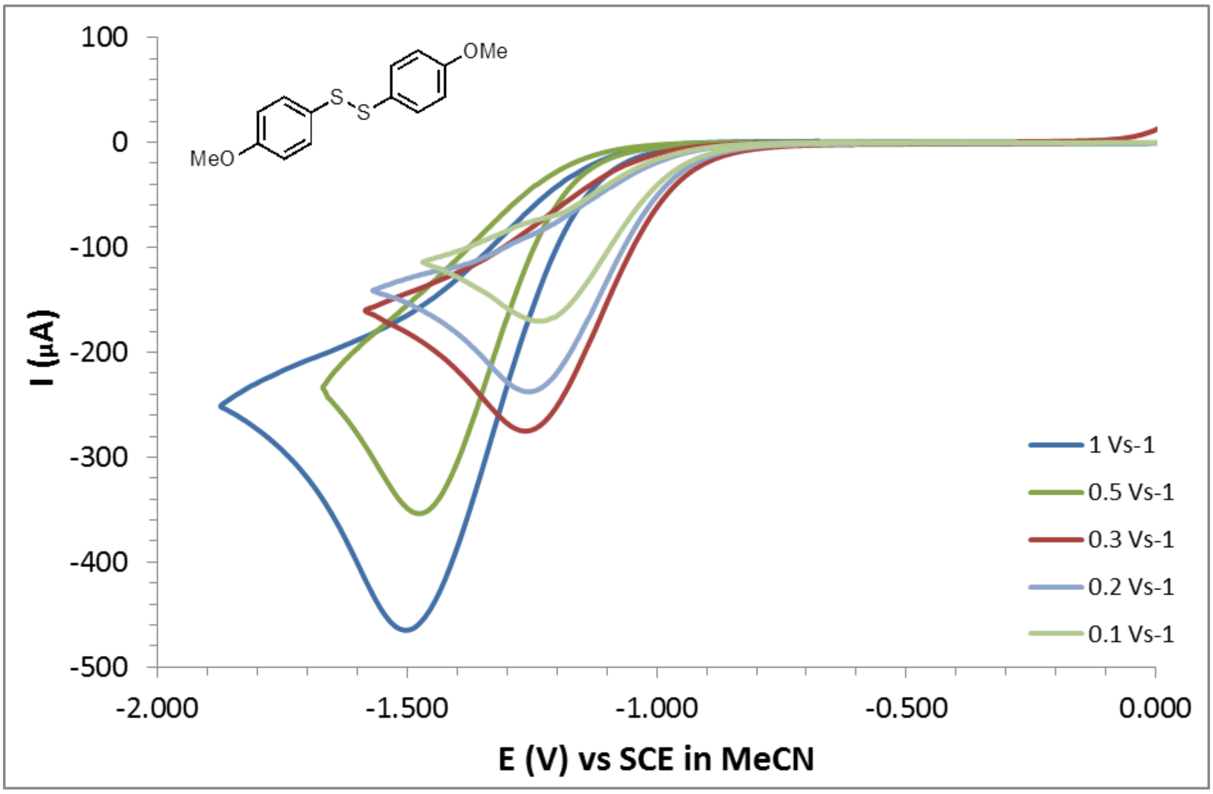

OMe
MeO
S
S
100
0
-100
-200
-300
-400
-500
I (µA)
-2.000
-1.500
-1.000
-0.500
0.000
E (V) vs SCE in MeCN
1 Vs-1
0.5 Vs-1
0.3 Vs-1
0.2 Vs-1
0.1 Vs-1


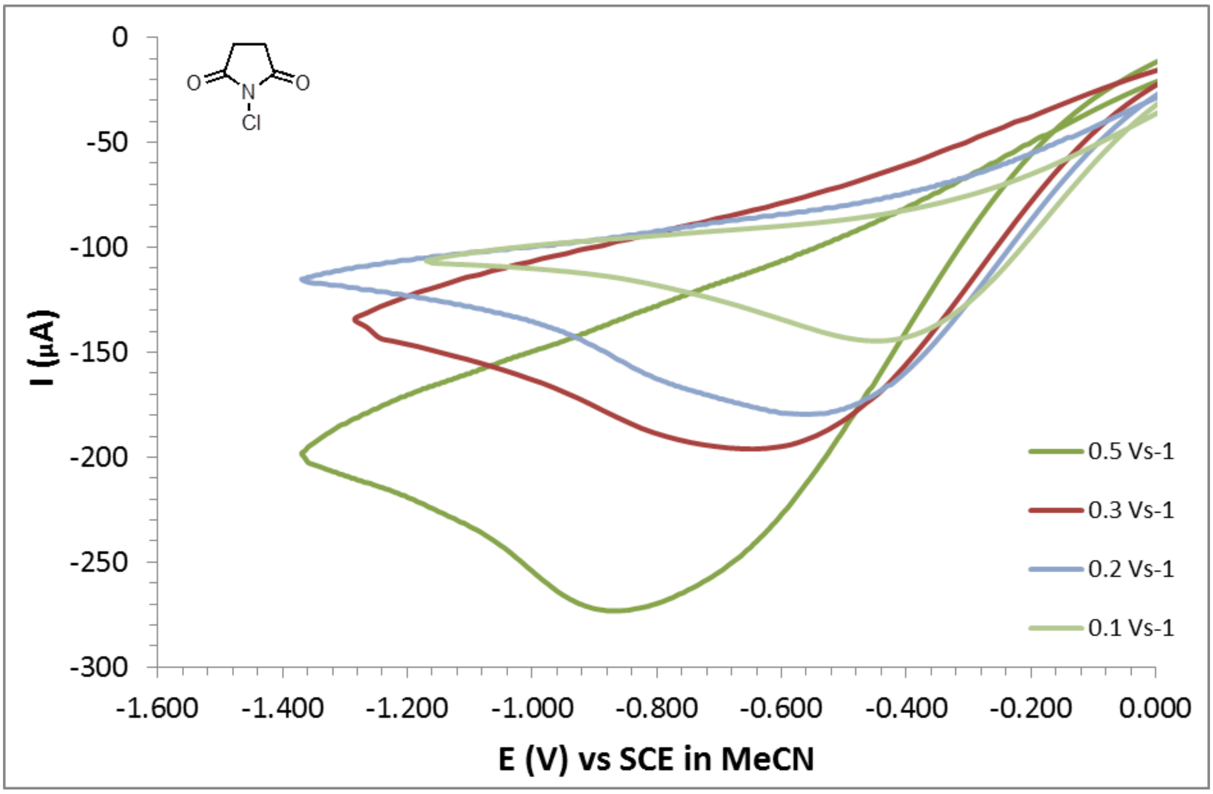

O
O
N
Cl
0
-50
-100
-150
-200
-250
-300
I (µA)
-1.600
-1.400
-1.200
-1.000
-0.800
-0.600
-0.400
-0.200
0.000
E (V) vs SCE in MeCN
0.5 Vs-1
0.3 Vs-1
0.2 Vs-1
0.1 Vs-1

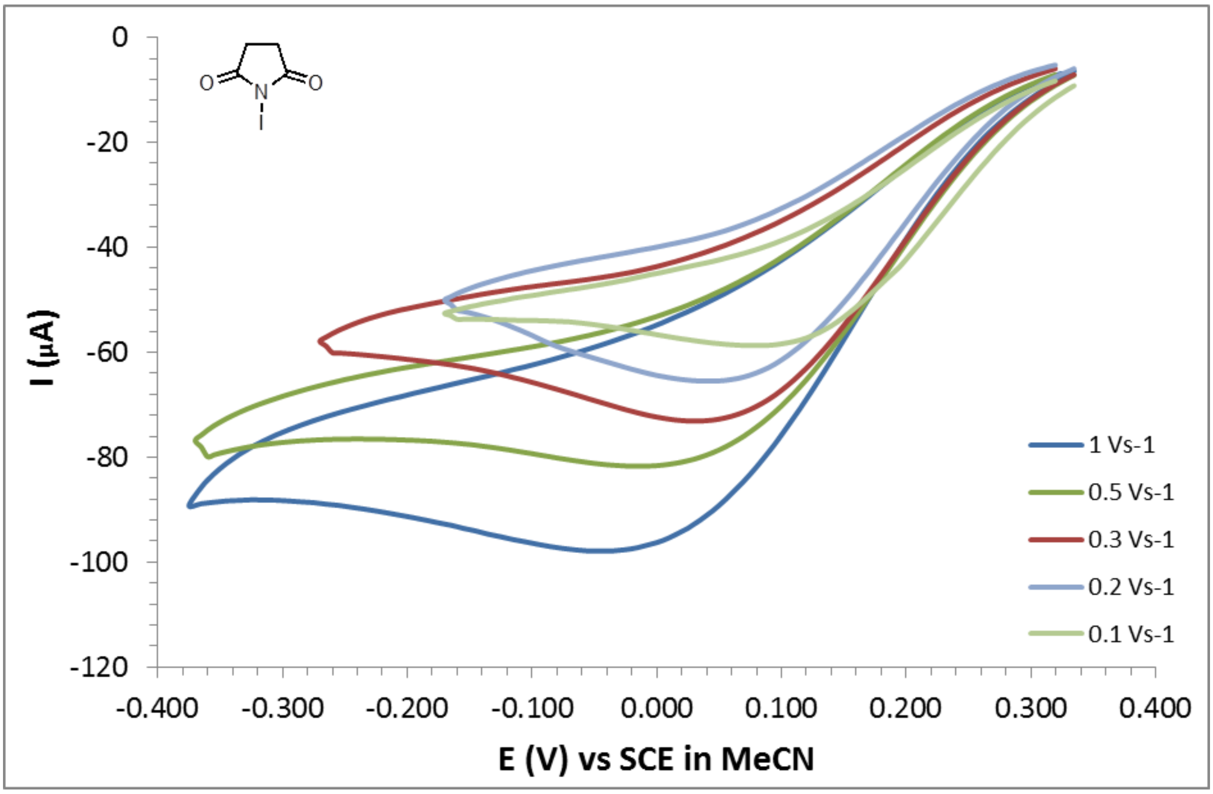
O
N
O
I
0
-20
-40
-60
-80
-100
-120
I (μA)
-0.400
-0.300
-0.200
-0.100
0.000
0.100
0.200
0.300
0.400
E (V) vs SCE in MeCN
1 Vs-1
0.5 Vs-1
0.3 Vs-1
0.2 Vs-1
0.1 Vs-1

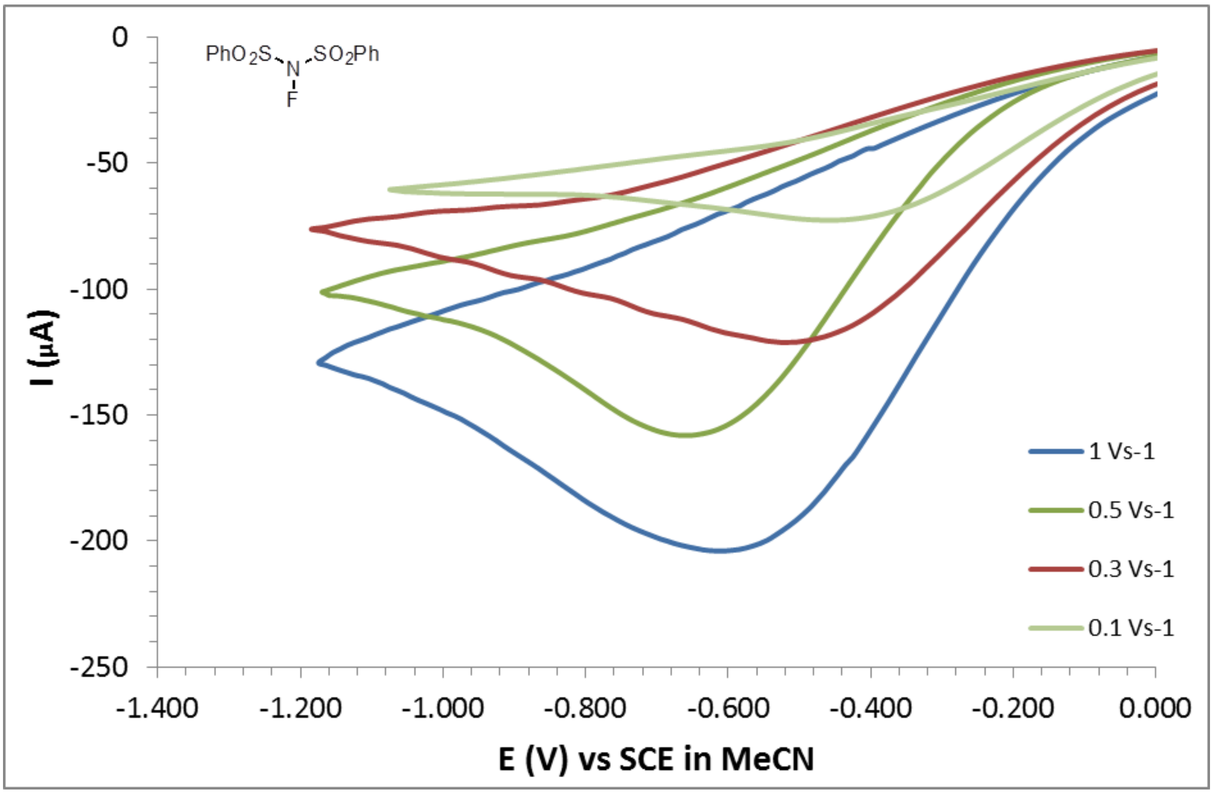
PhO2S
N
SO2Ph
F
0
-50
-100
-150
-200
-250
I (μA)
-1.400
-1.200
-1.000
-0.800
-0.600
-0.400
-0.200
0.000
E (V) vs SCE in MeCN
1 Vs-1
0.5 Vs-1
0.3 Vs-1
0.1 Vs-1

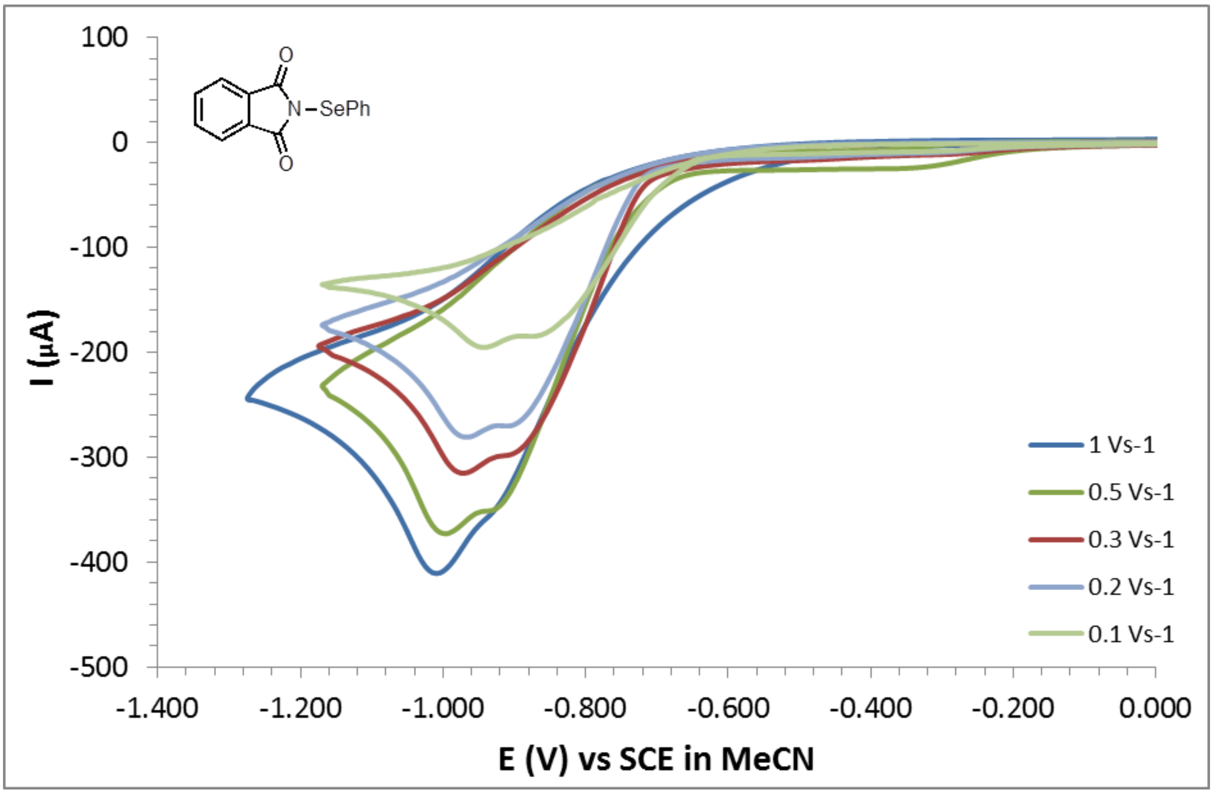

N–SePh
I (μA)
E (V) vs SCE in MeCN
1 Vs-1
0.5 Vs-1
0.3 Vs-1
0.2 Vs-1
0.1 Vs-1


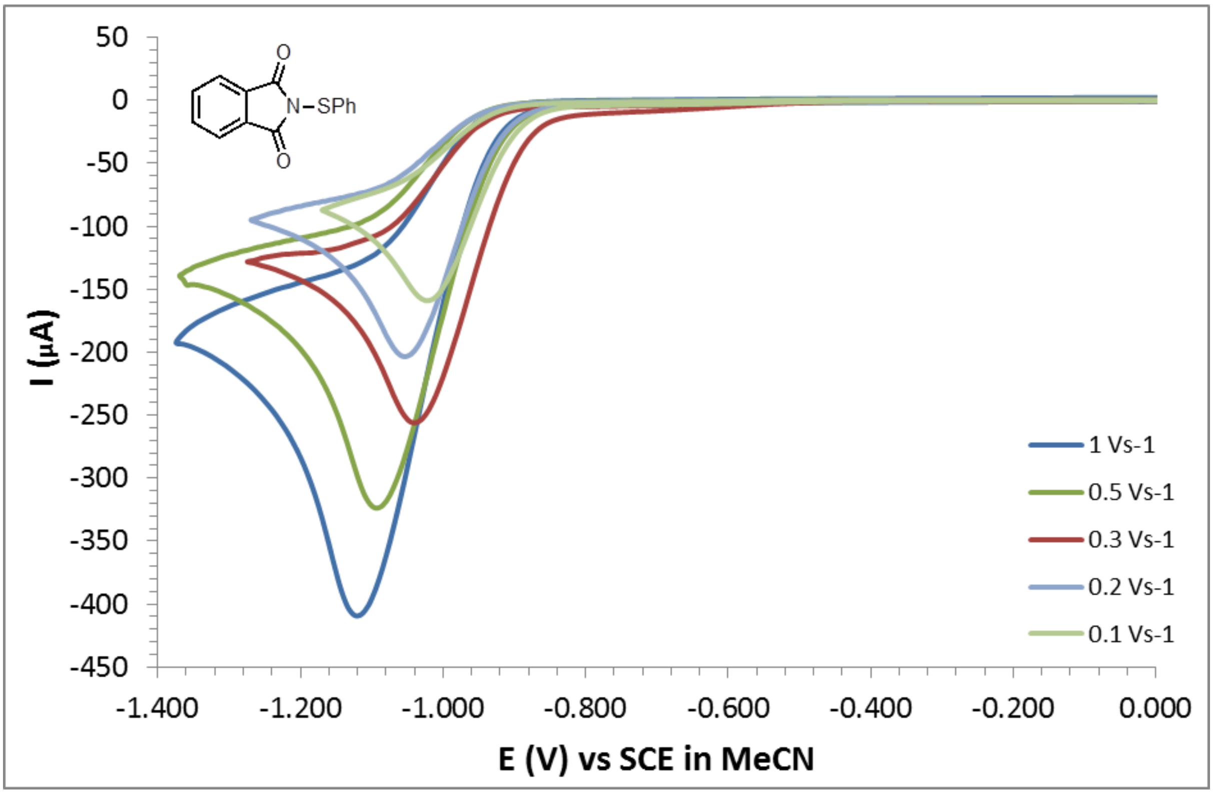

N–SPh
I (μA)
E (V) vs SCE in MeCN
1 Vs-1
0.5 Vs-1
0.3 Vs-1
0.2 Vs-1
0.1 Vs-1

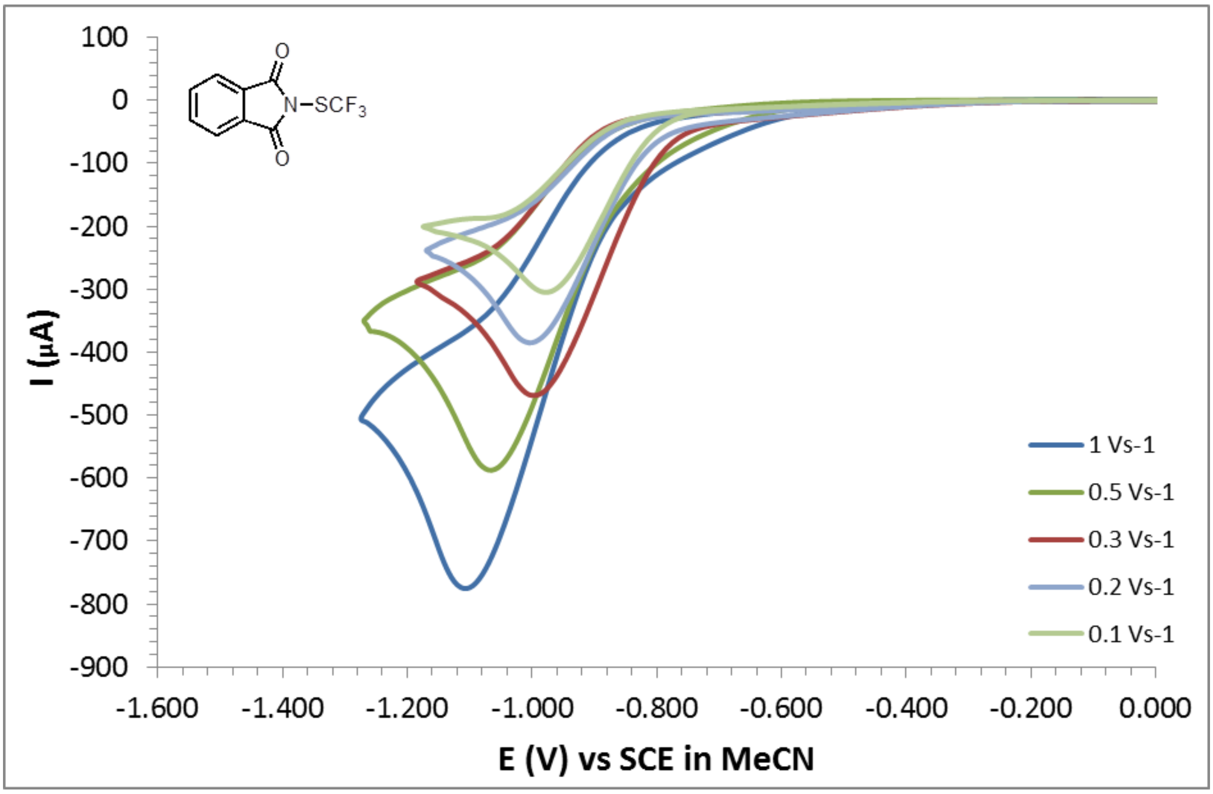
N–SCF3
I (µA)
E (V) vs SCE in MeCN
1 Vs-1
0.5 Vs-1
0.3 Vs-1
0.2 Vs-1
0.1 Vs-1

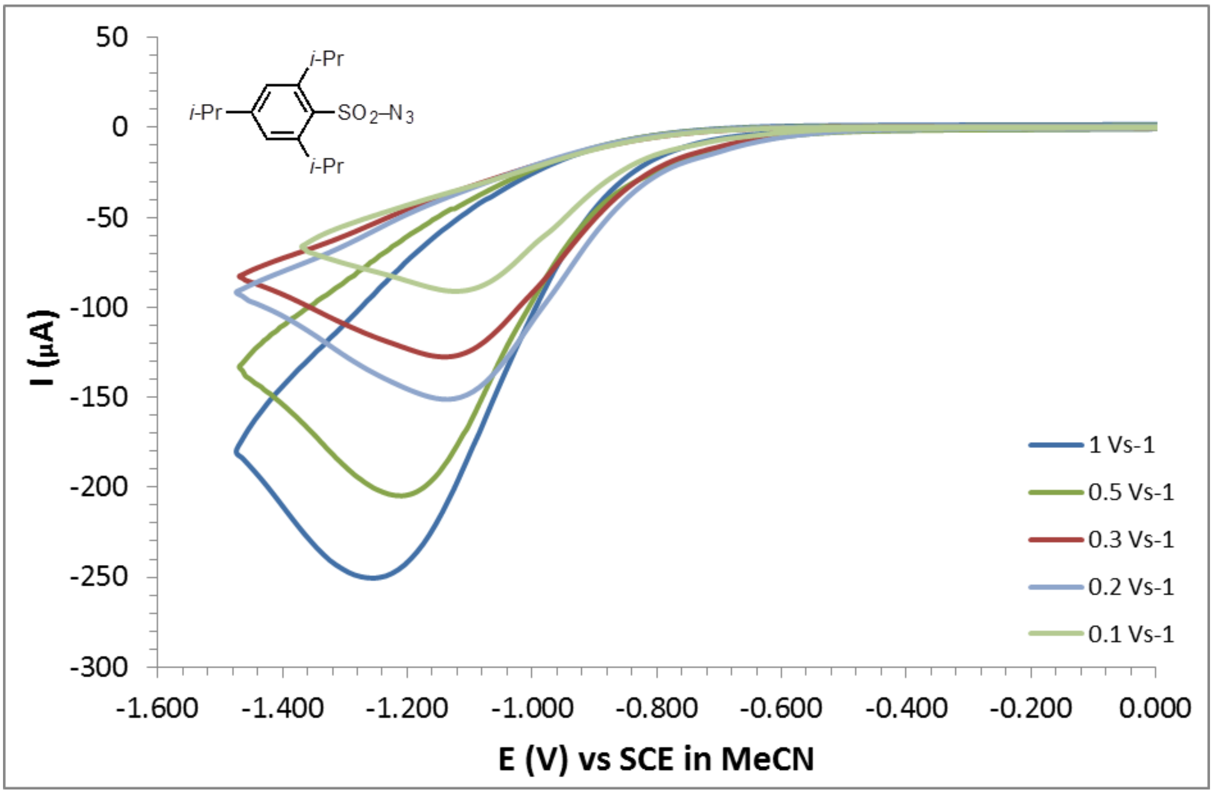
i-Pr
SO2–N3
I (µA)
E (V) vs SCE in MeCN
1 Vs-1
0.5 Vs-1
0.3 Vs-1
0.2 Vs-1
0.1 Vs-1

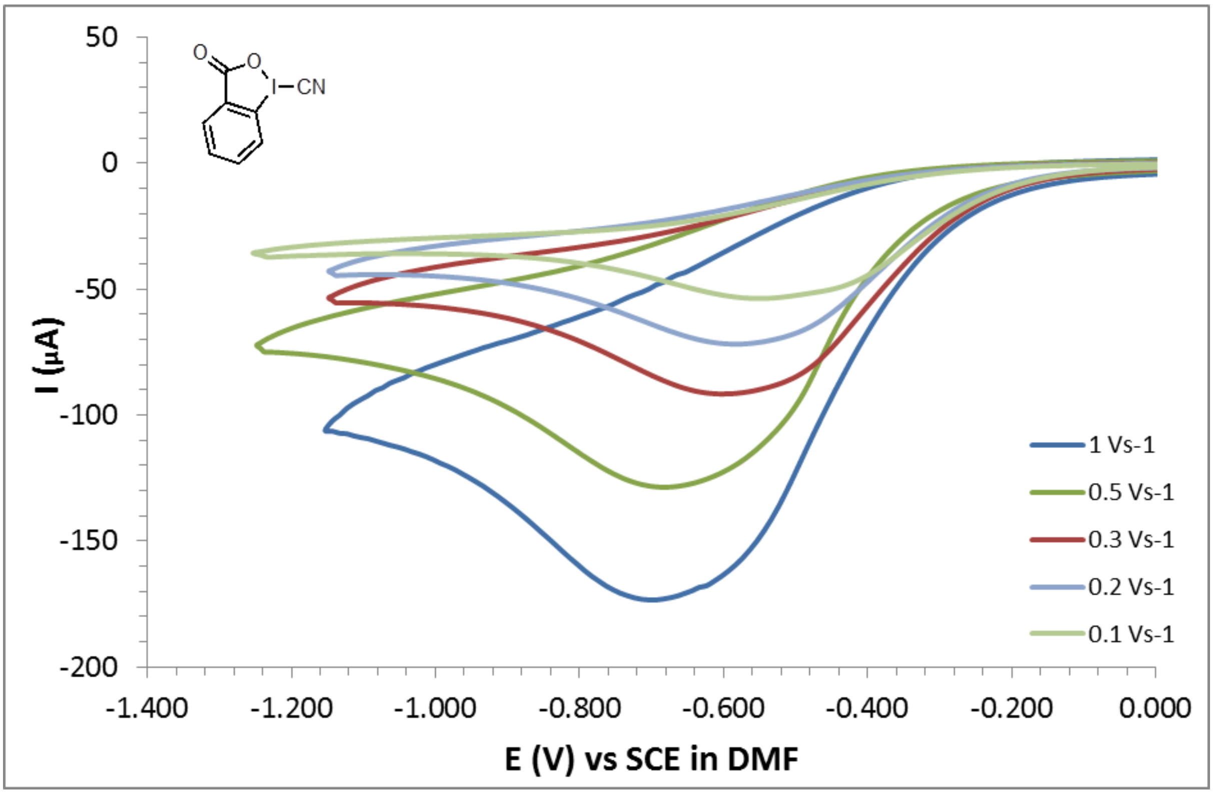
50
0
-50
-100
-150
-200
I (μA)
O
O
I
CN
1 Vs-1
0.5 Vs-1
0.3 Vs-1
0.2 Vs-1
0.1 Vs-1
-1.400
-1.200
-1.000
-0.800
-0.600
-0.400
-0.200
0.000
E (V) vs SCE in DMF

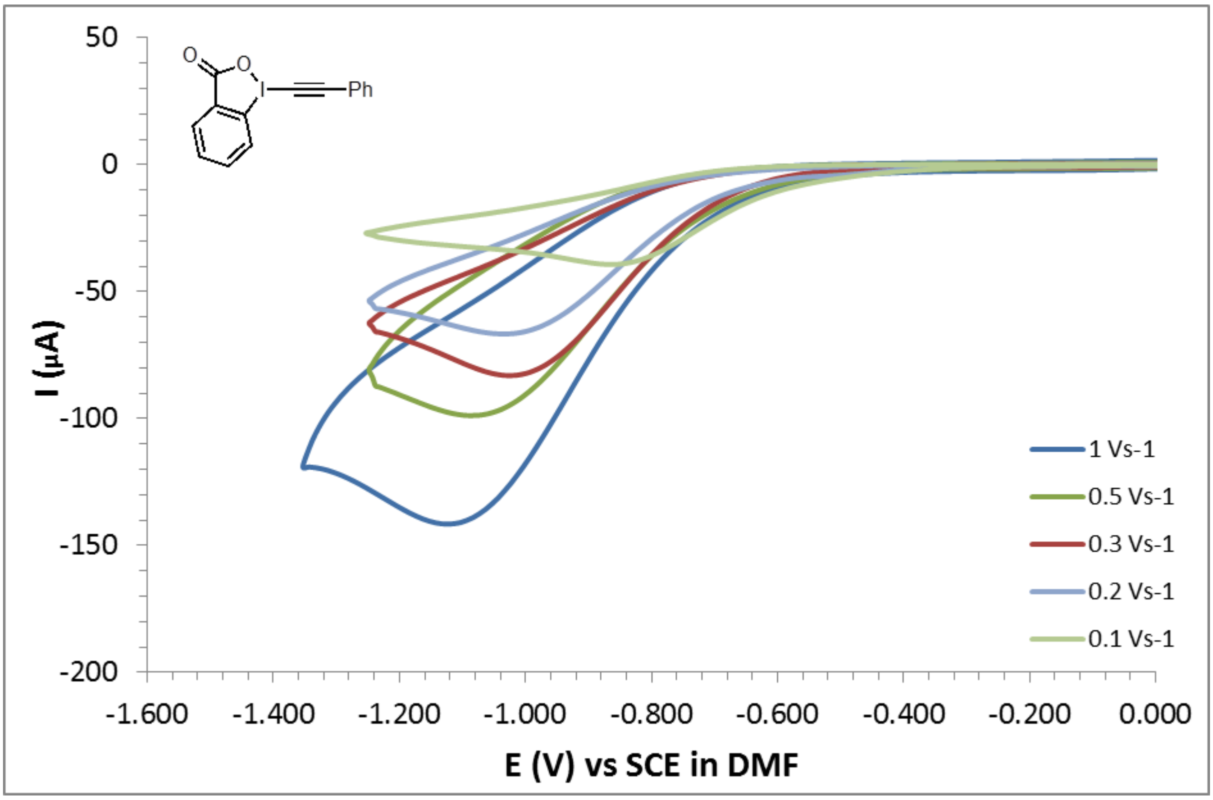
50
0
-50
-100
-150
-200
I (μA)
O
O
I
Ph
1 Vs-1
0.5 Vs-1
0.3 Vs-1
0.2 Vs-1
0.1 Vs-1
-1.600
-1.400
-1.200
-1.000
-0.800
-0.600
-0.400
-0.200
0.000
E (V) vs SCE in DMF

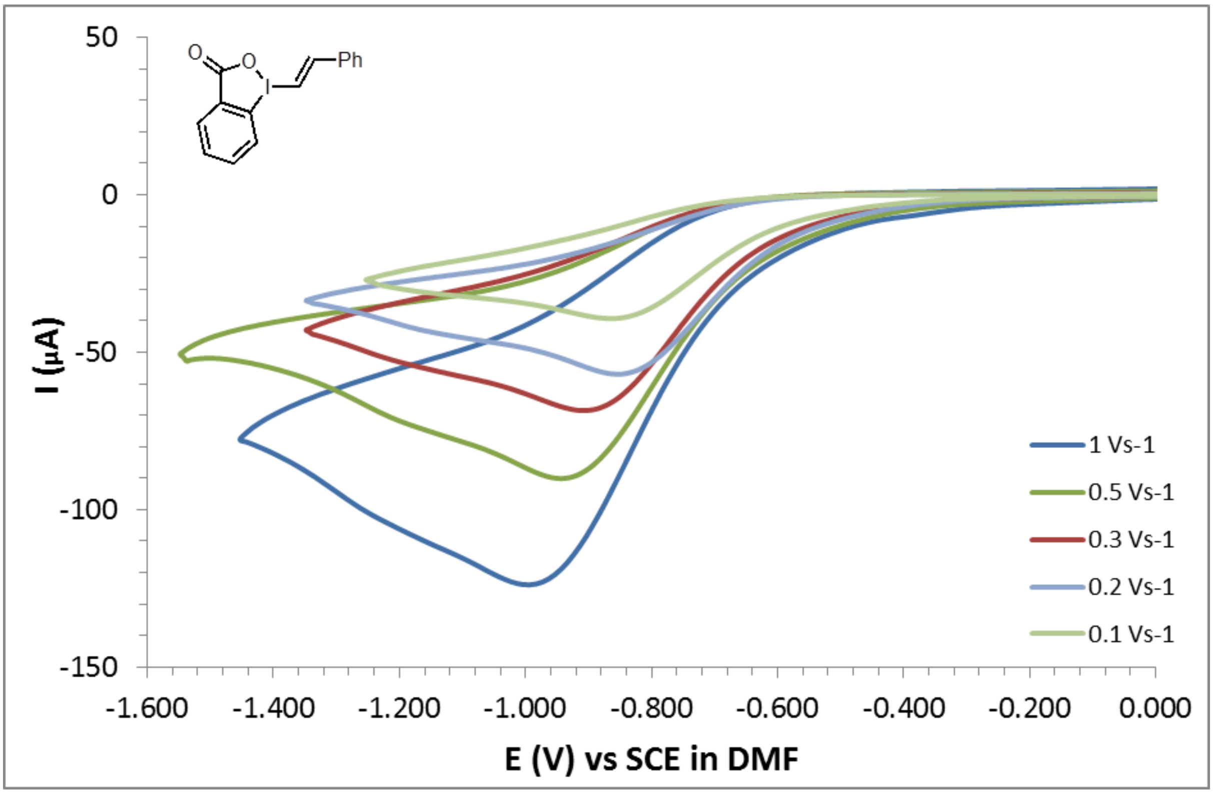
50
0
-50
-100
-150
I (µA)
O
O
I
Ph
1 Vs-1
0.5 Vs-1
0.3 Vs-1
0.2 Vs-1
0.1 Vs-1
-1.600
-1.400
-1.200
-1.000
-0.800
-0.600
-0.400
-0.200
0.000
E (V) vs SCE in DMF

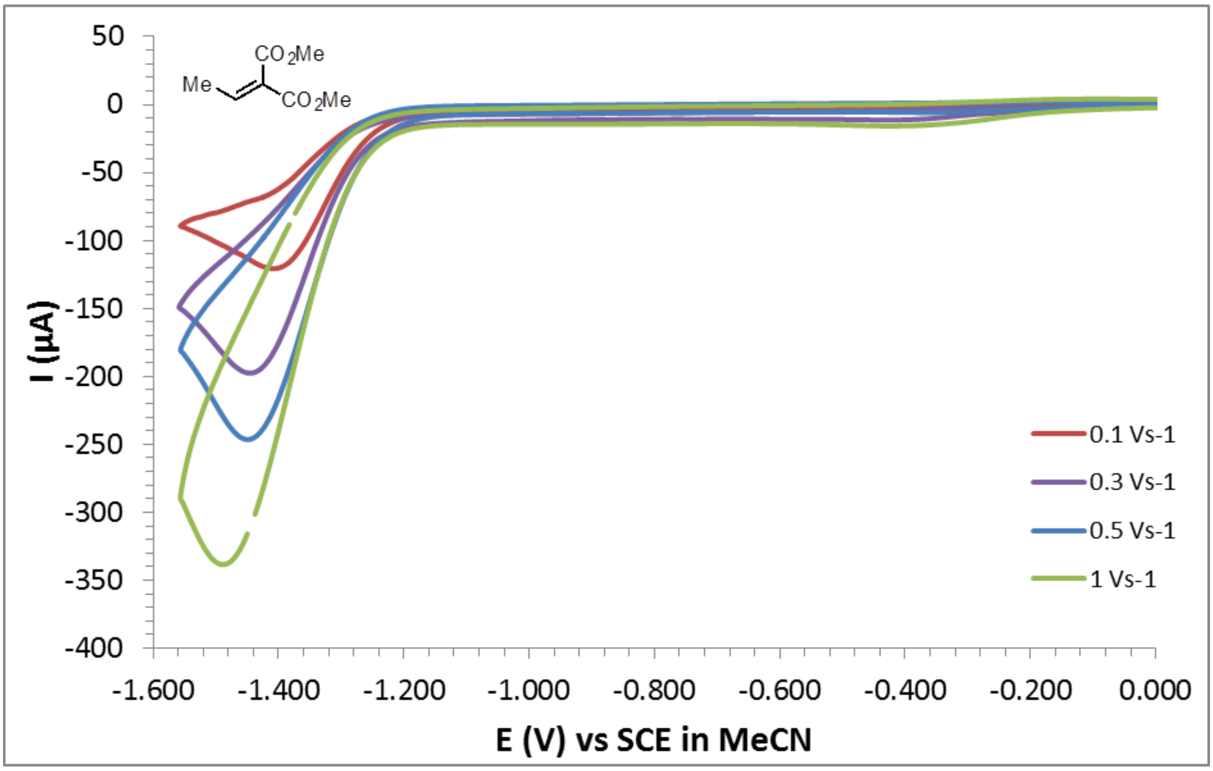
50
0
-50
-100
-150
-200
-250
-300
-350
-400
I (µA)
CO2Me
Me
CO2Me
0.1 Vs-1
0.3 Vs-1
0.5 Vs-1
1 Vs-1
-1.600
-1.400
-1.200
-1.000
-0.800
-0.600
-0.400
-0.200
0.000
E (V) vs SCE in MeCN

## 5 Reaction Optimizations

### 5.1 Hydro-imination

**General Procedure for the Reaction Optimization – GP4**

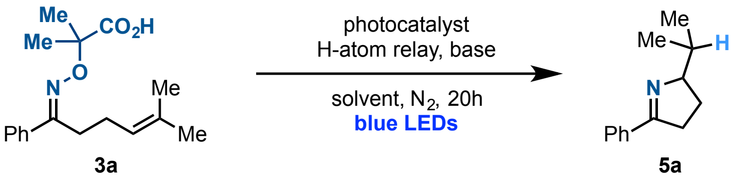


To dry tube was added **3a** (13 mg, 0.05 mmol, 1.0 equiv.), the photocatalyst (5 mol%), RSSR and the base (if solid). A stirrer bar was added and the tube capped with a Supelco aluminium crimp seal with septum (PTFE/butyl). The tube was evacuated and refilled with $N_2$ (x 3) and the solvent (dry and degassed by bubbling through with nitrogen for 20 mins) was added. The base (if liquid) (0.1 mmol, 1.0 equiv.) and RSH/RSSR (if liquid) was added via micro-syringe. The nitrogen inlet was then removed and the cap sealed with para-film. The mixture was stirred at room temperature for 20 h in front of blue LEDs. 1,3,5-Trimethoxybenzene (3.0 mg, 0.018 mmol, 0.28 equiv.) was added and the solvent removed under reduced pressure. $CDCl_3$ (0.4 mL) was added and the mixture was analysed by $^1$H NMR spectroscopy to determine the NMR yield.

The optimum reaction conditions identified by this optimisation study were:

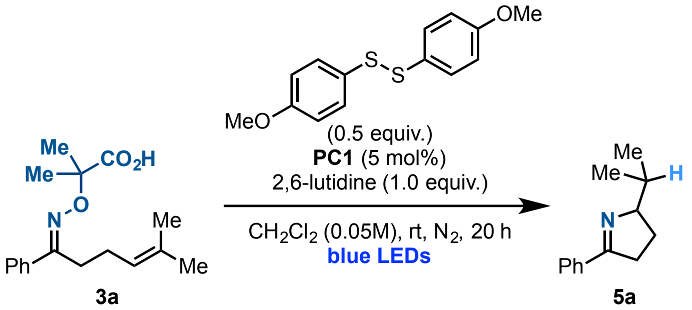


The following Table reports all the experiments performed.

| Entry | PC (mol%) | H-Atom Relay (equiv.) | Base (equiv.) | Solvent (M) | Yield (%) |
|---|---|---|---|---|---|
| **1** | **PC1** (5) | 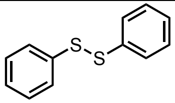 (0.5) | 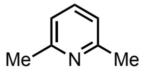 (1.0) | DCE (0.025) | 37 |
| **2** | **PC1** (5) | 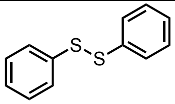 (0.5) | 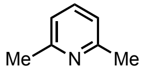 (1.0) | DCE (0.05) | 40 |
| **3** | **PC1** (5) | 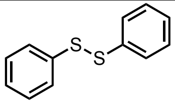 (0.5) | 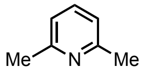 (1.0) | $CH_2Cl_2$ (0.05) | 60 |
| **4** | **PC1** (5) | 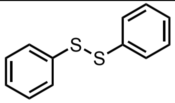 (0.5) | 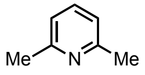 (1.0) | $CHCl_3$ (0.05) | 20 |
| **5** | **PC1** (5) | 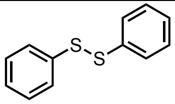 (0.5) | 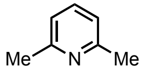 (1.0) | acetone (0.05) | 15 |
| **6** | **PC1** (5) | 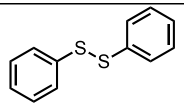 (0.5) | 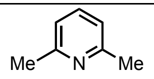 (1.0) | DMF (0.05) | – |

| Entry | PC (mol%) | H-Atom Relay (equiv.) | Base (equiv.) | Solvent (M) | Yield (%) |
|---|---|---|---|---|---|
| **7** | **PC1** (5) | 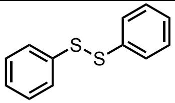 (0.5) | 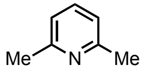 (1.0) | $CH_3CN$ (0.05) | – |
| **8** | **PC1** (5) | 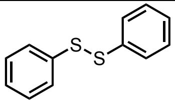 (0.5) | 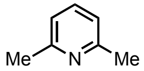 (1.0) | THF (0.05) | – |
| **9** | **PC1** (5) | 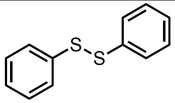 (0.5) | 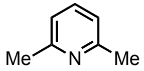 (1.0) | $CH_2Cl_2$ (0.1) | 50 |
| **10** | **PC1** (5) | 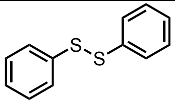 (0.5) | 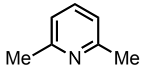 (1.0) | $CH_2Cl_2$ (0.025) | 43 |
| **11** | **PC1** (5) | 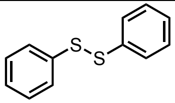 (0.5) | 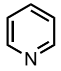 (1.0) | $CH_2Cl_2$ (0.05) | 15 |
| **12** | **PC1** (5) | 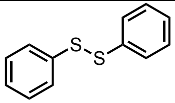 (0.5) | 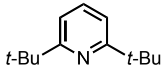 (1.0) | $CH_2Cl_2$ (0.05) | 5 |

| Entry | PC (mol%) | H-Atom Relay (equiv.) | Base (equiv.) | Solvent (M) | Yield (%) |
|---|---|---|---|---|---|
| **13** | **PC1** (5) | 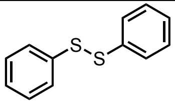 (0.5) | $Cs_2CO_3$ (1.0) | $CH_2Cl_2$ (0.05) | – |
| **14** | **PC1** (5) | 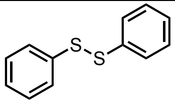 (0.5) | $Na_2HPO_4$ (1.0) | $CH_2Cl_2$ (0.05) | 63 |
| **15** | **PC1** (5) | 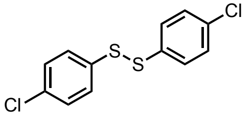  (0.5) | 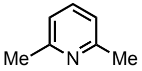  (1.0) | $CH_2Cl_2$ (0.05) | 55 |
| **16** | **PC1** (5) | 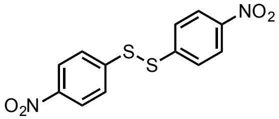  (0.5) | 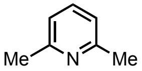  (1.0) | $CH_2Cl_2$ (0.05) | 38 |
| **17** | **PC1** (5) | 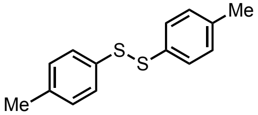  (0.5) | 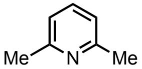  (1.0) | $CH_2Cl_2$ (0.05) | 60 |
| **18** | **PC1** (5) | 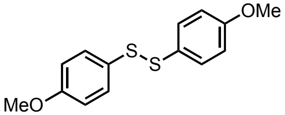  (0.5) | 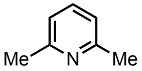  (1.0) | $CH_2Cl_2$ (0.05) | 65 |

| Entry | PC (mol%) | H-Atom Relay (equiv.) | Base (equiv.) | Solvent (M) | Yield (%) |
|---|---|---|---|---|---|
| **21** | **PC1** (5) | 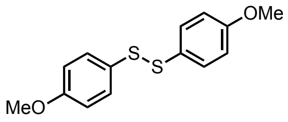 (0.1) | 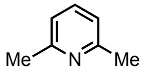 (0.2) | $CH_2Cl_2$ (0.05) | 58 |
| **23** | **PC1** (5) | 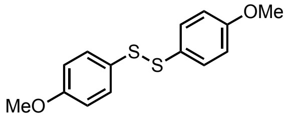 (0.1) | $Na_2HPO_4$ (0.2) | $CH_2Cl_2$ (0.05) | 48 |
| **24** | **PC2** (5) | 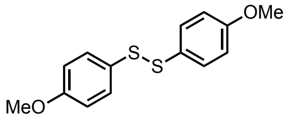 (0.1) | 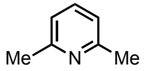 (0.2) | $CH_2Cl_2$ (0.05) | 20 |
| **25**[a] | **PC3** (5) | 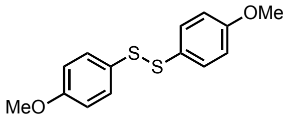 (0.1) | 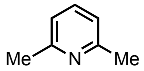 (0.2) | $CH_2Cl_2$ (0.05) | 40 |
| **26** | **PC4** (5) | 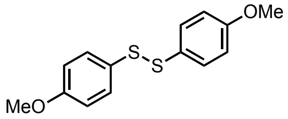 (0.1) | 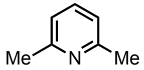 (0.2) | $CH_2Cl_2$ (0.05) | 10 |
| **27** | 5 | 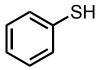 (0.2) | 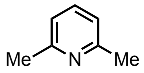 (0.2) | $CH_2Cl_2$ (0.05) | 36 |

| Entry | PC (mol%) | H-Atom Relay (equiv.) | Base (equiv.) | Solvent (M) | Yield (%) |
|---|---|---|---|---|---|
| *Control experiments* | | | | | |
| **29** | **PC1** (5) | 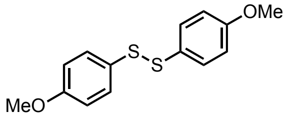 (0.5) | – | $CH_2Cl_2$ (0.05) | 20 |
| **30** | **PC1** (5) | – | 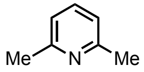 (1.0) | $CH_2Cl_2$ (0.05) | 18 |
| **31** | – | 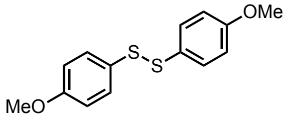 (0.5) | 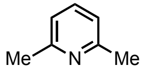 (1.0) | $CH_2Cl_2$ (0.05) | – |
| **32**[b] | **PC1** (5) | 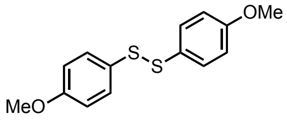 (0.5) | 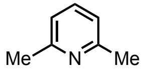 (1.0) | $CH_2Cl_2$ (0.05) | – |
| **33**[c] | **PC1** (5) | 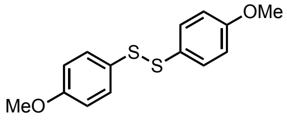 (0.5) | 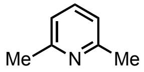 (1.0) | $CH_2Cl_2$ (0.05) | – |

[a] The reaction was run under black light (375 nm) irradiation.

[b] The reaction was run in the dark.

[c] The reaction was run under air.

The optimised reaction conditions were then applied to substrate **SI17**. In [illegible] product was obtained in a much lower yield, which is in line with a more difficu[illegible] oxidation.

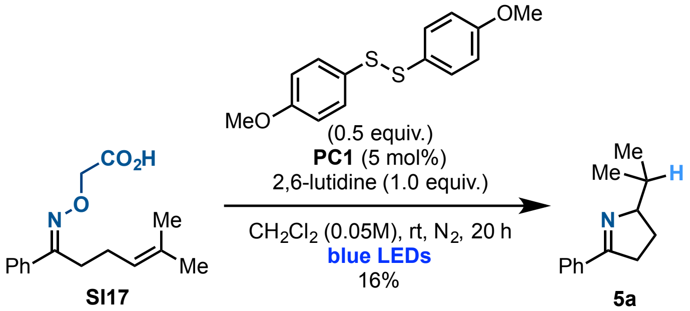


**SI17** **5a**

## 5.2 Imino-chlorination

### General Procedure for the Reaction Optimization – GP5

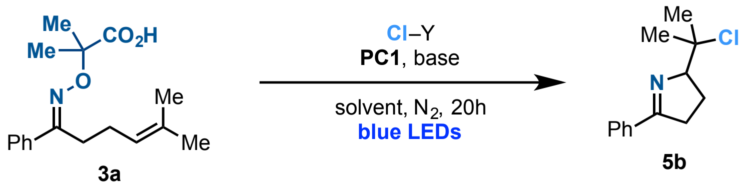


To dry tube was added **3a** (0.05 mmol, 1.0 equiv.), **PC1** (5 mol%), the base and the chlorinating agent (if solid). A stirrer bar was added and the tube capped with a Supelco aluminium crimp seal with septum (PTFE/butyl). The tube was evacuated and refilled with $N_2$ (x 3). The solvent (dry and degassed by bubbling through with nitrogen for 20 mins) was added and X-Y (if liquid). The nitrogen inlet was then removed and the cap sealed with para-film. The mixture was stirred at room temperature for 20 h in front of blue LEDs. 1,3,5-Trimethoxybenzene (3.0 mg, 0.018 mmol, 0.28 equiv.) was added and the solvent removed under reduced pressure. $CDCl_3$ (0.4 mL) was added and the mixture was analysed by $^1H$ NMR spectroscopy to determine the NMR yield.

The optimum reaction conditions identified by this optimisation study were:

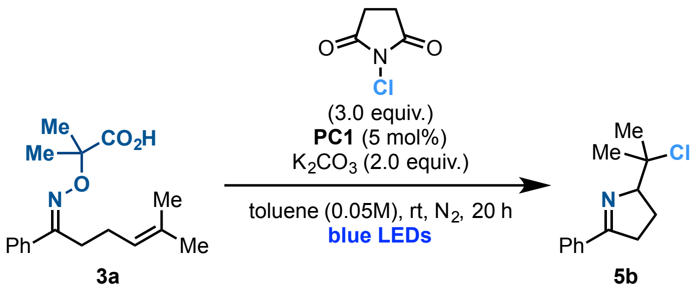


The following Table reports all the experiments performed.

| Entry | **Cl–Y (equiv.)** | **Base (equiv.)** | **Solvent (M)** | **Yield (%)** |
|---|---|---|---|---|
| **1** | $CCl_4$ (2.0) | $Cs_2CO_3$ (1.0) | $CH_2Cl_2$ (0.1) | trace |
| **2** | 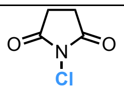 (2.0) | $Cs_2CO_3$ (1.0) | $CH_2Cl_2$ (0.1) | 23 |
| **3** | $Cl_3CCN$ (2.0) | $Cs_2CO_3$ (1.0) | $CH_2Cl_2$ (0.1) | 28 |
| **4** | 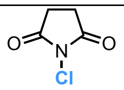 (2.0) | $Cs_2CO_3$ (1.0) | $CH_2Cl_2$ (0.05) | 28 |
| **5** | 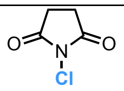 (2.0) | $Cs_2CO_3$ (1.0) | $CH_3CN$ (0.05) | 36 |
| **6** | 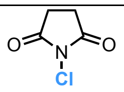 (2.0) | $Cs_2CO_3$ (1.0) | acetone (0.05) | 24 |
| **7** | 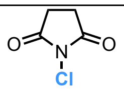 (2.0) | $Cs_2CO_3$ (1.0) | MeCN (0.1) | 27 |
| **8** | 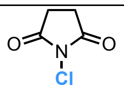 (2.0) | $Cs_2CO_3$ (1.0) | THF (0.05) | – |
| **9** | 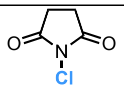 (2.0) | $Cs_2CO_3$ (1.0) | DMF (0.05) | – |
| **10** | 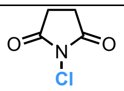 (2.0) | $Cs_2CO_3$ (1.0) | toluene (0.05) | 66 |
| **11** | 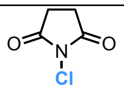 (2.0) | $Cs_2CO_3$ (1.0) | toluene (0.1) | 40 |
| **12** | 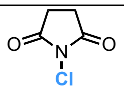 (2.0) | $K_2CO_3$ (1.0) | toluene (0.05) | 68 |

| **Entry** | **Cl–Y (equiv.)** | **Base (equiv.)** | **Solvent (M)** | **Yield (%)** |
|---|---|---|---|---|
| **13** | 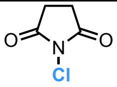 (2.0) | $Na_2HPO_4$ (1.0) | toluene (0.05) | – |
| **14** | 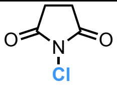 (3.0) | $K_2CO_3$ (1.0) | toluene (0.05) | 81 |
| **16**[a] | 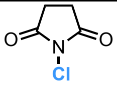 (2.0) | $K_2CO_3$ (1.0) | toluene (0.05) | – |
| **17**[b] | 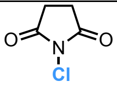 (2.0) | $K_2CO_3$ (1.0) | toluene (0.05) | – |

[a] The reaction was run in the dark.

[b] The reaction was run without **PC1**.

Note: Although reaction optimisation was performed using 1 equiv. of base, it was found that using 2 equiv. gave more reproducible results and therefore was used in when exploring the scope of this transformation.

## 5.3 Imino-bromination

### General Procedure for the Reaction Optimization – GP6

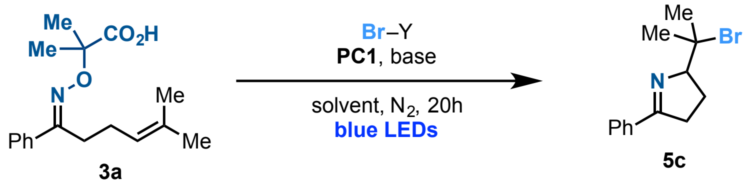


To dry tube was added **3a** (0.05 mmol, 1.0 equiv.), **PC1** (5 mol%), the base and the brominating agent (if solid). A stirrer bar was added and the tube capped with a Supelco aluminium crimp seal with septum (PTFE/butyl). The tube was evacuated and refilled with $N_2$ (x 3). The solvent (dry and degassed by bubbling through with nitrogen for 20 mins) was added and the brominating agent (if liquid). The nitrogen inlet was then removed and the cap sealed with para-film. The mixture was stirred at room temperature for 20 h in front of blue LEDs. 1,3,5-Trimethoxybenzene (3.0 mg, 0.018 mmol, 0.28 equiv.) was added and the solvent removed under reduced pressure. $CDCl_3$ (0.4 mL) was added and the mixture was analysed by $^1$H NMR spectroscopy to determine the NMR yield.

The optimum reaction conditions identified by this optimisation study were:

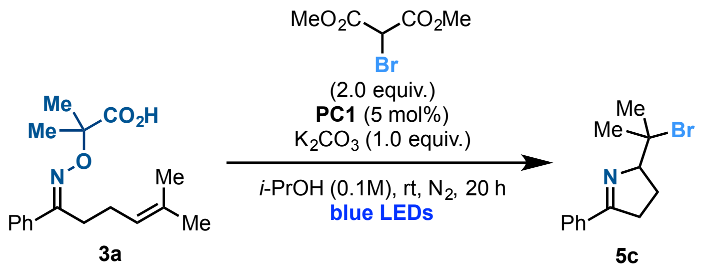


The following Table reports all the experiments performed.

| Entry | Br–Y (equiv.) | Base (equiv.) | Solvent (M) | Yield (%) |
|---|---|---|---|---|
| **1** | 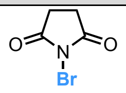 (2.0) | $Cs_2CO_3$ (1.0) | $CH_2Cl_2$ (0.05) | trace |
| **2** | 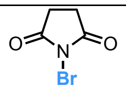 (2.0) | $Cs_2CO_3$ (1.0) | toluene (0.05) | – |
| **3** | 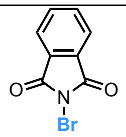 (2.0) | $Cs_2CO_3$ (1.0) | $CH_2Cl_2$ (0.05) | trace |
| **4** | Br–$CCl_3$ (2.0) | $Cs_2CO_3$ (1.0) | toluene (0.05) | 10 |
| **5** | Br–$CCl_3$ (2.0) | $Cs_2CO_3$ (1.0) | $CH_2Cl_2$ (0.1) | 42 |
| **6** | Br–$CCl_3$ (2.0) | $Cs_2CO_3$ (1.0) | $CH_2Cl_2$ (0.05) | 21 |
| **7** | 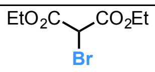 (2.0) | $Cs_2CO_3$ (1.0) | MeOH (0.1) | 37 |
| **8** | 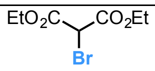 (2.0) | $Cs_2CO_3$ (1.0) | acetone (0.1) | - |
| **9** | 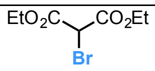 (2.0) | $Cs_2CO_3$ (1.0) | *i*-PrOH (0.1) | 43 |
| **10** | 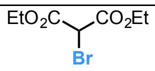 (2.0) | $Cs_2CO_3$ (1.0) | HFIP (0.1) | Trace |
| **11** | 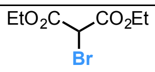 (2.0) | $Cs_2CO_3$ (1.0) | *i*-PrOH (0.2) | 7 |
| **12** | 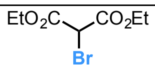 (2.0) | $Cs_2CO_3$ (1.0) | *i*-PrOH (0.05) | 30 |
| **13** | 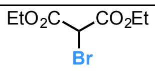 (2.0) | $K_2CO_3$ (1.0) | *i*-PrOH (0.1) | 60 |

| Entry | Br–Y (equiv.) | Base (equiv.) | Solvent (M) | Yield (%) |
|---|---|---|---|---|
| **14** | 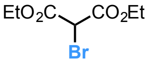 (2.0) | CsOBz (1.0) | *i*-PrOH (0.1) | 39 |
| **15** | 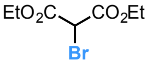 (3.0) | $K_2CO_3$ (1.0) | *i*-PrOH 0.1) | 45 |
| *Control experiments* | | | | |
| **16**[a] | 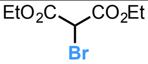 (2.0) | $K_2CO_3$ (1.0) | *i*-PrOH (0.1) | – |
| **17**[b] | 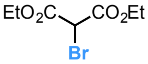 (2.0) | $K_2CO_3$ (1.0) | *i*-PrOH (0.1) | – |

[a] The reaction was run in the dark.

[b] The reaction was run without **PC1**.

## 5.4 Imino-iodination

### General Procedure for the Reaction Optimization – GP7

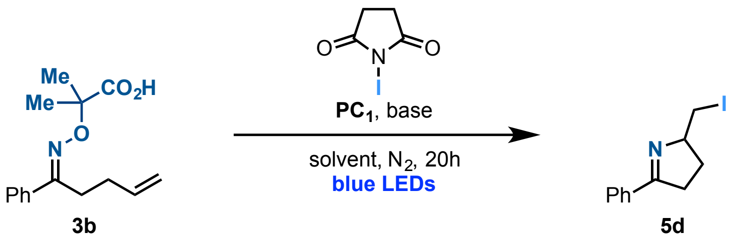


To dry tube was added **3b** (0.05 mmol, 1.0 equiv.), **PC1** (5 mol%), the base and NIS. A stirrer bar was added and the tube capped with a Supelco aluminium crimp seal with septum (PTFE/butyl). The tube was evacuated and refilled with $N_2$ (x 3). The solvent (dry and degassed by bubbling through with nitrogen for 20 mins) was added. The nitrogen inlet was then removed and the cap sealed with para-film. The mixture was stirred at room temperature for 20 h in front of blue LEDs. 1,3,5-Trimethoxybenzene (3.0 mg, 0.018 mmol, 0.28 equiv.) was added and the solvent removed under reduced pressure. $CDCl_3$ (0.4 mL) was added and the mixture was analysed by $^1$H NMR spectroscopy to determine the NMR yield.

The optimum reaction conditions identified by this optimisation study were:

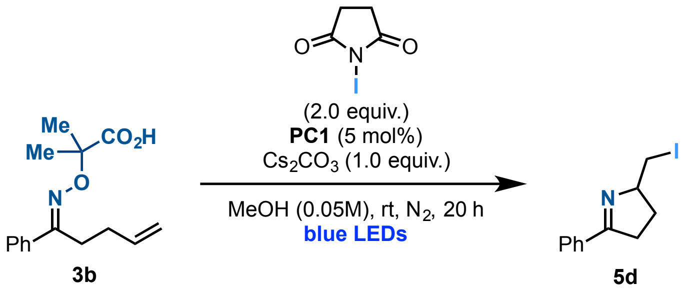


The following Table reports all the experiments performed.

| Entry | NIS (equiv.) | Base (equiv.) | Solvent (M) | Yield (%) |
|---|---|---|---|---|
| **1** | 2.0 | $Cs_2CO_3$ (1.0) | $CH_2Cl_2$ (0.1) | 28 |
| **2** | 2.0 | $Cs_2CO_3$ (1.0) | $CH_2Cl_2$ (0.05) | 17 |
| **3** | 2.0 | $Cs_2CO_3$ (1.0) | toluene (0.05) | 33 |
| **4** | 2.0 | $Cs_2CO_3$ (1.0) | MeOH (0.05) | 51 |
| **5** | 2.0 | $Cs_2CO_3$ (1.0) | $CH_3CN$ (0.05) | 50 |
| **6** | 2.0 | $K_2CO_3$ (1.0) | $CH_3CN$ (0.05) | 40 |
| **7** | 3.0 | $Cs_2CO_3$ (1.0) | $CH_3CN$ (0.05) | 40 |
| **8**[a] | 2.0 | $Cs_2CO_3$ (1.0) | MeOH (0.05) | 60 |
| **9**[b] | 2.0 | $Cs_2CO_3$ (1.0) | MeOH (0.05) | 67 |
| *Control Experiments* | | | | |
| **10**[c] | 2.0 | $Cs_2CO_3$ (1.0) | MeOH (0.05) | 14 |
| **11**[d] | 2.0 | $Cs_2CO_3$ (1.0) | MeOH (0.05) | 11 |

[a] Reaction time: 3 h.

[b] Reaction time: 8 h.

[c] The reaction was run in the dark.

[d] The reaction was run without **PC1**.

## 5.5 Imino-fluorination

### General Procedure for the Reaction Optimization – GP8

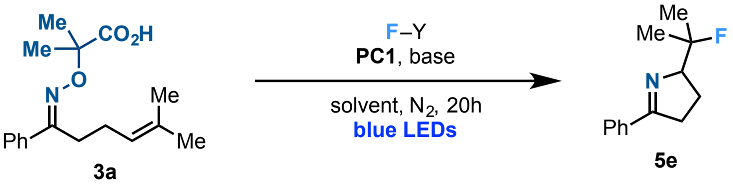


To dry tube was added **3a** (0.05 mmol, 1.0 equiv.), **PC1** (5 mol%), the base and the fluorinating agent. A stirrer bar was added and the tube capped with a Supelco aluminium crimp seal with septum (PTFE/butyl). The tube was evacuated and refilled with $N_2$ (x 3). The solvent (dry and degassed by bubbling through with nitrogen for 20 mins) was added. The nitrogen inlet was then removed and the cap sealed with para-film. The mixture was stirred at room temperature for 20 h in front of blue LEDs. 1,3,5-Trimethoxybenzene (3.0 mg, 0.018 mmol, 0.28 equiv.) was added and the solvent removed under reduced pressure. $CDCl_3$ (0.4 mL) was added and the mixture was analysed by $^1$H NMR spectroscopy to determine the NMR yield.

The optimum reaction conditions identified by this optimisation study were:

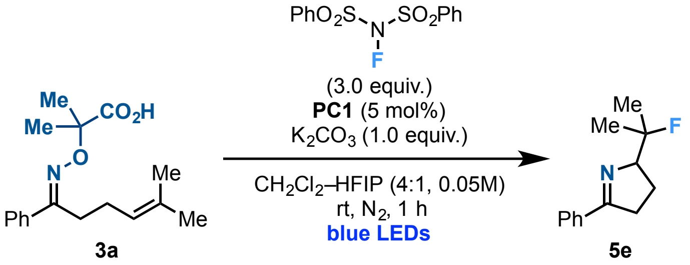


The following Table reports all the experiments performed.

| Entry | F–Y (equiv.) | Base (equiv.) | Solvent (M) | Yield (%) |
|---|---|---|---|---|
| **1** | 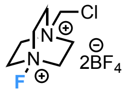  (2.0) | $Cs_2CO_3$ (1.0) | $CH_2Cl_2$:$H_2O$ 1:1 (0.1) | 7 |
| **2** | 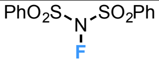  (2.0) | $Cs_2CO_3$ (1.0) | $CH_2Cl_2$ (0.1) | 6 |
| **3** | 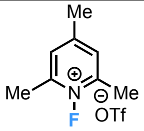  (2.0) | $Cs_2CO_3$ (1.0) | $CH_2Cl_2$ (0.1) | traces |
| **4** | 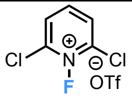  (2.0) | $Cs_2CO_3$ (1.0) | $CH_2Cl_2$ (0.05) | – |
| **5** | 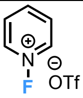  (2.0) | $Cs_2CO_3$ (1.0) | $CH_2Cl_2$ (0.05) | – |
| **6** | 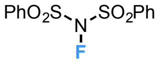  (2.0) | $Cs_2CO_3$ (1.0) | $CH_2Cl_2$ (0.05) | 33 |
| **7** | 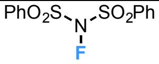  (2.0) | $Cs_2CO_3$ (1.0) | $CH_2Cl_2$ (0.025) | 25 |
| **8** | 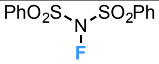  (2.0) | $Cs_2CO_3$ (1.0) | DCE (0.1) | traces |
| **9** | 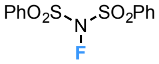  (2.0) | $Cs_2CO_3$ (1.0) | MeOH (0.1) | 20 |
| **10** | 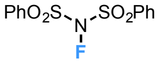  (2.0) | $Cs_2CO_3$ (1.0) | MeOH (0.05) | 22 |
| **11** | 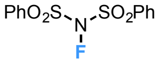  (2.0) | $Cs_2CO_3$ (1.0) | MeCN (0.1) | 30 |

| Entry | F–Y (equiv.) | Base (equiv.) | Solvent (M) | Yield (%) |
|---|---|---|---|---|
| **12** | 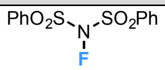 (2.0) | $Cs_2CO_3$ (1.0) | MeCN (0.05) | 6 |
| **13** | 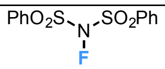 (2.0) | $Cs_2CO_3$ (1.0) | MeCN (0.2) | trace |
| **14** | 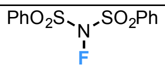 (2.0) | $Cs_2CO_3$ (1.0) | DCE (0.05) | 6 |
| **15** | 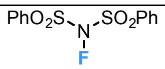 (2.0) | $Cs_2CO_3$ (1.0) | $CHCl_3$ (0.05) | 30 |
| **16** | 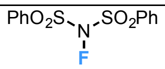 (2.0) | $Cs_2CO_3$ (1.0) | *i*-PrOH (0.05) | – |
| **17** | 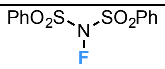 (2.0) | $Cs_2CO_3$ (1.0) | $CF_3CH_2OH$ (0.05) | 30 |
| **18** | 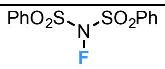 (2.0) | $Cs_2CO_3$ (1.0) | HFIP (0.05) | 61 |
| **19** | 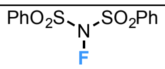 (2.0) | $Cs_2CO_3$ (1.0) | HFIP (0.1) | 40 |
| **20** | 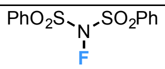 (2.0) | $Cs_2CO_3$ (1.0) | $CH_2Cl_2$:HFIP 1:1 (0.05) | 61 |
| **21** | 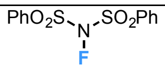 (2.0) | $Cs_2CO_3$ (1.0) | $CH_3CN$:HFIP 1:1 (0.05) | 26 |
| **22** | 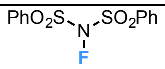 (2.0) | $Cs_2CO_3$ (1.0) | $CH_2Cl_2$:HFIP 2:1 (0.05) | 60 |
| **23** | 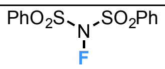 (2.0) | $Cs_2CO_3$ (1.0) | $CH_2Cl_2$:HFIP 4:1 (0.05) | 59 |
| **24** | 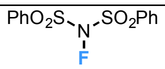 (2.0) | $Cs_2CO_3$ (1.0) | $CH_2Cl_2$:HFIP 10:1 (0.05) | 10 |

| Entry | F–Y (equiv.) | Base (equiv.) | Solvent (M) | Yield (%) |
|---|---|---|---|---|
| **25** | $PhO_2S{-}N(F){-}SO_2Ph$ (2.0) | $K_2CO_3$ (1.0) | $CH_2Cl_2$:HFIP 4:1 (0.05) | 70 |
| **26** | $PhO_2S{-}N(F){-}SO_2Ph$ (2.0) | $Na_2HPO_4$ (1.0) | $CH_2Cl_2$:HFIP 4:1 (0.05) | 65 |
| **27** | $PhO_2S{-}N(F){-}SO_2Ph$ (2.0) | $K_2CO_3$ (1.0) | $CH_2Cl_2$:HFIP 4:1 (0.05) | – |
| **28** | $PhO_2S{-}N(F){-}SO_2Ph$ (3.0) | $K_2CO_3$ (1.0) | $CH_2Cl_2$:HFIP 4:1 (0.05) | 80 |
| *Control Experiments* | | | | |
| **29**[a] | $PhO_2S{-}N(F){-}SO_2Ph$ (2.0) | $K_2CO_3$ (1.0) | $CH_2Cl_2$:HFIP 4:1 (0.05) | – |
| **30**[b] | $PhO_2S{-}N(F){-}SO_2Ph$ (2.0) | $K_2CO_3$ (1.0) | $CH_2Cl_2$:HFIP 4:1 (0.05) | – |
| **30** | – | $Cs_2CO_3$ (1.0) | HFIP (0.05) | – |

[a] The reaction was run in the dark.

[b] The reaction was run without **PC1**.

## 5.6 Imino-azidation

### General Procedure for the Reaction Optimization – GP9

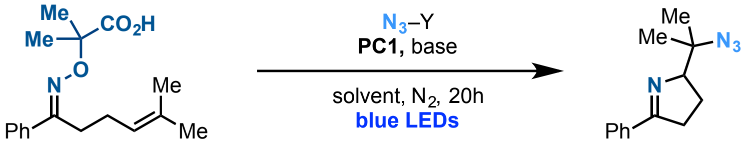


3a 5f

To dry tube was added **3a** (0.05 mmol, 1.0 equiv.), **PC1** (5 mol%), the base and the azidating agent (if solid). A stirrer bar was added and the tube capped with a Supelco aluminium crimp seal with septum (PTFE/butyl). The tube was evacuated and refilled with $N_2$ (x 3). The solvent (dry and degassed by bubbling through with nitrogen for 20 mins) was added and the azidating agent (if liquid, this was also degassed by bubbling through with nitrogen after addition). The nitrogen inlet was then removed and the cap sealed with para-film. The mixture was stirred at room temperature for 20 h in front of blue LEDs. 1,3,5-Trimethoxybenzene (3.0 mg, 0.018 mmol, 0.28 equiv.) was added and the solvent removed under reduced pressure. $CDCl_3$ (0.4 mL) was added and the mixture was analysed by $^1H$ NMR spectroscopy to determine the NMR yield.

The optimum reaction conditions identified by this optimisation study were:

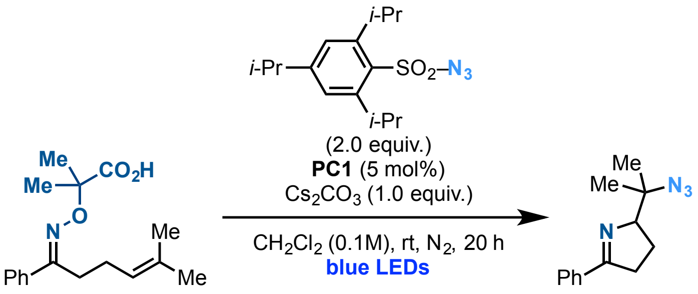


3a 5f

The following Table reports all the experiments performed.

| Entry | $N_3$–Y (equiv.) | Base (equiv.) | Solvent (M) | Yield (%) |
|---|---|---|---|---|
| **1** | 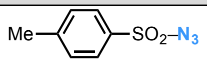 (2.0)[a] | $Cs_2CO_3$ (1.0) | toluene (0.1) | 17 |
| **2** | 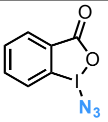 (2.0) | $Cs_2CO_3$ (1.0) | $CH_2Cl_2$ (0.1) | 14 |
| **3** | 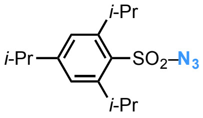 (2.0)[b] | $Cs_2CO_3$ (1.0) | $CH_2Cl_2$ (0.1) | 95 |
| **4** | 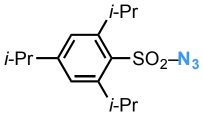 (2.0)[a] | $Cs_2CO_3$ (2.0) | toluene (0.1) | 95 |
| | | *Control Experiments* | | |
| **5**[d] | 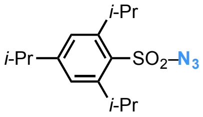 (2.0)[b] | $Cs_2CO_3$ (1.0) | DCM (0.1) | - |
| **6**[e] | 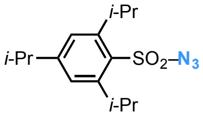 (2.0)[b] | $Cs_2CO_3$ (1.0) | DCM (0.1) | - |

[a] 10–15% wt solution in toluene

[b] 10% wt $H_2O$ as stabiliser.

[c] 10–12% wt solution in toluene

[d] The reaction was run in the dark.

[e] The reaction was run without **PC1**.

## 5.7 Imino-amination

### General Procedure for the Reaction Optimization – GP10

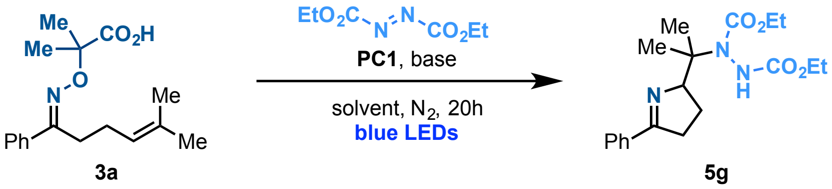


To dry tube was added **3a** (0.05 mmol, 1.0 equiv.), **PC1** (5 mol%) and the base. A stirrer bar was added and the tube capped with a Supelco aluminium crimp seal with septum (PTFE/butyl). The tube was evacuated and refilled with $N_2$ (x 3). The solvent (dry and degassed by bubbling through with nitrogen for 20 mins) was added and diethyl (*E*)-diazene-1,2-dicarboxylate. The nitrogen inlet was then removed and the cap sealed with para-film. The mixture was stirred at room temperature for 20 h in front of blue LEDs. 1,3,5-Trimethoxybenzene (3.0 mg, 0.018 mmol, 0.28 equiv.) was added and the solvent removed under reduced pressure. $CDCl_3$ (0.4 mL) was added and the mixture was analysed by $^1H$ NMR spectroscopy to determine the NMR yield.

The optimum reaction conditions identified by this optimisation study were:

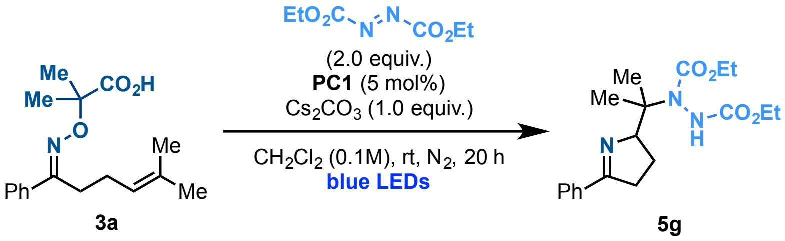


The following Table reports all the experiments performed.

| Entry | 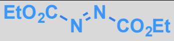 (equiv.) | Base (equiv.) | Solvent (M) | Yield (%) |
|---|---|---|---|---|
| **1** | 2.0 | $Cs_2CO_3$ (1.0) | $CH_2Cl_2$ (0.1) | 82 |
| *Control Experiments* | | | | |
| **2**[a] | 2.0 | $Cs_2CO_3$ (1.0) | $CH_2Cl_2$ (0.1) | – |
| **3**[b] | 2.0 | $Cs_2CO_3$ (1.0) | $CH_2Cl_2$ (0.1) | – |

[a] The reaction was run in the dark.

[b] The reaction was run without **PC1**.

## 5.8 Imino-thioetherificaiton

### General Procedure for the Reaction Optimization – GP11

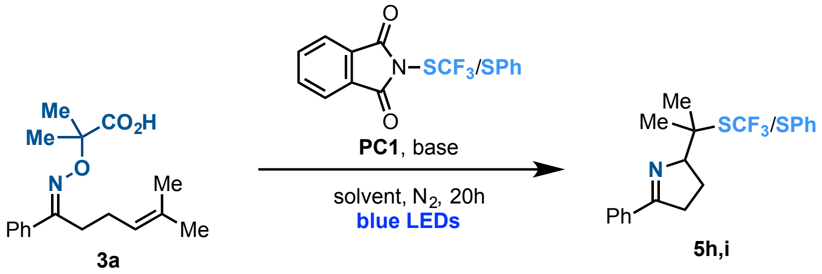


To dry tube was added **xx** (0.05 mmol, 1.0 equiv.), **PC1** (5 mol%), the base and the thioetherification agent. A stirrer bar was added and the tube capped with a Supelco aluminium crimp seal with septum (PTFE/butyl). The tube was evacuated and refilled with $N_2$ (x 3). The solvent (dry and degassed by bubbling through with nitrogen for 20 mins) was added. The nitrogen inlet was then removed and the cap sealed with para-film. The mixture was stirred at room temperature for 20 h in front of blue LEDs. 1,3,5-Trimethoxybenzene (3.0 mg, 0.018 mmol, 0.28 equiv.) was added and the solvent removed under reduced pressure. $CDCl_3$ (0.4 mL) was added and the mixture was analysed by $^1$H NMR spectroscopy to determine the NMR yield.

The optimum reaction conditions identified by this optimisation study were:

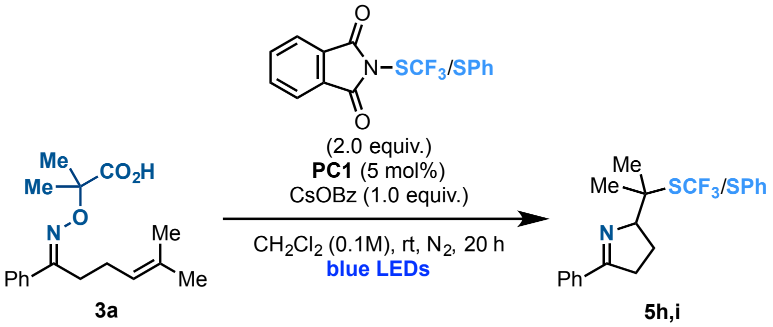


The following Table reports all the experiments performed.

| Entry | RS–Y (equiv.) | Base (equiv.) | Solvent (M) | Yield (%) |
| --- | --- | --- | --- | --- |
| **1** | 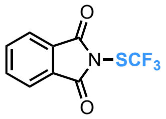<br>(2.0) | $Cs_2CO_3$ (1.0) | $CH_2Cl_2$ (0.1) | 26 |
| **2** | 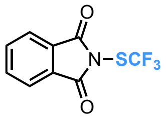<br>(2.0) | $Cs_2CO_3$ (1.0) | toluene (0.1) | Trace |
| **3** | 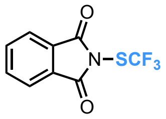<br>(2.0) | $Cs_2CO_3$ (1.0) | DCE (0.1) | 13 |
| **4** | 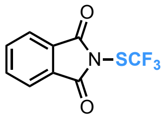<br>(2.0) | $Cs_2CO_3$ (1.0) | $CH_3CN$ (0.1) | 15 |
| **5** | 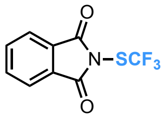<br>(2.0) | $Cs_2CO_3$ (1.0) | $C_6H_5CF_3$ (0.1) | 22[a] |
| **6** | 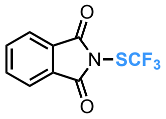<br>(2.0) | $Cs_2CO_3$ (1.0) | $CH_2Cl_2$ (0.05) | 15 |
| **7** | 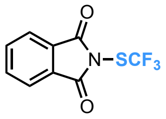<br>(2.0) | $Cs_2CO_3$ (1.0) | $CH_2Cl_2$ (0.2) | 15 |
| **8** | 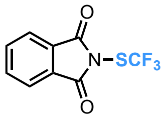<br>(2.0) | $Cs_2CO_3$ (1.0) | $CH_2Cl_2$ (0.1) | 25[a] |
| **9** | 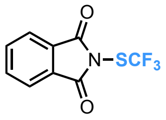<br>(2.0) | $Cs_2CO_3$(1.0)<br>$Bu_4NBr$ (1.0) | $CH_2Cl_2$ (0.1) | 27[a] |

| **Entry** | **RS–Y (equiv.)** | **Base (equiv.)** | **Solvent (M)** | **Yield (%)** |
|---|---|---|---|---|
| **10** | 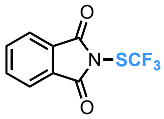 (2.0) | $K_2CO_3$ (1.0) | $CH_2Cl_2$ (0.1) | 37[b] |
| **11** | 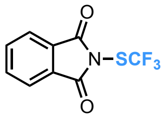 (2.0) | CsOBz (1.0) | $CH_2Cl_2$ (0.1) | 55[b] |
| **12** | 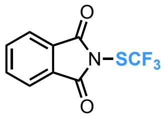 (3.0) | CsOBz (1.0) | $CH_2Cl_2$ (0.1) | 49[b] |
| **13** | 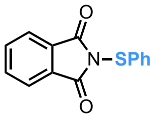 (2.0) | $Cs_2CO_3$ (1.0) | $CH_2Cl_2$ (0.1) | 45 |
| **14** | 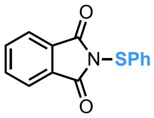 (2.0) | $Cs_2CO_3$ (1.0) | $CH_2Cl_2$ (0.05) | 48 |
| **15** | 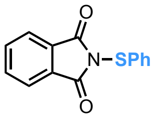 (3.0) | $Cs_2CO_3$ (1.0) | $CH_2Cl_2$ (0.05) | 56 |
| **16** | 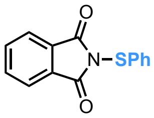 (2.0) | CsOBz (1.0) | $CH_2Cl_2$ (0.05) | 27 |
| *Control Experiments* | | | | |
| **17**[c] | 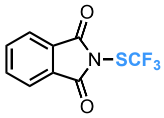 (2.0) | CsOBz (1.0) | $CH_2Cl_2$ (0.1) | – |
| **18**[d] | 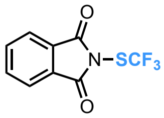 (2.0) | CsOBz (1.0) | $CH_2Cl_2$ (0.1) | – |

| Entry | RS–Y (equiv.) | Base (equiv.) | Solvent (M) | Yield (%) |
|---|---|---|---|---|

[a] Reaction time 2 h

[b] Reaction time 14 h

[c] The reaction was run in the dark.

[d] The reaction was run without **PC1**.

## 5.9 Imino-selenation

### General Procedure for the Reaction Optimization – GP12

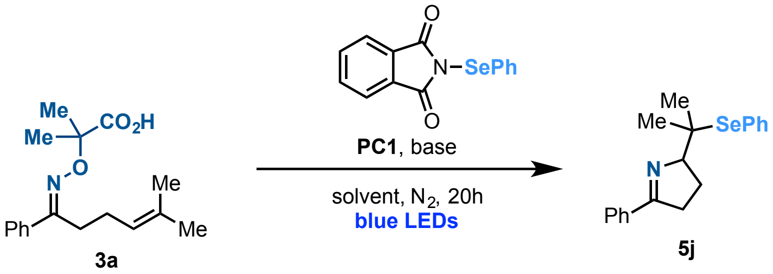


To dry tube was added **3a** (0.05 mmol, 1.0 equiv.), **PC1** (5 mol%), the base and 2-(phenylselanyl)isoindoline-1,3-dione. A stirrer bar was added and the tube capped with a Supelco aluminium crimp seal with septum (PTFE/butyl). The tube was evacuated and refilled with $N_2$ (x 3). The solvent (dry and degassed by bubbling through with nitrogen for 20 mins) was added. The nitrogen inlet was then removed and the cap sealed with para-film. The mixture was stirred at room temperature for 20 h in front of blue LEDs. 1,3,5-Trimethoxybenzene (3.0 mg, 0.018 mmol, 0.28 equiv.) was added and the solvent removed under reduced pressure. $CDCl_3$ (0.4 mL) was added and the mixture was analysed by $^1$H NMR spectroscopy to determine the NMR yield.

The optimum reaction conditions identified by this optimisation study were:

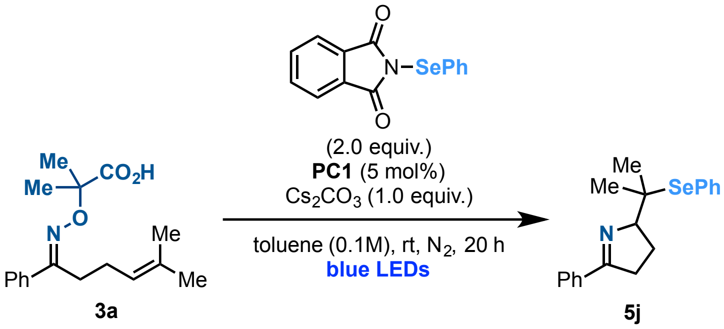


The following Table reports all the experiments performed.

| Entry | 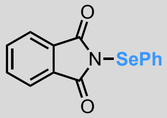 (equiv.) | Base (equiv.) | Solvent (M) | Yield (%) |
|---|---|---|---|---|
| **1** | 2.0 | $Cs_2CO_3$ (1.0) | $CH_2Cl_2$ (0.1) | 55 |
| **1** | 2.0 | $Cs_2CO_3$ (1.0) | toluene (0.05) | 80 |
| | | *Control Experiments* | | |
| **2**[a] | 2.0 | $Cs_2CO_3$ (1.0) | $CH_2Cl_2$ (0.1) | – |
| **3**[b] | 2.0 | $Cs_2CO_3$ (1.0) | $CH_2Cl_2$ (0.1) | – |

[a] The reaction was run in the dark.

[b] The reaction was run without **PC1**.

## 5.10 Imino-Michael

### General Procedure for the Reaction Optimization – GP13

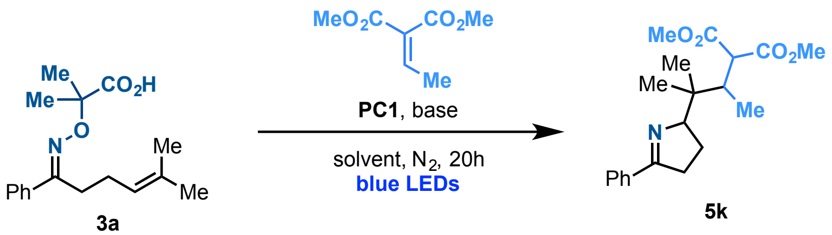

3a 5k

To dry tube was added **3a** (0.05 mmol, 1.0 equiv.), **PC1** (5 mol%), the base. A stirrer bar was added and the tube capped with a Supelco aluminium crimp seal with septum (PTFE/butyl). The tube was evacuated and refilled with $N_2$ (x 3). The solvent (dry and degassed by bubbling through with nitrogen for 20 mins) and dimethyl 2-ethylidenemalonate were added. The nitrogen inlet was then removed and the cap sealed with para-film. The mixture was stirred at room temperature for 20 h in front of blue LEDs. 1,3,5-Trimethoxybenzene (3.0 mg, 0.018 mmol, 0.28 equiv.) was added and the solvent removed under reduced pressure. $CDCl_3$ (0.4 mL) was added and the mixture was analysed by $^1H$ NMR spectroscopy to determine the NMR yield.

The optimum reaction conditions identified by this optimisation study were:

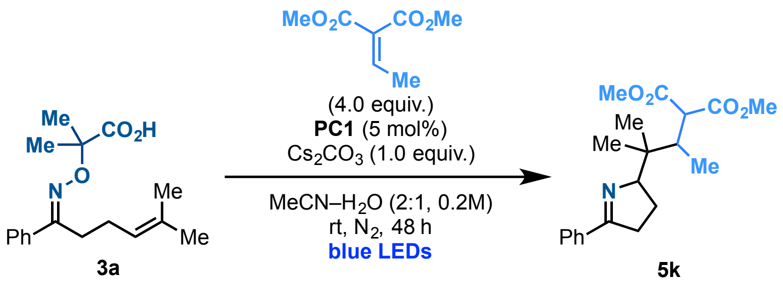

3a 5k

The following Table reports all the experiments performed.

| Entry | 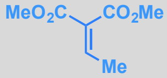 (equiv.) | Base (equiv.) | Solvent (M) | Yield (%) |
|---|---|---|---|---|
| **1** | 1.5 | $Cs_2CO_3$ (0.2) | MeCN (0.2) | 12 |
| **2** | 1.5 | $Cs_2CO_3$ (0.2) | MeOH (0.2) | 26 |
| **3** | 1.5 | $Cs_2CO_3$ (0.2) | MeCN:$H_2O$ 2:1 (0.2) | 29 |
| **4** | 1.5 | $Cs_2CO_3$ (0.2) | MeOH:$H_2O$ 2:1 (0.2) | 16 |
| **5** | 1.5 | $Cs_2CO_3$ (0.2) | MeCN:$H_2O$ 2:1 (0.3) | 25 |
| **6** | 1.5 | $Cs_2CO_3$ (0.2) | MeCN:$H_2O$ 2:1 (0.1) | 22 |
| **7** | 1.5 | $Cs_2CO_3$ (0.2) | MeOH:$H_2O$ 1:1 (0.2) | 22 |
| **8** | 1.5 | $Cs_2CO_3$ (0.2) | MeOH:$H_2O$ 4:1 (0.2) | 26 |
| **9** | 1.5 | $Cs_2CO_3$ (1.0) | MeCN:$H_2O$ 2:1 (0.2) | 32 |
| **10** | 1.5 | CsF (1.0) | MeCN:$H_2O$ 2:1 (0.2) | 34 |
| **11** | 1.5 | CsOBz (1.0) | MeCN:$H_2O$ 2:1 (0.2) | 11 |
| **12** | 1.5 | $CsHCO_3$ (1.0) | MeCN:$H_2O$ 2:1 (0.2) | 28 |
| **13** | 1.5 | $K_2CO_3$ (1.0) | MeCN:$H_2O$ 2:1 (0.2) | 27 |
| **14** | 1.5 | KOAc (1.0) | MeCN:$H_2O$ 2:1 (0.2) | 34 |
| **15** | 2.5 | $Cs_2CO_3$ (1.0) | MeCN:$H_2O$ 2:1 (0.2) | 38 |
| **16** | 4.0 | $Cs_2CO_3$ (1.0) | MeCN:$H_2O$ 2:1 (0.2) | 44 |
| **17**[a] | 4.0 | $Cs_2CO_3$ (1.0) | MeCN:$H_2O$ 2:1 (0.2) | 55 |
| *Control Experiments* | | | | |
| **18**[b] | 2.0 | $Cs_2CO_3$ (1.0) | $CH_2Cl_2$ (0.1) | – |
| **19**[c] | 2.0 | $Cs_2CO_3$ (1.0) | $CH_2Cl_2$ (0.1) | – |

[a] The reaction time was 48h.

[b] The reaction was run in the dark.

[c] The reaction was run without **PC1**.

## 5.11 Imino-cyanation

### General Procedure for the Reaction Optimization – GP14

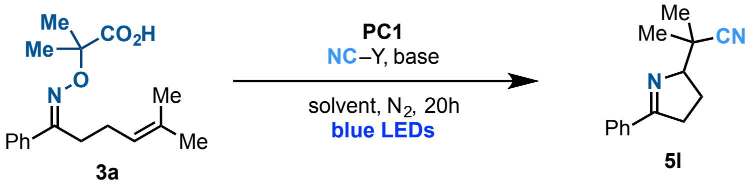


To dry tube was added **3a** (0.05 mmol, 1.0 equiv.), **PC1** (5 mol%), the base and the cyanating agent (if solid). A stirrer bar was added and the tube capped with a Supelco aluminium crimp seal with septum (PTFE/butyl). The tube was evacuated and refilled with $N_2$ (x 3). The solvent (dry and degassed by bubbling through with nitrogen for 20 mins) was added and the cyanating agent (if liquid). The nitrogen inlet was then removed and the cap sealed with parafilm. The mixture was stirred at room temperature for 20 h in front of blue LEDs. 1,3,5-Trimethoxybenzene (3.0 mg, 0.018 mmol, 0.28 equiv.) was added and the solvent removed under reduced pressure. $CDCl_3$ (0.4 mL) was added and the mixture was analysed by $^1$H NMR spectroscopy to determine the NMR yield.

The optimum reaction conditions identified by this optimisation study were:

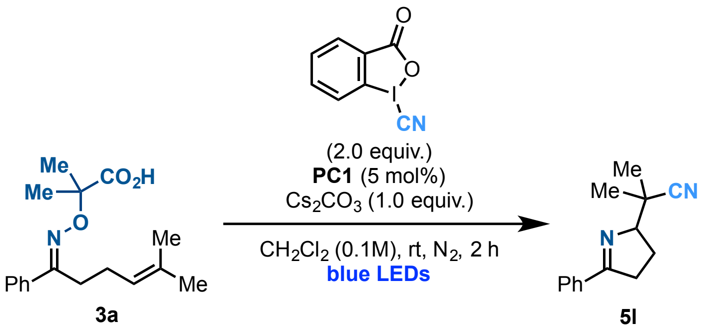


The following Table reports all the experiments performed.

| Entry | NC–Y (equiv.) | Base (equiv.) | Solvent (M) | Yield (%) |
|---|---|---|---|---|
| **1** | 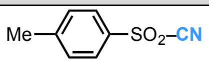 (2.0) | $Cs_2CO_3$ (1.0) | $CH_2Cl_2$ (0.1) | – |
| **2** | 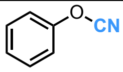 (2.0) | $Cs_2CO_3$ (1.0) | $CH_2Cl_2$ (0.1) | – |
| **3** | BrCN (2.0) | $Cs_2CO_3$ (1.0) | $CH_2Cl_2$ (0.1) | – |
| **4** | 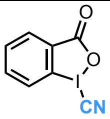 (2.0) | $Cs_2CO_3$ (2.0) | DMF (0.1) | Trace |
| **5** | 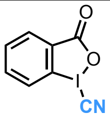 (2.0) | $Cs_2CO_3$ (1.0) | MeOH (0.1) | 25 |
| **6** | 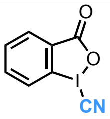 (2.0) | $Cs_2CO_3$ (1.0) | MeCN (0.1) | 21 |
| **7** | 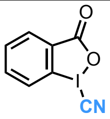 (2.0) | $Cs_2CO_3$ (1.0) | acetone (0.1) | 25 |
| **8** | 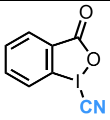 (2.0) | $Cs_2CO_3$ (1.0) | MeOH (0.05) | 38 |
| **9** | 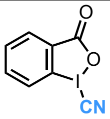 (2.0) | $Cs_2CO_3$ (1.0) | MeOH (0.2) | 20 |
| **10** | 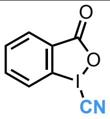 (2.0) | $Cs_2CO_3$ (1.0) | $CH_2Cl_2$ (0.1) | 50 |

| Entry | NC–Y (equiv.) | Base (equiv.) | Solvent (M) | Yield (%) |
|---|---|---|---|---|
| **11** | 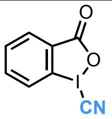 (2.0) | $Cs_2CO_3$ (1.0) | THF (0.1) | 47 |
| **12** | 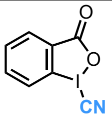 (2.0) | $Cs_2CO_3$ (1.0) | $CH_2Cl_2$ (0.05) | 50 |
| **13** | 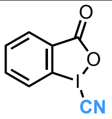 (2.0) | $Cs_2CO_3$ (1.0) | $CH_2Cl_2$ (0.2) | 50 |
| **14** | 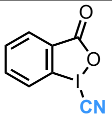 (2.0) | $Cs_2CO_3$ (1.0) | DCE (0.1) | 48 |
| **15** | 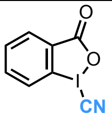 (2.0) | $Cs_2CO_3$ (1.0) | $CH_2Cl_2$ (0.1) | 55[a] |
| **16** | 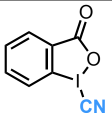 (2.0) | $Cs_2CO_3$ (1.0) | $CH_2Cl_2$ (0.1) | 63[b] |
| *Control Experiments* | | | | |
| **17**[c] | 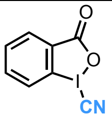 (2.0) | $Cs_2CO_3$ (1.0) | $CH_2Cl_2$ (0.1) | – |
| **18**[d] | 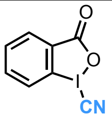 (2.0) | $Cs_2CO_3$ (1.0) | $CH_2Cl_2$ (0.1) | – |

| Entry | NC–Y (equiv.) | Base (equiv.) | Solvent (M) | Yield (%) |
|---|---|---|---|---|

[a] The reaction time was 30 min.

[b] The reaction time was 90 min.

[c] The reaction was run in the dark.

[d] The reaction was run without **PC1**.

## 5.12 Imino-olefination

### General Procedure for the Reaction Optimization – GP15

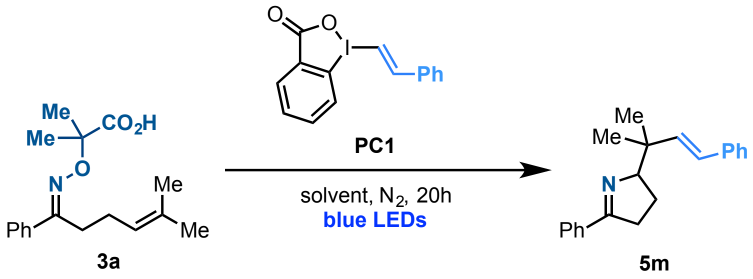


To dry tube was added **3a** (0.05 mmol, 1.0 equiv.), **PC1** (5 mol%), the base and **S3**. A stirrer bar was added and the tube capped with a Supelco aluminium crimp seal with septum (PTFE/butyl). The tube was evacuated and refilled with $N_2$ (x 3). The solvent (dry and degassed by bubbling through with nitrogen for 20 mins) was added. The nitrogen inlet was then removed and the cap sealed with para-film. The mixture was stirred at room temperature for 20 h in front of blue LEDs. 1,3,5-Trimethoxybenzene (3.0 mg, 0.018 mmol, 0.28 equiv.) was added and the solvent removed under reduced pressure. $CDCl_3$ (0.4 mL) was added and the mixture was analysed by $^1$H NMR spectroscopy to determine the NMR yield.

The optimum reaction conditions identified by this optimisation study were:

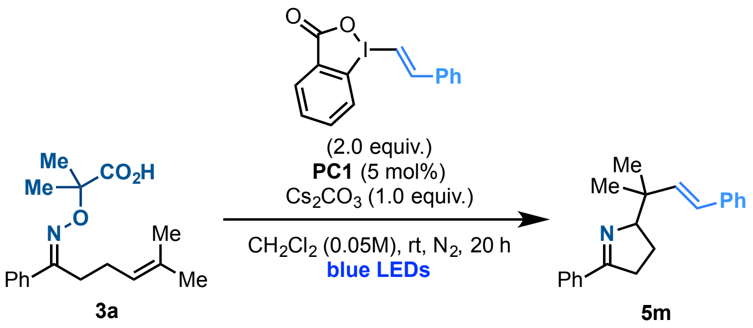


The following Table reports all the experiments performed.

| Entry | 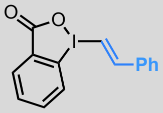 (equiv.) | Base (equiv.) | Solvent (M) | Yield (%) |
|---|---|---|---|---|
| **1** | 2.0 | $Cs_2CO_3$ (1.0) | $CH_2Cl_2$ (0.1) | 42 |
| **2** | 2.0 | $Cs_2CO_3$ (1.0) | THF (0.1) | 55 |
| **3** | 2.0 | $Cs_2CO_3$ (1.0) | $CH_2Cl_2$ (0.05) | 83 |
| **4** | 2.0 | $Cs_2CO_3$ (1.0) | THF (0.05) | 38 |
| *Control Experiments* | | | | |
| **5**[a] | 2.0 | $Cs_2CO_3$ (1.0) | $CH_2Cl$ (0.05) | – |
| **6**[b] | 2.0 | $Cs_2CO_3$ (1.0) | $CH_2Cl$ (0.05) | – |

[a] The reaction was run in the dark.

[b] The reaction was run without **PC1**.

## 5.13 Imino-alkynylation

### General Procedure for the Reaction Optimization – GP16

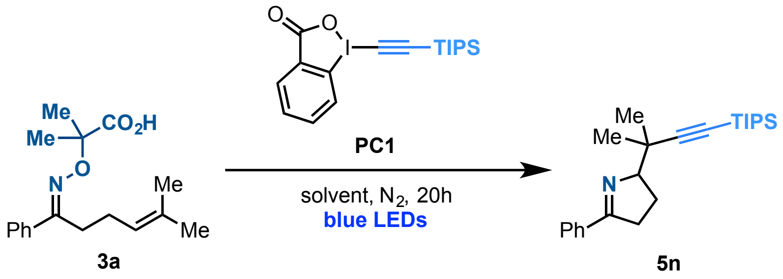


To dry tube was added **30** (0.05 mmol, 1.0 equiv.), **PC1** (5 mol%), the base and 1-((triisopropylsilyl)ethynyl)-1λ$^3$-benzo[*d*][1,2]iodaoxol-3(1*H*)-one. A stirrer bar was added and the tube capped with a Supelco aluminium crimp seal with septum (PTFE/butyl). The tube was evacuated and refilled with $N_2$ (x 3). The solvent (dry and degassed by bubbling through with $N_2$ for 20 mins) was added. The nitrogen inlet was then removed and the cap sealed with para-film. The mixture was stirred at room temperature for 20 h in front of blue LEDs. 1,3,5-Trimethoxybenzene (3.0 mg, 0.018 mmol, 0.28 equiv.) was added and the solvent removed under reduced pressure. $CDCl_3$ (0.4 mL) was added and the mixture was analysed by $^1$H NMR spectroscopy to determine the NMR yield.

The optimum reaction conditions identified by this optimisation study were:

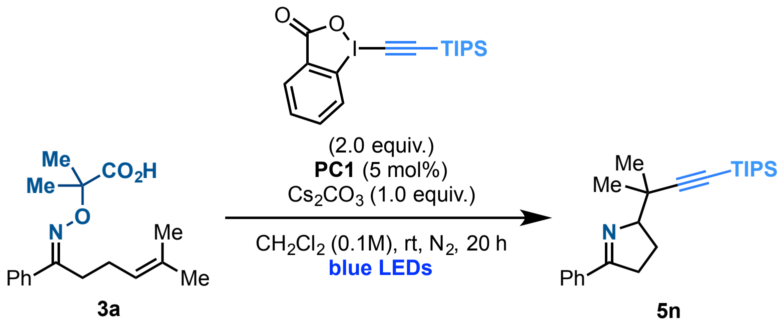


The following Table reports all the experiments performed.

| **Entry** | 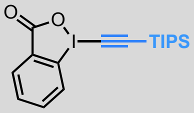 **(equiv.)** | **Base (equiv.)** | **Solvent (M)** | **Yield (%)** |
|---|---|---|---|---|
| **1** | 2.0 | $Cs_2CO_3$ (1.0) | $CH_2Cl_2$ (0.1) | 68 |
| | | *Control Experiments* | | |
| **2**[a] | 2.0 | $Cs_2CO_3$ (1.0) | $CH_2Cl_2$ (0.1) | – |
| **3**[b] | 2.0 | $Cs_2CO_3$ (1.0) | $CH_2Cl_2$ (0.1) | – |

[a] The reaction was run in the dark.

[b] The reaction was run without **PC1**.

## 6 Iminofunctionalization Reaction Products

### 6.1 General Procedure for the Purification of the Iminofunctionalization Products

In general we have found that almost all our reaction products can be purified by simple acid-base wash without flash column chromatography. A detailed procedure for the acid-base was is given below:

The crude mixture was dissolved in $Et_2O$ (5 mL) and transferred to a separating funnel. The organic layer was washed repeatedly (x 4) with a 1M HCl solution (5 mL). The combined aqueous layers were drained into a 1M KOH solution (25 mL). $CH_2Cl_2$ (10 mL) was added and the aqueous layer was extracted with $CH_2Cl_2$ (3 x 10 mL). The combined organic layers were dried ($Mg_2SO_4$), filtered and evaporated to give the pure product.

In some cases, due to presence of minor impurities (generally <5%) we have re-purified the product through quick column chromatography on silica gel.

### 6.2 Picture of Reaction Set-Up

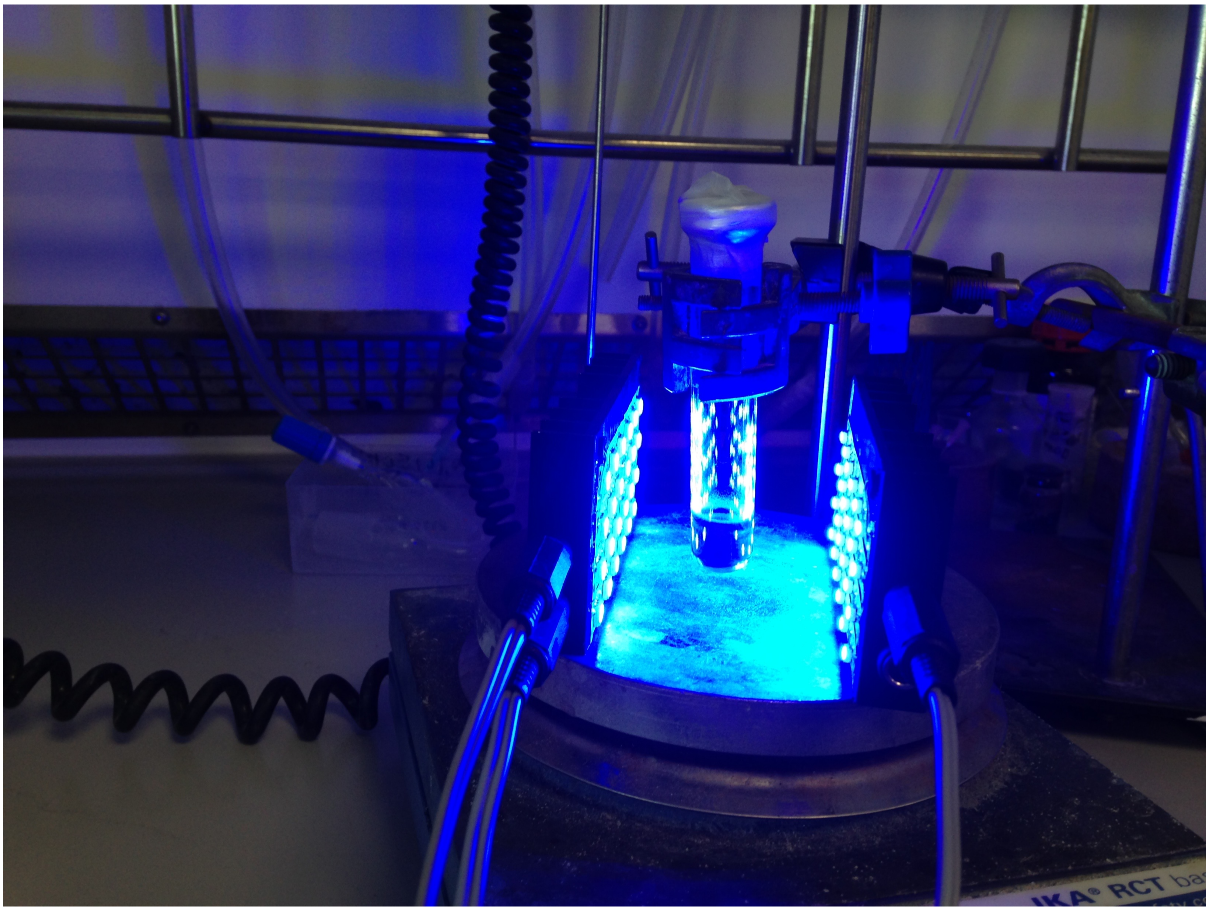

## 6.3 Product Structures

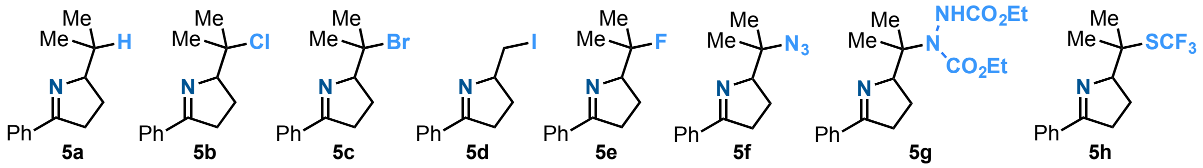


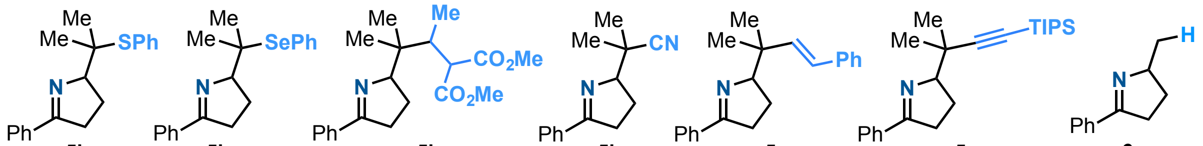


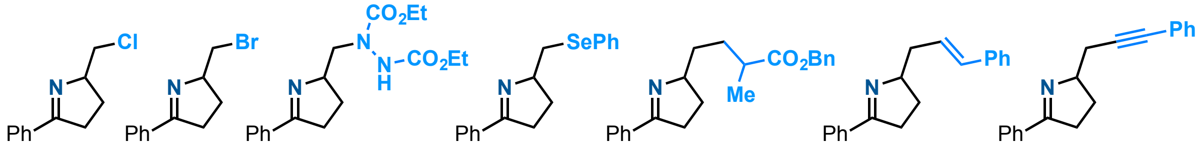


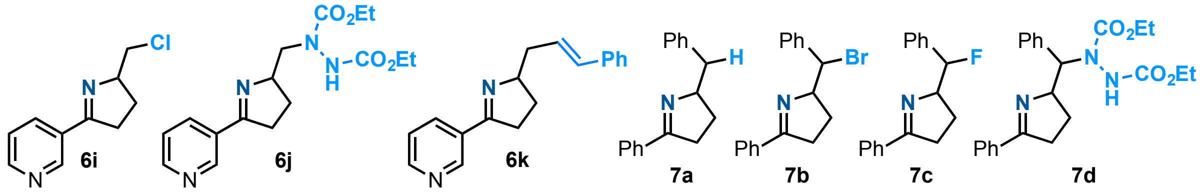


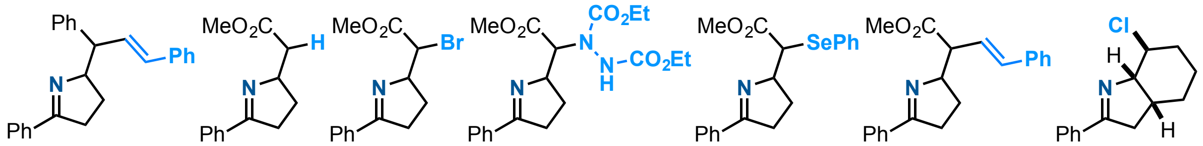


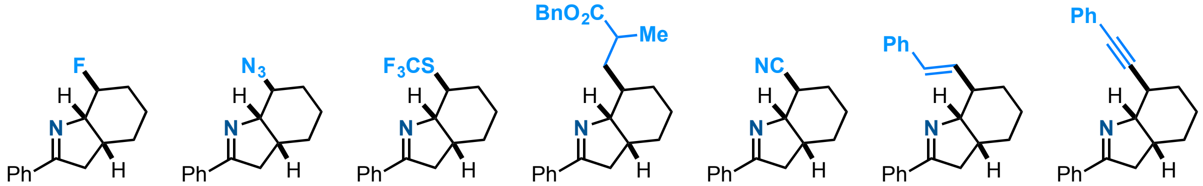


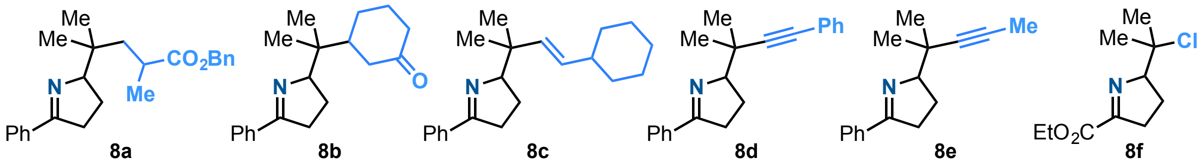


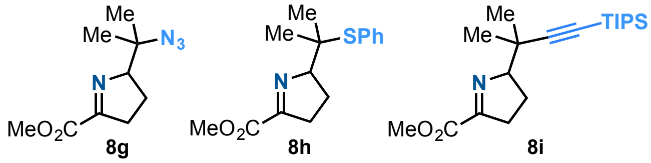


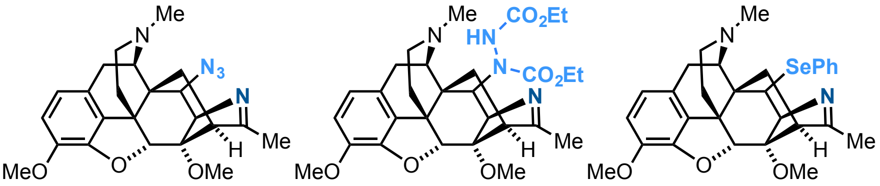

## 6.4 Product characterizations

**2-Isopropyl-5-phenyl-3,4-dihydro-2H-pyrrole (5a)**

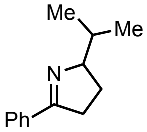


Following **GP4**, **3a** (29 mg, 0.10 mmol) gave **5a** (15 mg, 81%). FT-IR $\nu_{max}$ (film)/cm$^{-1}$ 2968, 1730, 1616, 1576, 1495, 1448, 1366, 1341, 1275, 1151, 1106, 1064, 1029; $^1$H NMR (400 MHz, $CDCl_3$) δ 7.88–7.81 (2H, m), 7.44–7.36 (3H, m), 4.02 (1H, q, *J* = 6.2 Hz), 3.01–2.93 (1H, m), 2.93–2.82 (1H, m), 2.13–2.02 (1H, m), 1.96 (1H, dq, *J* = 13.2, 6.6 Hz), 1.73–1.62 (1H, m), 1.08 (3H, d, *J* = 6.7 Hz), 0.91 (3H, d, *J* = 6.7 Hz); $^{13}$C NMR (101 MHz, $CDCl_3$) δ 172.0, 135.0, 130.3, 128.5, 127.8, 79.3, 35.4, 33.6, 25.2, 20.2, 18.5; HRMS (APCI): Found MH$^+$ 188.1434 $C_{13}H_{18}N$ requires 188.1434.

**2-(2-Chloropropan-2-yl)-5-phenyl-3,4-dihydro-2H-pyrrole (5b)**

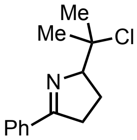


Following **GP5**, **3a** (43.5 mg, 0.15 mmol) gave **5b** (26 mg, 80%). FT-IR $\nu_{max}$ (film)/cm$^{-1}$ 2974, 1707, 1616, 1576, 1448, 1383, 1366, 1342, 1178, 1112, 1063; $^1$H NMR (400 MHz, $CDCl_3$) δ 7.88–7.85 (2H, m), 7.45–7.38 (3H, m), 4.34 (1H, ddt, *J* = 8.6, 6.9, 2.1 Hz), 3.06 (1H, dddd, *J* = 17.3, 10.2, 4.8, 2.3 Hz), 2.95 (1H, dddd, *J* = 17.2, 9.6, 7.4, 1.8 Hz), 2.24–2.15 (1H, m), 2.11–2.02 (1H, m), 1.74 (3H, s), 1.70 (3H, s); $^{13}$C NMR (101 MHz, $CDCl_3$) δ 174.1, 134.6, 130.7, 128.5, 128.0, 82.9, 73.7, 35.7, 31.3, 29.2, 24.9; HRMS (EI): Found M$^+$ 222.1041 $C_{13}H_{17}NCl$ requires 222.1044.

**2-(2-Bromopropan-2-yl)-5-phenyl-3,4-dihydro-2H-pyrrole (5c)**

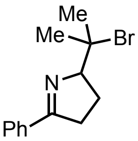


Following **GP6**, **3a** (29 mg, 0.1 mmol) gave **5c** (16 mg, 62%).[3] FT-IR $\nu_{max}$ (film)/cm$^{-1}$ 2921, 1628, 1598, 1512, 1449, 1394, 1360, 1296, 1111; $^1$H NMR (400 MHz, $CDCl_3$) δ 7.87 (2H, d, *J* = 7.5 Hz), 7.47–7.37 (3H, m), 4.23 (1H, br t, *J* = 7.2 Hz), 3.13–3.01 (1H, m), 3.01–2.88 (1H, m), 2.23 (1H, dtd, *J* = 13.6, 9.2, 4.7 Hz), 2.13–2.00 (1H, m), 1.91 (6H, s); $^{13}$C NMR

[3] After acid-base wash, **5c** was purified by column chromatography on silica gel eluting with $CH_2Cl_2$.

(101 MHz, $CDCl_3$) δ 174.0, 134.6, 130.7, 128.6, 128.0, 127.6, 83.6, 35.7, 32.8, 31.0, 26.2; HRMS (EI): Found $MH^+$ 266.0538 $C_{13}H_{17}NBr$ requires 266.0539.

**2-(Iodomethyl)-5-phenyl-3,4-dihydro-2H-pyrrole (5d)**

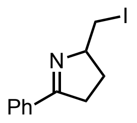

Following **GP7**, **3b** (39 mg, 0.15 mmol) gave **5d** (29 mg, 66%). FT-IR $\nu_{max}$ (film)/$cm^{-1}$ 2965, 2925, 1618, 1572, 1493, 1449, 1410, 1339, 1316, 1279, 1264, 1202, 1128, 1076; $^1H$ NMR (400 MHz, $CDCl_3$) δ 7.80–7.75 (2H, m), 7.39–7.30 (3H, m), 4.35–4.27 (1H, m), 3.53 (1H, dd, *J* = 9.8, 4.0 Hz), 3.41 (1H, dd, *J* = 9.8, 6.6 Hz), 3.02 (1H, dddd, *J* = 17.3, 10.2, 4.9, 2.2 Hz), 2.86 (1H, dddd, *J* = 17.3, 10.1, 7.2, 1.6 Hz), 2.21 (1H, dddd, *J* = 13.0, 10.2, 8.0, 4.9 Hz), 1.71 (1H, dddd, *J* = 13.3, 10.2, 7.1, 6.4 Hz); $^{13}C$ NMR (101 MHz, $CDCl_3$) δ 174.2, 134.3, 130.9, 128.6, 128.0, 73.0, 35.8, 29.2, 14.2; HRMS (EI): Found $MH^+$ 285.0076 $C_{11}H_{13}NI$ requires 285.0087.

**2-(2-Fluoropropan-2-yl)-5-phenyl-3,4-dihydro-2H-pyrrole (5e)**

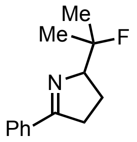

Following **GP8**, **3a** (45 mg, 0.15 mmol) gave **5e** (22 mg, 73%). FT-IR $\nu_{max}$ (film)/$cm^{-1}$ 2979, 1616, 1576, 1495, 1446, 1382, 1370, 1343, 1237, 1205, 1152, 1061; $^1H$ NMR (400 MHz, $CDCl_3$) δ 7.88 (2H, d, *J* = 6.9 Hz), 7.47–7.37 (3H, m), 4.29 (1H, dt, *J* = 15.7, 7.6 Hz), 3.10–2.99 (1H, m), 2.95 (1H, dt, *J* = 17.0, 8.6 Hz), 2.20–2.09 (1H, m), 2.03–1.92 (1H, m), 1.49 (3H, d, *J* = 21.6 Hz) 1.46 (3H, d, *J* = 21.6 Hz); $^{13}C$ NMR (101 MHz, $CDCl_3$) δ 174.1, 134.5, 130.7, 128.5, 128.0, 97.2 (d, *J* = 170.0 Hz), 80.2 (d, *J* = 23.0 Hz), 35.5, 25.2 (d, *J* = 23.9 Hz), 23.9 (d, *J* = 24.4 Hz), 23.7 (d, *J* = 4.6 Hz); $^{19}F$ NMR (376 MHz, $CDCl_3$) δ –148.2; HRMS (EI): Found $M^+$ 206.1334 $C_{13}H_{17}NF$ requires 206.1340.

**2-(2-Azidopropan-2-yl)-5-phenyl-3,4-dihydro-2H-pyrrole (5f)**

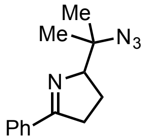

Following **GP9**, **3a** (43.5 mg, 0.15 mmol) gave **5f** (32 mg, 95%). $^1$H NMR (400 MHz, $CDCl_3$) δ 7.86 (2H, d, *J* = 7.3 Hz), 7.46–7.38 (3H, m), 4.18 (1H, t, *J* = 7.4 Hz), 3.09–2.99 (1H, m), 2.99–2.89 (1H, m), 2.17–2.06 (1H, m), 1.94–1.83 (1H, m), 1.47 (3H, s), 1.34 (3H, s); $^{13}$C NMR (101 MHz, $CDCl_3$) δ 174.2, 134.5, 130.7, 128.5, 127.9, 81.5, 64.3, 53.6, 35.6, 24.3, 23.7. Data in accordance with the literature.[18]

**Diethyl 1-(2-(5-Phenyl-3,4-dihydro-2H-pyrrol-2-yl)propan-2-yl)hydrazine-1,2-dicarboxylate (5g)**

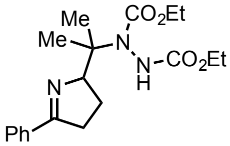

Following **GP10**, **3a** (29 mg, 0.1 mmol) gave **5g** (29 mg, 82%).[4] FT-IR $\nu_{max}$ (film)/cm$^{-1}$ 3304, 2982, 1707, 1616, 1467, 1447, 1400, 1374, 1330, 1220, 1177, 1081, 1056; $^1$H NMR (500 MHz, toluene-$d_8$, T = 100 ºC)[5] δ 7.72–7.65 (2H, m), 7.14–7.07 (3H, m), 6.69 (1H, br s), 4.96 (1H, br s), 4.03 (4H, br q, *J* = 6.4 Hz), 2.67–2.57 (1H, m), 2.49–2.38 (1H, m), 1.85–1.76 (1H, m), 1.65–1.52 (1H, m), 1.09 (3H, t, *J* = 6.3 Hz), 1.09 (3H, t, *J* = 6.8 Hz); $^{13}$C NMR (125 MHz, toluene-$d_9$, T = 100 ºC)[5] δ 172.5, 156.6, 156.5, 135.4, 130.4, 128.6, 128.4, 79.4, 65.7, 61.7, 61.4, 35.4, 25.0, 24.5, 21.5, 14.7, 14.6; HRMS (EI): Found MH$^+$ 362.2064 $C_{19}H_{28}O_4N_3$ requires 362.2074.

**5-Phenyl-2-(2-((trifluoromethyl)thio)propan-2-yl)-3,4-dihydro-2H-pyrrole (5h)**

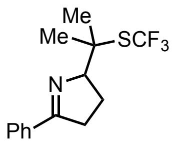

Following **GP11**, **3a** (29 mg, 0.1 mmol) gave **5h** (16 mg, 55%).[6] FT-IR $\nu_{max}$ (film)/cm$^{-1}$ 2970, 2363, 1717, 1616, 1576, 1448, 1368, 1342, 1095, 1029; $^1$H NMR (500 MHz, $CDCl_3$) δ 7.85 (2H, br d, *J* = 7.9 Hz), 7.47–7.38 (3H, m), 4.35 (1H, t, *J* = 7.7 Hz), 3.04 (1H, dddd, *J* =

[4] **5g** was purified by column chromatography on silica gel eluting with $CH_2Cl_2$→$CH_2Cl_2$:MeOH 99.5:0.5.
[5] The high temperature NMR analysis was required due to the presence of rotamers.
[6] After acid-base wash, **5h** was purified by column chromatography on silica gel eluting with $CH_2Cl_2$.

16.8, 10.2, 4.1, 2.3 Hz), 3.00–2.90 (1H, m), 2.26–2.17 (1H, m), 1.96 (1H, ddt, *J* = 13.3, 10.2, 7.9 Hz), 1.67 (3H, s), 1.52 (3H, s); $^{13}$C NMR (126 MHz, $CDCl_3$) δ 174.2, 134.4, 131.5 (q, *J* = 307.6 Hz),130.9, 128.6, 128.0, 81.8 (d, *J* = 1.2 Hz), 55.9, 35.7, 27.3 (d, *J* = 0.7 Hz), 25.9 (d, *J* = 0.9 Hz), 24.8; $^{19}$F NMR (376 MHz, $CDCl_3$) δ –34.44; HRMS (ESI): Found $MH^+$ 288.1028 $C_{14}H_{17}NF_3S$ requires 288.1028.

**5-Phenyl-2-(2-(phenylthio)propan-2-yl)-3,4-dihydro-2H-pyrrole (5i)**

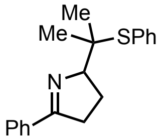


Following **GP11**, **3a** (29 mg, 0.1 mmol) gave **5i** (13 mg, 56%). FT-IR $\nu_{max}$ (film)/cm$^{-1}$ 2966, 1616, 1575, 1473, 1447, 1378, 1360, 1340, 1273, 1131, 1112, 1062, 1024; $^{1}$H NMR (500 MHz, $CDCl_3$) δ 7.84 (2H, dd, *J* = 7.6, 1.2 Hz), 7.60–7.55 (2H, m), 7.43–7.37 (3H, m), 7.35–7.28 (3H, m), 4.23 (1H, t, *J* = 7.5 Hz), 3.03 (1H, dddd, *J* = 15.0, 9.7, 5.1, 2.3 Hz), 2.94 (1H, dddd, *J* = 17.3, 9.5, 7.9, 1.6 Hz), 2.26–2.12 (2H, m), 1.45 (3H, s), 1.23 (3H, s); $^{13}$C NMR (126 MHz, $CDCl_3$) δ 173.2, 137.9, 134.8, 132.1, 130.5, 128.8, 128.6, 128.5, 127.9, 81.5, 53.3, 35.6, 27.9, 24.9, 24.4; HRMS (ASAP): Found $MH^+$ 296.1459 $C_{19}H_{22}NS$ requires 296.1467.

**5-Phenyl-2-(2-(phenylselanyl)propan-2-yl)-3,4-dihydro-2H-pyrrole (5j)**

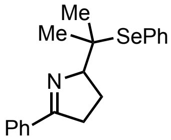


Following **GP12**, **3a** (43.5 mg, 0.15 mmol) gave **5j** (39 mg, 75%). FT-IR $\nu_{max}$ (film)/cm$^{-1}$ 2962, 1615, 1575, 1475, 1436, 1378, 1361, 1341, 1109, 1063; $^{1}$H NMR (400 MHz, $CDCl_3$) δ 7.81 (2H, d, *J* = 7.2 Hz), 7.64 (2H, d, *J* = 7.5 Hz), 7.43–7.29 (4H, m), 7.28–7.21 (2H, m), 4.25 (1H, t, *J* = 7.2 Hz), 3.07–2.97 (1H, m), 2.91 (1H, m), 2.22–2.11 (1H, m), 2.11–1.99 (1H, m), 1.48 (3H, s), 1.38 (3H, s); $^{13}$C NMR (101 MHz, $CDCl_3$) δ 173.2, 138.6, 134.7, 130.5, 128.7, 128.5, 128.5, 127.9, 127.8, 82.5, 51.9, 35.6, 28.3, 26.4, 25.6; HRMS (EI): Found $MH^+$ 344.0907 $C_{19}H_{22}NSe$ requires 344.0912.

**Dimethyl 2-(3-Methyl-3-(5-phenyl-3,4-dihydro-2H-pyrrol-2-yl)butan-2-yl)malonate (5k)**

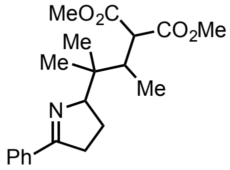


Following **GP13**, **3a** (29 mg, 0.1 mmol) gave **5k** (23 mg, 66%).[7] d.r 1.5:1. FT-IR $\nu_{max}$ (film)/cm$^{-1}$ 2952, 1731,1618, 1434, 1341, 1272, 1194, 1147; $^1$H NMR (500 MHz, $CDCl_3$, diastereomers) δ 7.88–7.82 (2H, m), 7.43–7.36 (3H, m), 4.22 (0.4H, t, *J* = 7.5 Hz), 4.19 (0.6H, t, *J* = 7.7 Hz), 4.04 (0.4H, d, *J* = 4.7 Hz), 3.94 (0.6H, d, *J* = 4.7 Hz), 3.74–3.72 (3H, m), 3.71 (1.8H, s), 3.67 (1.2H, s), 2.99–2.89 (1H, m), 2.89–2.80 (1H, m), 2.79–2.72 (0.6H, m), 2.64–2.57 (0.4H, m), 2.07–1.98 (1H, m), 1.84–1.70 (1H, m), 1.14 (1.8H, d, *J* = 7.2 Hz), 1.12 (1.2H, d, *J* = 7.2 Hz), 0.98 (1.2H, s), 0.87 (1.8H, s), 0.83 (1.8H, s), 0.80 (1.2H, s); $^{13}$C NMR (126 MHz, $CDCl_3$, diastereomers) δ 172.0$^m$ & 171.9$^M$, 171.1$^m$ & 170.9$^M$, 170.5$^m$ & 170$^M$, 135.0$^M$ & 134.9$^m$, 130.3, 128.5, 127.8$^M$ & 127.8$^m$, 79.8$^M$ & 79.2$^m$, 53.2$^M$ & 52.7$^m$, 52.6$^M$ & 52.6$^m$, 52.2, 40.7$^M$ & 40.5$^m$, 35.3$^m$ & 35.1$^M$, 24.3$^M$ & 24.0$^m$, 21.6$^M$ & 21.2$^m$, 20.9$^m$ & 19.0$^M$, 12.3$^M$ & 12.0$^m$; HRMS (ESI): Found MH$^+$ 346.2013 $C_{20}H_{28}O_4N$ requires 346.2013.

**2-Methyl-2-(5-phenyl-3,4-dihydro-2H-pyrrol-2-yl)propanenitrile (5l)**

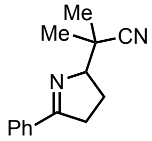


Following **GP14**, **3a** (29 mg, 0.1 mmol) gave **5l** (13 mg, 63%).[8] FT-IR $\nu_{max}$ (film)/cm$^{-1}$ 2976, 2931, 2231, 1618, 1574, 1447, 1369, 1343, 1278, 1193, 1063; $^1$H NMR (400 MHz, $CDCl_3$) δ 7.87 (2H, d, *J* = 7.5 Hz), 7.48–7.38 (3H, m), 4.11 (1H, t, *J* = 7.6 Hz), 3.20–3.09 (1H, m), 3.03–2.92 (1H, m), 2.34–2.22 (1H, m), 1.99–1.87 (1H, m), 1.56 (3H, s), 1.47 (3H, s); $^{13}$C NMR (101 MHz, $CDCl_3$) δ 174.8, 134.2, 131.0, 128.6, 128.0, 124.1, 79.7, 38.2, 35.7, 25.5, 24.8, 24.3; HRMS (APCI): Found MH$^+$ 213.1385 $C_{14}H_{17}N_2$ requires 213.1386.

[7] **5k**was purified by column chromatography on silica gel eluting with $CH_2Cl_2$→$CH_2Cl_2$:MeOH 99.9:0.1 as the acid-base wash did not remove completely the excess dimethyl 2-ethylidenemalonate.

[8] **5l** was purified by column chromatography on silica gel eluting with $CH_2Cl_2$. In this case the acid-base wash was not performed in order to avoid any potential formation of toxic HCN.

**(*E*)-2-(2-Methyl-4-phenylbut-3-en-2-yl)-5-phenyl-3,4-dihydro-2H-pyrrole (5m)**

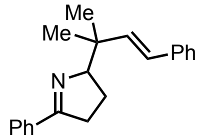

Following **GP15**, **3a** (29 mg, 0.1 mmol) gave **5m** (25 mg, 83%). FT-IR $\nu_{max}$ (film)/cm$^{-1}$ 2961, 1616, 1575, 1494, 1447, 1381, 1360, 1339, 1269, 1060, 1026; $^1$H NMR (400 MHz, $CDCl_3$) δ 7.86–7.81 (2H, m), 7.41–7.35 (3H, m), 7.33–7.28 (2H, m), 7.27–7.21 (2H, m), 7.17–7.12 (1H, m), 6.35 (1H, d, *J* = 16.3 Hz), 6.24 (1H, d, *J* = 16.3 Hz), 4.14–4.06 (1H, m), 2.88–2.82 (2H, m), 2.06–1.94 (1H, m), 1.76–1.66 (1H, m), 1.25 (3H, s), 1.22 (3H, s); $^{13}$C NMR (101 MHz, $CDCl_3$) δ 172.8, 138.1, 138.0, 135.0, 130.4, 128.6, 128.5, 127.8, 127.3, 127.0, 126.3, 82.3, 41.1, 35.5, 25.4, 24.7, 24.5; HRMS (APCI): Found MH$^+$ 290.1900 $C_{21}H_{24}N$ requires 290.1903.

**2-(2-Methyl-4-(triisopropylsilyl)but-3-yn-2-yl)-5-phenyl-3,4-dihydro-2H-pyrrole (5n)**

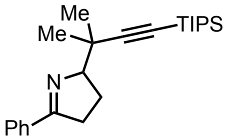

Following **GP16**, **3a** (29 mg, 0.1 mmol) gave **5n** (23 mg, 63%).[9] FT-IR $\nu_{max}$ (film)/cm$^{-1}$ 2941, 2864, 2156, 1619, 1462, 1381, 1360, 1340, 1260, 1060, 1016; $^1$H NMR (500 MHz, $CDCl_3$) δ 7.84 (2H, dd, *J* = 7.7, 1.6 Hz), 7.41–7.35 (3H, m), 4.12–4.07 (1H, m), 3.01 (1H, dddd, *J* = 15.0, 10.2, 4.7, 2.4 Hz), 2.96–2.88 (1H, m), 2.20–2.12 (1H, m), 2.11–2.03 (1H, m), 1.39 (3H, s), 1.37 (3H, s), 0.99–0.95 (18H, m); $^{13}$C NMR (126 MHz, $CDCl_3$) δ 173.0, 135.0, 130.3, 128.3, 127.9, 114.7, 81.9, 80.2, 37.9, 35.7, 28.2, 26.4, 25.3, 18.7, 11.3; HRMS (EI): Found MH$^+$ 368.2770 $C_{24}H_{38}NSi$ requires 368.2768.

**2-Methyl-5-phenyl-3,4-dihydro-2H-pyrrole (6a)**

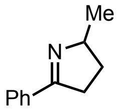

Following **GP4**, **3b** (26 mg, 0.10 mmol) gave **6a** (11 mg, 68%). $^1$H NMR (400 MHz, $CDCl_3$) δ 7.84 (1H, dd, *J* = 7.3, 2.0 Hz), 7.45–7.36 (2H, m), 4.29 (1H, q, *J* = 6.9 Hz), 3.06 (1H, dddd,

[9] **5n** was purified by column chromatography on silica gel eluting with $CH_2Cl_2$. **5n**-H$^+$ was too apolar to remain in the aqueous layer making the acid-base wash ineffective.

*J* = 14.5, 9.8, 4.7, 2.1 Hz), 2.95–2.81 (1H, m), 2.30–2.19 (1H, m), 1.60–1.47 (1H, m), 1.36 (1H, d, *J* = 6.8 Hz). Data in accordance with the literature.[19]

**2-(Chloromethyl)-5-phenyl-3,4-dihydro-2H-pyrrole (6b)**

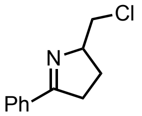


Following **GP5**, **3b** (39 mg, 0.1 mmol) gave **6b** (21 mg, 87%). $^1$H NMR (400 MHz, $CDCl_3$) δ 7.84 (1H, dd, *J* = 7.3, 2.0 Hz), 7.45–7.36 (2H, m), 4.29 (1H, q, *J* = 6.9 Hz), 3.06 (1H, dddd, *J* = 14.5, 9.8, 4.7, 2.1 Hz), 2.95–2.81 (1H, m), 2.30–2.19 (1H, m), 1.60–1.47 (1H, m), 1.36 (1H, d, *J* = 6.8 Hz). $^{13}$C NMR (101 MHz, $CDCl_3$) δ 174.2, 134.3, 130.9, 128.6, 128.0, 73.0, 35.8, 29.2, 14.2. Data in accordance with the literature.[20]

**2-(Bromomethyl)-5-phenyl-3,4-dihydro-2H-pyrrole (6c)**

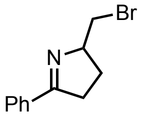


Following **GP6** but with 1 equiv. $Cs_2CO_3$ in 0.1 M MeOH, **3b** (13 mg, 0.05 mmol) gave **6c** (7.5 mg, 63%). $^1$H NMR (500 MHz, $CDCl_3$) δ 7.87–7.83 (2H, m), 7.47–7.38 (3H, m), 4.63–4.56 (1H, m), 3.79 (1H, dd, *J* = 10.1, 4.0 Hz), 3.64 (1H, dd, *J* = 10.1, 6.4 Hz), 3.11 (1H, dddd, *J* = 17.4, 10.2, 5.1, 2.3 Hz), 2.95 (1H, dddd, *J* = 17.1, 10.1, 7.0, 1.4 Hz), 2.28 (1H, dddd, *J* = 13.1, 10.2, 8.2, 5.1 Hz), 1.92 (1H, ddt, *J* = 13.1, 10.2, 6.6 Hz). $^{13}$C NMR (126 MHz, $CDCl_3$) δ 174.7, 134.3, 130.9, 128.6, 128.0, 73.3, 38.4, 35.8, 27.4. Data in accordance with the literature.[21]

**Diethyl 1-((5-Phenyl-3,4-dihydro-2H-pyrrol-2-yl)methyl)hydrazine-1,2-dicarboxylate (6d)**

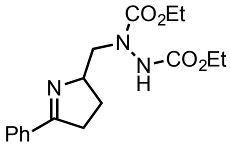


Following **GP10**, **3b** (26 mg, 0.1 mmol) gave **6d** (21 mg 65%).[10] FT-IR $\nu_{max}$ (film)/cm$^{-1}$ 3286, 2980, 1708, 1615, 1447, 1414, 1382, 1258, 1217, 1172, 1127, 1095, 1057, 1024; $^1$H NMR (500 MHz, toluene-$d_8$, T = 100 ºC)[5] δ 7.78 (2H, d, *J* = 4.3 Hz), 7.15–7.10 (3H, m),

[10] **6d** was purified by column chromatography on silica gel eluting with $CH_2Cl_2$→ $CH_2Cl_2$:MeOH 99.5:0.5.

4.43–4.34 (1H, m), 4.05 (2H, q, $J$ = 6.9 Hz), 3.97 (2H, q, $J$ = 6.9 Hz), 3.87 (1H, d, $J$ = 11.9 Hz), 3.57 (1H, dd, $J$ = 12.6, 7.3 Hz), 2.62–2.54 (1H, m), 2.47–2.37 (1H, m), 1.85–1.76 (1H, m), 1.46–1.38 (1H, m), 1.06 (3H, t, $J$ = 7.0 Hz), 1.00 (3H, t, $J$ = 6.9 Hz); $^{13}$C NMR (125 MHz, toluene-$d_9$, T = 100 ºC)[5] δ 174.3, 156.8, 156.3, 131.3, 128.7, 128.4, 71.8, 62.3, 61.6, 55.2, 34.9, 26.3, 14.7, 14.6; HRMS (ESI): Found MH$^+$ 334.1748 $C_{17}H_{24}O_4N_3$ requires 334.1761.

**5-Phenyl-2-((phenylselanyl)methyl)-3,4-dihydro-2H-pyrrole (6e)**

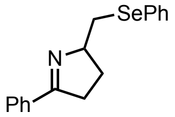


Following **GP12** but in 0.1 M $CH_2Cl_2$, **3b** (39 mg, 0.15 mmol) gave **6e** (32 mg, 74%). $^1$H NMR (400 MHz, $CDCl_3$) δ 7.80 (2H, d, $J$ = 7.3 Hz), 7.56 (2H, d, $J$ = 7.8 Hz), 7.44–7.36 (3H, m), 7.28–7.21 (3H, m), 4.55–4.48 (1H, m), 3.47 (1H, dd, $J$ = 12.1, 4.7 Hz), 3.08 (2H, dd, $J$ = 12.0, 8.0 Hz), 2.98–2.86 (1H, m), 2.34–2.24 (1H, m), 1.86–1.75 (1H, m). GCMS (EI): 315 (M), 238, 220, 144. Data in accordance with the literature.[22]

**Benzyl 2-Methyl-4-(5-phenyl-3,4-dihydro-2*H*-pyrrol-2-yl)butanoate (6f)**

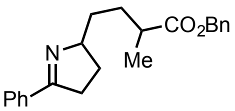


Following **GP13** but using benzyl methacrylate, **3b** (39 mg, 0.15 mmol) gave **6f** (31 mg, 61%). d.r 1.5:1. FT-IR $\nu_{max}$ (film)/cm$^{-1}$2938, 1732, 1615, 1575, 1496, 1455, 1384, 1337, 1258, 1212, 1149; $^1$H NMR (500 MHz, toluene-$d_8$, 100 ˚C) δ 7.80 (2H, br s), 7.22 – 7.06 (7H, m), 7.06 – 7.01 (1H, m), 5.05 – 4.95 (2H, m), 4.06 – 3.92 (1H, m), 2.70 – 2.41 (3.2H, m), 1.93 – 1.80 (1.6H, m), 1.79 – 1.49 (3H, m), 1.37 – 1.26 (1.4H, m), 1.21 (1.2H, d, $J$ = 6.1 Hz, 1H), 1.14 (1.8H, d, $J$ = 6.6 Hz); HRMS (ASAP): Found MH$^+$ 336.1945 $C_{22}H_{26}O_2N$ requires 336.1958.

**2-Cinnamyl-5-phenyl-3,4-dihydro-2H-pyrrole (6g)**

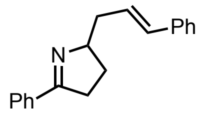

Following **GP15**, **3b** (26 mg, 0.1 mmol) gave **6g** (13 mg, 51%).[11] FT-IR $\nu_{max}$ (film)/cm$^{-1}$ 2920, 1610, 1574, 1493, 1447, 1339, 1246, 1074, 1051, 1024; $^{1}$H NMR (500 MHz, $CDCl_3$) δ 7.86 (2H, d, *J* = 7.6), 7.44–7.39 (3H, m), 7.36 (2H, d, *J* = 7.4 Hz), 7.29 (2H, t, *J* = 7.6 Hz), 7.20 (1H, t, *J* = 7.3 Hz), 6.49 (1H, d, *J* = 15.8 Hz), 6.29 (1H, dt, *J* = 15.7, 7.2 Hz), 4.41–4.34 (1H, m), 3.02 (1H, dddd, *J* = 17.1, 9.9, 5.0, 2.1 Hz), 2.95–2.86 (1H, m), 2.82–2.75 (1H, m), 2.51–2.43 (1H, m), 2.20 (1H, m), 1.79–1.69 (1H, m); $^{13}$C NMR (126 MHz, $CDCl_3$) δ 172.7, 137.8, 134.8, 132.1, 130.5, 128.6, 128.6, 127.9, 127.7, 127.2, 126.2, 73.1, 40.1, 35.2, 28.0; HRMS (ASAP): Found MH$^+$ 262.1582 $C_{19}H_{20}N$ requires 262.1590.

**5-Phenyl-2-(3-phenylprop-2-yn-1-yl)-3,4-dihydro-2H-pyrrole (6h)**

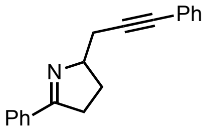

Following **GP16** but with 3.0 equiv. 1-[(phenyl]-1,2-benziodoxol-3(1H)-one in 0.05 M $CH_2Cl_2$, **3b** (26 mg, 0.1 mmol) gave **6h** (15 mg, 58%). FT-IR $\nu_{max}$ (film)/cm$^{-1}$ 2965, 1613, 1574, 1488, 1447, 1345, 1264, 1053, 1023; $^{1}$H NMR (500 MHz, $CDCl_3$) δ 7.86 (2H, dd, *J* = 7.7, 1.4 Hz), 7.45–7.39 (3H, m), 7.38–7.33 (2H, m), 7.27–7.23 (3H, m), 4.54–4.47 (1H, m), 3.13 (1H, dddd, *J* = 15.3, 9.9, 5.2, 2.2 Hz), 3.03–2.91 (2H, m), 2.70 (1H, dd, *J* = 16.7, 7.7 Hz), 2.33 (1H, dddd, *J* = 13.1, 10.0, 8.0, 5.3 Hz), 1.99 (1H, ddt, *J* = 12.9, 10.0, 6.5 Hz); $^{13}$C NMR (126 MHz, $CDCl_3$) δ 173.7, 134.5, 131.7, 130.7, 128.6, 128.3, 128.0, 127.8, 123.9, 87.6, 81.8, 71.9, 35.5, 28.0, 26.9; HRMS (APCI): Found MH$^+$ 260.1429 $C_{19}H_{18}N$ requires 260.1425.

---

[11] After acid-base wash, **5h** was purified by column chromatography on silica gel eluting with $CH_2Cl_2 \rightarrow CH_2Cl_2$:MeOH 99.5:0.5.

**3-(2-(Chloromethyl)-3,4-dihydro-2H-pyrrol-5-yl)pyridine (6i)**

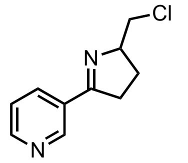


Following **GP5**, **3c** (27 mg, 0.1 mmol) gave **6i** (12 mg, 64%).[12] FT-IR $\nu_{max}$ (film)/cm$^{-1}$ 2923, 1660, 1620, 1586, 1568, 1468, 1436 1348, 1287, 1260, 1152, 1045; $^1$H NMR (500 MHz, $CDCl_3$) δ 8.66 (1H, d, *J* = 4.8 Hz), 8.13 (1H, d, *J* = 7.9 Hz), 7.75 (1H, td, *J* = 7.7, 1.6 Hz), 7.34 (1H, dd, *J* = 7.2, 5.0 Hz), 4.67–4.59 (1H, m), 3.90 (1H, dd, *J* = 10.9, 4.3 Hz), 3.80 (1H, dd, *J* = 10.9, 5.9 Hz), 3.28 (1H, dddd, *J* = 17.8, 10.2, 5.0, 2.5 Hz), 3.14–3.06 (1H, m), 2.27 (1H, dddd, *J* = 13.2, 10.2, 8.3, 5.0 Hz), 1.95 (1H, ddt, *J* = 13.3, 10.2, 6.7 Hz); $^{13}$C NMR (126 MHz, $CDCl_3$) δ 176.5, 152.9, 149.4, 136.6, 125.1, 122.5, 74.2, 48.7, 35.7, 26.1; HRMS (APCI): Found MH$^+$ 195.0679 $C_{10}H_{12}N_2Cl$ requires 195.0684.

**Diethyl 1-((5-(Pyridin-3-yl)-3,4-dihydro-2H-pyrrol-2-yl)methyl)hydrazine-1,2-dicarboxylate (6j)**

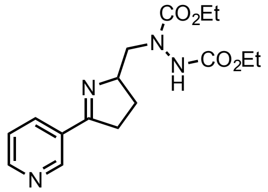


Following **GP10**, **3c** (26 mg, 0.1 mmol) gave **6j** (20 mg, 60%).[13] FT-IR $\nu_{max}$ (film)/cm$^{-1}$ 3292, 2981, 1708, 1513, 1468, 1439, 1413, 1382, 1257, 1216, 1172, 1057; $^1$H NMR (500 MHz, toluene-$d_8$, T = 100 ºC)[5] δ 8.37 (1H, d, *J* = 3.8 Hz), 8.06 (1H, d, *J* = 7.8 Hz), 7.18 (1H, t, *J* = 7.7 Hz), 6.78–6.73 (1H, m), 4.44–4.35 (1H, m), 4.04 (2H, q, *J* = 7.1 Hz), 3.99 (2H, q, *J* = 7.5 Hz), 3.83 (1H, dd, *J* = 13.9, 6.0 Hz), 3.58 (1H, dd, *J* = 13.9, 7.5 Hz), 3.16–3.08 (1H, m), 2.91–2.82 (1H, m), 1.90–1.82 (1H, m), 1.51–1.41 (1H, m), 1.07–1.03 (3H, m), 1.03–0.99 (3H, m); $^{13}$C NMR (500 MHz, toluene-$d_9$, T = 100 ºC)[5] δ 175.7, 156.8, 156.3, 154.6, 149.4, 135.8, 124.4, 122.2, 73.6, 62.3, 61.7, 55.7, 35.3, 27.0, 14.7, 14.6; HRMS (ESI): Found MH$^+$ 335.1707 $C_{16}H_{23}O_4N_4$ requires 335.1714.

[12] After acid-base wash, **6i** was purified by column chromatography on silica gel eluting with $CH_2Cl_2$→ $CH_2Cl_2$:MeOH 99.6:0.4.
[13] **6k** was purified by column chromatography on silica gel eluting with $CH_2Cl_2$→ $CH_2Cl_2$:MeOH 98:2.

**3-(2-Cinnamyl-3,4-dihydro-2H-pyrrol-5-yl)pyridine (6k)**

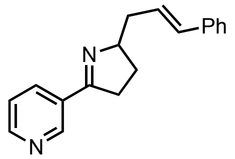


Following **GP15**, **3c** (26 mg, 0.1 mmol) gave **6k** (11 mg, 41%).[14] FT-IR $\nu_{max}$ (film)/cm$^{-1}$ 2922, 1698, 1619, 1586, 1565, 1495, 1468, 1438, 1340, 1259, 1042; $^{1}$H NMR (400 MHz, $CDCl_3$) δ 8.58 (1H, d, *J* = 4.2 Hz), 8.07 (1H, d, *J* = 7.9 Hz), 7.68 (1H, t, *J* = 7.6 Hz), 7.32–7.18 (5H, m), 7.13 (1H, t, *J* = 7.2 Hz), 6.43 (1H, d, *J* = 15.9 Hz), 6.28–6.19 (1H, m), 4.39–4.29 (1H, m), 3.19–3.08 (1H, m), 3.04–2.90 (1H, m), 2.78–2.69 (1H, m), 2.45–2.36 (1H, m), 2.20–2.09 (1H, m), 1.68 (1H, tt, *J* = 15.9, 8.0 Hz); $^{13}$C NMR (101 MHz, $CDCl_3$) δ 174.4, 153.4, 149.3, 137.8, 136.5, 132.2, 128.6, 127.5, 127.2, 126.2, 124.8, 122.3, 73.7, 40.0, 35.1, 28.0; HRMS (APCI): Found MH$^+$ 263.1538 $C_{18}H_{19}N_2$ requires 263.1543.

**2-Methyl-5-phenyl-3,4-dihydro-2H-pyrrole (7a)**

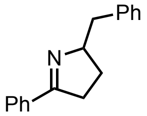


Following **GP4**, **3d** (34 mg, 0.10 mmol) gave **7a** (15 mg, 66%). $^{1}$H NMR (400 MHz, $CDCl_3$) δ 7.77 (2H, d, *J* = 7.0 Hz), 7.39–7.31 (3H, m), 7.23–7.18 (4H, m), 7.16–7.11 (1H, m), 4.48–4.36 (1H, m), 3.23 (1H, dd, *J* = 13.5, 4.8 Hz), 2.78 (2H, t, *J* = 8.1 Hz), 2.64 (1H, dd, *J* = 13.4, 8.8 Hz), 1.99 (1H, td, *J* = 14.6, 7.6 Hz), 1.63 (1H, dt, *J* = 14.5, 7.8 Hz). $^{13}$C NMR (101 MHz, $CDCl_3$) δ 172.8, 139.6, 134.7, 130.5, 129.6, 128.5, 128.4, 127.8, 126.2, 74.4, 42.6, 35.0, 27.9. GCMS (EI): 235 (M), 207, 144, 117. Data in accordance with the literature.[19]

**2-(Bromo(phenyl)methyl)-5-phenyl-3,4-dihydro-2H-pyrrole (7b)**

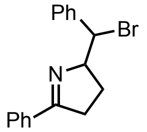


Following **GP6** but using 3 equiv. $BrCCl_3$ as the brominating agent in 0.05 M $CH_2Cl_2$, **3d** (34 mg, 0.1 mmol) gave **7b** (11 mg, 35%). d.r 2:1. FT-IR $\nu_{max}$ (film)/cm$^{-1}$ 2923, 1631, 1589, 1453, 1394, 1362, 1301, 1108; $^{1}$H NMR (400 MHz, $CDCl_3$) δ 7.88 (0.7H, dd, *J* = 7.8, 1.6 Hz), 7.84 (1.3H, dd, *J* = 8.0, 1.5 Hz), 7.60–7.56 (0.7H, m), 7.54–7.49 (1.3H, m), 7.48–7.27

[14] After acid-base wash, **6k** was purified by column chromatography on silica gel eluting with $CH_2Cl_2$→ $CH_2Cl_2$:MeOH 99.5:0.5.

(6H, m), 5.49 (0.7H, d, $J$ = 4.9 Hz), 5.10 (0.3H, d, $J$ = 6.1 Hz), 4.88–4.81 (0.3H, m), 4.79–4.72 (0.7H, m), 3.07 (0.7H, dddd, $J$ = 17.2, 9.5, 5.5, 2.2 Hz), 2.97 (0.6H, ddd, $J$ = 9.4, 7.7, 1.7 Hz), 2.90 (0.7H, ddd, $J$ = 9.5, 5.5, 1.8 Hz), 2.26–2.08 (2H, m), 1.81 (0.7H, dtd, $J$ = 13.3, 8.5, 6.7 Hz); $^{13}$C NMR (126 MHz, $CDCl_3$)[15] δ 174.5$^{M}$ & 174.5$^{m}$, 140.3$^{M}$ & 140.1$^{m}$, 134.4$^{m}$ & 134.3$^{M}$, 130.9$^{M}$ & 130.9$^{m}$, 128.77, 128.63, 128.58, 128.55, 128.52, 128.43, 128.41, 128.30, 128.06, 128.04, 78.7$^{M}$ & 78.6$^{m}$, 60.7$^{M}$ & 59.3$^{m}$, 35.8$^{M}$ & 35.6$^{m}$, 27.4$^{m}$ & 26.4$^{M}$; HRMS (ASAP): Found $MH^+$ 314.0537 $C_{17}H_{17}NBr$ requires 314.0539.

**2-(Fluoro(phenyl)methyl)-5-phenyl-3,4-dihydro-2H-pyrrole (7c)**

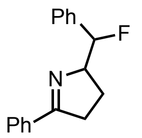


Following **GP8** but in 0.05 M HFIP, **3d** (34 mg, 0.1 mmol) gave **7c** (14.5 mg, 38%).[16] d.r 9:1. FT-IR $\nu_{max}$ (film)/cm$^{-1}$ 2919, 1613, 1575, 1537, 1495, 1448, 1344, 1285, 1255, 1192, 1186, 1018; $^{1}$H NMR (400 MHz, $CDCl_3$, major diastereomer) δ 7.77 (2H, dd, $J$ = 8.0, 1.5 Hz), 7.45–7.31 (8H, m), 5.60 (1H, dd, $J$ = 46.2, 5.8 Hz), 4.81–4.69 (1H, m), 2.89–2.79 (1H, m), 2.77–2.66 (1H, m), 2.04–1.94 (1H, m), 1.87–1.75 (m, 1H); $^{13}$C NMR (101 MHz, $CDCl_3$, major diastereomer) δ 175.2, 137.6 (d, $J$ = 20.1 Hz), 134.4, 130.8, 128.5, 128.5 (d, $J$ = 1.8 Hz), 128.3, 128.0, 126.7 (d, $J$ = 6.9 Hz), 95.9 (d, $J$ = 176.2 Hz), 76.8 (d, $J$ = 23.4 Hz), 35.2, 24.5 (d, $J$ = 3.4 Hz); $^{19}$F NMR (376 MHz, $CDCl_3$) δ –180.5$^{M}$, 196.8$^{m}$ ; HRMS (EI): Found $MH^+$ 254.1337 $C_{17}H_{17}F$ requires 254.1340.

**Diethyl 1-(Phenyl(5-phenyl-3,4-dihydro-2H-pyrrol-2-yl)methyl)hydrazine-1,2-dicarboxylate (7d)**

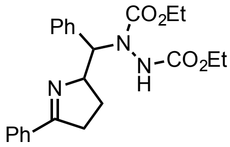


Following **GP10**, **3d** (34 mg, 0.1 mmol) gave **7d** (25 mg, 62%).[17,18] FT-IR $\nu_{max}$ (film)/cm$^{-1}$ 3286, 2980, 1703, 1614, 1575, 1448, 1408, 1379, 1330, 1300, 1217, 1173, 1096, 1059; $^{1}$H NMR (500 MHz, $C_6D_6$, T = 75 ºC)[5] δ 7.70 (2H, d, $J$ = 7.2 Hz), 7.56 (2H, d, $J$ = 7.4 Hz), 7.15–7.04 (6H, m), 5.49 (1H, d, $J$ = 7.0 Hz), 4.95 (1H, d, $J$ = 4.3 Hz), 4.04–3.85 (4H, m), 2.55 (1H,

[15] Not all $^{13}$C NMR peaks could be assigned between the major and the minor diastereomer.
[16] After acid-base wash, **7c** was purified by column chromatography on silica gel eluting with $CH_2Cl_2$.
[17] **7d** was purified by column chromatography on silica gel eluting with $CH_2Cl_2$→$CH_2Cl_2$:MeOH 99.5:0.5.
[18] In this case we have not been able to determine the d.r by any spectroscopic method.

s), 2.49–2.38 (1H, m), 2.23 – 2.13 (1H, br s), 1.90 (1H, br s), 1.03 (3H, t, $J$ = 7.0 Hz), 0.95–0.89 (3H, m); $^{13}$C NMR (126 MHz, $C_6D_6$, T = 75 ºC)[5] δ 173.6, 156.6, 156.4, 135.4, 130.4, 130.3, 128.5, 128.4, 128.4, 128.2, 128.0, 75.1, 66.1, 62.5, 61.6, 34.7, 27.4, 14.6, 14.4; HRMS (ASAP): Found $MH^+$ 410.2063 $C_{23}H_{28}O_4N_3$ requires 410.2074.

**(*E*)-2-(1,3-Diphenylallyl)-5-phenyl-3,4-dihydro-2H-pyrrole (7e)**

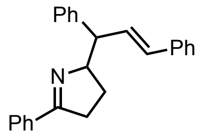


Following **GP15** but with 3.0 equiv. 1-[(phenyl]-1,2-benziodoxol-3(1H)-one, **3d** (34 mg, 0.1 mmol) gave **7e** (16 mg, 48%). d.r 9:1. FT-IR $\nu_{max}$ (film)/cm$^{-1}$ 3025, 2962, 1614, 1575, 1493, 1448, 1339, 1260, 1073, 1027; $^1$H NMR (500 MHz, $CDCl_3$, major diastereomer) δ 7.86 (2H, dd, $J$ = 7.6, 1.7 Hz), 7.44–7.36 (7H, m), 7.30 (4H, dt, $J$ = 16.8, 6.3 Hz), 7.25–7.17 (2H, m), 6.72 (1H, dd, $J$ = 15.9, 7.7 Hz), 6.44 (1H, d, $J$ = 15.9 Hz), 4.70 (1H, app q, $J$ = 6.8 Hz), 3.66 (1H, t, $J$ = 7.3 Hz), 2.87–2.77 (1H, m), 2.73 (1H, dddd, $J$ = 16.9, 9.8, 5.0, 2.2 Hz), 2.12–2.02 (1H, m), 1.78–1.69 (1H, m); $^{13}$C NMR (126 MHz, $CDCl_3$, major diastereomer) δ 173.2, 142.4, 137.8, 134.8, 131.9, 131.3, 130.5, 129.0, 128.6, 128.5, 128.5, 127.9, 127.2, 126.6, 126.5, 77.2, 55.0, 34.9, 27.3; HRMS (ESI): Found $MH^+$ 338.1897 $C_{25}H_{24}N$ requires 338.1903.

**Methyl 2-(5-phenyl-3,4-dihydro-2H-pyrrol-2-yl)acetate (7f)**

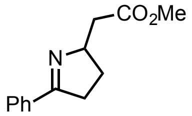


Following **GP4**, **3e** (32 mg, 0.1 mmol) gave **7f** (18 mg, 82%).[19] FT-IR $\nu_{max}$ (film)/cm$^{-1}$ 2950, 1733, 1614, 1575, 1493, 1435, 1337, 1247, 1172, 1060, 1026; $^1$H NMR (500 MHz, $CDCl_3$) δ 7.85–7.81 (2H, m), 7.44–7.37 (3H, m), 4.63–4.56 (1H, m), 3.72 (3H, s), 3.07 (1H, dddd, $J$ = 16.8, 10.0, 4.6, 2.1 Hz), 2.97–2.90 (2H, m), 2.47 (1H, dd, $J$ = 15.5, 8.9 Hz), 2.34 (1H, dddd, $J$ = 12.7, 9.8, 7.8, 4.7 Hz), 1.69 (1H, ddt, $J$ = 13.2, 10.0, 7.3 Hz); $^{13}$C NMR (126 MHz, $CDCl_3$) δ 173.3, 172.5, 134.4, 130.7, 128.5, 127.9, 69.6, 51.8, 41.1, 35.3, 28.8; HRMS (APCI): Found $MH^+$ 219.1177 $C_{13}H_{16}O_2N$ requires 218.1176.

[19] **7f** was not stable upon acid-base wash. As a result this compound was purified by direct column chromatography on silica gel eluting with $CH_2Cl_2 \rightarrow CH_2Cl_2$:MeOH 99.6:0.4.

**Methyl 2-Bromo-2-(5-phenyl-3,4-dihydro-2H-pyrrol-2-yl)acetate (7g)**

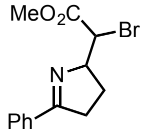


Following **GP6** but using 4 equiv. $BrCCl_3$ as the brominating agent in 0.1 M $CH_2Cl_2$ and irradiating for 40 h, **3e** (32 mg, 0.1 mmol) gave **7f** (13 mg, 44%). d.r 2.3:1. FT-IR $\nu_{max}$ (film)/cm$^{-1}$ 2951, 1739, 1614, 1575, 1447, 1435, 1339, 1266, 1201, 1151; $^1$H NMR (400 MHz, $CDCl_3$) δ 7.87–7.81 (2H, m), 7.48–7.37 (3H, m), 4.82–4.73 (1H, m), 4.56 (0.7H, d, *J* = 6.5 Hz), 4.52 (0.3H, d, *J* = 6.5 Hz), 3.85 (2.1H, s), 3.79 (0.9H, s), 3.19–3.05 (1H, m), 3.05–2.89 (1H, m), 2.38–2.25 (1H, m), 2.10–1.99 (1H, m); $^{13}$C NMR (101 MHz, $CDCl_3$) δ 175.4 , 169.6$^M$ & 169.1$^m$, 134.1 131.1$^M$ & 131.0$^m$ 128.65$^M$ & 128.6$^m$, 128.2$^m$ & 128.1$^M$, 75.3$^m$ & 74.7$^M$, 53.2$^M$ & 53.2$^m$, 51.1$^M$ & 49.8$^m$, 36.0$^m$ & 35.8$^M$, 26.4$^M$ & 26.3$^m$; HRMS (ASAP): Found MH$^+$ 296.0277 $C_{13}H_{15}O_2NBr$ requires 296.0281.

**Diethyl 1-(2-Methoxy-2-oxo-1-(5-phenyl-3,4-dihydro-2H-pyrrol-2-yl)ethyl)hydrazine-1,2-dicarboxylate (7h)**

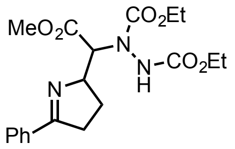


Following **GP10**, **3e** (32 mg, 0.1 mmol) gave **7h** (61%).[20] d.r 2:1. FT-IR $\nu_{max}$ (film)/cm$^{-1}$ 3303, 2980, 1712, 1617, 1409, 1381, 1294, 1212, 1175, 1061, 1025; $^1$H NMR (500 MHz, toluene-$d_8$, T = 100 ºC)[5] δ 7.75–7.67 (2H, m), 7.14–7.07 (3H, m), 5.24 (0.6H, s), 5.03 (0.3H, d, *J* = 5.8 Hz), 4.87–4.77 (1H, m), 4.08–3.53 (4H, m), 3.48 (2H, s), 3.39 (0.9H, s), 2.96–2.87 (0.6H, m), 2.71–2.62 (0.3H, m), 2.51–2.39 (1H, m), 2.00–1.91 (1H, m), 1.04 (1.8H, t, *J* = 7.1 Hz), 1.00 (2.4H, t, *J* = 6.9 Hz), 0.83 (2H, t, *J* = 7.1 Hz); $^{13}$C NMR (500 MHz, toluene-$d_9$, T = 100 ºC)[5] δ 173.9$^m$ & 173.3$^M$, 171.0 , 157.4$^m$ & 156.7$^M$, 156.3$^m$ & 155.7$^M$, 135.6$^M$ & 135.4$^m$, 130.5$^m$ & 130.3$^M$, 129.3 (under the toluene-$d_9$ peaks), 128.5$^m$ & 128.3$^M$, 73.6$^M$ & 73.4$^m$, 65.2$^m$ & 64.2$^M$, 62.9 , 61.7$^m$ & 61.3$^M$, 51.9$^M$ & 51.4$^m$, 35.7$^M$ & 34.9$^m$, 27.6$^m$ & 25.4$^M$ & 14.6$^m$, 14.5$^m$, 14.5$^M$, 14.3$^M$; HRMS (ESI): Found MH$^+$ 392.1805 $C_{19}H_{26}O_6N_3$ requires 392.1816.

[20] In this case the yield is based on internal $^1$H NMR standard as we were not able to fully purify **7g** from excess DEAD. We have been able to obtain a clean fraction (10 mg, 26%) which was isolated by column chromatography on silica gel eluting with $CH_2Cl_2$→$CH_2Cl_2$:MeOH 98:2.

**Methyl 2-(5-phenyl-3,4-dihydro-2H-pyrrol-2-yl)-2-(phenylselanyl)acetate (7i)**

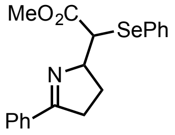


Following **GP12**, **3e** (32 mg, 0.1 mmol) gave **7i** (39 mg, 78%). d.r 1:1. FT-IR $\nu_{max}$ (film)/cm$^{-1}$ 2948, 1727, 1613, 1575, 1476, 1447, 1436, 1338, 1258, 1193, 1142, 1021 $^1$H NMR (400 MHz, $CDCl_3$, diastereomers) δ 7.78 (1H, d, *J* = 7.1 Hz), 7.72 (1H, d, *J* = 7.3 Hz), 7.61 (1H, d, *J* = 7.0 Hz), 7.56 (1H, d, *J* = 7.1 Hz), 7.38–7.27 (3H, m), 7.25–7.17 (3H, m), 4.68–4.55 (1H, m), 3.90 (0.5H, d, *J* = 8.2 Hz), 3.70 (0.5H, d, *J* = 8.3 Hz), 3.65 (1.5H, s), 3.45 (1.5H, s), 3.10–2.94 (1H, m), 2.88 (1H, td, *J* = 17.2, 8.3 Hz), 2.46–2.37 (0.5H, m), 2.37–2.28 (0.5H, m), 1.94–1.80 (1H, m); $^{13}$C NMR (101 MHz, $CDCl_3$, diastereomers) δ 174.2 & 174.1, 172.9 & 171.9, 135.8 & 135.4, 134.3, 130.8 & 130.8, 129.2 & 129.1, 128.79, 128.52, 128.50, 128.47, 128.45, 128.42, 128.1 & 128.0, 74.3 & 73.9, 52.3 & 52.1, 50.6 & 49.5, 35.7 & 35.6, 27.7 & 27.4; HRMS (EI): Found $MH^+$ 374.0647 $C_{19}H_{20}O_2NSe$ requires 374.0654.

**Methyl (*E*)-4-phenyl-2-(5-phenyl-3,4-dihydro-2H-pyrrol-2-yl)but-3-enoate (7j)**

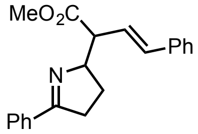


Following **GP15**, **3e** (32 mg, 0.1 mmol) gave **7l** (20 mg 63%).[21] d.r 9:1. FT-IR $\nu_{max}$ (film)/cm$^{-1}$ 2949, 1768, 1730, 1614, 1575, 1495, 1448, 1433, 1340, 1259, 1157, 1027; $^1$H NMR (500 MHz, $CDCl_3$, major diastereomer) δ 7.84 (2H, dd, *J* = 8.0, 1.4 Hz), 7.44–7.37 (5H, m), 7.30 (2H, t, *J* = 7.6 Hz), 7.25–7.20 (1H, m), 6.54 (1H, d, *J* = 16.0 Hz), 6.37 (1H, dd, *J* = 16.0, 9.0 Hz), 4.75–4.69 (1H, m), 3.74 (3H, s), 3.47 (1H, dd, *J* = 8.8, 6.8 Hz), 3.04–2.88 (2H, m), 2.23 (1H, dddd, *J* = 13.1, 9.6, 8.2, 5.1 Hz), 1.96–1.86 (1H, m); $^{13}$C NMR (126 MHz, $CDCl_3$, major diastereomer) δ 173.8, 173.2, 137.0, 134.5, 133.7, 130.7, 128.6, 128.5, 128.0, 127.7, 126.6, 125.2, 75.0, 55.0, 52.1, 35.3, 26.4; HRMS (ASAP): Found $MH^+$ 318.1491 $C_{21}H_{20}O_2N$ requires 318.1489.

[21] **7i** was purified by column chromatography on silica gel eluting with $CH_2Cl_2 \rightarrow CH_2Cl_2$:MeOH 99.5:0.5.

**7-Chloro-2-phenyl-3a,4,5,6,7,7a-hexahydro-3H-indole (7k)**

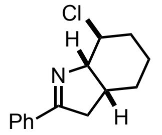

Following **GP5**, **3f** (45 mg, 0.15 mmol) gave **7k** (26 mg, 75%). dr 20:1. FT-IR $\nu_{max}$ (film)/cm$^{-1}$ 2937, 2864, 1603, 1573, 1495, 1447, 1336, 1254, 1233, 1214, 1195, 1052, 1015; $^{1}$H NMR (400 MHz, $CDCl_3$) δ 7.84 (2H, d, *J* = 7.4 Hz), 7.46–7.39 (3H, m), 4.69 (1H, app q, *J* = 4.0 Hz), 4.12 (1H, br s), 2.92 (1H, dd, *J* = 16.1, 6.4 Hz), 2.76–2.65 (2H, m), 1.94–1.87 (2H, m), 1.83–1.74 (1H, m), 1.71–1.62 (1H, m), 1.45–1.36 (1H, m), 1.23–1.12 (1H, m); $^{13}$C NMR (101 MHz, $CDCl_3$) δ 174.8, 134.8, 130.9, 128.6, 127.7, 76.6, 60.6, 42.3, 35.0, 30.8, 27.0, 18.4; HRMS (ESI): Found M$^+$ 234.1046 $C_{14}H_{17}NCl$ requires 234.1044.

**7-Fluoro-2-phenyl-3a,4,5,6,7,7a-hexahydro-3H-indole (7l)**

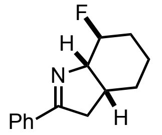

Following **GP8**, **3f** (30 mg, 0.1 mmol) gave **7l** (12 mg, 53%). d.r 4:1. FT-IR $\nu_{max}$ (film)/cm$^{-1}$ 2936, 2864, 1683, 1604, 1573, 1538, 1495, 1447, 1430, 1338, 1253, 1056, 1020; $^{1}$H NMR (400 MHz, $CDCl_3$) δ 7.88 (0.4H, dd, *J* = 7.7, 1.8 Hz), 7.84 (1.6H, d, *J* = 7.0 Hz), 7.48–7.36 (3H, m), 5.23 (0.8H, br d, *J* = 50.4 Hz), 5.07–4.90 (0.2H, m), 4.23–4.05 (1H, m), 2.96 (0.8H, dd, *J* = 16.2, 6.8 Hz), 2.92–2.86 (0.2H, m), 2.80–2.71 (1H, m), 2.64 (0.8H, td, *J* = 13.2, 6.8 Hz), 2.58–2.50 (0.2H, m), 2.01–1.86 (1H, m), 1.73–1.54 (3.2H m), 1.43–1.32 (1H, m), 1.11 (0.8H, m); $^{13}$C NMR (126 MHz, $CDCl_3$) δ 176.1$^{m}$ & 174.8$^{M}$ (d, *J* = 1.9 Hz), 134.9$^{m}$ & 134.8$^{M}$ (d, *J* = 1.3 Hz), 130.8$^{M}$ & 130.7$^{m}$, 128.6$^{M}$ & 128.5$^{m}$, 127.9$^{m}$ & 127.7$^{M}$, 91.3$^{M}$ (d, *J* = 165.4 Hz) & 90.6$^{m}$ (d, *J* = 176.6 Hz), 73.5$^{M}$ (d, *J* = 26.6 Hz) & 72.5$^{m}$ (d, *J* = 16.1 Hz), 42.8$^{M}$ & 41.3$^{m}$ (d, *J* = 1.7 Hz), 37.0$^{m}$ (d, *J* = 2.7 Hz) & 35.3$^{M}$, 28.0$^{m}$ (d, *J* = 20.1 Hz) & 28.0$^{M}$ (d, *J* = 20.7 Hz), 27.2$^{M}$ & 25.9$^{m}$, 18.1$^{m}$ (d, *J* = 6.7 Hz) & 17.6$^{M}$ (d, *J* = 3.8 Hz); $^{19}$F NMR (376 MHz, $CDCl_3$) δ –180.3$^{M}$, –184.1$^{m}$; HRMS (APCI): Found MH$^+$ 218.1333 $C_{14}H_{17}NF$ requires 218.1340.

**7-Azido-2-phenyl-3a,4,5,6,7,7a-hexahydro-3H-indole (7m)**

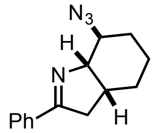

Following **GP9**, **3f** (30 mg, 0.1 mmol) gave **7m** (17 mg, 72%). d.r. 4:1. FT-IR $\nu_{max}$ (film)/cm$^{-1}$ 2932, 2858, 2090, 1603, 1573, 1495, 1447, 1336, 1253, 1016; $^1$H NMR (400 MHz, $CDCl_3$, major diastereomer) δ 7.85 (2H, d, *J* = 7.6 Hz), 7.47–7.37 (3H, m), 3.96 (1H, t, *J* = 5.9 Hz), 3.85 (1H, dd, *J* = 9.7, 6.2 Hz), 2.96 (1H, dd, *J* = 16.2, 7.4 Hz), 2.70 (1H, dd, *J* = 16.3, 5.4 Hz), 2.64–2.53 (1H, m), 1.74–1.55 (4H, m), 1.48–1.31 (2H, m); $^{13}$C NMR (101 MHz, $CDCl_3$) δ 174.8, 134.8, 130.9, 128.6, 127.7, 74.2, 62.1, 41.3, 36.0, 27.3, 26.4, 18.9; HRMS (APCI): Found MH$^+$ 241.1445 $C_{14}H_{17}N_4$ requires 241.1448.

**2-Phenyl-7-((trifluoromethyl)thio)-3a,4,5,6,7,7a-hexahydro-3H-indole (7n)**

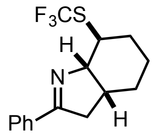

Following **GP11**, **3f** (30 mg, 0.1 mmol) gave **7n** (20 mg, 66%). d.r 8:1 FT-IR $\nu_{max}$ (film)/cm$^{-1}$ 2932, 2857, 1723, 1604, 1574, 1448, 1337, 1259, 1102, 1014; $^1$H NMR (500 MHz, $CDCl_3$, major diastereomer) δ 7.84 (2H, dd, *J* = 8.0, 1.3 Hz), 7.46–7.39 (3H, m), 4.06 (1H, t, *J* = 5.1 Hz), 3.92 (1H, q, *J* = 4.8 Hz), 2.95 (1H, ddd, *J* = 16.2, 6.8, 2.2 Hz), 2.75 (1H, dd, *J* = 16.0, 3.3 Hz), 2.62–2.55 (1H, m), 2.01–1.92 (1H, m), 1.88–1.81 (1H, m), 1.69–1.62 (1H, m), 1.56–1.46 (2H, m), 1.29–1.21 (1H, m); $^{13}$C NMR (126 MHz, $CDCl_3$) δ 174.9$^m$ & 174.9$^M$, 134.8$^M$ & 134.7$^m$, 131.4$^M$ (q, *J* = 306.3 Hz), 131.0$^M$ & 130.9$^m$, 128.7$^M$ & 128.5$^m$, 127.8$^M$ & 127.7$^m$, 74.3$^M$ & 73.2$^m$, 45.8$^m$ (d, *J* = 1.4 Hz) & 45.6$^M$ (d, *J* = 0.8 Hz), 43.9$^m$ & 42.2$^M$, 38.6$^m$ & 35.4$^M$, 31.0$^m$, 28.9$^M$, 27.2$^m$, 26.7$^M$, 24.3$^m$, 19.7$^M$; $^{19}$F NMR (471 MHz, $CDCl_3$) δ –39.5$^M$, –40.6$^m$; HRMS (ESI): Found MH$^+$ 300.1031 $C_{15}H_{17}NF_3S$ requires 300.1028.

**Benzyl 2-Methyl-3-(2-phenyl-3a,4,5,6,7,7a-hexahydro-3*H*-indol-7-yl)propanoate (7o)**

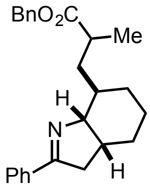


Following **GP13** but using benzyl methacrylate, **3f** (30 mg, 0.1 mmol) gave **7o** (23 mg, 61%).[22] d.r 3:1. FT-IR $\nu_{max}$ (film)/cm$^{-1}$ 2922, 2853, 1730, 1604, 1573, 1496, 1447, 1336, 1261, 1161, 1076, 1023; $^{1}$H NMR (500 MHz, $CDCl_3$ diastereomers) δ 7.78–7.73 (2H, m), 7.36–7.29 (3H, m), 7.26–7.17 (5H, m), 5.03–4.94 (2H, m), 3.63 (1H, m), 2.83–2.76 (1H, m), 2.76–2.71 (1H, m), 2.57–2.52 (0.2H, m), 2.49 (0.8H, ddd, *J* = 16.1, 8.6, 0.9 Hz), 2.42–2.33 (1H, m), 2.20–2.12 (0.8H, m), 1.64–1.55 (1.2H, m), 1.53–1.47 (2H, m), 1.47–1.41 (1H, m), 1.37 (1H, ddd, *J* = 13.7, 8.0, 5.9 Hz), 1.32–1.17 (2H, m), 1.12 (2.2H, d, *J* = 6.9 Hz), 1.09 (0.8H, d, *J* = 7.0 Hz), 1.03–0.95 (1H, m); $^{13}$C NMR (126 MHz, $CDCl_3$, diastereomers) δ 177.3$^{m}$ & 176.9$^{M}$, 173.1 , 136.5$^{m}$ & 136.4$^{M}$, 135.4$^{M}$ & 135.3$^{m}$, 130.4 , 128.6$^{m}$ & 128.6$^{M}$, 128.5 , 128.3$^{M}$ & 128.2$^{m}$, 128.1$^{m}$ & 128.1$^{M}$, 127.7 , 76.1$^{m}$ & 75.7$^{M}$, 66.1 , 39.5$^{m}$ & 39.5$^{M}$, 39.3$^{M}$ & 39.1$^{m}$, 37.9$^{M}$ & 37.9$^{m}$, 38.0$^{m}$ & 37.7$^{M}$, 36.8$^{m}$ & 36.7$^{M}$, 28.4$^{M}$ & 28.3$^{m}$, 26.4$^{m}$ & 26.4$^{M}$, 20.4$^{M}$ & 20.4$^{m}$, 18.1$^{M}$ & 17.7$^{m}$; HRMS (ESI): Found $MH^+$ 376.2257 $C_{25}H_{30}O_2N$ requires 376.2271.

**2-Phenyl-3a,4,5,6,7,7a-hexahydro-3H-indole-7-carbonitrile (7p)**

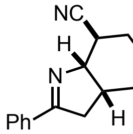


Following **GP14**, **3f** (30 mg, 0.1 mmol) gave **7p** (15 mg, 66%). d.r 4:1. FT-IR $\nu_{max}$ (film)/cm$^{-1}$ 2961, 2857, 1704, 1603, 1571, 1448, 1343, 1259, 1076, 1019; $^{1}$H NMR (400 MHz, $CDCl_3$, major diastereomer) δ 7.90 (2H, dd, *J* = 8.0, 1.4 Hz), 7.48–7.38 (3H, m), 4.14 (1H, t, *J* = 4.8 Hz), 3.08–3.03 (1H, m), 2.97 (1H, ddd, *J* = 16.2, 6.7, 2.4 Hz), 2.86–2.80 (1H, m), 2.49–2.42 (1H, m), 1.94–1.88 (1H, m), 1.77–1.57 (3H, m), 1.35–1.14 (2H, m); $^{13}$C NMR (101 MHz, $CDCl_3$, major diastereomer) δ 175.6, 134.5, 131.0, 128.6, 127.9, 121.6, 69.3, 42.9, 36.6, 31.5, 26.6, 25.2, 21.8; HRMS (ESI): Found $MH^+$ 225.1382 $C_{15}H_{17}N_2$ requires 225.1386.

[22] **7n** was purified by column chromatography on silica gel eluting with $CH_2Cl_2$→$CH_2Cl_2$:MeOH 99.8:0.2.

**(*E*)-2-Phenyl-7-styryl-3a,4,5,6,7,7a-hexahydro-3H-indole (7q)**

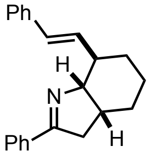

Following **GP15**, **3f** (30 mg, 0.1 mmol) gave **7q** (18 mg, 61%). d.r 2:1. FT-IR $\nu_{max}$ (film)/cm$^{-1}$ 2925, 2851, 1699, 1600, 1573, 1494, 1447, 1339, 1260, 1016; $^1$H NMR (500 MHz, $CDCl_3$, diastereomers) δ 7.91–7.85 (2H, m), 7.46 (1.4H, d, *J* = 7.3 Hz), 7.44–7.38 (3.6H, m), 7.33–7.28 (2H, m), 7.22–7.17 (1H, m), 6.90 (0.7H, dd, *J* = 16.0, 8.2 Hz), 6.55–6.45 (1.3H, m), 4.01–3.92 (1H, m), 2.95 (0.3H, dd, *J* = 16.1, 7.8 Hz), 2.88 (0.7H, ddd, *J* = 15.8, 6.1, 3.1 Hz), 2.79–2.73 (0.3H, m), 2.71 (0.7H, d, *J* = 15.9 Hz), 2.62 (0.7H, ddd, *J* = 16.2, 8.1, 4.0 Hz), 2.59–2.53 (0.3H, m), 2.42 (0.7H, td, *J* = 11.5, 5.9 Hz), 2.38–2.32 (0.3H, m), 1.70 – 1.59 (3H, m), 1.48 – 1.26 (2.3H, m), 0.97 (0.7H, qd, *J* = 12.8, 3.2 Hz); $^{13}$C NMR (126 MHz, $CDCl_3$, diastereomers) δ 174.1$^M$ & 173.4$^m$, 138.3$^M$ & 138.1$^m$, 135.7$^M$ & 135.4$^m$, 135.6$^M$ & 134.3$^m$, 130.5$^m$ & 130.3$^M$, 129.3$^m$ & 129.0$^M$, 128.5$^m$, 128.5$^m$, 128.5$^M$, 128.5$^M$, 127.7$^m$ & 127.6$^M$, 127.0$^m$ & 126.8$^M$, 126.4$^M$ & 126.3$^m$, 75.0$^m$ & 74.8$^M$, 44.3$^M$ & 43.3$^m$, 43.0$^M$ & 39.9$^m$, 37.9$^M$ & 36.7$^m$, 28.8$^m$ & 28.0$^M$, 27.9$^M$ & 26.5$^m$, 24.1$^M$ & 20.4$^m$; HRMS (APCI): Found $MH^+$ 302.1891 $C_{22}H_{24}N$ requires 302.1903.

**2-Phenyl-7-(phenylethynyl)-3a,4,5,6,7,7a-hexahydro-3H-indole (7r)**

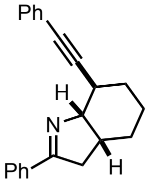

Following **GP16** but in 0.05 M $CH_2Cl_2$, **3f** (30 mg, 0.1 mmol) gave **7r** (16 mg, 53%). d.r 1.5:1. FT-IR $\nu_{max}$ (film)/cm$^{-1}$ 2930, 2855, 1684, 1599, 1572, 1489, 1447, 1330, 1265, 1070, 1016; $^1$H NMR (500 MHz, $CDCl_3$, diastereoisomers) δ 7.91 (1.2H, dd, *J* = 7.8, 1.5 Hz), 7.86 (0.8H, dd, *J* = 7.6, 1.7 Hz), 7.47–7.38 (4H, m), 7.32–7.25 (2.3H, m), 7.22–7.18 (1.7H, m), 4.19 (0.6H, t, *J* = 5.7 Hz), 4.11 (0.4H, s, br), 3.40 (0.4H, q, *J* = 4.3 Hz), 3.23–3.18 (0.6H, m), 2.95–2.87 (1H, m), 2.85 (0.6H, dd, *J* = 15.9, 4.9 Hz), 2.73 (0.4H, dd, *J* = 16.0, 2.3 Hz), 2.64 (0.4H, dtd, *J* = 15.6, 6.3, 3.1 Hz, 1H), 2.51 – 2.43 (0.6H, m), 1.84 – 1.73 (3.3H, m), 1.71 – 1.64 (1H, m), 1.48 – 1.35 (1.7H, m); $^{13}$C NMR (126 MHz, $CDCl_3$, diastereoisomers) δ 175.1$^M$ & 174.4$^m$, 135.3$^M$ & 135.2$^m$, 131.8$^m$ & 131.8$^M$, 130.7$^m$ & 130.5$^M$, 128.6$^m$, 128.5$^M$, 128.3$^m$, 128.1$^M$, 127.9$^M$, 127.7 & 127.4$^m$, 124.3$^M$ & 124.2$^m$, 93.1$^m$ & 92.6$^M$, 82.3$^M$ & 81.9$^m$,

74.4$^{m}$ & 72.3$^{M}$, 42.4$^{m}$ & 42.1$^{M}$, 37.2$^{M}$ & 35.4$^{m}$, 32.7$^{M}$ & 31.8$^{m}$, 28.2$^{M}$ & 27.4$^{m}$, 27.3$^{m}$ & 26.7$^{M}$, 21.3$^{M}$ & 19.8$^{m}$; HRMS (ESI): Found MH$^{+}$ 300.1741 $C_{22}H_{22}N$ requires 300.1747.

**Benzyl 2,4-Dimethyl-4-(5-phenyl-3,4-dihydro-2H-pyrrol-2-yl)pentanoate (8a)**

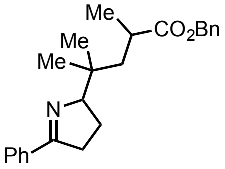


Following **GP13** but using benzyl methacrylate, **3a** (29 mg, 0.1 mmol) gave **8a** (18 mg, 48%).[23] d.r 3:1. FT-IR $\nu_{max}$ (film)/cm$^{-1}$2965, 1732, 1617, 1496, 1455, 1386, 1364, 1340, 1268, 1147, 1065; $^{1}$H NMR (400 MHz, $CDCl_3$, diastereomers) δ 7.83 (2H, dd, *J* = 7.5, 1.6 Hz), 7.42–7.29 (8H, m), 5.16–5.06 (2H, m), 4.02–3.95 (1H, m), 2.96–2.82 (2H, m), 2.82–2.72 (1H, m), 2 2.07 (1H, dd, *J* = 14.3, 9.5 Hz), 2.01–1.91 (1H, m), 1.79–1.69 (m, 1H), 1.47 (0.25H, dd, *J* = 14.2, 2.9 Hz), 1.40 (0.75H, dd, *J* = 14.2, 2.7 Hz), 1.23 (0.75H, d, *J* = 6.9 Hz), 1.22 (2.25H, d, *J* = 7.1 Hz), 0.98 (0.75H, s), 0.93 (2.25H, s), 0.87 (2.25H, s), 0.82 (0.75H, s); $^{13}$C NMR (126 MHz, $CDCl_3$, diastereomers) δ 178.0$^{M}$ & 178.0$^{m}$, 172.1$^{m}$ & 172.0$^{M}$, 136.3$^{M}$ & 136.3$^{m}$, 135.0, 130.3$^{m}$, 128.6, 128.5, 128.3$^{M}$ & 128.3$^{m}$, 128.2, 127.8, 82.2$^{m}$ & 81.6$^{M}$, 66.3$^{M}$, 44.2$^{M}$ & 43.6$^{M}$, 37.8$^{m}$ & 37.7$^{M}$, 35.9$^{M}$ & 35.9$^{m}$, 35.4$^{M}$ & 35.4$^{m}$, 24.2$^{M}$ & 23.9$^{m}$, 23.8$^{M}$ & 23.6$^{m}$, 23.4$^{M}$ & 23.1$^{m}$, 20.8$^{M}$ & 20.7$^{m}$; HRMS (ESI): Found MNa$^{+}$ 386.2080 $C_{24}H_{29}O_2NNa$ requires 386.2091.

**3-(2-(5-Phenyl-3,4-dihydro-2*H*-pyrrol-2-yl)propan-2-yl)cyclohexan-1-one (8b)**

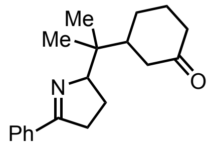


Following **GP13** but using cyclohexanone and with an extra 2 equiv. added after 24 h, **3a** (29 mg, 0.1 mmol) gave **7n** (14 mg, 51%). d.r 1:1. FT-IR $\nu_{max}$ (film)/cm$^{-1}$ 2961, 2869, 1708, 1617, 1576, 1448, 1385, 1365, 1341, 1267, 1234, 1204, 1157, 1059; $^{1}$H NMR (400 MHz, $CDCl_3$, diastereomers) δ 7.86–7.80 (2H, m), 7.43–7.36 (3H, m), 4.23–4.12 (1H, m), 2.99–2.91 (1H, m), 2.91 – 2.81 (1H, m), 2.59 (0.5H, d, *J* = 13.8 Hz), 2.49 (0.5H, br d, *J* = 13.0 Hz), 2.40 (1.5H, br d, *J* = 13.5 Hz), 2.36–1.86 (6H, m), 1.80–1.47 (4.5H, m), 1.27 (2H, dd, *J* = 12.9, 4.5 Hz), 1.00 (1.5H, s), 0.96 (1.5H, s), 0.81 (1.5H, s), 0.78 (1.5H, s); $^{13}$C NMR (101 MHz, $CDCl_3$) δ 213.3 & 213.3, 172.0 & 171.9, 135.0, 130.3, 128.5, 127.7, 79.0 & 78.9, 46.7

[23] **8a** was purified by column chromatography on silica gel eluting with $CH_2Cl_2$→ $CH_2Cl_2$:MeOH 99.9:0.1.

& 46.3, 43.8 & 43.7, 41.7, 39.8 & 39.7, 35.4 & 35.2, 26.2 & 26.1, 25.9 & 25.8, 23.9 & 23.8, 20.9 & 20.6, 20.3; HRMS (APCI): found MH$^+$ 284.2008 $C_{19}H_{26}ON$ requires 284.2009.

**(*E*)-2-(4-Cyclohexyl-2-methylbut-3-en-2-yl)-5-phenyl-3,4-dihydro-2H-pyrrole (8c)**

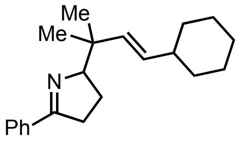


Following **GP15** but using 3 equiv. of **S1**, **3a** (29 mg, 0.1 mmol) gave **8c** (10 mg, 35%). FT-IR $\nu_{max}$ (film)/cm$^{-1}$ 2921, 2850, 1617, 1447, 1381, 1359, 1339, 1271, 1059; $^1$H NMR (400 MHz, $CDCl_3$) δ 7.85 (2H, dd, *J* = 7.0, 2.3 Hz), 7.43–7.38 (3H, m), 5.36 (1H, d, *J* = 15.9 Hz), 5.30 (1H, dd, *J* = 15.9, 6.1 Hz), 4.06–4.00 (1H, m), 2.88–2.82 (2H, m), 2.01–1.92 (1H, m), 1.91–1.82 (1H, m), 1.73–1.58 (6H, m), 1.25–1.17 (2H, m), 1.14 (3H, s), 1.09 (3H, s), 1.02 (3H, m); $^{13}$C NMR (101 MHz, $CDCl_3$) δ 172.5, 134.5, 134.1, 130.3, 128.5, 127.9, 82.3, 41.1, 40.3, 35.5, 33.6, 33.5, 26.4, 26.2, 25.9, 24.4, 24.3; HRMS (APCI): Found MH$^+$ 296.2368 $C_{21}H_{30}N$ requires 296.2373.

**2-(2-Methyl-4-phenylbut-3-yn-2-yl)-5-phenyl-3,4-dihydro-2H-pyrrole (8d)**

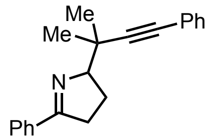


Following **GP16**, **3a** (29 mg, 0.1 mmol) gave **8d** (22 mg, 77%).[24] FT-IR $\nu_{max}$ (film)/cm$^{-1}$ 2968, 1617, 1575, 1489, 1447, 1359, 1340, 1269, 1177, 1060, 1027, 1009; $^1$H NMR (500 MHz, $CDCl_3$) δ 7.91–7.87 (2H, m), 7.44–7.38 (3H, m), 7.31–7.27 (2H, m), 7.24–7.19 (3H, m), 4.24–4.18 (1H, m), 3.05 (1H, dddd, *J* = 15.4, 10.1, 4.9, 2.2 Hz), 3.00–2.90 (1H, m), 2.26–2.17 (1H, m), 2.12 (1H, ddt, *J* = 13.3, 10.3, 6.8 Hz), 1.47 (3H, s), 1.44 (3H, s); $^{13}$C NMR (126 MHz, $CDCl_3$) δ 173.4, 135.0, 131.7, 130.4, 128.5, 128.2, 127.9, 127.5, 124.1, 95.7, 81.9, 81.2, 37.3, 35.7, 28.0, 25.9, 25.3; HRMS (APCI): Found MH$^+$ 288.1747 $C_{21}H_{22}N$ requires 288.1747.

[24] After acid-base wash, **8d** was purified by column chromatography on silica gel eluting with $CH_2Cl_2$.

**2-(2-Methylpent-3-yn-2-yl)-5-phenyl-3,4-dihydro-2H-pyrrole (8e)**

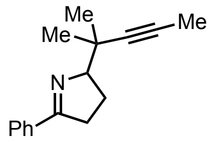

Following **GP16**, **3a** (29 mg, 0.1 mmol) gave **8e** (15 mg, 64%). FT-IR $\nu_{max}$ (film)/cm$^{-1}$ 2968, 1733, 1616, 1575, 1496, 1448, 1340, 1277, 1208, 1167, 1109, 1054; $^{1}$H NMR (500 MHz, $CDCl_3$) δ 7.87 (2H, d, *J* = 7.7 Hz), 7.44–7.37 (3H, m), 4.08 (1H, t, *J* = 7.1 Hz), 3.04–2.96 (1H, m), 2.95–2.86 (1H, m), 2.12 (1H, ddd, *J* = 18.1, 9.0, 4.8 Hz), 2.04–1.95 (1H, m), 1.73 (3H, s), 1.33 (3H, s), 1.30(3H, s); $^{13}$C NMR (126 MHz, $CDCl_3$) δ 173.1, 135.0, 130.4, 128.4, 127.9, 85.0, 82.0, 76.1, 36.5, 35.6, 28.1, 26.1, 25.2, 3.7; HRMS (ASAP): Found MH$^+$ 226.1590 $C_{19}H_{18}N$ requires 226.1590.

**Methyl 2-(2-Chloropropan-2-yl)-3,4-dihydro-2H-pyrrole-5-carboxylate (8f)**

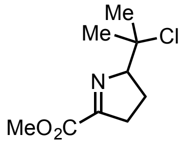

Following **GP5**, **3g** (27 mg, 0.1 mmol) gave **8f** (10 mg, 48%).[25] FT-IR $\nu_{max}$ (film)/cm$^{-1}$ 2961, 1703, 1436, 1387, 1258, 1179, 1020; $^{1}$H NMR (400 MHz, $CDCl_3$) δ 4.38–4.32 (1H, m), 3.88 (3H, s), 3.04–2.94 (1H, m), 2.85–2.74 (1H, m), 2.19–2.08 (1H, m), 2.08–1.99 (1H, m), 1.71 (3H, s), 1.68 (3H, s); $^{13}$C NMR (101 MHz, $CDCl_3$) δ 169.0, 163.4, 84.1, 72.4, 52.9, 36.4, 31.2, 29.7, 24.4; HRMS (APCI): Found MH$^+$ 204.0787 $C_9H_{15}O_2NCl$ requires 204.0786.

**Methyl 2-(2-Azidopropan-2-yl)-3,4-dihydro-2H-pyrrole-5-carboxylate (8g)**

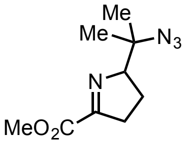

Following **GP9**, **3g** (27 mg, 0.1 mmol) gave **8g** (11 mg, 55%).[25] FT-IR $\nu_{max}$ (film)/cm$^{-1}$ 2961, 2101, 1729, 1437, 1369, 1258, 1144, 1112; $^{1}$H NMR (500 MHz, $CDCl_3$) δ 4.17 (1H, tt, *J* = 8.0, 2.7 Hz), 3.88 (3H, s), 3.00–2.91 (1H, m), 2.78 (1H, dddd, *J* = 18.2, 10.2, 8.0, 2.5 Hz), 2.09–2.01 (1H, m), 1.88 (1H, ddt, *J* = 13.3, 10.4, 7.7 Hz), 1.45 (3H, s), 1.38 (3H, s); $^{13}$C NMR (126 MHz, $CDCl_3$) δ 169.0, 163.1, 82.2, 63.6, 52.8, 36.1, 24.5, 23.5, 23.4; HRMS (APCI): Found MH$^+$ 211.1184 $C_9H_{15}O_2N_4$ requires 211.1190.

[25] **8f** and **8g** was not stable upon acid-base wash. As a result this compound was purified by direct column chromatography on silica gel eluting with $CH_2Cl_2$→$CH_2Cl_2$:MeOH 99.6:0.4.

**Methyl 2-(2-(Phenylthio)propan-2-yl)-3,4-dihydro-2H-pyrrole-5-carboxylate (8h)**

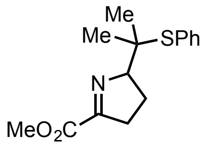


Following **GP11**, **3g** (27, 0.1 mmol) gave **8h** (13 mg, 44%). $^{1}$H NMR (400 MHz, $CDCl_3$) δ 7.54 (2H, dd, *J* = 8.0, 1.4 Hz), 7.39–7.29 (3H, m), 4.22 (1H, tt, *J* = 8.0, 2.8 Hz), 3.86 (3H, s), 3.01–2.90 (1H, m), 2.84–2.73 (1H, m), 2.19–2.10 (2H, m), 1.42 (3H, s), 1.21 (3H, s). $^{13}$C NMR (126 MHz, $CDCl_3$) δ 168.3, 163.5, 137.8, 131.5, 129.1, 128.7, 82.9, 52.8, 52.5, 36.3, 28.0, 24.4, 24.3. HRMS (ASAP): Found MH+ 278.1202 $C_{15}H_{20}O_2NS$ requires 278.1202.

**Methyl 2-(2-methyl-4-(triisopropylsilyl)but-3-yn-2-yl)-3,4-dihydro-2H-pyrrole-5-carboxylate (8i)**

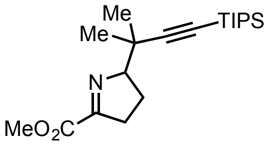


Following **GP16**, **3g** (27 mg, 0.1 mmol) gave **8i** (23 mg, 68%).[26] FT-IR $\nu_{max}$ (film)/cm$^{-1}$ 2942, 2864, 1731, 1462, 1438, 1364, 1260, 1108, 1016; $^{1}$H NMR (400 MHz, $CDCl_3$) δ 4.11 (1H, tt, *J* = 8.2, 2.7 Hz), 3.86 (3H, s), 2.95 (1H, dddd, *J* = 18.4, 9.9, 5.2, 3.2 Hz), 2.75 (1H, dddd, *J* = 18.2, 9.9, 7.9, 2.2 Hz), 2.14–2.00 (2H, m), 1.39 (3H, s), 1.34 (3H, s), 1.02–0.99 (21H, m); $^{13}$C NMR (101 MHz, $CDCl_3$) δ 167.9, 163.6, 113.4, 83.3, 80.9, 52.7, 37.7, 36.3, 28.2, 26.8, 24.7, 18.7, 11.3; HRMS (APCI): Found MH$^{+}$ 350.2508 $C_{21}H_{36}ON_2Si$ requires 350.2512.

[26] **8i** was purified by column chromatography on silica gel eluting with $CH_2Cl_2 \rightarrow CH_2Cl_2$:MeOH 98:2. **8i**-H$^{+}$ was too apolar to remain in the aqueous layer making the acid-base wash ineffective.

**(4b*S*,8*R*,8a*R*,9*R*,9a*R*,12*R*,12a*S*)-9-Azido-1,12a-dimethoxy-7,11-dimethyl-5,6,7,8,9a,12,12a,12b-octahydro-9H-4,8:8a,12-dimethanobenzofuro[3,2-e]pyrrolo[3,2-g]isoquinoline (10a)**

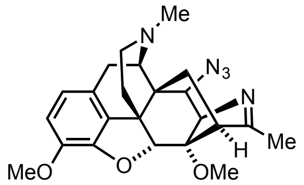


Following **GP9**, **3h** (48 mg, 0.1 mmol) gave **10a** (18 mg, 43).[27] FT-IR $\nu_{max}$ (film)/cm$^{-1}$ 2928, 1637, 1598, 1440, 1377, 1319, 1276, 1256, 1204, 1152, 1090, 1048, 1006; $^{1}$H NMR (400 MHz, $CDCl_3$) δ 6.72 (1H, d, *J* = 8.1 Hz), 6.59 (1H, d, *J* = 8.0 Hz), 4.90 (1H, s), 3.88 (3H, s), 3.83 (1H, d, *J* = 7.4 Hz), 3.42 (3H, s), 3.11 (1H, d, *J* = 18.5 Hz), 2.77–2.65 (2H, m), 2.46 (2H, d, *J* = 8.9 Hz), 2.36 (1H, t, *J* = 12.0 Hz), 2.28 (3H, s), 2.16 (2H, dd, *J* = 17.3, 3.9 Hz), 2.06 (3H, s), 1.67 (1H, d, *J* = 12.9 Hz), 1.23 (1H, d, *J* = 13.6 Hz), 0.95–0.83 (2H, m); $^{13}$C NMR (101 MHz, $CDCl_3$) δ 181.1, 146.5, 142.7, 132.5, 128.1, 119.5, 114.0, 92.3, 87.6, 62.1, 61.5, 56.8, 53.8, 49.7, 45.4, 44.9, 43.6, 38.7, 33.2, 27.9, 21.9, 19.2. In this case the HRMS could not be obtained.

**Diethyl 1-((4bS,8R,8aR,9R,9aR,12R,12aS)-1,12a-dimethoxy-7,11-dimethyl 5,6,7,8,9a,12,12a,12b-octahydro-9H-4,8:8a,12-dimethanobenzofuro[3,2-e]pyrrolo[3,2-g]isoquinolin-9-yl)hydrazine-1,2-dicarboxylate (10b)**

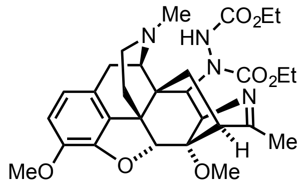


Following **GP11**, **3h** (48 mg, 0.1 mmol) gave **10b** (26 mg 41 %).[28] d.r 4:1 FT-IR $\nu_{max}$ (film)/cm$^{-1}$ 2933, 1714, 1634, 1499, 1442, 1372, 1312, 1216, 1158, 1094, 1054; $^{1}$H NMR (500 MHz, toluene-$d_8$, 100 ˚C) δ 6.73 (1H, d, *J* = 8.1 Hz), 6.59 (1H, d, *J* = 7.7 Hz), 4.66 (1H, s), 4.39 (1H,d, *J* = 7.7 Hz), 4.01 (2H, m), 3.91 – 3.82 (3H, m), 3.15 (3H, s), 3.05 (1H, d, *J* = 18.4 Hz), 2.60 – 2.47 (2H, m), 2.41 – 2.35 (1H, m), 2.28 – 2.21 (5H, m), 2.05 – 1.97 (2H, m), 1.88 (3H, s), 1.75 (1H, d, *J* = 14.9 Hz), 1.40 (1H, dd, *J* = 13.1, 3.9 Hz), 1.05 (3H, t, *J* = 7.1 Hz), 0.92 (3H, t, *J* = 7.1 Hz); $^{13}$C NMR (126 MHz, toluene-$d_8$, T = 100 ºC) δ 183.4, 157.0, 156.4, 147.9, 143.8, 133.2, 128.5, 121.1, 117.7, 93.4, 89.4, 67.6, 62.7, 61.9, 61.7, 58.7, 57.8,

[27] **10a** was purified by column chromatography on silica gel eluting with $CH_2Cl_2$→$CH_2Cl_2$:MeOH 99.5:0.5.
[28] **10b** was purified by column chromatography on silica gel eluting with $CH_2Cl_2$→$CH_2Cl_2$:MeOH 95:5.

54.2, 53.8, 52.3, 46.4, 46.0, 43.7, 42.5, 34.5, 23.6, 22.7, 18.7, 14.7, 14.5. HRMS (APCI): Found MH$^+$ 555.2801 $C_{29}H_{39}O_7N_4$ requires 555.2813.

**(4b*R*,8*R*,8a*R*,9a*S*,12*S*,12a*S*,12b*R*)-1,12a-Dimethoxy-7,11-dimethyl-9-(phenylselanyl)-5,6,7,8,9a,12,12a,12b-octahydro-9*H*-4,8:8a,12-dimethanobenzofuro[3,2-*e*]pyrrolo[3,2-*g*]isoquinoline (10c)**

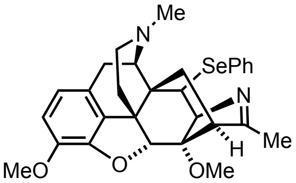


Following **GP12**, **3h** (48 mg, 0.1 mmol) gave **10c** (33 mg, 61%).[29] FT-IR $\nu_{max}$ (film)/cm$^{-1}$ 2933, 1635, 1501, 1437, 1259, 1207, 1151, 1085; $^1$H NMR (400 MHz, $CDCl_3$, isolated diastereomer) δ 7.31–7.27 (2H, m), 7.25–7.14 (3H, m), 6.70 (1H, d, *J* = 8.1 Hz), 6.17 (1H, d, *J* = 8.1 Hz), 4.85 (1H, s), 4.33 (1H, d, *J* = 8.8 Hz), 3.91 (3H, s), 3.44 (3H, s), 2.98 (1H, d, *J* = 6.5 Hz), 2.78–2.71 (1H, m), 2.60 (1H, dd, *J* = 8.4, 2.3 Hz), 2.49–2.44 (2H, m), 2.41–2.32 (1H, m), 2.26–2.21 (2H, m), 2.17 (3H, s), 2.13 (3H, s), 1.70 (1H, dd, *J* = 13.1, 2.9 Hz), 1.58 (1H, d, *J* = 14.4 Hz), 0.74 (1H, dd, *J* = 18.9, 6.6 Hz);$^{13}$C NMR (101 MHz, $CDCl_3$, isolated diastereomer) δ 184.2, 146.6, 142.5, 134.2, 132.0, 131.8, 129.2, 127.4, 120.2, 114.3, 92.7, 87.7, 65.0, 60.9, 56.9, 54.2, 50.5, 50.2, 46.4, 45.5, 43.7, 43.5, 34.9, 24.1, 21.1, 18.9; HRMS (APCI): Found MH$^+$ 537.1637 $C_{29}H_{33}O_3N_2Se$ requires 537.1651.
In this case we have observed the formation of the demethylated product **nor-10c**.

**(4b*R*,8*R*,8a*R*,9a*S*,12*S*,12a*S*,12b*R*)-1,12a-dimethoxy-11-methyl-9-(phenylselanyl)-5,6,7,8,9a,12,12a,12b-octahydro-9*H*-4,8:8a,12-dimethanobenzofuro[3,2-*e*]pyrrolo[3,2-*g*]isoquinoline (nor-10c)**

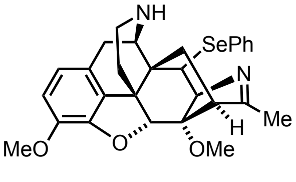


FT-IR $\nu_{max}$ (film)/cm$^{-1}$ 2960, 2359, 1635, 1502, 1437, 1288, 1250, 1205, 1164, 1087, 1055; $^1$H NMR (400 MHz, $CDCl_3$) δ 7.29 (2H, d, *J* = 7.3 Hz), 7.23 (1H, d, *J* = 7.1 Hz), 7.16 (2H, t, *J* = 7.2 Hz), 6.72 (1H, d, *J* = 8.1 Hz), 6.18 (1H, d, *J* = 7.9 Hz), 4.83 (1H, s), 4.36 (1H, d, *J* = 8.4 Hz), 3.92 (3H, s, *J* = 9.8 Hz), 3.44 (3H, s), 3.30 (1H, d, *J* = 6.4 Hz), 2.87 (1H, td, *J* =

[29] **10c** was purified by column chromatography on silica gel eluting with $CH_2Cl_2$→$CH_2Cl_2$:MeOH 96:4.

12.8, 2.3 Hz), 2.77 (1H, dd, $J$ = 12.4, 4.3 Hz), 2.71 – 2.62 (1H, m), 2.60 (1H, d, $J$ = 8.4 Hz), 2.51 (1H, d, $J$ = 10.1 Hz), 2.34 (1H, d, $J$ = 18.8 Hz), 2.14 (3H, s), 2.12 – 2.04 (1H, m), 1.70 – 1.62 (2H, m), 1.31 (1H, dd, $J$ = 18.9, 6.8 Hz); $^{13}$C NMR (126 MHz, $CDCl_3$) δ 184.1, 146.7, 142.7, 134.3, 131.7, 131.2, 129.3, 127.6, 120.3, 114.5, 92.8, 87.4, 65.1, 56.9, 54.3, 53.7, 50.3, 50.0, 47.0, 42.7, 36.9, 33.5, 32.2, 23.7, 19.0; GCMS (EI): 522 ($M^+$), 491, 442, 365, 333, 157.

## 7 Mechanistic Considerations

### 7.1 Emission Quenching Experiments – Stern-Volmer Studies

Emission intensities were recorded using a Steady State emission spectra were recorded on an Edinburgh Instrument FP920 Phosphorescence Lifetime Spectrometer equipped with a 5 watt microsecond pulsed xenon flash lamp and a 450 watt steady state xenon lamp and a red sensitive photomultiplier in peltier (air cooled) housing, (Hamamatsu R928P) spectrophotometer. All the solutions of **PC1** were excited at 450 nm and the emission intensity was collected at 510 nm.

*Experimental procedures:*

A Schlenck quartz cuvette was charged with a 1.6 $10^{-2}$ mM solution of **PC1** in $CH_2Cl_2$ (2.0 mL) and the initial emission was collected then the appropriate amount of the **1d**-Cs as a 1.6 $10^{-2}$ M solution in $CH_3CN$ was added. The sample was shaken for 1 min and then the emission of the sample was collected. It is important to keep the solutions completely $O_2$-free.

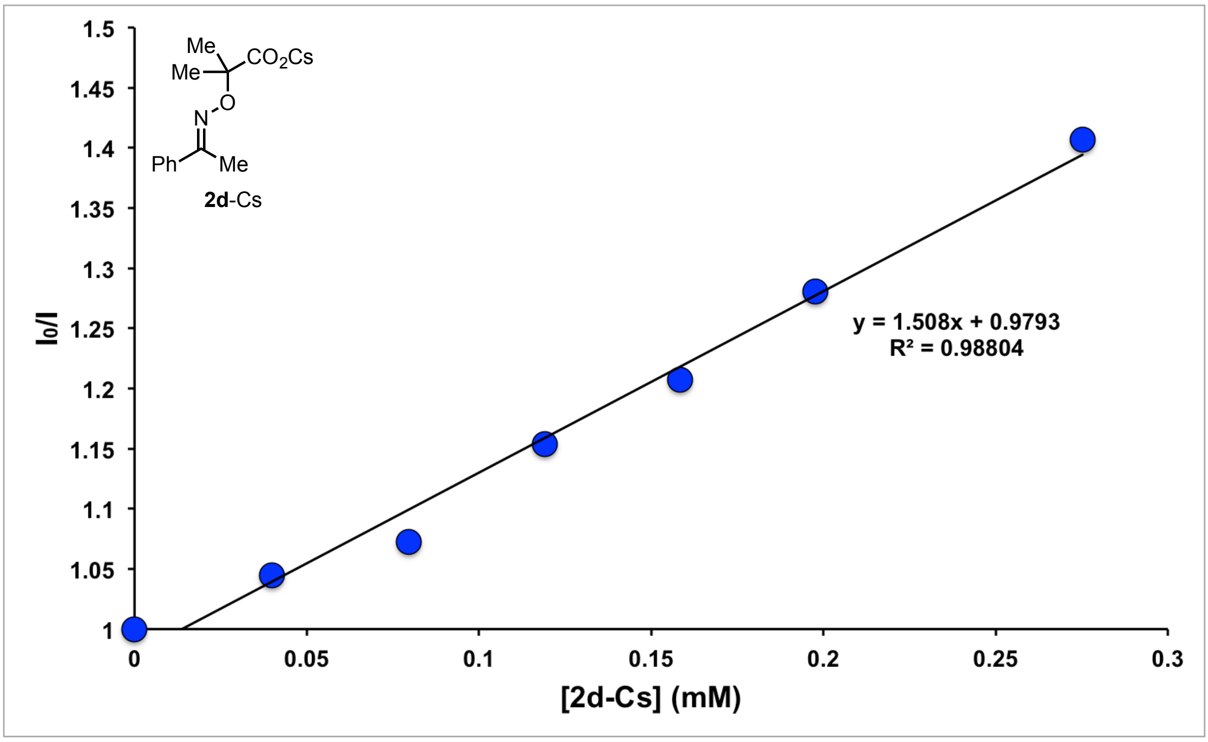

## 7.2 Quantum Yield Determination

We have selected the hydroimination, iminofluorination and iminoazidation for quantum yield determination as these three processes covers the range of reaction times for all the imino-functionalization reactions. The quantum yield determination was performed following the procedure reported by Yoon[23] and are the average of two runs.

| Reaction | Quantum Yield (Φ) |
|---|---|
| 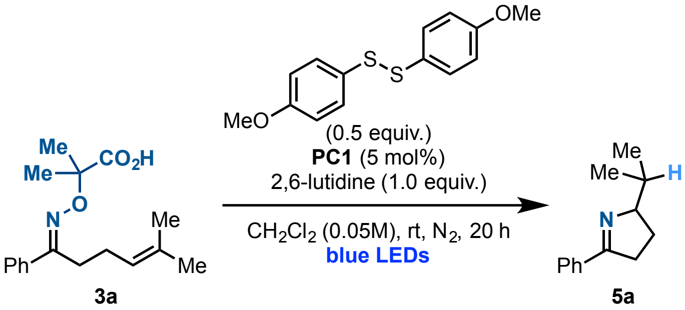  | 0.09 |
| 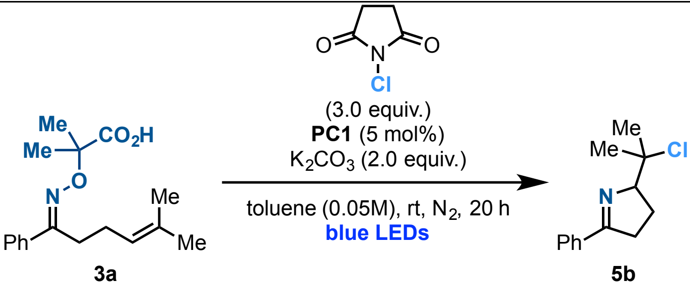  | 0.27 |
| 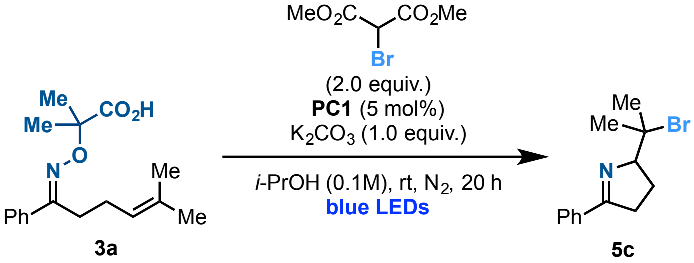  | 0.15 |
| 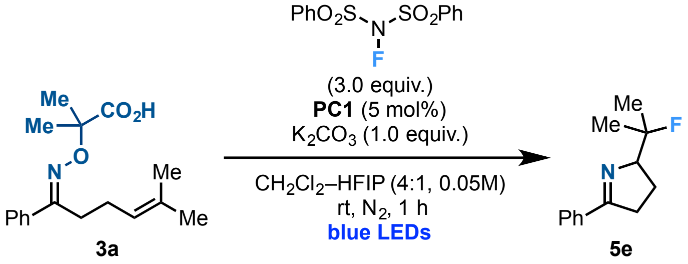  | 0.32 |

| Reaction | Quantum Yield (Φ) |
|---|---|
| 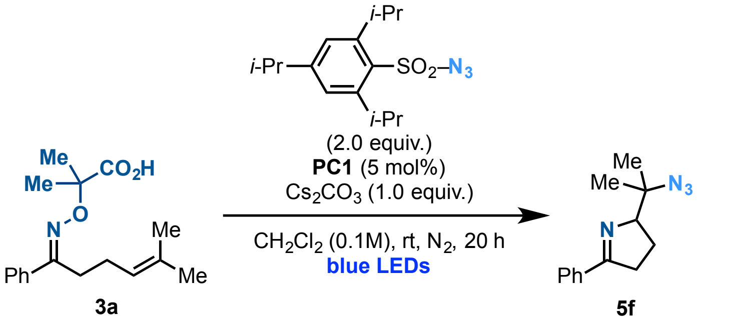 | 0.15 |
| 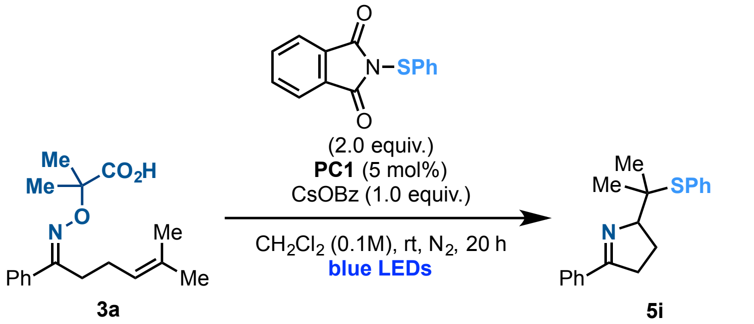 | 0.01 |
| 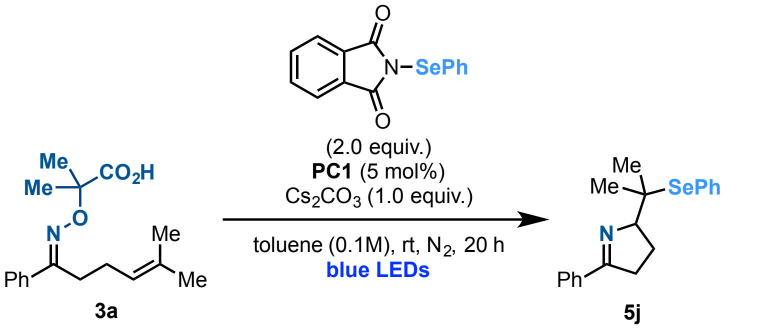 | 0.02 |
| 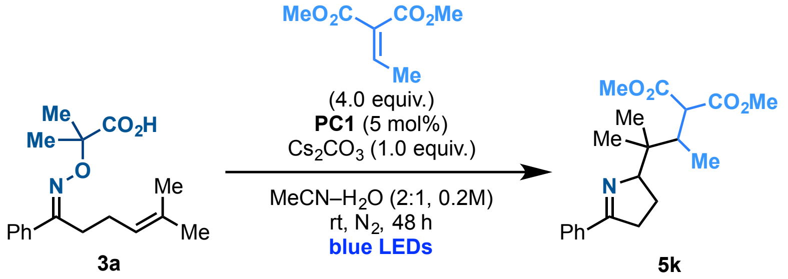 | 0.02 |
| 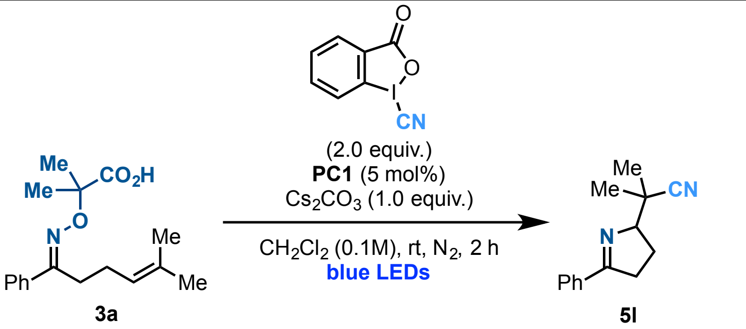 | 2.13 |

| Reaction | Quantum Yield (Φ) |
|---|---|
| 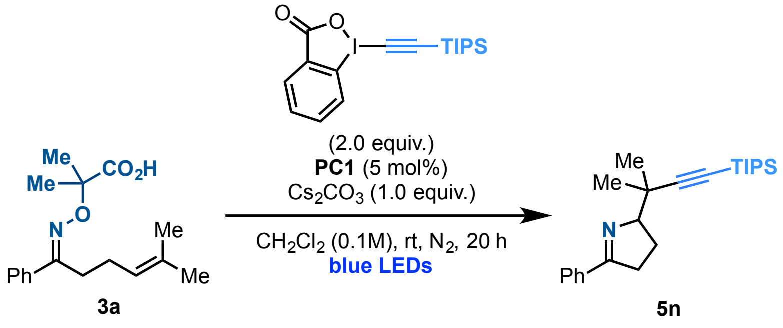 | 4.89 |

In the case of the reaction with the IBX-reagents quantum yields higher than 1 were observed. This suggests that productive short-lived radical chain processes might be operating alongside the main photoredox cycle.

## 7.3 Substrate Oxidation and Fragmentation

### *Substrate Oxidation*

The high oxidation potential observed for the oximes **2a–c** is difficult to rationalise. A possible explanation might be found in an anomeric effect whereby one of the carboxylate lone-pairs is in hyperconjugation with the N–O σ* orbital.

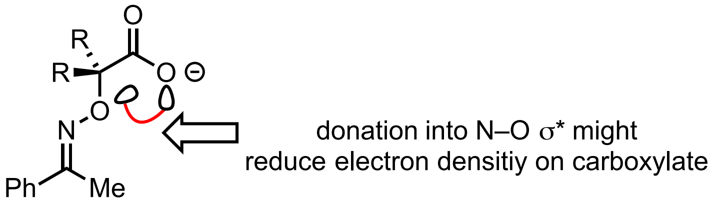


In this case, the introduction of R groups at the methylenic position might destabilise this conformation and favour one where this anomeric effect is not present. We have evaluated the relative stability of several possible conformations for all the model oximes **2a–c** but unfortunately our studies did not provide conclusive evidence (see Section 8.4).

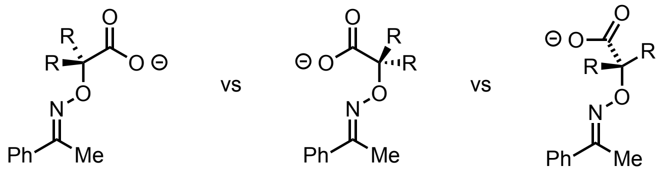

### *Substrate Fragmentation*

In general, upon SET oxidation a significant weakening of the N–O bond takes place which is in line with a very fast fragmentation. This is also in line with the irreversible oxidation profile of the oximes **2a–c** found in the electrochemical studies (see Sections 4.4 and 4.5). The weakening of the N–O bond is shown here for the model oxime **2c** and the full study is reported in Section 8.3.

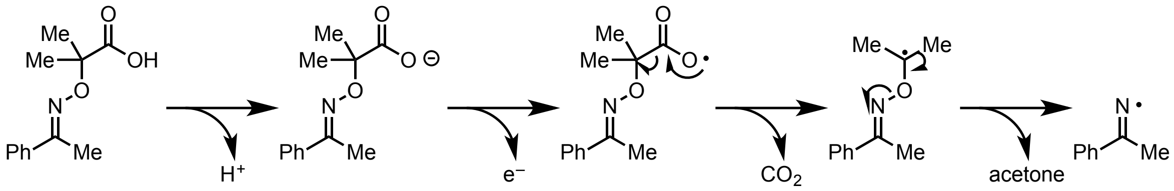


| BDE$_{N–O}$ (Kcal mol$^{-1}$) | | | |
|---|---|---|---|
| 52.6 | 36.0 | –47.7 | –25.9 |

## 7.4 Nucleophilicity of the Carbon-Radicals

In general, the reaction of α–N and α–O radicals with some of the X–Y reagents described here is very favourable owing to the high nucleophilicity of these radicals.

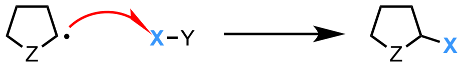


However, the reactivity of carbon-radicals with a β–N or a β–O substituents is modulated by a generalised anomeric effect whereby the semi-occupied orbital undergoes stabilising interactions with the C–N or C–O σ* orbital.[24] This enthalpic effect can decrease the radical reactivity.

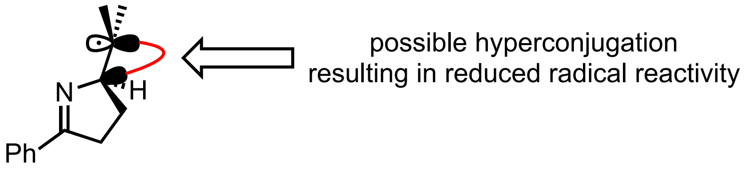


We have performed DFT studies to evaluate the most stable conformation of carbon-radicals **A1–3** after iminyl cyclization.

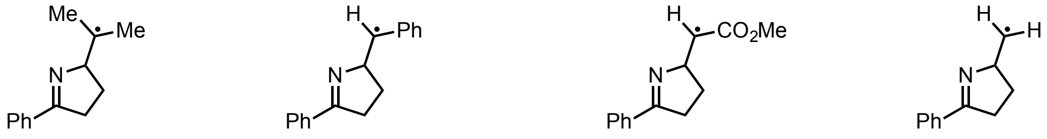


According to our calculations, the more stable conformer for all the radicals (with the exception of α–ester **A3**) arranges the semi-occupied orbital sin-periplanar to the C–N bond thus leading to a potential hyper-conjugation with the C–N σ* orbital. This should decrease the overall nucleophilicity of the radical.

We have then calculated the radicals' electrophilicity and compared them with the values obtained for the related cycloexenyl radicals **B1–4** that are lacking the N-atom.

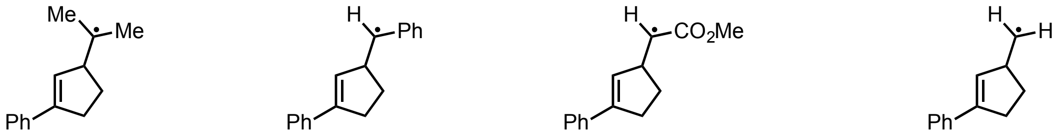

According to these results, there is no significant difference in terms radical nucleophilicty between the two series. As a result, we do not believe that the β–N-substituent enthalpic effect decreases the reactivity of the radicals used in this work. The full DFT studies are reported in Section 8.2.

**Electrophilicity Index ($\omega^{+}_{rc}$) Scale**

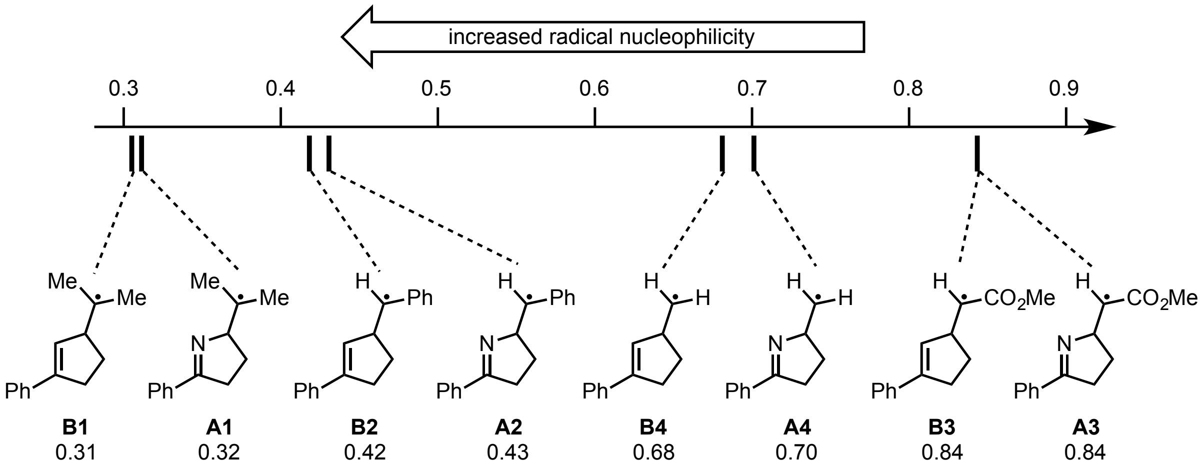

# 8 Computational Studies

## 8.1 Computational Methods

Density functional theory (DFT)[25] calculations were performed using the Gaussian 09W programme[26] and the results were produced with GaussView 5.0. For calculation of global and local electrophilicity index, B3LYP functional[27-30] was used and the geometries of studied radicals were optimized at the UB3LYP/6-311+G(d,p) level of theory, followed by frequency calculations at the same level.[31] The computed Hirshfeld charges[32] on the carbon atom bearing radical, the transition structures for the cyclization and radical addition reactions were also calculated at the same level of theory.[33] The frequency calculations were run to confirm each stationary point to be either a minimum or a transition structure. Representative transition states were also linked to their corresponding minima through the intrinsic reaction coordinate (IRC)[34-36] calculations,[30] which confirm the connection of transition structures with the reactants and products. For substrates having more than one conformations, low energy conformation of the transition state could possibly be different from the low energy ground state.[37] In such a situation, the conformations with the lowest energy have been chosen. Structural analysis for the carboxylate anions was performed using UB3LYP/6-31+G(d,p) level of theory. Homolytic bond dissociation enthalpies (BDE) were calculated using (RO)B3P86/6-311G(d,p) for the determination of geometries, frequencies (scaled by a factor of 0.9806) and molecular energies.[38-40]

[30] Due to unsuccessful IRC calculations for some cases, the transition state structures were linked to their corresponding minima *via* perturbing their structures in the direction along the vibrational coordinate linked with the imaginary frequency, followed by optimization.

**Computed Energies** [values are in Hartree]

| No. | Radical | Total Electronic Energy | Sum of Electronic and Zero-point Energies | Gibbs Free Energy |
|---|---|---|---|---|
| 1 | 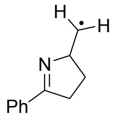 | -481.2035736 | -481.004815 | -481.042986 |
| 2 | 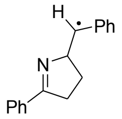 | -712.3334397 | -712.052246 | -712.097996 |
| 3 | 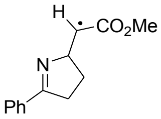 | -709.1623377 | -708.919344 | -708.964516 |
| 4 | 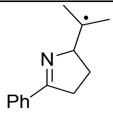 | -559.863734 | -559.608509 | -559.650973 |
| 5 | 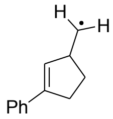 | -465.1507162 | -464.940489 | -464.978706 |
| 6 | 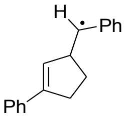 | -696.2808285 | -695.988122 | -696.034100 |
| 7 | 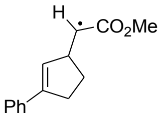 | -693.1091196 | -692.854294 | -692.899624 |
| 8 | 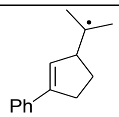 | -543.8090446 | -543.542665 | -543.585572 |

## 8.2 Carbon-Centered Radicals

***DFT Method:*** UB3LYP/6-311+G(d,p) [values are in eV]

**Electronic Properties**

| No. | Radical | Ionization Potential (IP) | Electron affinity (A) | Electro negativity (χ) | Electronic Chemical Potential (μ) | Chemical Hardness (η) | Chemical Softness (S, meV) | Global Electrophilicity (ω) | Electrophilicity Index ($\omega^{+}_{rc}$) | Hirshfeld Charge |
|---|---|---|---|---|---|---|---|---|---|---|
| 1 | 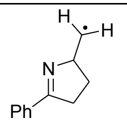 | 8.00 | 0.38 | 4.19 | -4.19 | 7.62 | 131.20 | 1.15 | 0.70 | 0.003668 |
| 2 | 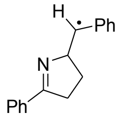 | 6.62 | 1.02 | 3.82 | -3.82 | 5.59 | 178.83 | 1.30 | 0.43 | 0.004686 |
| 3 | 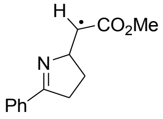 | 8.11 | 1.40 | 4.76 | -4.76 | 6.72 | 148.86 | 1.68 | 0.84 | 0.051386 |
| 4 | 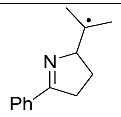 | 6.78 | -0.04 | 3.37 | -3.37 | 6.81 | 146.75 | 0.83 | 0.32 | 0.012554 |
| 5 | 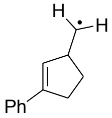 | 7.54 | 0.28 | 3.91 | -3.91 | 7.26 | 137.70 | 1.05 | 0.68 | -0.008185 |
| |  | | | | | | | | | |

| | | | | | | | | | | |
|---|---|---|---|---|---|---|---|---|---|---|
| 7 | 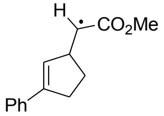 | 7.84 | 1.50 | 4.67 | -4.67 | 6.34 | 157.73 | 1.72 | 0.84 | 0.049823 |
| 8 | 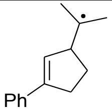 | 6.54 | -0.15 | 3.20 | -3.20 | 6.70 | 149.35 | 0.76 | 0.31 | 0.014275 |

**Computed Energies** [values are in Hartree]

| No. | Radical | Total Electronic Energy | Sum of Electronic and Zero-point Energies | Gibbs Free Energy |
|---|---|---|---|---|
| 1 | 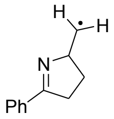 | -481.2035736 | -481.004815 | -481.042986 |
| 2 | 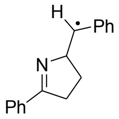 | -712.3334397 | -712.052246 | -712.097996 |
| 3 | 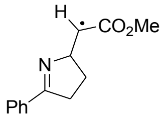 | -709.1623377 | -708.919344 | -708.964516 |
| 4 | 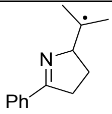 | -559.863734 | -559.608509 | -559.650973 |
| 5 | 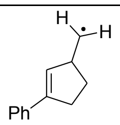 | -465.1507162 | -464.940489 | -464.978706 |
| 6 | 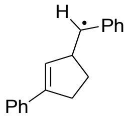 | -696.2808285 | -695.988122 | -696.034100 |
| 7 | 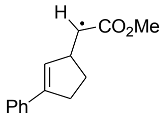 | -693.1091196 | -692.854294 | -692.899624 |
| 8 | 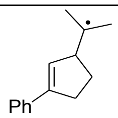 | -543.8090446 | -543.542665 | -543.585572 |

## Optimized Structures and Cartesian Coordinates

| No. | Radical Species | Optimized Structure |
|---|---|---|
| 1 | 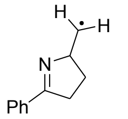 | 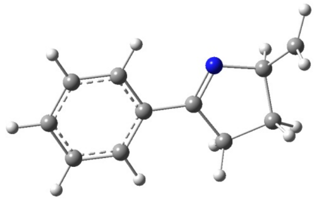 |

Cartesian Coordinates

| | | | |
|---|---|---|---|
| C | -0.59183800 | 0.18822100 | -0.11303200 |
| C | -1.29699100 | 1.53671600 | -0.04258300 |
| C | -2.76287500 | 1.11131700 | 0.13991000 |
| C | -2.78039100 | -0.35963100 | -0.35534100 |
| H | -1.13604200 | 2.08619500 | -0.97817700 |
| H | -0.92557800 | 2.16867400 | 0.76749700 |
| H | -3.03479300 | 1.14260900 | 1.19895000 |
| H | -3.47056100 | 1.74129400 | -0.40110600 |
| H | -3.05192800 | -0.40345800 | -1.41859200 |
| N | -1.36460200 | -0.81793500 | -0.27996600 |
| C | -3.69041400 | -1.25623400 | 0.41155900 |
| H | -3.72792400 | -1.18461400 | 1.49327600 |
| H | -4.17739000 | -2.09991500 | -0.06104100 |
| C | 0.87825600 | 0.03804400 | -0.03960200 |
| C | 1.71860700 | 1.15729600 | 0.02739900 |
| C | 1.45770600 | -1.24120700 | -0.04180000 |
| C | 3.10308300 | 1.00485500 | 0.08885900 |
| H | 1.29612500 | 2.15513800 | 0.02730300 |
| C | 2.83657900 | -1.39151000 | 0.02385500 |
| H | 0.80417300 | -2.10281600 | -0.09521300 |
| C | 3.66560900 | -0.26868000 | 0.08861400 |
| H | 3.73973600 | 1.88116500 | 0.13744300 |
| H | 3.27028500 | -2.38540100 | 0.02395600 |
| H | 4.74212600 | -0.38846000 | 0.13844800 |

| No. | Radical Species | Optimized Structure |
|---|---|---|
| 2 | 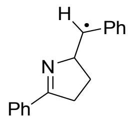 | 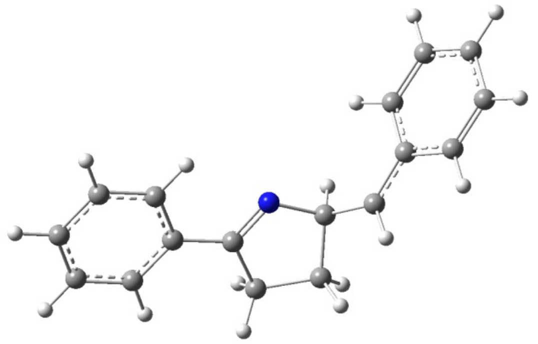 |

Cartesian Coordinates

| | | | |
|---|---|---|---|
| C | -1.55571300 | 0.70631100 | 0.12945000 |
| C | -1.52878800 | 2.22178700 | 0.28431900 |
| C | -0.04077600 | 2.53146900 | 0.05444200 |
| C | 0.65617100 | 1.16856600 | 0.30921100 |
| H | -1.85965700 | 2.49348000 | 1.29418800 |
| H | -2.19109900 | 2.73373500 | -0.41770500 |
| H | 0.12297400 | 2.83972800 | -0.98216800 |
| H | 0.34411700 | 3.32354200 | 0.69858400 |
| H | 0.97797700 | 1.10610200 | 1.35633900 |
| N | -0.40700200 | 0.14218500 | 0.14424300 |
| C | 1.82478500 | 0.90820400 | -0.58763400 |
| H | 1.74686300 | 1.29247100 | -1.60156100 |
| C | -2.80653600 | -0.07476200 | 0.01445400 |
| C | -4.05942300 | 0.54590600 | 0.10611400 |

| | | | |
|---|---|---|---|
| C | -2.75492800 | -1.46403100 | -0.18541200 |
| C | -5.23253000 | -0.20050400 | 0.00341300 |
| H | -4.12532100 | 1.61608700 | 0.26271700 |
| C | -3.92428000 | -2.20545700 | -0.29104700 |
| H | -1.78470500 | -1.93961800 | -0.25550600 |
| C | -5.16853700 | -1.57686700 | -0.19641500 |
| H | -6.19416000 | 0.29462800 | 0.07894600 |
| H | -3.86969700 | -3.27709500 | -0.44762000 |
| H | -6.08019700 | -2.15808900 | -0.27861100 |
| C | 2.98283900 | 0.16651000 | -0.26291100 |
| C | 4.02652000 | 0.03118100 | -1.22462200 |
| C | 3.17751700 | -0.47158700 | 0.99543400 |
| C | 5.18083400 | -0.67699100 | -0.94282200 |
| H | 3.90263000 | 0.50056900 | -2.19506300 |
| C | 4.33767600 | -1.17876800 | 1.26589200 |
| H | 2.40044600 | -0.42015400 | 1.74774900 |
| C | 5.34994600 | -1.28744900 | 0.30611500 |
| H | 5.95803300 | -0.76024500 | -1.69472800 |
| H | 4.45798000 | -1.65834100 | 2.23132500 |
| H | 6.25416700 | -1.84319100 | 0.52552500 |

3

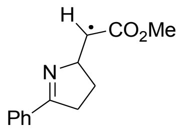


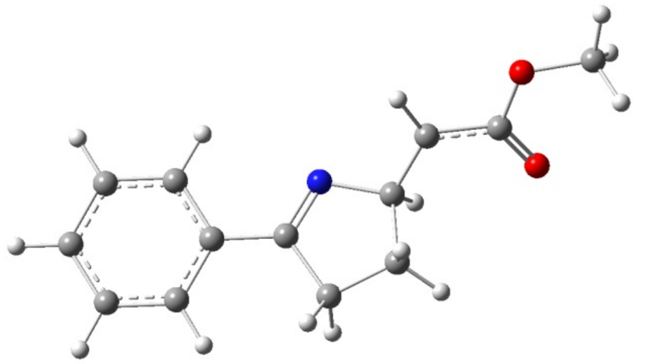

Cartesian Coordinates

| | | | |
|---|---|---|---|
| C | -1.10043600 | 0.42546600 | 0.08402700 |
| C | -0.64516900 | 1.86330900 | -0.13490800 |
| C | 0.87497700 | 1.69500800 | -0.28357800 |
| C | 1.12748500 | 0.31330000 | 0.40657700 |
| H | -0.91052800 | 2.47113200 | 0.73869200 |
| H | -1.11250300 | 2.32844400 | -1.00551700 |
| H | 1.14840500 | 1.63748900 | -1.34046200 |
| H | 1.46195500 | 2.49015200 | 0.17456600 |
| H | 1.37920200 | 0.48884400 | 1.46192600 |
| N | -0.15906800 | -0.39745500 | 0.35917700 |
| C | 2.22365300 | -0.48312600 | -0.20082400 |
| C | -2.51583400 | 0.00434500 | 0.02415400 |
| C | -3.53873200 | 0.93609000 | -0.19748400 |
| C | -2.85835700 | -1.34702100 | 0.19462400 |
| C | -4.87140100 | 0.53048000 | -0.24768800 |
| H | -3.30006600 | 1.98494500 | -0.32753600 |
| C | -4.18596200 | -1.74999900 | 0.13959100 |
| H | -2.06529000 | -2.06305600 | 0.36955800 |
| C | -5.19820300 | -0.81262400 | -0.08150700 |
| H | -5.65167500 | 1.26382700 | -0.41722800 |
| H | -4.43679500 | -2.79677000 | 0.27031900 |
| H | -6.23420900 | -1.12982400 | -0.12286800 |
| C | 3.60673800 | -0.19382300 | 0.10748800 |
| O | 3.98733900 | 0.67279700 | 0.87794200 |
| O | 4.45965900 | -1.00851300 | -0.57083200 |
| C | 5.85720200 | -0.79433000 | -0.32149800 |
| H | 6.08706600 | -0.94840500 | 0.73435100 |
| H | 6.37801600 | -1.52535700 | -0.93640500 |
| H | 6.14732600 | 0.21961300 | -0.60301900 |
| H | 1.99082500 | -1.27556900 | -0.90135400 |

4

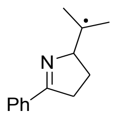

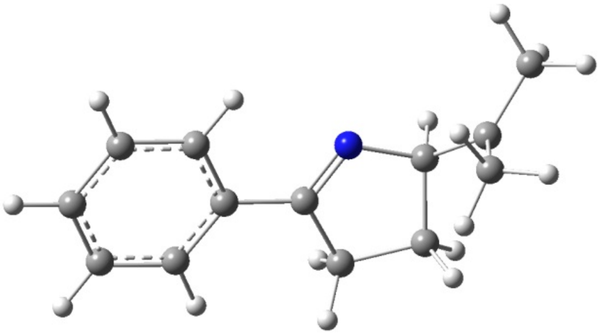


Cartesian Coordinates

| | | | |
|---|---|---|---|
| C | 0.15066900 | 0.50146200 | -0.18970800 |
| C | -0.30167000 | 1.94654200 | -0.02090500 |
| C | -1.82854000 | 1.78956700 | 0.07087000 |
| C | -2.09037100 | 0.37907200 | -0.53575800 |
| H | 0.00474000 | 2.53247100 | -0.89608300 |
| H | 0.13850800 | 2.42825200 | 0.85572400 |
| H | -2.15006700 | 1.81325700 | 1.11462400 |
| H | -2.37725600 | 2.56969600 | -0.45891400 |
| H | -2.32227300 | 0.47653300 | -1.60452400 |
| N | -0.78414100 | -0.32708300 | -0.46528900 |
| C | -3.19541700 | -0.40844900 | 0.11052900 |
| C | 1.56579200 | 0.08133400 | -0.08928400 |
| C | 2.58755200 | 1.02041200 | 0.10535000 |
| C | 1.90969300 | -1.27632400 | -0.19417600 |
| C | 3.91915600 | 0.61656300 | 0.19181000 |
| H | 2.34817000 | 2.07414700 | 0.18558900 |
| C | 3.23602700 | -1.67760700 | -0.10371600 |
| H | 1.11762900 | -1.99847300 | -0.34748600 |
| C | 4.24699300 | -0.73260300 | 0.08931400 |
| H | 4.69788700 | 1.35639000 | 0.33970000 |
| H | 3.48697600 | -2.72955100 | -0.18406900 |
| H | 5.28194300 | -1.04852300 | 0.15867600 |
| C | -4.20311100 | -1.10246200 | -0.74750700 |
| C | -3.04781600 | -0.85560600 | 1.53016100 |
| H | -5.13063500 | -1.30360300 | -0.20117900 |
| H | -4.45076100 | -0.52093200 | -1.64107600 |
| H | -3.82784700 | -2.07919600 | -1.09965500 |
| H | -4.01916500 | -0.91565500 | 2.03410900 |
| H | -2.60084600 | -1.86127000 | 1.58520000 |
| H | -2.40175300 | -0.19538400 | 2.11451500 |

5

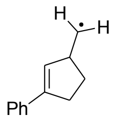


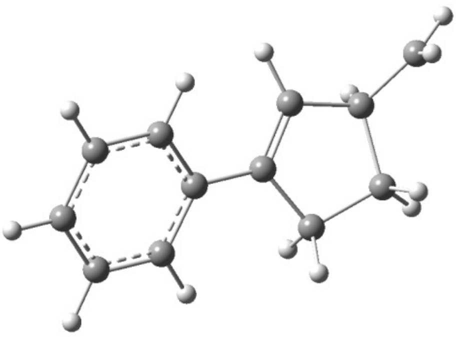

Cartesian Coordinates

| | | | |
|---|---|---|---|
| C | -0.55317000 | 0.16520800 | -0.08804900 |
| C | -1.25039000 | 1.49855400 | 0.13224500 |
| C | -2.72947800 | 1.10251800 | 0.31731100 |
| C | -2.88494400 | -0.27670100 | -0.40477800 |
| H | -1.11559400 | 2.14595600 | -0.74439500 |
| H | -0.85392200 | 2.04602500 | 0.99250400 |

| | | | |
|---|---|---|---|
| H | -2.93895500 | 0.96318700 | 1.38174600 |
| H | -3.43195600 | 1.84730800 | -0.06194600 |
| H | -3.18782000 | -0.10837300 | -1.44821400 |
| C | -3.87408200 | -1.18958100 | 0.23472900 |
| H | -3.71070600 | -1.53453600 | 1.25028600 |
| H | -4.77349300 | -1.51114400 | -0.27511600 |
| C | 0.91077500 | 0.01448600 | -0.03112100 |
| C | 1.74937500 | 1.13718900 | -0.12065600 |
| C | 1.51656900 | -1.24589400 | 0.11858900 |
| C | 3.13621400 | 1.00566500 | -0.08275400 |
| H | 1.31516000 | 2.12381600 | -0.23140900 |
| C | 2.89921200 | -1.37744300 | 0.15743300 |
| H | 0.89491600 | -2.12757000 | 0.22267400 |
| C | 3.71853200 | -0.25149000 | 0.05467800 |
| H | 3.76102900 | 1.88875000 | -0.15965200 |
| H | 3.34152000 | -2.36030800 | 0.27818300 |
| H | 4.79713000 | -0.35499700 | 0.08879400 |
| C | -1.45218900 | -0.78453600 | -0.39218000 |
| H | -1.21585600 | -1.80595800 | -0.66612900 |

6

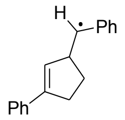


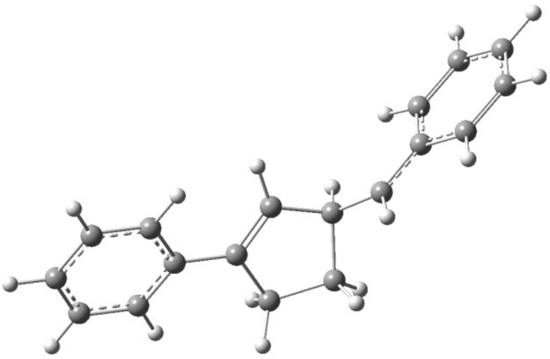

Cartesian Coordinates

| | | | |
|---|---|---|---|
| C | -1.66482300 | 0.53281900 | -0.00853000 |
| C | -1.42944100 | 2.02675900 | -0.17095900 |
| C | 0.07658700 | 2.12040100 | -0.49120300 |
| C | 0.70828200 | 0.82091000 | 0.09487800 |
| H | -1.67454200 | 2.55202100 | 0.76164600 |
| H | -2.05116600 | 2.47042900 | -0.95424100 |
| H | 0.21812500 | 2.12122200 | -1.57595500 |
| H | 0.54997200 | 3.02273800 | -0.09920800 |
| H | 1.04486100 | 1.01233900 | 1.12142900 |
| C | 1.84415100 | 0.28812900 | -0.71912300 |
| H | 1.63043000 | 0.13609600 | -1.77503100 |
| C | -3.01227400 | -0.06020600 | 0.03120900 |
| C | -4.14115700 | 0.74279400 | 0.26020200 |
| C | -3.21660100 | -1.43839600 | -0.16140500 |
| C | -5.41955100 | 0.19038800 | 0.31394900 |
| H | -4.02007900 | 1.80947100 | 0.40758100 |
| C | -4.49092500 | -1.98943400 | -0.10871100 |
| H | -2.36856400 | -2.07971300 | -0.37137900 |
| C | -5.60140600 | -1.17785000 | 0.13171800 |
| H | -6.27391500 | 0.83264600 | 0.49745000 |
| H | -4.62165000 | -3.05468700 | -0.26468400 |
| H | -6.59566900 | -1.60825700 | 0.16905400 |
| C | 3.13957400 | -0.05757400 | -0.27304500 |
| C | 4.09467600 | -0.55557400 | -1.20949500 |
| C | 3.57144900 | 0.06252700 | 1.08019700 |
| C | 5.37693600 | -0.89969400 | -0.82275700 |
| H | 3.79652300 | -0.66014900 | -2.24757000 |
| C | 4.85900900 | -0.28503700 | 1.45530100 |
| H | 2.88507300 | 0.43027300 | 1.83284800 |
| C | 5.77461000 | -0.76779800 | 0.51389300 |
| H | 6.07830600 | -1.27422000 | -1.56049700 |
| H | 5.15826000 | -0.18205900 | 2.49284500 |

| | | | |
|---|---|---|---|
| H | 6.77969300 | -1.03794500 | 0.81589700 |
| C | -0.49507100 | -0.10735800 | 0.14970800 |
| H | -0.37980800 | -1.16501700 | 0.35484200 |

| | | |
|---|---|---|
| 7 | 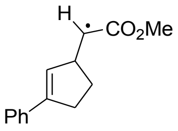 | 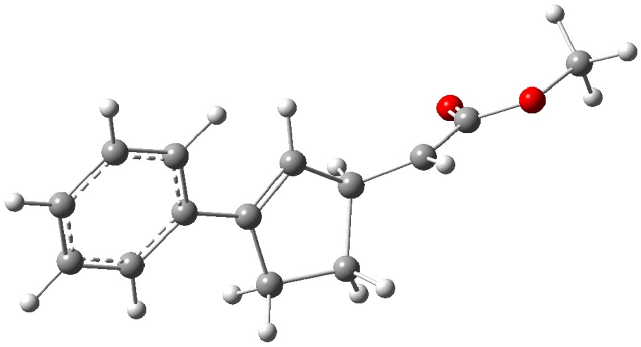 |

Cartesian Coordinates

| | | | |
|---|---|---|---|
| C | -1.15390000 | 0.45640500 | 0.00212500 |
| C | -0.79553000 | 1.91156700 | -0.25233000 |
| C | 0.72860400 | 1.87505500 | -0.48465700 |
| C | 1.22126000 | 0.57838200 | 0.24056300 |
| H | -1.05237100 | 2.52283000 | 0.62244200 |
| H | -1.33380100 | 2.33690000 | -1.10429400 |
| H | 0.93153800 | 1.78445800 | -1.55562400 |
| H | 1.24846300 | 2.76365600 | -0.12315900 |
| H | 1.53738800 | 0.81748400 | 1.26325000 |
| C | 2.36096900 | -0.08059000 | -0.44575500 |
| C | -2.54393000 | -0.03011500 | 0.00891600 |
| C | -3.61558200 | 0.87169700 | 0.10707700 |
| C | -2.84575500 | -1.40039100 | -0.08633100 |
| C | -4.93461900 | 0.42288100 | 0.12911000 |
| H | -3.41862100 | 1.93488800 | 0.17703500 |
| C | -4.16072000 | -1.84822800 | -0.06522900 |
| H | -2.04131200 | -2.11841600 | -0.19483200 |
| C | -5.21409300 | -0.93842400 | 0.04504500 |
| H | -5.74391200 | 1.14001800 | 0.21098700 |
| H | -4.36733400 | -2.90989400 | -0.14397000 |
| H | -6.23987200 | -1.28881300 | 0.05803900 |
| C | 3.67134300 | -0.18685200 | 0.16093300 |
| O | 3.97424100 | 0.22348400 | 1.26895800 |
| O | 4.55816700 | -0.81089600 | -0.66363100 |
| C | 5.88981400 | -0.95932600 | -0.14837400 |
| H | 6.33216700 | 0.01640900 | 0.06077600 |
| H | 5.88349000 | -1.54912700 | 0.77008000 |
| H | 6.44860800 | -1.47266000 | -0.92820200 |
| C | -0.04811300 | -0.25471900 | 0.27549300 |
| H | -0.02983000 | -1.29745500 | 0.56899300 |
| H | 2.22764200 | -0.48502900 | -1.44365500 |

| | | |
|---|---|---|
| 8 | 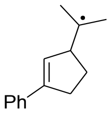 | 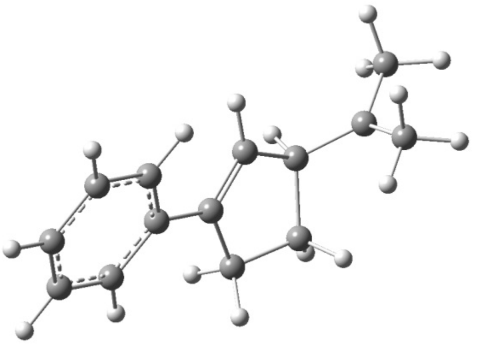 |

Cartesian Coordinates

| | | | |
|---|---|---|---|
| C | 0.21787500 | 0.44499300 | -0.11945200 |
| C | -0.26342700 | 1.84955300 | 0.21149700 |

| | | | |
|---|---|---|---|
| C | -1.79451200 | 1.69092800 | 0.32422800 |
| C | -2.14816800 | 0.40866200 | -0.49637100 |
| H | 0.00832300 | 2.54194700 | -0.59633900 |
| H | 0.18625000 | 2.24486900 | 1.12760600 |
| H | -2.06843500 | 1.53030900 | 1.37020800 |
| H | -2.34603500 | 2.56570800 | -0.02609100 |
| H | -2.39184700 | 0.70268000 | -1.52803600 |
| C | -3.31132200 | -0.38501900 | 0.03688200 |
| C | 1.63802400 | 0.05831000 | -0.06610100 |
| C | 2.64520200 | 1.03608600 | -0.02765900 |
| C | 2.03522200 | -1.29107100 | -0.04956000 |
| C | 3.99304600 | 0.68273700 | 0.00456900 |
| H | 2.37413500 | 2.08516900 | -0.03328200 |
| C | 3.37857400 | -1.64398200 | -0.01728300 |
| H | 1.28118300 | -2.06953000 | -0.04618000 |
| C | 4.36728500 | -0.65819900 | 0.00764900 |
| H | 4.75044400 | 1.45859400 | 0.02821000 |
| H | 3.65777200 | -2.69192100 | -0.00209500 |
| H | 5.41506000 | -0.93501800 | 0.03628400 |
| C | -4.45845300 | -0.73316700 | -0.85640800 |
| C | -3.20172600 | -1.09117900 | 1.35223200 |
| H | -5.38763100 | -0.86165500 | -0.28963200 |
| H | -4.62838900 | 0.03110300 | -1.62103400 |
| H | -4.29287800 | -1.68508600 | -1.39210500 |
| H | -4.13383400 | -1.01779600 | 1.92629900 |
| H | -3.00901500 | -2.16939500 | 1.22260300 |
| H | -2.39036300 | -0.69825500 | 1.96970400 |
| C | -0.81341800 | -0.31779900 | -0.51583100 |
| H | -0.72994900 | -1.33683900 | -0.87647800 |

## 8.3 Bond Dissociation Enthalpies

### DFT Methods:

Structure optimization: (RO)B3P86/6-311G(d,p)

Frequency calculations: (RO)B3P86/6-311G(d,p) scaled by a factor of 0.9806

### Carboxylate Radicals:

| entry | Reaction | BDE (Kcal mol$^{-1}$) |
|---|---|---|
| 1 | 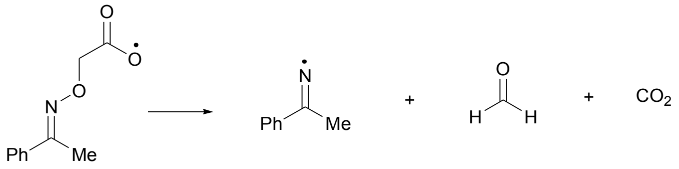 | -36.7 |
| 2 | 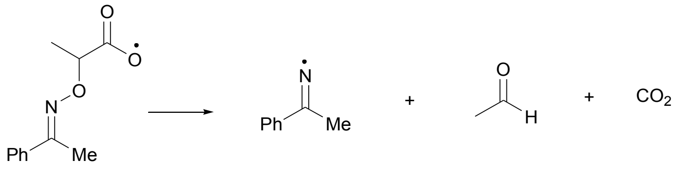 | -43.2 |
| 3 | 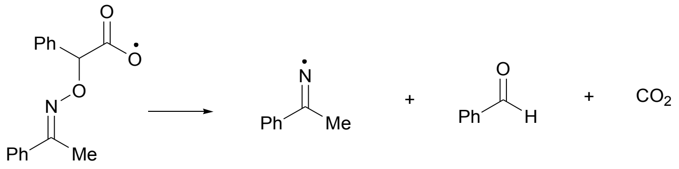 | -49.8 |
| 4 | 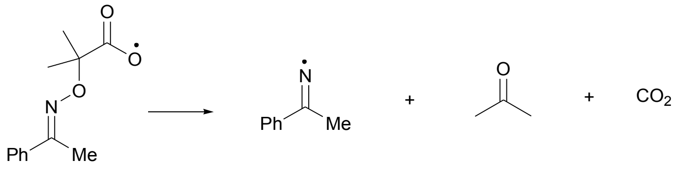 | -47.7 |
| 5 | 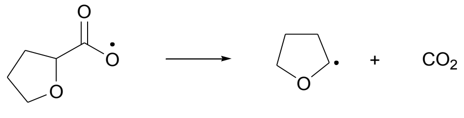 | -22.0 |
| 6 | 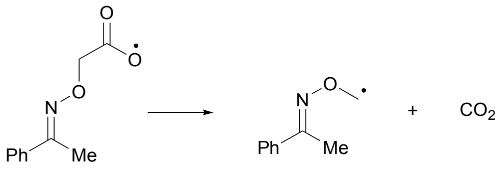 | -19.5 |
| 7 | 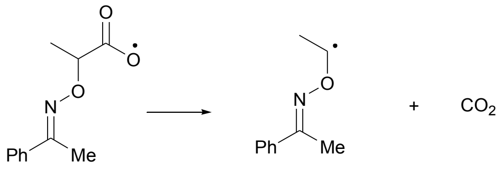 | -20.4 |
| 8 | 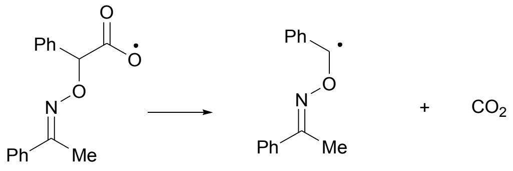 | -30.5 |

| | | |
|---|---|---|
| 9 | 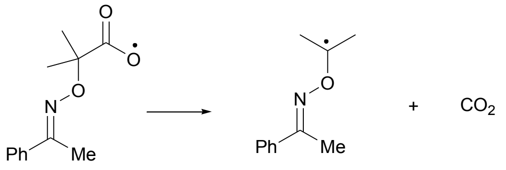 | -21.7 |

## Carbon Radicals

| entry | Reaction | BDE (Kcal mol$^{-1}$) |
|---|---|---|
| 1 | 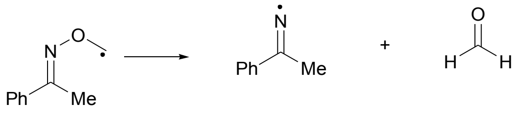 | -17.2 |
| 2 | 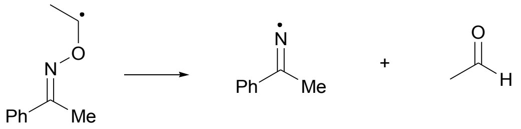 | -22.8 |
| 3 | 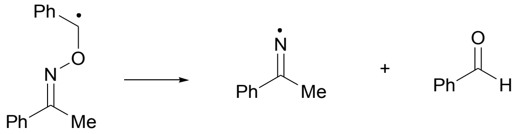 | -19.3 |
| 4 | 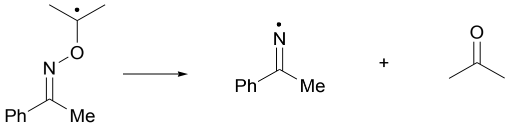 | -25.9 |

## Carboxylic Acids

| entry | Reaction | BDE (Kcal mol$^{-1}$) |
|---|---|---|
| 1 | 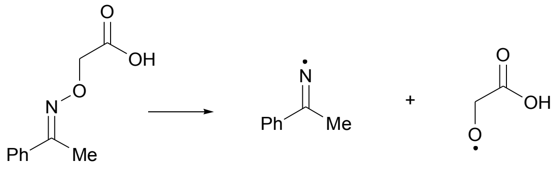 | 52.2 |
| 2 | 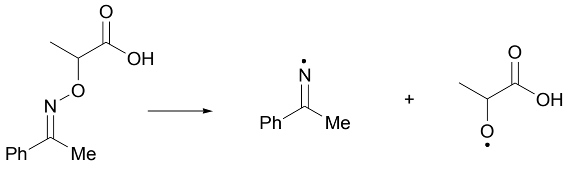 | 46.0 |
| 3 | 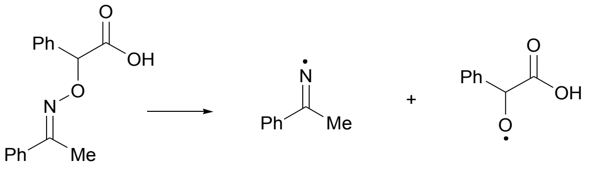 | 46.3 |

| | | |
|---|---|---|
| 4 | 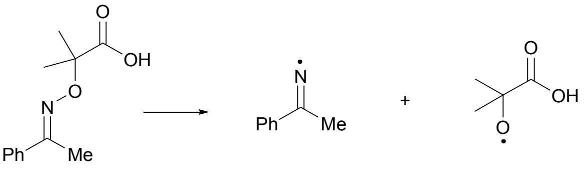 | 52.6 |

## Carboxylate Anions

| entry | Reaction | BDE (Kcal mol$^{-1}$) |
|---|---|---|
| 1 | 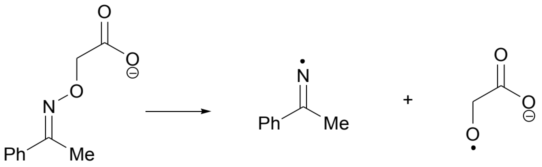 | 39.2 |
| 2 | 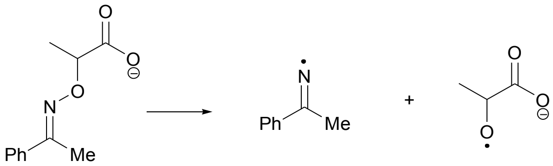 | 36.5 |
| 3 | 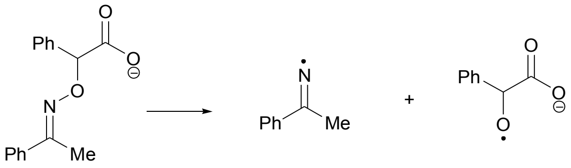 | 22.4 |
| 4 | 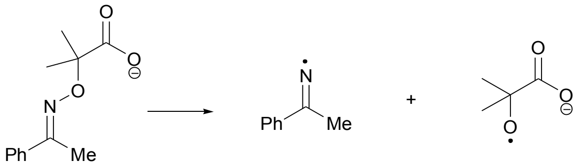 | 36.0 |

**Computed Energies** [values are in Hartree]

| No. | Species | Total Electronic Energy | Sum of Electronic and Zero-point Energies | Sum of Electronic and Thermal Enthalpies | Gibbs Free Energy |
|---|---|---|---|---|---|
| 1 | 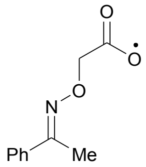 | -669.3622986 | -669.182666 | -669.168285 | -669.226207 |
| 2 | 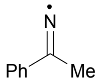 | -365.5781449 | -365.443386 | -365.434429 | -365.477117 |
| 3 | 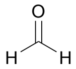 | -114.8154915 | -114.789393 | -114.785578 | -114.810391 |
| 4 | $CO_2$ | -189.0220034 | -189.010387 | -189.006813 | -189.031081 |
| 5 | 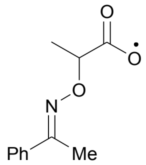 | -708.8382663 | -708.631070 | -708.615226 | -708.675986 |
| 6 | 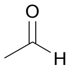 | -154.3020421 | -154.247713 | -154.242846 | -154.272663 |
| 7 | 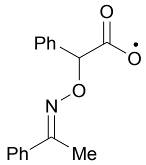 | -901.1845991 | -900.925593 | -900.906482 | -900.976137 |
| 8 | 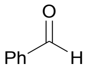 | -346.6596925 | -346.551884 | -346.544539 | -346.582482 |
| 9 | 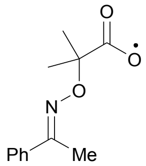 | -748.3139027 | -748.079934 | -748.062527 | -748.126271 |
| 10 | 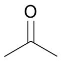 | -193.7845059 | -193.702818 | -193.697244 | -193.729760 |
| 11 | 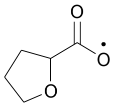 | -421.5476498 | -421.431796 | -421.423343 | -421.465734 |
| 12 | 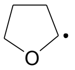 | -232.5585275 | -232.457377 | -232.451530 | -232.485575 |

| | | | | | |
|---|---|---|---|---|---|
| 13 | 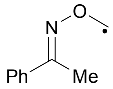 | -480.3692136 | -480.204329 | -480.192558 | -480.242038 |
| 14 | 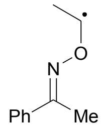 | -519.846638 | -519.654256 | -519.640923 | -519.695002 |
| 15 | 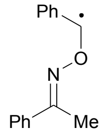 | -712.2096583 | -711.964440 | -711.948240 | -712.010091 |
| 16 | 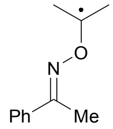 | -559.3249216 | -559.105307 | -559.090359 | -559.148699 |
| 17 | 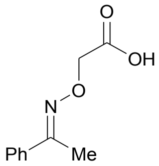 | -670.0652007 | -669.871212 | -669.856939 | -669.913721 |
| 18 | 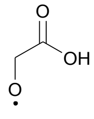 | -304.3972978 | -304.345134 | -304.339288 | -304.373678 |
| 19 | 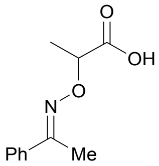 | -709.5303464 | -709.309634 | -709.293688 | -709.354052 |
| 20 | 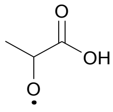 | -343.8735709 | -343.793200 | -343.785934 | -343.823807 |
| 21 | 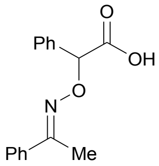 | -901.8809094 | -901.607975 | -901.588956 | -901.656886 |
| 22 | 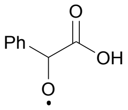 | -536.2231449 | -536.091055 | -536.080680 | -536.127191 |
| 23 | 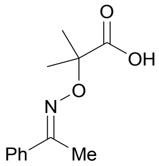 | -749.0174153 | -748.769308 | -748.752179 | -748.813796 |
| 24 | 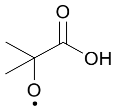 | -383.3498903 | -383.242712 | -383.233900 | -383.275021 |

| | | | | | |
|---|---|---|---|---|---|
| 25 | 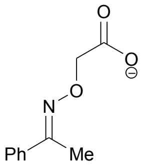 | -669.5060876 | -669.325692 | -669.311790 | -669.367018 |
| 26 | 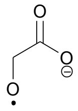 | -303.859446 | -303.821432 | -303.814921 | -303.851628 |
| 27 | 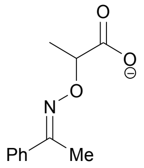 | -708.9794941 | -708.772014 | -708.756408 | -708.815640 |
| 28 | 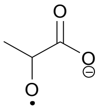 | -343.3373445 | -343.271764 | -343.263820 | -343.304242 |
| 29 | 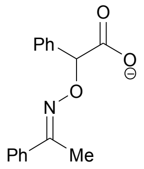 | -901.3347282 | -901.075358 | -901.056680 | -901.123586 |
| 30 | 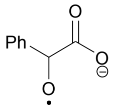 | -535.7144738 | -535.598458 | -535.586495 | -535.638924 |
| 31 | 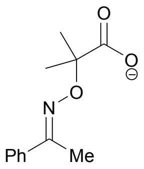 | -748.455346 | -748.221144 | -748.204048 | -748.265966 |
| 32 | 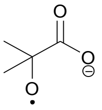 | -382.8146061 | -382.721626 | -382.712278 | -382.755220 |

## Optimized Structures and Cartesian Coordinates

| No. | Species | Optimized Structure |
|---|---|---|
| 1 | 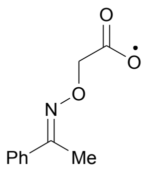 | 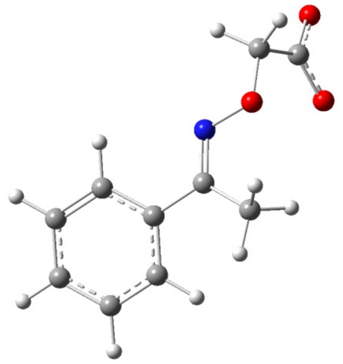 |



Cartesian Coordinates

| | | | |
|---|---|---|---|
| N | -0.63769800 | 0.08557600 | -0.65591500 |
| C | 0.28803900 | -0.76868800 | -0.40803000 |
| O | -1.84375800 | -0.55022100 | -0.95602300 |
| C | 0.03050800 | -2.24258900 | -0.38380600 |
| H | 0.94297000 | -2.81736100 | -0.24464600 |
| H | -0.68113300 | -2.48270500 | 0.41089800 |
| H | -0.43776400 | -2.54691400 | -1.32219900 |
| C | 1.62598400 | -0.20398100 | -0.14223900 |
| C | 1.96575100 | 1.06881700 | -0.61771600 |
| C | 2.56970700 | -0.91307400 | 0.60621300 |
| C | 3.21145900 | 1.61198300 | -0.35072200 |
| H | 1.23851000 | 1.61743500 | -1.20272900 |
| C | 3.81626200 | -0.36370400 | 0.87671300 |
| H | 2.32672700 | -1.89142600 | 1.00319300 |
| C | 4.14304900 | 0.89785100 | 0.39730200 |
| H | 3.46125500 | 2.59544500 | -0.73274100 |
| H | 4.53276100 | -0.92444400 | 1.46618900 |
| H | 5.11896300 | 1.32288500 | 0.60292000 |
| C | -2.88416400 | 0.39233100 | -0.91693600 |
| H | -2.53986300 | 1.37688300 | -1.24282200 |
| H | -3.67068900 | 0.04259200 | -1.58993300 |
| C | -3.44039500 | 0.47347100 | 0.48013000 |
| O | -3.07042600 | -0.31221600 | 1.38463700 |
| O | -4.30894800 | 1.28669700 | 0.84611400 |

| No. | Species | Optimized Structure |
|---|---|---|
| 2 | 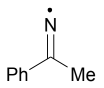 | 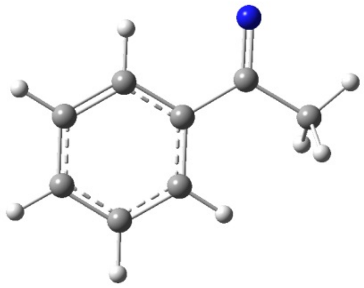 |



Cartesian Coordinates

| | | | |
|---|---|---|---|
| N | -2.26144400 | 1.37623300 | -0.00039600 |
| C | -1.71562800 | 0.24660400 | -0.00009700 |
| C | -2.59621800 | -0.98422400 | 0.00036800 |
| H | -2.39828800 | -1.59147500 | 0.88715700 |
| H | -3.64370100 | -0.68717800 | 0.00052100 |
| H | -2.39864300 | -1.59176800 | -0.88631600 |
| C | -0.23282100 | 0.09493500 | -0.00010400 |
| C | 0.58049200 | 1.23343200 | 0.00015100 |
| C | 0.36667400 | -1.16480900 | -0.00020200 |
| C | 1.95956000 | 1.11046500 | 0.00019900 |

| | | | |
|---|---|---|---|
| H | 0.11325000 | 2.21133000 | 0.00035700 |
| C | 1.75171400 | -1.28560500 | -0.00015400 |
| H | -0.24314400 | -2.06043400 | -0.00026600 |
| C | 2.55058100 | -0.15054100 | 0.00001800 |
| H | 2.57904400 | 2.00025100 | 0.00041500 |
| H | 2.20485400 | -2.27051900 | -0.00019000 |
| H | 3.63060900 | -0.24538900 | 0.00002400 |

| | | |
|---|---|---|
| 3 | 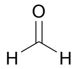 | 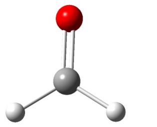 |



Cartesian Coordinates

| | | | |
|---|---|---|---|
| C | 0.00000000 | 0.00000000 | -0.52492900 |
| H | 0.00000000 | 0.93917700 | -1.11631100 |
| H | 0.00000000 | -0.93917700 | -1.11631100 |
| O | 0.00000000 | 0.00000000 | 0.67277500 |

| | | |
|---|---|---|
| 4 | $CO_2$ | 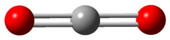 |

Cartesian Coordinates

| | | | |
|---|---|---|---|
| C | 0.00000000 | 0.00000000 | 0.00000000 |
| O | 0.00000000 | 0.00000000 | 1.15823300 |
| O | 0.00000000 | 0.00000000 | -1.15823300 |

| | | |
|---|---|---|
| 5 | 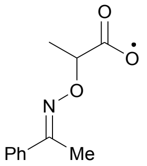 | 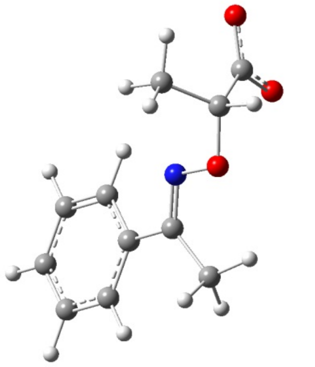 |



Cartesian Coordinates

| | | | |
|---|---|---|---|
| N | -0.54196600 | 0.40272300 | 0.20888100 |
| C | 0.52960600 | 1.01721500 | -0.13172100 |
| O | -1.67777800 | 1.20322200 | 0.08986900 |
| C | 0.56552100 | 2.44228900 | -0.59075000 |
| H | 1.32009100 | 2.57868600 | -1.36613100 |
| H | -0.40775800 | 2.74023700 | -0.97416000 |
| H | 0.81406000 | 3.10663000 | 0.24325400 |
| C | 1.77842900 | 0.23147700 | -0.04176800 |
| C | 3.01725600 | 0.87447600 | 0.02521200 |
| C | 1.74540200 | -1.16793200 | -0.01333100 |
| C | 4.19212900 | 0.13990100 | 0.12839200 |
| H | 3.06960700 | 1.95693700 | 0.00930200 |
| C | 2.91845700 | -1.89754600 | 0.08540200 |
| H | 0.78812600 | -1.66882300 | -0.08302600 |
| C | 4.14738700 | -1.24733700 | 0.15828300 |
| H | 5.14418400 | 0.65551700 | 0.18528200 |
| H | 2.87637300 | -2.98094200 | 0.09731400 |
| H | 5.06442700 | -1.82092400 | 0.23225800 |
| C | -2.81889200 | 0.45078900 | 0.45879900 |
| H | -3.64658200 | 1.15283900 | 0.30715500 |
| C | -3.04448700 | -0.66355700 | -0.53638300 |
| O | -2.57635800 | -0.65129800 | -1.70143600 |
| O | -3.75780000 | -1.66198500 | -0.31951100 |
| C | -2.81220500 | -0.04178400 | 1.89148000 |
| H | -2.77445100 | 0.80733300 | 2.57557200 |
| H | -3.72216900 | -0.61251100 | 2.08963100 |

| | | | |
|---|---|---|---|
| H | -1.94827900 | -0.68150300 | 2.06831500 |

| | | |
|---|---|---|
| 6 | 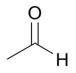 | 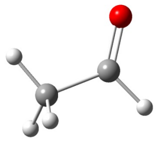 |



Cartesian Coordinates

| | | | |
|---|---|---|---|
| C | 0.23281300 | 0.39618300 | 0.00004700 |
| H | 0.30188100 | 1.50747700 | -0.00007600 |
| C | -1.16354200 | -0.14820200 | -0.00001700 |
| O | 1.22963500 | -0.27532100 | -0.00000600 |
| H | -1.14939900 | -1.23750700 | -0.00088100 |
| H | -1.70243100 | 0.22162000 | 0.87848000 |
| H | -1.70275100 | 0.22309600 | -0.87765900 |

| | | |
|---|---|---|
| 7 | 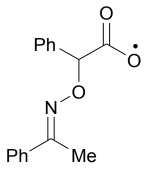 | 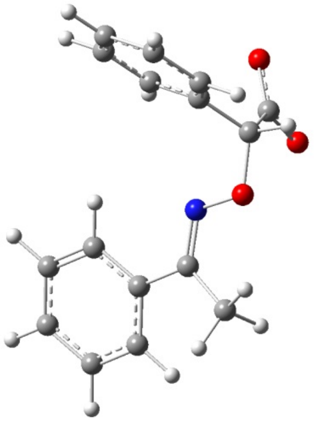 |



Cartesian Coordinates

| | | | |
|---|---|---|---|
| N | 0.36875600 | -0.66437500 | -0.26996700 |
| C | 1.50243400 | -0.77710900 | -0.85739500 |
| O | -0.60781000 | -1.47069700 | -0.85114000 |
| C | 1.74836800 | -1.68340000 | -2.02397400 |
| H | 2.48117200 | -1.25051600 | -2.70544600 |
| H | 2.13190400 | -2.64906300 | -1.67889600 |
| H | 0.82004000 | -1.87165100 | -2.55891900 |
| C | 2.60230400 | 0.04063500 | -0.30445800 |
| C | 2.33624000 | 1.15398000 | 0.50159900 |
| C | 3.93368700 | -0.29106500 | -0.56933300 |
| C | 3.37276200 | 1.90688300 | 1.02744400 |
| H | 1.30554900 | 1.42093900 | 0.69755700 |
| C | 4.97187800 | 0.46306900 | -0.03668900 |
| H | 4.16678800 | -1.15309500 | -1.18332600 |
| C | 4.69592000 | 1.56469800 | 0.76167000 |
| H | 3.14978700 | 2.77016000 | 1.64454700 |
| H | 5.99885100 | 0.18625800 | -0.24681100 |
| H | 5.50563800 | 2.15659900 | 1.17326700 |
| C | -1.83416300 | -1.30841200 | -0.15308700 |
| H | -2.49997700 | -2.01138900 | -0.66533500 |
| C | -1.69221400 | -1.84617200 | 1.25602500 |
| O | -0.90659000 | -2.76808100 | 1.55013100 |
| O | -2.40059100 | -1.45782800 | 2.21163000 |
| C | -2.43836700 | 0.06625600 | -0.21998900 |
| C | -3.38123600 | 0.34451500 | -1.20948100 |
| C | -2.06593600 | 1.07831500 | 0.66874500 |
| C | -3.94074300 | 1.60945700 | -1.31823100 |
| H | -3.67601900 | -0.43803600 | -1.90144700 |
| C | -2.62923200 | 2.34304800 | 0.56244200 |
| H | -1.33475500 | 0.87192600 | 1.43938700 |
| C | -3.56452600 | 2.61144800 | -0.42982300 |
| H | -4.67234200 | 1.81348700 | -2.09169000 |
| H | -2.33800400 | 3.12112600 | 1.25877500 |
| H | -4.00305500 | 3.59985000 | -0.50966400 |

| | | |
|---|---|---|
| 8 | 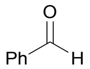 | 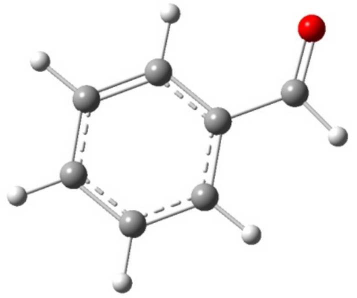 |

Cartesian Coordinates

| | | | |
|---|---|---|---|
| C | 1.98468900 | 0.46570300 | 0.00000500 |
| H | 2.26483400 | 1.54198300 | -0.00001200 |
| C | 0.53081500 | 0.21333000 | 0.00001600 |
| C | 0.04570800 | -1.09643400 | 0.00001000 |
| C | -0.35886000 | 1.28666600 | 0.00001300 |
| C | -1.31968000 | -1.32561000 | 0.00000100 |
| H | 0.76112400 | -1.91099300 | 0.00002500 |
| C | -1.72808000 | 1.05615800 | -0.00001300 |
| H | 0.02780400 | 2.30164800 | 0.00003900 |
| C | -2.20615200 | -0.24948900 | -0.00000600 |
| H | -1.70106100 | -2.34041900 | 0.00001800 |
| H | -2.42171500 | 1.88911000 | -0.00002600 |
| H | -3.27518700 | -0.43174500 | -0.00003900 |
| O | 2.83169500 | -0.39394100 | -0.00002000 |

| | | |
|---|---|---|
| 9 | 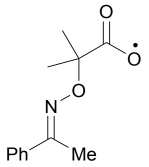 | 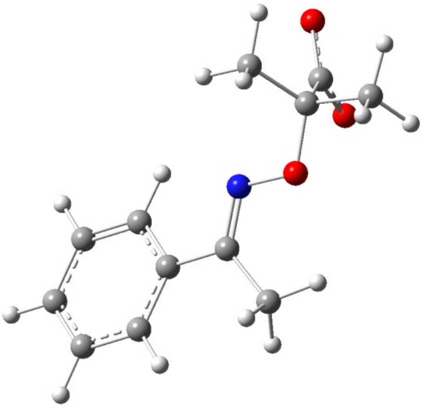 |

Cartesian Coordinates

| | | | |
|---|---|---|---|
| N | 0.29397500 | 0.28657200 | -0.18591800 |
| C | -0.74125700 | 0.96455100 | 0.14724200 |
| O | 1.47520600 | 1.00963700 | -0.05403900 |
| C | -0.69637900 | 2.38700400 | 0.61400200 |
| H | -0.93344400 | 3.06782100 | -0.21000900 |
| H | -1.42631300 | 2.55454400 | 1.40706500 |
| H | 0.29817600 | 2.63499300 | 0.97692500 |
| C | -2.03622100 | 0.25936700 | 0.04025700 |
| C | -2.09204400 | -1.13858400 | -0.01784700 |
| C | -3.23208400 | 0.98045400 | -0.01228400 |
| C | -3.30899400 | -1.78988500 | -0.13106400 |
| H | -1.16874700 | -1.70088900 | 0.03993900 |
| C | -4.45125800 | 0.32445600 | -0.12979300 |
| H | -3.21538400 | 2.06332200 | 0.02692800 |
| C | -4.49439700 | -1.06183000 | -0.18908100 |
| H | -3.33566200 | -2.87328500 | -0.16614400 |
| H | -5.36869900 | 0.90047800 | -0.17475900 |
| H | -5.44577500 | -1.57471300 | -0.27456200 |
| C | 2.59085200 | 0.19653500 | -0.41783800 |
| C | 2.67464800 | -0.95692600 | 0.56351100 |
| O | 2.24188600 | -0.88710600 | 1.74076500 |
| O | 3.23620200 | -2.04428300 | 0.32704500 |

| | | | |
|---|---|---|---|
| C | 2.50410100 | -0.30892900 | -1.84627900 |
| C | 3.80712500 | 1.08763300 | -0.19060900 |
| H | 2.54498600 | 0.53595000 | -2.53690100 |
| H | 3.34213900 | -0.97690600 | -2.05700600 |
| H | 1.57294600 | -0.85231800 | -2.00174400 |
| H | 3.74425500 | 1.95327200 | -0.85234300 |
| H | 3.83904000 | 1.43442700 | 0.84335300 |
| H | 4.72376200 | 0.53822600 | -0.41078600 |
| 10 | 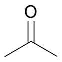 | 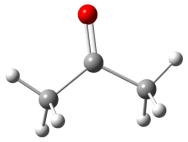 | |
| Cartesian Coordinates | | | |
| C | -0.00000600 | 0.18523200 | 0.00000000 |
| C | -0.00000600 | -0.61134000 | 1.28388100 |
| C | -0.00000600 | -0.61134000 | -1.28388100 |
| O | 0.00000800 | 1.39266600 | 0.00000000 |
| H | -0.87897800 | -1.26149700 | -1.32867600 |
| H | 0.00058300 | 0.06548400 | -2.13663200 |
| H | 0.87841500 | -1.26230900 | -1.32815400 |
| H | 0.00058300 | 0.06548400 | 2.13663200 |
| H | -0.87897800 | -1.26149700 | 1.32867600 |
| H | 0.87841500 | -1.26230900 | 1.32815400 |
| 11 | 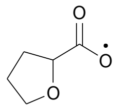 | 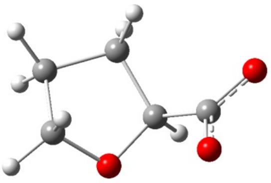 | |
| Cartesian Coordinates | | | |
| C | 1.58587000 | -0.90885400 | -0.36651700 |
| O | 0.64089400 | -0.98325600 | 0.69866200 |
| C | -0.08712600 | 0.22625700 | 0.70738600 |
| C | 0.76171200 | 1.29640300 | -0.02170700 |
| C | 2.05406600 | 0.53785300 | -0.35161800 |
| H | 2.36761000 | -1.63927500 | -0.15881600 |
| H | 1.10590700 | -1.16553000 | -1.31989400 |
| H | -0.28977600 | 0.49466100 | 1.74830000 |
| H | 0.25420400 | 1.63731000 | -0.92643200 |
| H | 0.93351400 | 2.17014600 | 0.60612200 |
| H | 2.49700000 | 0.85414900 | -1.29725600 |
| H | 2.79369600 | 0.67826300 | 0.43984200 |
| C | -1.41366000 | -0.00025400 | 0.02798200 |
| O | -1.75945000 | -1.13707800 | -0.38922800 |
| O | -2.26486100 | 0.87806600 | -0.19256100 |
| 12 | 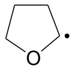 | 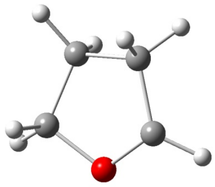 | |
| Cartesian Coordinates | | | |
| C | 1.09674800 | 0.46865300 | -0.17968400 |
| O | 0.85165500 | -0.90688800 | 0.12381500 |
| C | -0.48572300 | -1.13700700 | -0.04649800 |
| C | -1.25731000 | 0.14226300 | -0.13747300 |

| | | | |
|---|---|---|---|
| C | -0.18606700 | 1.18775700 | 0.21720000 |
| H | 1.98154500 | 0.77790100 | 0.37676500 |
| H | 1.29622500 | 0.56991900 | -1.25406100 |
| H | -0.83020200 | -2.06702200 | 0.38475000 |
| H | -1.64725300 | 0.31268700 | -1.15044900 |
| H | -2.11732500 | 0.17426600 | 0.53876800 |
| H | -0.31497600 | 2.13920700 | -0.30111800 |
| H | -0.18713500 | 1.37815300 | 1.29354700 |

13

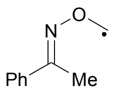


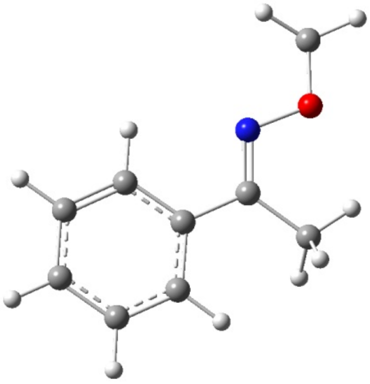

Cartesian Coordinates

| | | | |
|---|---|---|---|
| N | 1.65974500 | -0.45828800 | -0.01021800 |
| C | 0.82722000 | 0.52086000 | 0.00648100 |
| O | 2.98004800 | -0.04309500 | -0.01716800 |
| C | 1.22071900 | 1.96523500 | 0.02644400 |
| H | 0.78812100 | 2.46402300 | 0.89764700 |
| H | 2.30086600 | 2.07216900 | 0.05860600 |
| H | 0.84218900 | 2.47419800 | -0.86434900 |
| C | -0.60277800 | 0.14685800 | 0.00317300 |
| C | -0.99807000 | -1.19824100 | 0.01884300 |
| C | -1.59541000 | 1.13110800 | -0.01624400 |
| C | -2.33915900 | -1.53934500 | 0.01468700 |
| H | -0.23422700 | -1.96502300 | 0.03511500 |
| C | -2.94164000 | 0.78521300 | -0.02070600 |
| H | -1.32283600 | 2.17938700 | -0.02939000 |
| C | -3.31942700 | -0.54968900 | -0.00529000 |
| H | -2.62555900 | -2.58518300 | 0.02786300 |
| H | -3.69502500 | 1.56480900 | -0.03631700 |
| H | -4.36921400 | -0.82064800 | -0.00824800 |
| C | 3.84128100 | -1.08916600 | 0.04614800 |
| H | 3.44024000 | -2.07186000 | -0.15718300 |
| H | 4.86042700 | -0.79609500 | -0.15608700 |

14

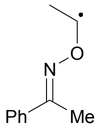


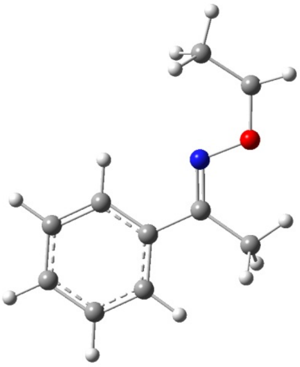

Cartesian Coordinates

| | | | |
|---|---|---|---|
| N | -1.35842100 | 0.01028700 | -0.01814800 |
| C | -0.37065000 | 0.83705300 | -0.01042400 |
| O | -2.58392000 | 0.64000100 | -0.00837000 |
| C | -0.51608000 | 2.32729700 | -0.00130000 |
| H | 0.00701600 | 2.76637300 | -0.85502000 |
| H | -1.56257100 | 2.61447300 | -0.04435900 |

| | | | |
|---|---|---|---|
| H | -0.07283100 | 2.74870800 | 0.90557000 |
| C | 0.97461200 | 0.22957800 | -0.00654100 |
| C | 1.14070500 | -1.16292200 | -0.03586500 |
| C | 2.11965200 | 1.03208600 | 0.02865200 |
| C | 2.40524800 | -1.72470600 | -0.03099700 |
| H | 0.25937800 | -1.79077900 | -0.06227900 |
| C | 3.38843600 | 0.46463700 | 0.03404300 |
| H | 2.02767000 | 2.11112100 | 0.05336500 |
| C | 3.53806700 | -0.91444700 | 0.00396800 |
| H | 2.51168100 | -2.80371400 | -0.05470400 |
| H | 4.26175000 | 1.10678800 | 0.06202600 |
| H | 4.52755800 | -1.35750500 | 0.00782800 |
| C | -3.64764300 | -0.21052000 | -0.10114500 |
| H | -4.56236800 | 0.32876600 | 0.10216400 |
| C | -3.50184300 | -1.66798200 | 0.07282200 |
| H | -3.05687100 | -1.93194000 | 1.04386700 |
| H | -4.48674100 | -2.13429700 | 0.00847300 |
| H | -2.85638500 | -2.11045800 | -0.69220800 |

| | | |
|---|---|---|
| 15 | 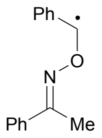 | 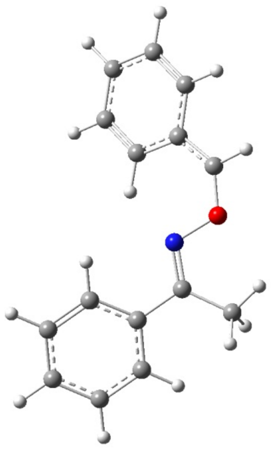 |

Cartesian Coordinates

| | | | |
|---|---|---|---|
| N | -0.18956000 | 0.88764300 | -0.23173700 |
| C | -1.40010900 | 1.25439200 | 0.00295600 |
| O | 0.69712500 | 1.95424000 | -0.18804700 |
| C | -1.81544200 | 2.65598400 | 0.32694600 |
| H | -2.73200700 | 2.91580000 | -0.20542500 |
| H | -2.01455700 | 2.75394000 | 1.39900100 |
| H | -1.03200100 | 3.36192400 | 0.06479500 |
| C | -2.40952900 | 0.17829400 | -0.05376600 |
| C | -2.11763900 | -1.05168400 | -0.65917700 |
| C | -3.67843000 | 0.36486000 | 0.50286300 |
| C | -3.06510700 | -2.06073200 | -0.69566600 |
| H | -1.14161700 | -1.19574900 | -1.10563800 |
| C | -4.62583100 | -0.65064900 | 0.46608400 |
| H | -3.92979700 | 1.30457600 | 0.98011700 |
| C | -4.32376600 | -1.86625100 | -0.13249900 |
| H | -2.82457000 | -3.00416700 | -1.17297600 |
| H | -5.60270100 | -0.48887900 | 0.90776600 |
| H | -5.06456900 | -2.65713500 | -0.16551500 |
| C | 2.00919000 | 1.65190600 | -0.32397800 |
| H | 2.55125100 | 2.54898600 | -0.59235500 |
| C | 2.70426700 | 0.45059700 | -0.08258000 |
| C | 2.15660600 | -0.76350700 | 0.40371700 |
| C | 4.10357800 | 0.47954400 | -0.33061800 |
| C | 2.96921200 | -1.86037900 | 0.62577900 |
| H | 1.09642900 | -0.82318400 | 0.59754400 |
| C | 4.89607700 | -0.62636600 | -0.10870200 |
| H | 4.55105100 | 1.39586900 | -0.70243800 |
| C | 4.33802000 | -1.81154900 | 0.37224900 |
| H | 2.52494700 | -2.77520400 | 1.00373900 |
| H | 5.96044500 | -0.56995300 | -0.30983600 |
| H | 4.96103600 | -2.68099900 | 0.54611100 |

| | | |
|---|---|---|
| 16 | 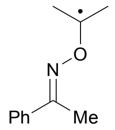 | 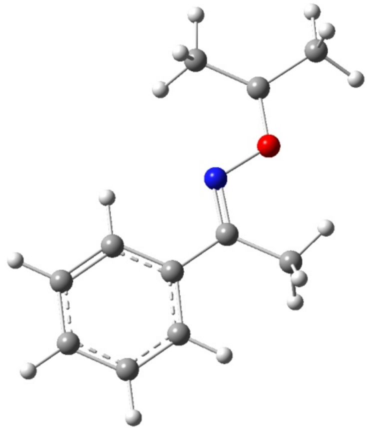 |

Cartesian Coordinates

| | | | |
|---|---|---|---|
| N | -0.92941500 | -0.07307300 | -0.04956100 |
| C | 0.02693900 | 0.78861100 | -0.00949500 |
| O | -2.17708400 | 0.50883300 | -0.04394600 |
| C | -0.17236600 | 2.27233900 | 0.02604900 |
| H | 0.26147400 | 2.73717400 | -0.86413200 |
| H | -1.22907100 | 2.51950800 | 0.06694700 |
| H | 0.32797700 | 2.70433300 | 0.89684800 |
| C | 1.39383400 | 0.23120900 | 0.00271600 |
| C | 1.61178700 | -1.15354200 | 0.05278500 |
| C | 2.50861500 | 1.07515700 | -0.03583300 |
| C | 2.89636600 | -1.66797400 | 0.06087500 |
| H | 0.75434200 | -1.81328600 | 0.08782800 |
| C | 3.79761200 | 0.55532700 | -0.02773100 |
| H | 2.37667900 | 2.14962600 | -0.07512600 |
| C | 3.99851800 | -0.81677000 | 0.02042800 |
| H | 3.04259000 | -2.74184900 | 0.10104800 |
| H | 4.64656300 | 1.22924700 | -0.05941300 |
| H | 5.00385200 | -1.22256200 | 0.02779900 |
| C | -3.22278900 | -0.37209200 | -0.20198200 |
| C | -2.98214200 | -1.82538200 | -0.05688100 |
| H | -2.62034100 | -2.08671700 | 0.95055000 |
| H | -3.91571900 | -2.36343500 | -0.23175800 |
| H | -2.22843000 | -2.18706300 | -0.75985300 |
| C | -4.51117800 | 0.27509100 | 0.14404800 |
| H | -4.55411000 | 1.29277600 | -0.25222900 |
| H | -5.34623800 | -0.29126400 | -0.27345200 |
| H | -4.66816300 | 0.34251400 | 1.23357200 |

| | | |
|---|---|---|
| 17 | 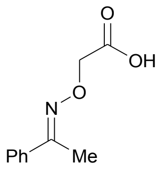 | 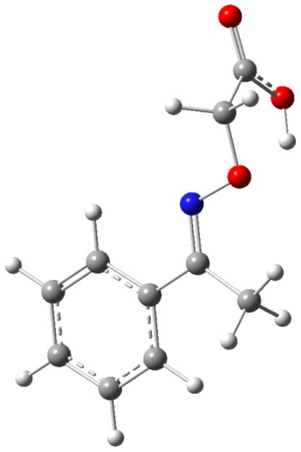 |

Cartesian Coordinates

| | | | |
|---|---|---|---|
| N | 0.65353600 | 0.23734600 | -0.40396500 |
| C | -0.38934100 | 0.94624400 | -0.16948000 |
| O | 1.82985300 | 0.99883100 | -0.46400700 |
| C | -0.37242800 | 2.43322500 | 0.00664100 |

| | | | |
|---|---|---|---|
| H | -1.14530800 | 2.89587900 | -0.61039600 |
| H | -0.58118000 | 2.69816800 | 1.04755500 |
| H | 0.59593300 | 2.84255700 | -0.26787300 |
| C | -1.66612000 | 0.20350800 | -0.07495900 |
| C | -1.72456700 | -1.16766300 | -0.35768200 |
| C | -2.84035400 | 0.85787500 | 0.30611400 |
| C | -2.92206400 | -1.85508900 | -0.26379300 |
| H | -0.81730700 | -1.68037300 | -0.65048800 |
| C | -4.04092100 | 0.16422500 | 0.40187500 |
| H | -2.82576600 | 1.91607000 | 0.53728500 |
| C | -4.08688800 | -1.19272000 | 0.11618400 |
| H | -2.94980500 | -2.91530700 | -0.48882600 |
| H | -4.94096600 | 0.68909500 | 0.70132400 |
| H | -5.02334300 | -1.73415600 | 0.18835600 |
| C | 2.89271200 | 0.16270200 | -0.89429700 |
| H | 2.56240200 | -0.51470300 | -1.68197900 |
| H | 3.65841400 | 0.83078000 | -1.29414200 |
| C | 3.51596300 | -0.65168800 | 0.23163700 |
| O | 3.12448700 | -0.30246700 | 1.46244200 |
| O | 4.32455300 | -1.50813200 | 0.02234600 |
| H | 2.46508400 | 0.40099200 | 1.36725400 |

18

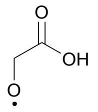


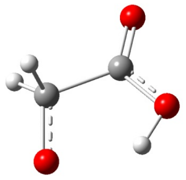

Cartesian Coordinates

| | | | |
|---|---|---|---|
| O | 1.77435300 | 0.10770300 | -0.17315100 |
| C | 0.77689500 | -0.73264100 | 0.13628900 |
| H | 0.77950300 | -1.69708200 | -0.38798800 |
| H | 0.82090900 | -0.93854000 | 1.22631800 |
| C | -0.61159100 | -0.04061600 | 0.00599200 |
| O | -0.53007300 | 1.28069500 | 0.05660300 |
| O | -1.62124500 | -0.66538500 | -0.10404800 |
| H | 0.42348600 | 1.49106200 | 0.07275100 |

19

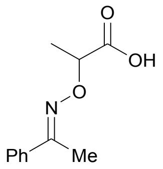


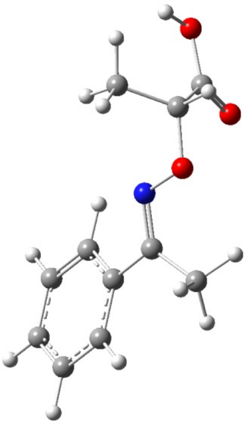

Cartesian Coordinates

| | | | |
|---|---|---|---|
| N | -0.48201100 | 0.40365200 | 0.28355000 |
| C | 0.57670400 | 1.00766400 | -0.11047800 |
| O | -1.60876900 | 1.21716900 | 0.18915700 |
| C | 0.57540000 | 2.41542400 | -0.62116400 |
| H | 1.44732500 | 2.61144400 | -1.24363200 |
| H | -0.33158900 | 2.58658300 | -1.20108700 |
| H | 0.56853800 | 3.13125300 | 0.20699700 |
| C | 1.82848000 | 0.22605700 | -0.03747500 |
| C | 3.06430200 | 0.86860400 | 0.07223100 |
| C | 1.79870800 | -1.17298200 | -0.06545400 |

| | | | |
|---|---|---|---|
| C | 4.23955800 | 0.13315300 | 0.16372900 |
| H | 3.11168300 | 1.95124800 | 0.10257700 |
| C | 2.97251600 | -1.90381800 | 0.02040900 |
| H | 0.84294700 | -1.67045100 | -0.17203300 |
| C | 4.19833600 | -1.25452600 | 0.13717900 |
| H | 5.18935200 | 0.64805500 | 0.25570200 |
| H | 2.93367900 | -2.98697000 | -0.01376700 |
| H | 5.11599000 | -1.82850800 | 0.20053800 |
| C | -2.76054200 | 0.45268800 | 0.46439500 |
| H | -3.57487200 | 1.18130800 | 0.37722400 |
| C | -2.99501700 | -0.57484600 | -0.65245400 |
| O | -2.44631200 | -0.54961300 | -1.71020000 |
| O | -3.93835800 | -1.50656300 | -0.38263300 |
| C | -2.74927700 | -0.12472100 | 1.87612200 |
| H | -2.49315000 | 0.66959900 | 2.57890100 |
| H | -3.73247700 | -0.50441600 | 2.17401900 |
| H | -2.01087000 | -0.92018700 | 1.96930400 |
| H | -4.23998100 | -1.41865200 | 0.52756800 |

| | | |
|---|---|---|
| 20 | 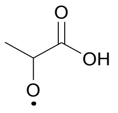 | 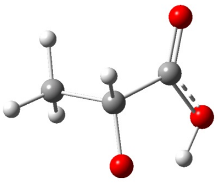 |

Cartesian Coordinates

| | | | |
|---|---|---|---|
| O | -1.16812600 | 1.17163800 | -0.42651100 |
| C | -0.67379000 | -0.07847100 | -0.48458700 |
| H | -0.79211700 | -0.55696600 | -1.46718300 |
| C | 0.82681900 | -0.09407500 | -0.08820800 |
| O | 1.54284300 | -1.01403100 | -0.35018900 |
| O | 1.20054600 | 0.98864500 | 0.58186500 |
| C | -1.40616400 | -0.94889700 | 0.57397900 |
| H | -2.46704800 | -0.99185400 | 0.33097800 |
| H | -0.97006100 | -1.94794200 | 0.53513400 |
| H | -1.27629600 | -0.52389000 | 1.56968700 |
| H | 0.42222600 | 1.57929100 | 0.58295500 |

| | | |
|---|---|---|
| 21 | 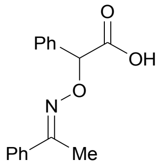 | 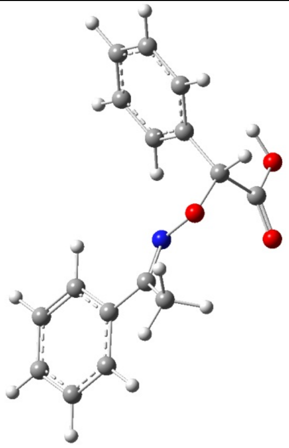 |

Cartesian Coordinates

| | | | |
|---|---|---|---|
| N | 0.40049600 | -0.54205900 | -0.35826600 |
| C | 1.56485500 | -0.78705800 | -0.83414500 |
| O | -0.56449000 | -1.35130200 | -0.95429900 |
| C | 1.81055800 | -1.86027200 | -1.84873800 |
| H | 2.83665300 | -1.85235900 | -2.21037500 |
| H | 1.57799100 | -2.83796400 | -1.41791900 |
| H | 1.13541000 | -1.72426900 | -2.69618100 |
| C | 2.66363000 | 0.05239900 | -0.31513100 |
| C | 2.41028400 | 1.34939800 | 0.14520200 |
| C | 3.97182100 | -0.43546200 | -0.25604800 |

| | | | |
|---|---|---|---|
| C | 3.43583500 | 2.13050800 | 0.65390500 |
| H | 1.40010000 | 1.73467700 | 0.08340700 |
| C | 4.99712300 | 0.34718800 | 0.25903800 |
| H | 4.19050600 | -1.44260200 | -0.59173600 |
| C | 4.73404400 | 1.63283400 | 0.71362400 |
| H | 3.22476800 | 3.13701100 | 0.99802400 |
| H | 6.00429100 | -0.05144700 | 0.30645800 |
| H | 5.53603200 | 2.24634700 | 1.10845500 |
| C | -1.77312300 | -1.27980300 | -0.22704100 |
| H | -2.42748400 | -1.96788400 | -0.77374900 |
| C | -1.60054400 | -1.92098000 | 1.16265500 |
| O | -0.72377200 | -2.68439500 | 1.42929500 |
| O | -2.57564900 | -1.62873800 | 2.04479300 |
| C | -2.42596400 | 0.08357000 | -0.22590500 |
| C | -3.49030900 | 0.33402600 | -1.09281500 |
| C | -1.97725700 | 1.11254600 | 0.60892100 |
| C | -4.09630300 | 1.58355100 | -1.13263900 |
| H | -3.84210300 | -0.45708100 | -1.74760900 |
| C | -2.58244400 | 2.36190300 | 0.56740200 |
| H | -1.14307100 | 0.93003000 | 1.27502100 |
| C | -3.64134700 | 2.60015700 | -0.30217000 |
| H | -4.92198600 | 1.76248000 | -1.81193600 |
| H | -2.22525500 | 3.15310800 | 1.21689100 |
| H | -4.11191300 | 3.57650500 | -0.33051100 |
| H | -3.13127900 | -0.92370000 | 1.68460700 |

| | | |
|---|---|---|
| 22 | 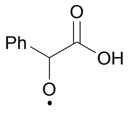 | 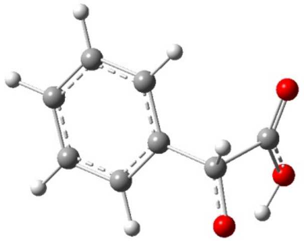 |

Cartesian Coordinates

| | | | |
|---|---|---|---|
| O | -1.61162500 | -1.51034700 | -0.85994500 |
| C | -1.11375200 | -0.29149600 | -0.89202300 |
| H | -1.30518000 | 0.28506600 | -1.80830000 |
| C | -1.93624100 | 0.49856600 | 0.28276500 |
| O | -2.10047700 | 1.67258000 | 0.22756000 |
| O | -2.33149500 | -0.31172000 | 1.23428500 |
| C | 0.32896800 | -0.12915900 | -0.42020300 |
| C | 0.95146600 | 1.11378600 | -0.51284700 |
| C | 1.02726400 | -1.22593600 | 0.07560200 |
| C | 2.27569700 | 1.25370500 | -0.12132200 |
| H | 0.39555500 | 1.96967200 | -0.88079300 |
| C | 2.35126700 | -1.07945400 | 0.46873700 |
| H | 0.53478100 | -2.18970500 | 0.12342000 |
| C | 2.97688200 | 0.15845800 | 0.37228200 |
| H | 2.75962600 | 2.22071500 | -0.19747200 |
| H | 2.89913700 | -1.93743400 | 0.84185900 |
| H | 4.01097500 | 0.26974300 | 0.67807300 |
| H | -2.11541800 | -1.21299900 | 0.91007200 |

| | | |
|---|---|---|
| 23 | 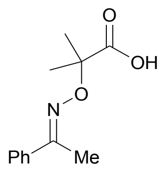 | 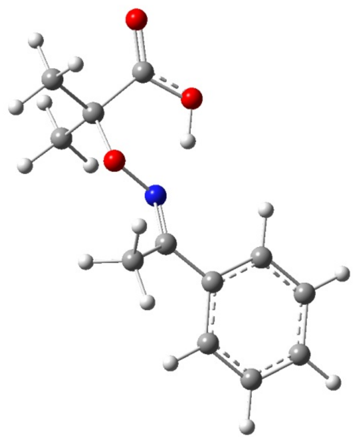 |



Cartesian Coordinates

| | | | |
|---|---|---|---|
| N | -0.26985100 | 0.26149500 | -0.02965400 |
| C | 0.77361100 | 0.95509800 | -0.30812000 |
| O | -1.44661800 | 0.93044500 | -0.30951300 |
| C | 0.67739100 | 2.36381000 | -0.80141400 |
| H | 0.13204400 | 2.98357700 | -0.08542100 |
| H | 1.65842500 | 2.79341300 | -0.99137200 |
| H | 0.09838100 | 2.38251900 | -1.72833600 |
| C | 2.07543200 | 0.28344100 | -0.13296100 |
| C | 2.20846500 | -1.08991400 | -0.36478900 |
| C | 3.19722100 | 1.00769000 | 0.28126500 |
| C | 3.42817800 | -1.72120100 | -0.17463300 |
| H | 1.35586500 | -1.65494100 | -0.72153300 |
| C | 4.41533400 | 0.37178300 | 0.47728400 |
| H | 3.11465800 | 2.07105800 | 0.47525700 |
| C | 4.53504000 | -0.99375700 | 0.24937200 |
| H | 3.51736500 | -2.78401400 | -0.36843100 |
| H | 5.27306300 | 0.94535200 | 0.80969300 |
| H | 5.48874500 | -1.48826900 | 0.39440900 |
| C | -2.61435800 | 0.28623500 | 0.26793000 |
| C | -2.78704000 | -1.17908900 | -0.20415200 |
| O | -3.87087500 | -1.63414900 | -0.44911300 |
| O | -1.68012100 | -1.91726600 | -0.24216800 |
| C | -2.51297300 | 0.30142700 | 1.78946100 |
| C | -3.76689600 | 1.12943700 | -0.23564700 |
| H | -2.43736200 | 1.33316900 | 2.14041900 |
| H | -3.40297900 | -0.15594100 | 2.22621000 |
| H | -1.63671500 | -0.25142400 | 2.13177500 |
| H | -3.67617900 | 2.14850500 | 0.14499000 |
| H | -3.77185000 | 1.15521000 | -1.32612400 |
| H | -4.70783900 | 0.69455500 | 0.09710900 |
| H | -0.91217400 | -1.32522400 | -0.04628600 |

| | | |
|---|---|---|
| 24 | 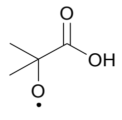 | 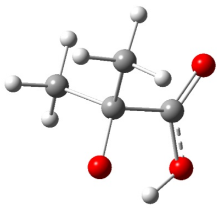 |



Cartesian Coordinates

| | | | |
|---|---|---|---|
| O | 0.69758100 | -1.28472200 | -0.75087600 |
| C | 0.58139400 | -0.11131500 | -0.09679700 |
| C | -0.94708900 | 0.25262800 | -0.01002100 |
| O | -1.32961000 | 1.35698600 | 0.23625000 |
| O | -1.73687600 | -0.79381300 | -0.20223600 |
| C | 0.97362400 | -0.37936700 | 1.39029200 |
| C | 1.40220400 | 1.02267000 | -0.69444100 |
| H | 2.02367700 | -0.67006800 | 1.42602400 |

| | | | |
|---|---|---|---|
| H | 0.82072000 | 0.54504900 | 1.94906300 |
| H | 0.36192200 | -1.17503500 | 1.81578600 |
| H | 2.46343100 | 0.77537600 | -0.63048800 |
| H | 1.14145200 | 1.15699300 | -1.74560900 |
| H | 1.21447100 | 1.95930700 | -0.16993800 |
| H | -1.13522600 | -1.52692500 | -0.44414400 |

25

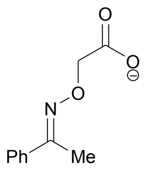


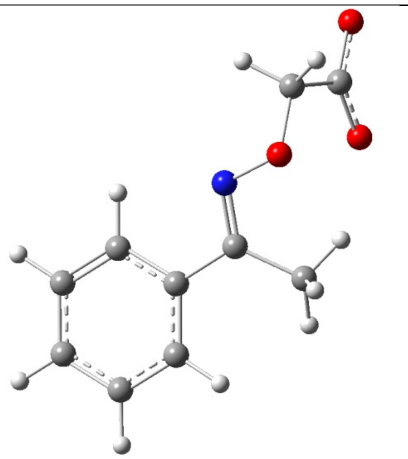

Cartesian Coordinates

| | | | |
|---|---|---|---|
| N | -0.59579600 | -0.20238600 | 0.88206200 |
| C | 0.21901100 | 0.65568200 | 0.37604200 |
| O | -1.79537000 | 0.36870100 | 1.20853500 |
| C | -0.22557600 | 2.03757100 | 0.01436100 |
| H | -0.32413400 | 2.68023000 | 0.89546600 |
| H | 0.45557900 | 2.51076500 | -0.69413700 |
| H | -1.21989800 | 1.91961900 | -0.43614900 |
| C | 1.58841100 | 0.16881900 | 0.11862900 |
| C | 1.88308900 | -1.20328000 | 0.16395400 |
| C | 2.63552600 | 1.05137600 | -0.16764800 |
| C | 3.16564400 | -1.66485100 | -0.07418500 |
| H | 1.07406000 | -1.88804700 | 0.38557900 |
| C | 3.92543000 | 0.58616300 | -0.40127600 |
| H | 2.44320900 | 2.11736400 | -0.19848800 |
| C | 4.19970400 | -0.77352200 | -0.35840700 |
| H | 3.36366800 | -2.73173800 | -0.04384000 |
| H | 4.71863200 | 1.29479400 | -0.61827400 |
| H | 5.20428300 | -1.13832700 | -0.54569900 |
| C | -2.88948900 | -0.49402700 | 0.87979500 |
| H | -2.59297200 | -1.53340900 | 1.05287400 |
| H | -3.69035700 | -0.22851300 | 1.57423600 |
| C | -3.40487200 | -0.33450700 | -0.58488300 |
| O | -2.80428900 | 0.48211800 | -1.30925700 |
| O | -4.38068700 | -1.07114300 | -0.81731300 |

26

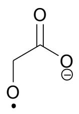


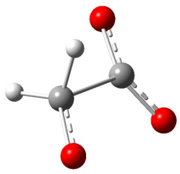

Cartesian Coordinates

| | | | |
|---|---|---|---|
| O | 1.96569100 | 0.27069600 | -0.00022000 |
| C | 1.06008100 | -0.60440900 | 0.00021500 |
| H | 0.85679800 | -1.20141200 | -0.91857700 |
| H | 0.85681600 | -1.20052300 | 0.91960000 |
| C | -0.80572900 | 0.09204100 | -0.00000800 |
| O | -0.84088700 | 1.29827300 | 0.00007000 |
| O | -1.52977000 | -0.88445100 | -0.00013300 |

27

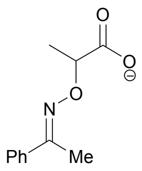


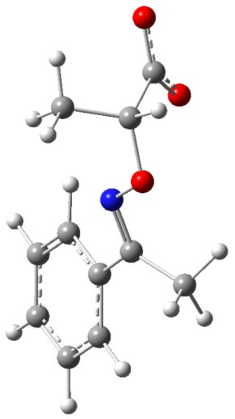

Cartesian Coordinates

| | | | |
|---|---|---|---|
| N | -0.51452800 | 0.41777800 | 0.34709200 |
| C | 0.53494900 | 1.02662300 | -0.07741800 |
| O | -1.63049100 | 1.18097100 | 0.27039900 |
| C | 0.48668200 | 2.40768200 | -0.65707100 |
| H | 1.36879300 | 2.62328800 | -1.26189600 |
| H | -0.40944400 | 2.48101300 | -1.27678200 |
| H | 0.39860500 | 3.17280700 | 0.12243700 |
| C | 1.78808600 | 0.25953100 | -0.00860200 |
| C | 3.03689200 | 0.88966800 | 0.03043600 |
| C | 1.75269500 | -1.14336300 | 0.01401300 |
| C | 4.21084900 | 0.14806500 | 0.10682100 |
| H | 3.09169700 | 1.97314700 | 0.01719900 |
| C | 2.92378500 | -1.87828400 | 0.09066900 |
| H | 0.78440500 | -1.62458700 | -0.05645300 |
| C | 4.16210500 | -1.23944400 | 0.14011300 |
| H | 5.16762600 | 0.66000000 | 0.14316700 |
| H | 2.87323500 | -2.96252300 | 0.09671600 |
| H | 5.07764100 | -1.81944100 | 0.19458900 |
| C | -2.82345200 | 0.38274700 | 0.44630700 |
| H | -3.61115700 | 1.13300700 | 0.32891900 |
| C | -3.03860900 | -0.68465200 | -0.68539200 |
| O | -2.17560300 | -0.74108500 | -1.57591300 |
| O | -4.09715600 | -1.32633600 | -0.52608900 |
| C | -2.89452800 | -0.22179700 | 1.83539500 |
| H | -2.88407900 | 0.54543600 | 2.61845000 |
| H | -3.81663100 | -0.80035200 | 1.89097300 |
| H | -2.04971000 | -0.89529200 | 1.99423000 |

28

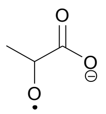


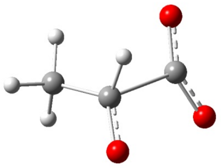

Cartesian Coordinates

| | | | |
|---|---|---|---|
| O | 1.42139000 | -1.21800600 | -0.27263900 |
| C | 0.93350000 | -0.07126400 | -0.42268500 |
| H | 0.71065100 | 0.30307000 | -1.44859900 |
| C | -1.09666100 | -0.03663700 | -0.01609900 |
| O | -1.51389100 | 1.02721600 | -0.42878300 |
| O | -1.44577800 | -1.05089100 | 0.53320100 |
| C | 1.35693800 | 1.05269900 | 0.52346600 |
| H | 2.40327000 | 1.33028700 | 0.32417100 |
| H | 0.72540000 | 1.93643500 | 0.40553100 |
| H | 1.30425200 | 0.69486800 | 1.55657500 |

| | | |
|---|---|---|
| 29 | 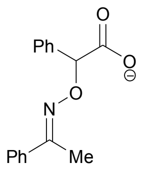 | 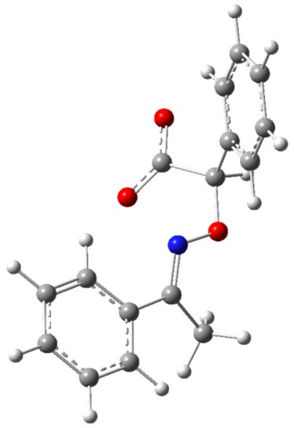 |

Cartesian Coordinates

| | | | |
|---|---|---|---|
| N | -0.41283100 | -0.44291700 | 0.45008200 |
| C | -1.58956900 | -0.61609100 | 0.93283400 |
| O | 0.55019100 | -1.06857000 | 1.19016600 |
| C | -1.85971600 | -1.47348300 | 2.13215600 |
| H | -2.91060000 | -1.75930300 | 2.19483000 |
| H | -1.24128500 | -2.37010000 | 2.05587800 |
| H | -1.57557800 | -0.96965600 | 3.06246000 |
| C | -2.67800100 | 0.04050600 | 0.18927600 |
| C | -2.51091800 | 0.34079100 | -1.17069000 |
| C | -3.89135200 | 0.37273000 | 0.79998500 |
| C | -3.52124500 | 0.96672700 | -1.88185300 |
| H | -1.58050000 | 0.04666100 | -1.64205800 |
| C | -4.90315200 | 1.00121600 | 0.08229000 |
| H | -4.04133100 | 0.15263200 | 1.85167100 |
| C | -4.72315500 | 1.30338100 | -1.26122400 |
| H | -3.37650300 | 1.18310500 | -2.93542700 |
| H | -5.83444600 | 1.25721100 | 0.57792700 |
| H | -5.51361800 | 1.78923800 | -1.82395800 |
| C | 1.78206300 | -1.17156500 | 0.46074300 |
| H | 2.39488500 | -1.77682300 | 1.13615500 |
| C | 1.64818000 | -2.05694300 | -0.85157400 |
| O | 0.50159700 | -2.38238300 | -1.17742900 |
| O | 2.75650400 | -2.34700900 | -1.34481400 |
| C | 2.46080700 | 0.16857600 | 0.29006400 |
| C | 1.99653300 | 1.31112600 | 0.94651200 |
| C | 3.61419000 | 0.28056700 | -0.49544200 |
| C | 2.65612500 | 2.52836200 | 0.81913000 |
| H | 1.10386700 | 1.24564900 | 1.55454000 |
| C | 4.27091000 | 1.49810800 | -0.61922300 |
| H | 3.94654600 | -0.61277100 | -1.01365100 |
| C | 3.79980500 | 2.63128500 | 0.03660500 |
| H | 2.26910400 | 3.40229700 | 1.33521500 |
| H | 5.15968000 | 1.56205300 | -1.24055900 |
| H | 4.31423400 | 3.58216500 | -0.06452300 |

| | | |
|---|---|---|
| 30 | 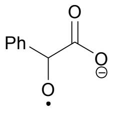 | 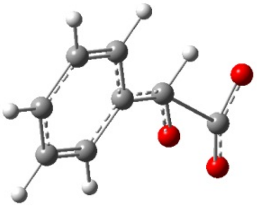 |

Cartesian Coordinates

| | | | |
|---|---|---|---|
| O | 1.48569400 | 2.14171100 | -0.40509500 |
| C | 0.85954000 | 1.21180100 | -0.96905000 |
| H | 1.22719600 | 0.80672600 | -1.94294000 |
| C | 2.13464400 | -1.04233100 | 0.51040600 |

| | | | |
|---|---|---|---|
| O | 2.07789600 | -0.53406700 | 1.55120200 |
| O | 2.28676700 | -1.65476200 | -0.46725200 |
| C | -0.34126100 | 0.59247700 | -0.50435900 |
| C | -0.96668900 | 1.00887500 | 0.71215000 |
| C | -0.99292400 | -0.44713000 | -1.23204400 |
| C | -2.13222900 | 0.42110500 | 1.15178000 |
| H | -0.48402800 | 1.80334900 | 1.27099300 |
| C | -2.15802100 | -1.02513100 | -0.77770600 |
| H | -0.54532700 | -0.78517900 | -2.16482000 |
| C | -2.75698700 | -0.60757700 | 0.42595300 |
| H | -2.57983900 | 0.76249900 | 2.08388100 |
| H | -2.62257300 | -1.81785000 | -1.36135200 |
| H | -3.67472600 | -1.06513100 | 0.78061700 |

| | | |
|---|---|---|
| 31 | 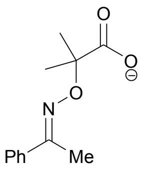 | 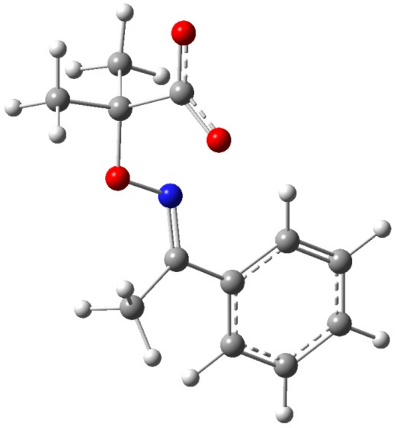 |

Cartesian Coordinates

| | | | |
|---|---|---|---|
| N | -0.26483900 | 0.30690700 | 0.32830900 |
| C | 0.74660500 | 0.96645100 | -0.11176000 |
| O | -1.42380400 | 0.99767800 | 0.22117900 |
| C | 0.61149600 | 2.31969100 | -0.74063100 |
| H | 0.39885800 | 3.09333100 | 0.00515200 |
| H | 1.50643200 | 2.60071400 | -1.29736200 |
| H | -0.24574200 | 2.29076000 | -1.41724200 |
| C | 2.04520400 | 0.28196500 | -0.01365500 |
| C | 2.09755200 | -1.11950700 | 0.03910800 |
| C | 3.25146100 | 0.99018200 | 0.02444400 |
| C | 3.31159300 | -1.77751700 | 0.14362600 |
| H | 1.16239200 | -1.66228700 | -0.03084100 |
| C | 4.46865200 | 0.32590400 | 0.12874800 |
| H | 3.23824300 | 2.07440500 | -0.01082400 |
| C | 4.50655500 | -1.06100500 | 0.19155400 |
| H | 3.32921700 | -2.86241900 | 0.17267000 |
| H | 5.39098000 | 0.89762400 | 0.16384900 |
| H | 5.45596500 | -1.58099800 | 0.26780900 |
| C | -2.58626900 | 0.14470000 | 0.40546000 |
| C | -2.68401100 | -0.96911100 | -0.71454300 |
| O | -3.69024600 | -1.69503100 | -0.57389800 |
| O | -1.79360500 | -0.97295700 | -1.57862000 |
| C | -2.56574800 | -0.48440300 | 1.78830100 |
| C | -3.75288700 | 1.10277400 | 0.24889100 |
| H | -2.62985200 | 0.27824600 | 2.57498100 |
| H | -3.41516800 | -1.16428700 | 1.85663600 |
| H | -1.64708900 | -1.05676600 | 1.92649500 |
| H | -3.75383500 | 1.86828700 | 1.03421600 |
| H | -3.70380400 | 1.59716200 | -0.72504000 |
| H | -4.67270400 | 0.51961900 | 0.29479400 |

| | | |
|---|---|---|
| 32 | 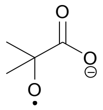 | 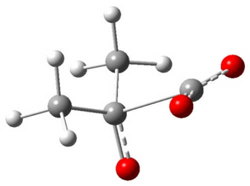 |

Cartesian Coordinates

| | | | |
|---|---|---|---|
| O | 0.96084500 | -0.00122700 | 1.42752900 |
| C | 0.78471300 | -0.00021800 | 0.17004600 |
| C | -1.23759600 | 0.00021300 | -0.02867200 |
| O | -1.63848600 | -1.14259100 | -0.04824100 |
| O | -1.63806800 | 1.14315500 | -0.04914200 |
| C | 1.14781600 | 1.28342500 | -0.58002700 |
| C | 1.14708000 | -1.28291200 | -0.58204400 |
| H | 2.23732800 | 1.42830000 | -0.53194200 |
| H | 0.84431100 | 1.26403700 | -1.63181600 |
| H | 0.65657500 | 2.12420400 | -0.09095600 |
| H | 2.23652000 | -1.42846600 | -0.53434300 |
| H | 0.65538200 | -2.12410400 | -0.09415500 |
| H | 0.84347700 | -1.26171400 | -1.63377400 |

## 8.4 Structural Analysis for Carboxylate Anions

***DFT Method:*** UB3LYP/6-31+G(d,p) – $CH_2Cl_2$ (solvent)

**Electronic Properties**

| No. | Structure | HOMO (Hartree) | LUMO (Hartree) | Ionization Potential (IP, eV) | Electron affinity (A, eV) | Electro negativity (χ, eV) | Electronic Chemical Potential (μ, eV) | Chemical Hardness (η, eV) | Chemical Softness (S, meV) |
|---|---|---|---|---|---|---|---|---|---|
| 1 | 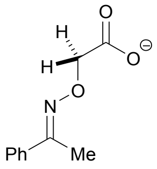 | -0.21544 | -0.04020 | 6.00 | 1.07 | 3.53 | -3.53 | 4.93 | 202.76 |
| 2 | 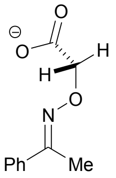 | -0.20667 | -0.03786 | 5.713 | 1.00 | 3.36 | -3.36 | 4.71 | 212.19 |
| 3 | 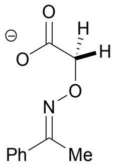 | -0.20653 | -0.03758 | 5.709 | 0.99 | 3.35 | -3.35 | 4.72 | 212.02 |
| 4 | 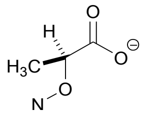 | -0.21206 | -0.03923 | 5.85 | 1.04 | 3.45 | -3.45 | 4.82 | 207.63 |

| | | | | | | | | | |
|---|---|---|---|---|---|---|---|---|---|
| 5 | 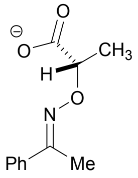 | -0.20505 | -0.03668 | 5.67 | 0.97 | 3.32 | -3.32 | 4.71 | 212.50 |
| 6 | 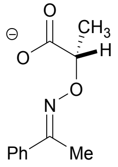 | -0.20335 | -0.03472 | 5.63 | 0.91 | 3.27 | -3.27 | 4.71 | 212.11 |
| 7 | 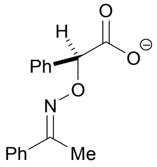 | -0.21311 | -0.04022 | 5.97 | 1.07 | 3.52 | -3.52 | 4.91 | 203.86 |
| 8 | 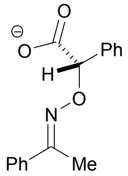 | -0.20595 | -0.03760 | 5.68 | 0.99 | 3.34 | -3.34 | 4.69 | 213.32 |
| 9 | 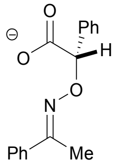 | -0.20554 | -0.03489 | 5.66 | 0.92 | 3.29 | -3.29 | 4.74 | 210.77 |
| 10 | 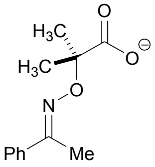 | -0.20894 | -0.03782 | 5.84 | 1.00 | 3.42 | -3.42 | 4.85 | 206.37 |

| | | | | | | | | | |
|---|---|---|---|---|---|---|---|---|---|
| 11 | 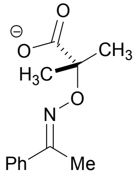 | -0.20301 | -0.03743 | 5.619 | 0.99 | 3.30 | -3.30 | 4.63 | 215.85 |
| 12 | 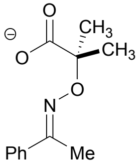 | -0.20304 | -0.03620 | 5.6 | 0.95 | 3.29 | -3.29 | 4.67 | 214.11 |
| 13 | 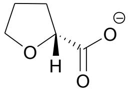 | -0.20286 | 0.01927 | 5.73 | -0.07 | 2.83 | -2.83 | 5.79 | 172.57 |
| 14 | 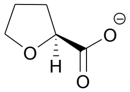 | -0.20633 | 0.02090 | 5.82 | -0.10 | 2.86 | -2.86 | 5.92 | 168.94 |

**HOMO Structures**

***DFT Method:*** UB3LYP/6-31+G(d,p) – $CH_2Cl_2$ (solvent)

| No. | Structure | HOMO Structures |
|---|---|---|
| 1 | 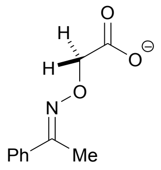 | 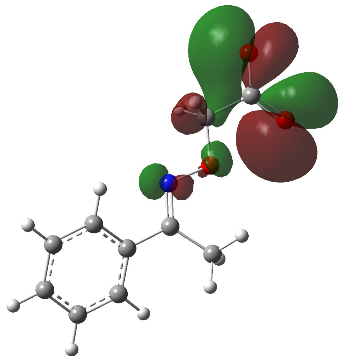 |
| 2 | 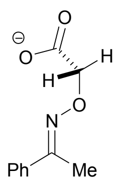 | 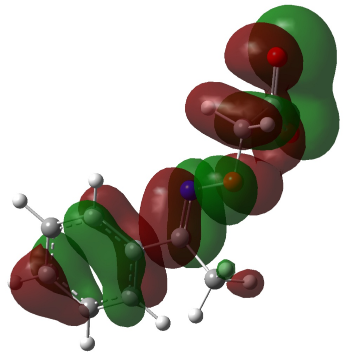 |
| 3 | 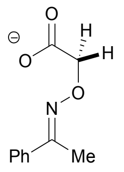 | 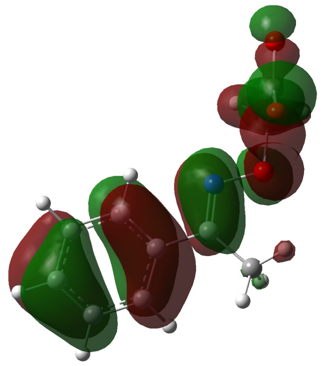 |

| | | |
|---|---|---|
| 4 | 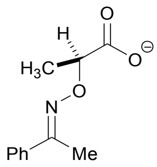 | 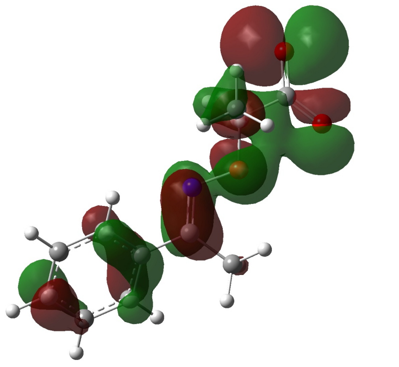 |
| 5 | 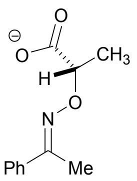 | 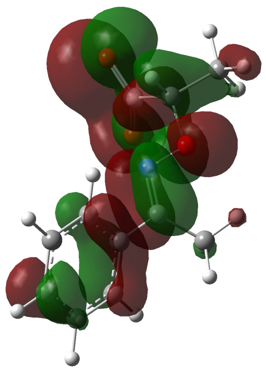 |
| 6 | 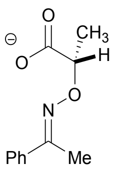 | 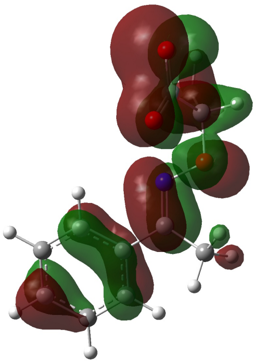 |
| 7 | 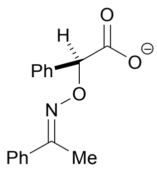 | 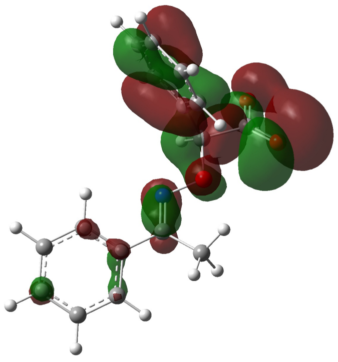 |

| | | |
|---|---|---|
| 8 | 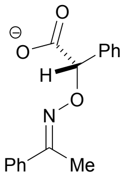 O<br>O<br>Ph<br>H<br>O<br>N<br>Ph<br>Me | 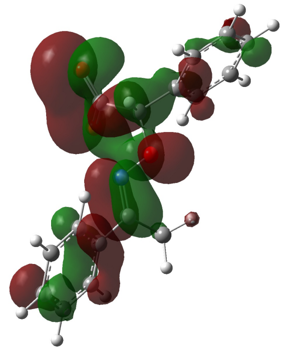 |
| 9 | 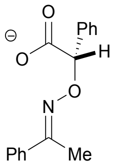 O<br>O<br>Ph<br>H<br>O<br>N<br>Ph<br>Me | 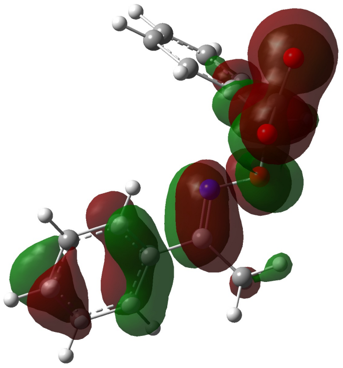 |
| 10 | 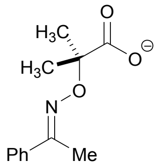 $H_3C$<br>$H_3C$<br>O<br>O<br>O<br>N<br>Ph<br>Me | 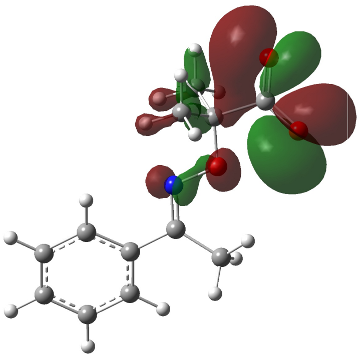 |
| 11 | 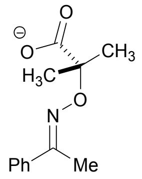 O<br>O<br>$CH_3$<br>$H_3C$<br>O<br>N<br>Ph<br>Me | 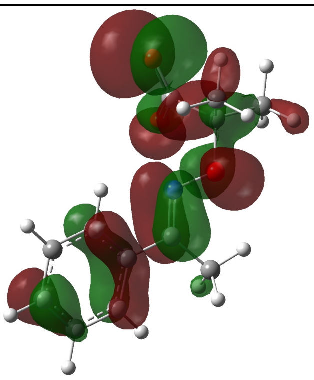 |

| | | |
|---|---|---|
| 12 | 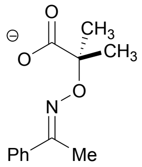 O<br>⊖<br>O<br>CH3<br>CH3<br>N<br>O<br>Ph<br>Me | 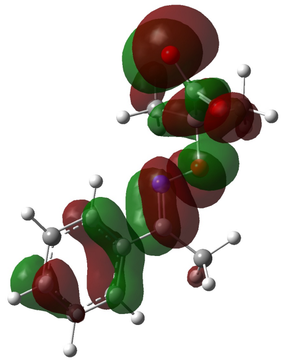 |
| 13 | 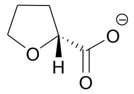 O<br>H<br>O<br>⊖<br>O | 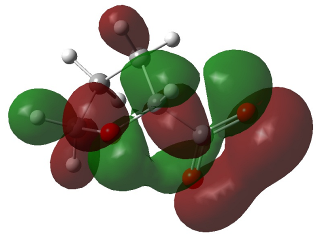 |
| 14 | 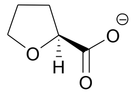 O<br>H<br>O<br>⊖<br>O | 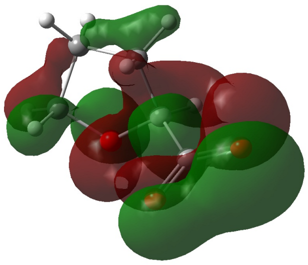 |

**Computed Energies** [values are in Hartree]

| No. | Species | Total Electronic Energy | Sum of Electronic and Zero-point Energies | Gibbs Free Energy |
|---|---|---|---|---|
| 1 | 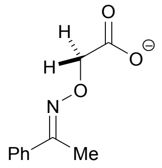 | -667.6539664 | -667.470454 | -667.512742 |
| 2 | 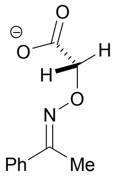 | -667.6541017 | -667.470300 | -667.512356 |
| 3 | 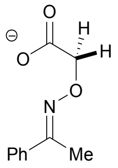 | -667.6545065 | -667.470510 | -667.512050 |
| 4 | 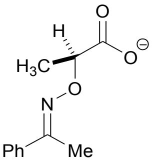 | -706.9736549 | -706.762121 | -706.805636 |
| 5 | 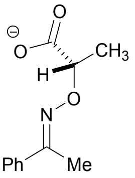 | -706.9743357 | -706.762550 | -706.805272 |
| 6 | 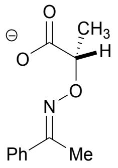 | -706.9708111 | -706.759068 | -706.801941 |
| 7 | 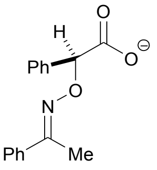 | -898.7207971 | -898.456304 | -898.505343 |
| 8 | 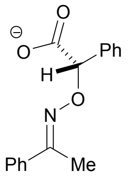 | -898.7206255 | -898.456047 | -898.505011 |

| | | | | |
|---|---|---|---|---|
| 9 | 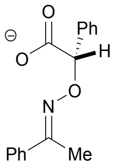 | -898.7160251 | -898.451371 | -898.499433 |
| 10 | 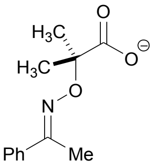 | -746.2901671 | -746.051231 | -746.095566 |
| 11 | 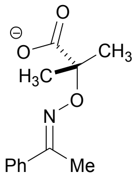 | -746.2905746 | -746.051643 | -746.096693 |
| 12 | 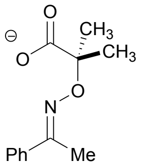 | -746.2909302 | -746.051710 | -746.095824 |
| 13 | 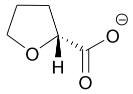 | -420.5873942 | -420.468561 | -420.500549 |
| 14 | 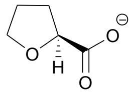 | -420.5880512 | -420.469372 | -420.501591 |

## Optimized Structures and Cartesian Coordinates

| No. | Species | Optimized Structures |
|---|---|---|
| 1 | 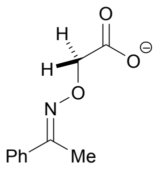 | 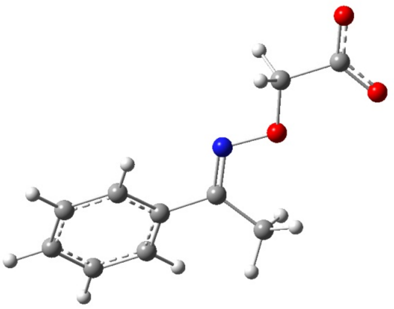 |



Cartesian Coordinates

| | | | |
|---|---|---|---|
| N | -0.44381500 | -0.26308100 | -0.05493800 |
| C | 0.46647400 | 0.63615200 | 0.12278900 |
| O | -1.73474600 | 0.26383400 | 0.02326300 |
| C | 0.14996500 | 2.08965000 | 0.37187100 |
| H | 1.00386800 | 2.61964500 | 0.79556900 |
| H | -0.14456100 | 2.58846800 | -0.55942200 |
| H | -0.69885800 | 2.17187600 | 1.05497900 |
| C | 1.87387500 | 0.15874900 | 0.05439600 |
| C | 2.20166900 | -1.17864800 | 0.35184500 |
| C | 2.91025900 | 1.03238600 | -0.32066700 |
| C | 3.51958200 | -1.62601300 | 0.26763200 |
| H | 1.41357700 | -1.85815200 | 0.65715100 |
| C | 4.23048800 | 0.58143300 | -0.40787900 |
| H | 2.68902000 | 2.06659700 | -0.56391700 |
| C | 4.54186000 | -0.74840200 | -0.11371500 |
| H | 3.75152600 | -2.65970800 | 0.50831000 |
| H | 5.01382400 | 1.27178900 | -0.70748500 |
| H | 5.56856700 | -1.09729000 | -0.17551500 |
| C | -2.69347200 | -0.79328200 | -0.15152300 |
| H | -2.54697000 | -1.55065800 | 0.62738800 |
| C | -4.14560000 | -0.27058800 | -0.07765900 |
| O | -4.35034500 | 0.95633100 | 0.10593600 |
| O | -5.00724000 | -1.18628500 | -0.21787600 |
| H | -2.53523300 | -1.27067200 | -1.12560900 |

| No. | Species | Optimized Structures |
|---|---|---|
| 2 | 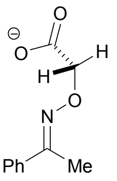 | 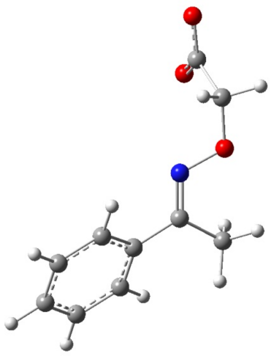 |



Cartesian Coordinates

| | | | |
|---|---|---|---|
| N | 0.65890700 | 0.14306500 | -0.51047800 |
| C | -0.36043800 | 0.90697200 | -0.29908500 |
| O | 1.83867400 | 0.86498100 | -0.68740300 |
| C | -0.25752300 | 2.40987800 | -0.22009700 |
| H | -1.23351200 | 2.88644200 | -0.31843200 |
| H | 0.18940200 | 2.71768300 | 0.73247300 |
| H | 0.39910200 | 2.77849700 | -1.01236000 |
| C | -1.66616300 | 0.21671300 | -0.12064800 |
| C | -1.89655900 | -1.05745500 | -0.67587000 |

| | | | |
|---|---|---|---|
| C | -2.70002300 | 0.81755800 | 0.61985500 |
| C | -3.11565600 | -1.70779100 | -0.48895500 |
| H | -1.11252600 | -1.52780500 | -1.25899100 |
| C | -3.92072700 | 0.16324200 | 0.80849500 |
| H | -2.55064700 | 1.79350200 | 1.07007200 |
| C | -4.13532400 | -1.10121600 | 0.25459700 |
| H | -3.27378400 | -2.68769600 | -0.93063600 |
| H | -4.70133000 | 0.64355800 | 1.39156900 |
| H | -5.08559300 | -1.60774300 | 0.39655500 |
| C | 2.94964300 | -0.02973600 | -0.83733300 |
| H | 2.66342200 | -0.85971900 | -1.49026900 |
| H | 3.71509700 | 0.56409500 | -1.34708400 |
| C | 3.55956100 | -0.58964800 | 0.47284700 |
| O | 3.14275600 | -0.15727000 | 1.57687000 |
| O | 4.47322800 | -1.44188300 | 0.26773600 |

| | | |
|---|---|---|
| 3 | 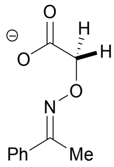  | 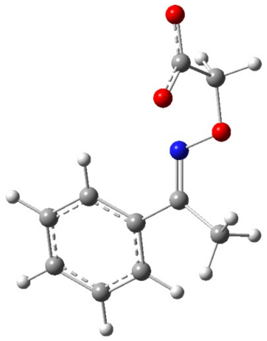 |

Cartesian Coordinates

| | | | |
|---|---|---|---|
| N | 0.64885700 | 0.29448800 | 0.50685600 |
| C | -0.35756100 | 0.93481300 | 0.01277300 |
| O | 1.81815600 | 1.05359200 | 0.51585100 |
| C | -0.26291500 | 2.34337300 | -0.51795800 |
| H | -1.07627800 | 2.56243500 | -1.21157300 |
| H | -0.30001100 | 3.07452700 | 0.29912100 |
| H | 0.69191500 | 2.48029100 | -1.02990000 |
| C | -1.65293000 | 0.20293900 | 0.00422700 |
| C | -1.69121900 | -1.20523700 | -0.00297200 |
| C | -2.87199300 | 0.90410600 | 0.01034100 |
| C | -2.90830800 | -1.88520600 | 0.00578200 |
| H | -0.75778100 | -1.75682400 | -0.02581100 |
| C | -4.09144000 | 0.22059100 | 0.02227100 |
| H | -2.87536800 | 1.98931700 | 0.02121800 |
| C | -4.11586500 | -1.17619500 | 0.01981300 |
| H | -2.91545100 | -2.97149900 | -0.00769600 |
| H | -5.02086600 | 0.78287800 | 0.03408000 |
| H | -5.06313600 | -1.70766500 | 0.02276400 |
| C | 2.93405200 | 0.23460600 | 0.89050300 |
| H | 2.64789800 | -0.40756200 | 1.72965400 |
| C | 3.54727000 | -0.63120500 | -0.23916200 |
| H | 3.69382800 | 0.94215800 | 1.23717600 |
| O | 4.48313300 | -1.38354900 | 0.16248300 |
| O | 3.10604900 | -0.50816500 | -1.40967600 |

| | | |
|---|---|---|
| 4 | 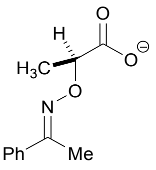 | 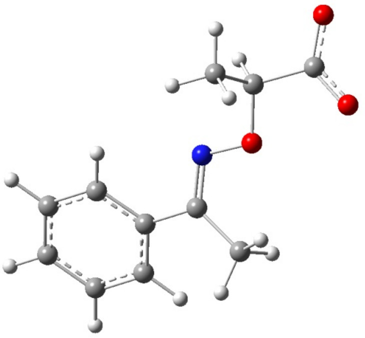 |

Cartesian Coordinates

| | | | |
|---|---|---|---|
| N | -0.26054700 | -0.14432400 | -0.06934800 |
| C | 0.68026400 | 0.70364500 | 0.18510100 |
| O | -1.53421500 | 0.40534800 | 0.07141600 |
| C | 0.42421300 | 2.13210300 | 0.59649900 |
| H | 1.25521900 | 2.53037400 | 1.18182700 |
| H | 0.28825800 | 2.77260800 | -0.28388100 |
| H | -0.49419600 | 2.19309400 | 1.18263100 |
| C | 2.07206000 | 0.19987400 | 0.03207900 |
| C | 2.36457700 | -1.17474300 | 0.13052700 |
| C | 3.13130200 | 1.09037500 | -0.21893800 |
| C | 3.66956700 | -1.63982800 | -0.02543000 |
| H | 1.55893500 | -1.87022400 | 0.33847100 |
| C | 4.43882000 | 0.62217400 | -0.37852100 |
| H | 2.93787000 | 2.15455300 | -0.30638600 |
| C | 4.71482200 | -0.74383600 | -0.28197400 |
| H | 3.87394200 | -2.70332800 | 0.06131900 |
| H | 5.23983200 | 1.32797600 | -0.57920700 |
| H | 5.73153900 | -1.10731400 | -0.39971100 |
| C | -2.54099500 | -0.61879200 | -0.13280500 |
| C | -3.91048300 | 0.06097200 | -0.40518200 |
| O | -4.09464000 | 1.23515400 | 0.01077800 |
| O | -4.74206200 | -0.68172800 | -1.00345100 |
| H | -2.25443800 | -1.18950300 | -1.02185700 |
| C | -2.61669800 | -1.54131300 | 1.08531100 |
| H | -1.64679600 | -2.01408600 | 1.26849500 |
| H | -2.90320200 | -0.97261600 | 1.97674000 |
| H | -3.36048800 | -2.32523700 | 0.91704700 |

| | | |
|---|---|---|
| 5 | 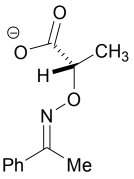 | 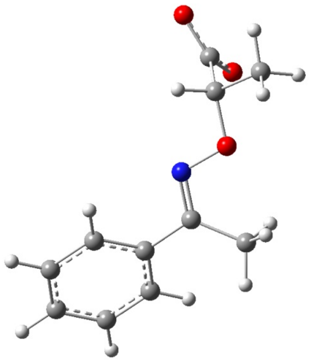 |

Cartesian Coordinates

| | | | |
|---|---|---|---|
| N | 0.40528500 | 0.10506800 | -0.40421900 |
| C | -0.58661900 | 0.88790500 | -0.13881200 |
| O | 1.61655300 | 0.79043400 | -0.47317200 |
| C | -0.41755200 | 2.36471700 | 0.11830100 |
| H | -1.37557900 | 2.88477400 | 0.13831000 |
| H | 0.09935800 | 2.53336700 | 1.07000100 |
| H | 0.20819700 | 2.80753100 | -0.66201300 |
| C | -1.92790600 | 0.24647900 | -0.09371900 |
| C | -2.20593400 | -0.90261000 | -0.85924700 |

| | | | |
|---|---|---|---|
| C | -2.94813900 | 0.76756100 | 0.72236600 |
| C | -3.45876300 | -1.51273300 | -0.80233600 |
| H | -1.43197600 | -1.30525000 | -1.50349000 |
| C | -4.20197900 | 0.15305000 | 0.78100200 |
| H | -2.76192200 | 1.64535800 | 1.33281400 |
| C | -4.46411000 | -0.98839600 | 0.01865400 |
| H | -3.65382700 | -2.39461700 | -1.40610700 |
| H | -4.97191600 | 0.56712900 | 1.42581600 |
| H | -5.44059900 | -1.46224400 | 0.05975100 |
| C | 2.71218700 | -0.13954100 | -0.61052800 |
| H | 2.42411900 | -0.89221300 | -1.35171800 |
| C | 3.07452900 | -0.87090600 | 0.71543400 |
| O | 2.77379800 | -0.31692800 | 1.80500800 |
| O | 3.69431500 | -1.96252500 | 0.55128200 |
| C | 3.89367900 | 0.68369800 | -1.11894600 |
| H | 4.76266300 | 0.03438900 | -1.25392800 |
| H | 3.65607500 | 1.15587500 | -2.07794400 |
| H | 4.15472700 | 1.46723200 | -0.39991700 |
| 6 | 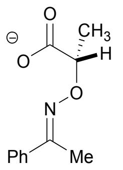  | 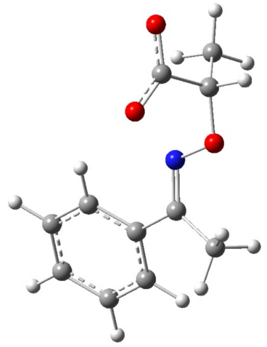 | |
| Cartesian Coordinates | | | |
| N | 0.48650100 | 0.35102300 | 0.25762300 |
| C | -0.57190300 | 0.98306800 | -0.12497100 |
| O | 1.63013700 | 1.14078000 | 0.19413200 |
| C | -0.55657500 | 2.41400500 | -0.60382800 |
| H | -1.43186700 | 2.63877800 | -1.21559100 |
| H | -0.53901300 | 3.11280800 | 0.24193700 |
| H | 0.34651200 | 2.59667500 | -1.19037900 |
| C | -1.84633600 | 0.21864500 | -0.05582800 |
| C | -1.85432800 | -1.18879000 | -0.12071600 |
| C | -3.07591400 | 0.88651200 | 0.08689600 |
| C | -3.05073100 | -1.89976100 | -0.03446400 |
| H | -0.91467500 | -1.71468300 | -0.24991100 |
| C | -4.27415600 | 0.17193500 | 0.17629000 |
| H | -3.10236400 | 1.96999300 | 0.14492600 |
| C | -4.26796100 | -1.22383700 | 0.11612200 |
| H | -3.03490900 | -2.98446100 | -0.09409100 |
| H | -5.21106600 | 0.70914900 | 0.29413200 |
| H | -5.19919700 | -1.77935000 | 0.17968900 |
| C | 2.82075500 | 0.38093200 | 0.51025700 |
| C | 3.24718600 | -0.61607000 | -0.61347600 |
| H | 3.58416500 | 1.16858800 | 0.51792400 |
| O | 4.25777100 | -1.32048100 | -0.32169700 |
| O | 2.61394100 | -0.60460900 | -1.70015800 |
| C | 2.74467400 | -0.24445200 | 1.90364400 |
| H | 3.71918900 | -0.65955500 | 2.16701500 |
| H | 2.46972000 | 0.51420700 | 2.64480200 |
| H | 2.00493800 | -1.04795600 | 1.93842700 |

| | | |
|---|---|---|
| 7 | 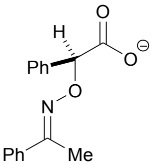 | 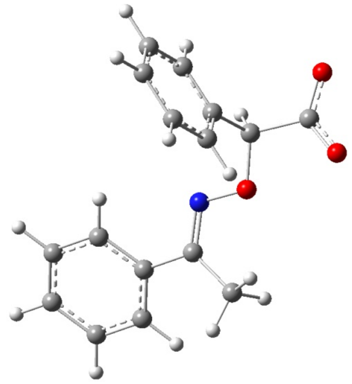 |



Cartesian Coordinates

| | | | |
|---|---|---|---|
| N | -0.56393400 | -0.57347800 | -0.27952200 |
| C | -1.61158800 | -0.93127900 | 0.38482500 |
| O | 0.56293700 | -1.33529800 | 0.04828300 |
| C | -1.61101200 | -2.04597400 | 1.40102100 |
| H | -2.41785400 | -1.92339600 | 2.12573400 |
| H | -1.73461000 | -3.01895100 | 0.90971800 |
| H | -0.65436000 | -2.07173800 | 1.92571200 |
| C | -2.86310800 | -0.18702300 | 0.07692400 |
| C | -2.82283400 | 1.11768700 | -0.45284900 |
| C | -4.11916600 | -0.77780500 | 0.30279800 |
| C | -4.00025200 | 1.80147900 | -0.75291800 |
| H | -1.86074100 | 1.59024000 | -0.61773400 |
| C | -5.29854400 | -0.09210100 | -0.00219700 |
| H | -4.18279800 | -1.78433800 | 0.70344100 |
| C | -5.24517300 | 1.20005500 | -0.53033400 |
| H | -3.94742200 | 2.80979600 | -1.15388200 |
| H | -6.25762500 | -0.57110900 | 0.17303700 |
| H | -6.16125900 | 1.73558300 | -0.76208400 |
| C | 1.70750200 | -0.85200400 | -0.68944500 |
| C | 2.83101600 | -1.94081200 | -0.63033500 |
| O | 2.84180800 | -2.72442200 | 0.35181100 |
| O | 3.64341400 | -1.89092700 | -1.59478500 |
| H | 1.41195700 | -0.73114500 | -1.73520700 |
| C | 2.20673500 | 0.47426800 | -0.14719200 |
| C | 2.57414000 | 1.50586700 | -1.02178300 |
| C | 2.33631800 | 0.68250700 | 1.23462800 |
| C | 3.06802000 | 2.71880300 | -0.53071600 |
| H | 2.47171400 | 1.35887000 | -2.09373400 |
| C | 2.82305700 | 1.89538600 | 1.72835300 |
| H | 2.05290800 | -0.11005300 | 1.92033600 |
| C | 3.19280700 | 2.91841700 | 0.84745300 |
| H | 3.34751600 | 3.50777800 | -1.22369900 |
| H | 2.91580000 | 2.04182000 | 2.80117900 |
| H | 3.57153600 | 3.86133100 | 1.23196500 |

| | | |
|---|---|---|
| 8 | 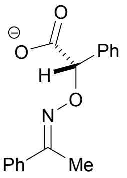 | 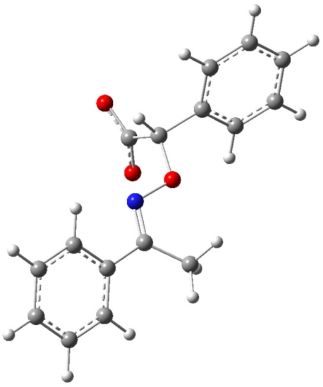 |

Cartesian Coordinates

| | | | |
|---|---|---|---|
| N | -0.82097400 | 0.10160700 | 0.16522800 |
| C | -1.70963600 | -0.79888300 | -0.09200500 |
| O | 0.47805700 | -0.37127800 | -0.02826700 |
| C | -1.35455000 | -2.17741100 | -0.59071100 |
| H | -2.22214600 | -2.83680000 | -0.61551700 |
| H | -0.91709100 | -2.12346600 | -1.59375100 |
| H | -0.59500400 | -2.62062400 | 0.06042000 |
| C | -3.12523100 | -0.40035000 | 0.13184600 |
| C | -3.45969100 | 0.57169900 | 1.09464300 |
| C | -4.16323300 | -0.97989900 | -0.61977700 |
| C | -4.78623100 | 0.95395900 | 1.29220000 |
| H | -2.66993100 | 1.01614000 | 1.69029100 |
| C | -5.49170100 | -0.59351300 | -0.42296100 |
| H | -3.93672500 | -1.72288100 | -1.37766700 |
| C | -5.81007300 | 0.37326500 | 0.53438400 |
| H | -5.02270200 | 1.70079000 | 2.04489200 |
| H | -6.27614400 | -1.04803900 | -1.02134500 |
| H | -6.84326800 | 0.66903900 | 0.69169900 |
| C | 1.42141600 | 0.70992400 | 0.14682800 |
| H | 1.15520400 | 1.24962400 | 1.06012200 |
| C | 1.41058200 | 1.73312300 | -1.03966400 |
| O | 1.00246800 | 1.33212900 | -2.15830800 |
| O | 1.85679800 | 2.87562200 | -0.73693700 |
| C | 2.78378500 | 0.07007400 | 0.30616200 |
| C | 3.55518200 | 0.31318800 | 1.45071600 |
| C | 3.30586100 | -0.76436800 | -0.69585400 |
| C | 4.82374400 | -0.25891800 | 1.59371700 |
| H | 3.16229500 | 0.95630600 | 2.23366800 |
| C | 4.56813100 | -1.34452700 | -0.55098900 |
| H | 2.72063600 | -0.94924400 | -1.59102500 |
| C | 5.33313100 | -1.09218500 | 0.59384500 |
| H | 5.40933400 | -0.05627500 | 2.48624900 |
| H | 4.95806300 | -1.98851900 | -1.33454200 |
| H | 6.31680400 | -1.54015000 | 0.70372100 |

9

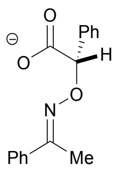


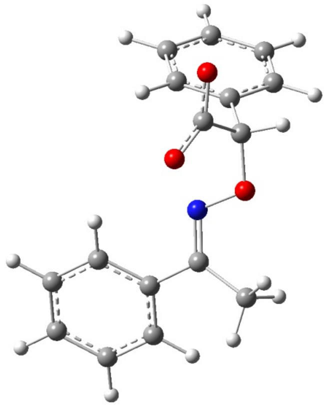

Cartesian Coordinates

| | | | |
|---|---|---|---|
| N | 0.33561600 | -0.63507300 | 0.32607400 |
| C | 1.50215200 | -0.86147100 | 0.82987100 |
| O | -0.66584000 | -1.36674500 | 0.95972800 |
| C | 1.74770200 | -1.81997800 | 1.96949000 |
| H | 2.78023300 | -2.17348900 | 1.97552600 |
| H | 1.54292800 | -1.34186400 | 2.93569400 |
| H | 1.07675600 | -2.67710300 | 1.88444500 |
| C | 2.62963300 | -0.10547200 | 0.22058000 |
| C | 2.54701700 | 0.37701600 | -1.10042200 |
| C | 3.80617000 | 0.14555700 | 0.94920700 |
| C | 3.60159000 | 1.09139200 | -1.66706200 |
| H | 1.65147100 | 0.17384200 | -1.67675100 |
| C | 4.86180500 | 0.86471300 | 0.38052800 |
| H | 3.89953800 | -0.20770200 | 1.97123600 |
| C | 4.76575400 | 1.34031500 | -0.92957500 |

| | | | |
|---|---|---|---|
| H | 3.51967500 | 1.44609900 | -2.69073700 |
| H | 5.75861000 | 1.05177100 | 0.96451900 |
| H | 5.58874900 | 1.89247500 | -1.37412600 |
| C | -1.95354100 | -1.21435000 | 0.30643500 |
| C | -2.07575500 | -1.97894900 | -1.05050300 |
| H | -2.60770500 | -1.74150700 | 1.00818500 |
| O | -3.25776600 | -2.01721200 | -1.49749800 |
| O | -1.04727600 | -2.50078000 | -1.54722000 |
| C | -2.39057800 | 0.24044200 | 0.25057800 |
| C | -2.99964500 | 0.82202400 | 1.37192500 |
| C | -2.17247100 | 1.03905600 | -0.88098100 |
| C | -3.37764100 | 2.16867500 | 1.36984800 |
| H | -3.18086400 | 0.21483100 | 2.25571600 |
| C | -2.54746900 | 2.38457300 | -0.88945800 |
| H | -1.70928500 | 0.60431300 | -1.76111000 |
| C | -3.15068100 | 2.95545100 | 0.23690000 |
| H | -3.85161600 | 2.59903500 | 2.24787900 |
| H | -2.37024700 | 2.98736100 | -1.77603500 |
| H | -3.44475500 | 4.00138100 | 0.22879600 |

10

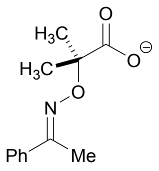


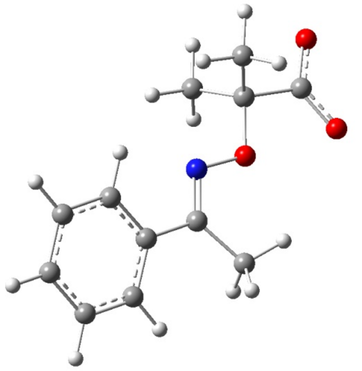

Cartesian Coordinates

| | | | |
|---|---|---|---|
| N | 0.09966500 | -0.07931900 | 0.00917000 |
| C | -0.85340500 | 0.78541200 | -0.10397300 |
| O | 1.36361700 | 0.49901100 | -0.04157700 |
| C | -0.62990500 | 2.26936100 | -0.26236800 |
| H | -1.37168800 | 2.70617300 | -0.93610200 |
| H | -0.71601000 | 2.78082500 | 0.70475200 |
| H | 0.37068100 | 2.46189700 | -0.64877900 |
| C | -2.23860100 | 0.24287800 | -0.05119600 |
| C | -2.50238600 | -1.11846300 | -0.30370600 |
| C | -3.32389200 | 1.08313100 | 0.25681700 |
| C | -3.80194100 | -1.61865800 | -0.24142500 |
| H | -1.67750400 | -1.77565000 | -0.55525000 |
| C | -4.62675900 | 0.57950900 | 0.32142500 |
| H | -3.15615000 | 2.13542100 | 0.46094200 |
| C | -4.87313400 | -0.77254800 | 0.07254000 |
| H | -3.98188600 | -2.67047400 | -0.44584200 |
| H | -5.44736300 | 1.24749600 | 0.56758600 |
| H | -5.88558700 | -1.16321600 | 0.11765900 |
| C | 2.42577500 | -0.50001200 | 0.07646300 |
| C | 2.31598800 | -1.20065300 | 1.43600600 |
| C | 2.33224100 | -1.49110700 | -1.09063900 |
| H | 2.37956400 | -0.96126500 | -2.04844700 |
| H | 3.16712700 | -2.19392600 | -1.04283100 |
| H | 1.39227300 | -2.04930900 | -1.04821400 |
| C | 3.77935400 | 0.29200000 | -0.01419100 |
| H | 1.37037100 | -1.74568200 | 1.51079700 |
| H | 3.14401500 | -1.90203100 | 1.55742700 |
| H | 2.36029700 | -0.46817600 | 2.24947100 |
| O | 4.81025700 | -0.42765300 | 0.11784100 |
| O | 3.74290000 | 1.53464700 | -0.21149900 |

11

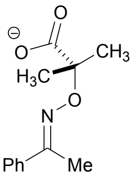


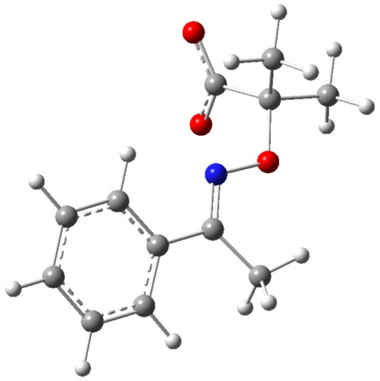

Cartesian Coordinates

| | | | |
|---|---|---|---|
| N | 0.29208800 | 0.25482700 | -0.19283300 |
| C | -0.76115800 | 0.98954600 | -0.06054400 |
| O | 1.47774500 | 0.97734700 | -0.19580800 |
| C | -0.73355700 | 2.49068400 | 0.09003400 |
| H | -1.50239400 | 2.95840600 | -0.53187800 |
| H | -0.92914700 | 2.77815700 | 1.13038000 |
| H | 0.24149000 | 2.88595300 | -0.19029700 |
| C | -2.06480500 | 0.26864500 | -0.04743200 |
| C | -2.15649800 | -1.08776800 | -0.42198300 |
| C | -3.24444600 | 0.92890400 | 0.34093700 |
| C | -3.38038200 | -1.75314500 | -0.40557700 |
| H | -1.25717700 | -1.61093500 | -0.72630800 |
| C | -4.47213600 | 0.25900800 | 0.35872700 |
| H | -3.21303500 | 1.97030100 | 0.64237300 |
| C | -4.54776500 | -1.08392500 | -0.01517800 |
| H | -3.42566400 | -2.79753300 | -0.70163600 |
| H | -5.36745200 | 0.79150200 | 0.66715300 |
| H | -5.50118500 | -1.60419400 | -0.00487000 |
| C | 2.63240200 | 0.09746600 | -0.36425200 |
| C | 2.72836000 | -0.93493500 | 0.82093400 |
| O | 2.43286600 | -0.50802700 | 1.96939500 |
| O | 3.15747900 | -2.08582300 | 0.52434000 |
| C | 2.56846000 | -0.55856300 | -1.74445200 |
| H | 2.47701600 | 0.21429400 | -2.51707000 |
| H | 3.47592500 | -1.13669500 | -1.92871100 |
| H | 1.71375500 | -1.23430700 | -1.81943900 |
| C | 3.82154900 | 1.05958000 | -0.26935900 |
| H | 3.82986100 | 1.56474500 | 0.70005300 |
| H | 4.75692600 | 0.50272900 | -0.38071500 |
| H | 3.77160300 | 1.81282900 | -1.06374800 |

12

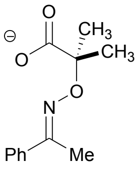


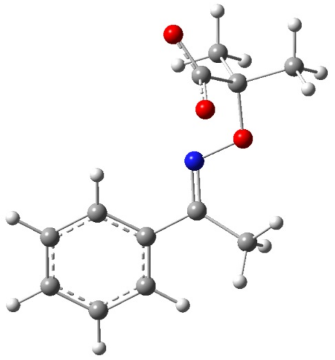

Cartesian Coordinates

| | | | |
|---|---|---|---|
| N | 0.28523200 | 0.28029800 | 0.17961400 |
| C | -0.75913100 | 0.97168300 | -0.13274200 |
| O | 1.46864700 | 1.00261400 | 0.09507900 |

| | | | |
|---|---|---|---|
| C | -0.71610600 | 2.41987400 | -0.55409800 |
| H | -1.47350900 | 2.62822800 | -1.31377700 |
| H | -0.90854100 | 3.08152700 | 0.30008800 |
| H | 0.26780100 | 2.66795400 | -0.95193000 |
| C | -2.06375300 | 0.26033400 | -0.03861200 |
| C | -2.12574100 | -1.14723000 | -0.00351300 |
| C | -3.27046400 | 0.98024100 | 0.02292300 |
| C | -3.34959500 | -1.80627000 | 0.09488700 |
| H | -1.20328800 | -1.71333500 | -0.06110400 |
| C | -4.49781600 | 0.31745100 | 0.12457600 |
| H | -3.25947400 | 2.06496000 | 0.00350300 |
| C | -4.54439900 | -1.07783800 | 0.16008700 |
| H | -3.37300900 | -2.89234200 | 0.11275100 |
| H | -5.41604600 | 0.89583100 | 0.17571700 |
| H | -5.49765900 | -1.59345300 | 0.23292400 |
| C | 2.62335200 | 0.15877700 | 0.39260300 |
| C | 2.54707600 | -0.31222900 | 1.84584600 |
| C | 2.73369800 | -1.01951100 | -0.64412600 |
| C | 3.81195000 | 1.10417100 | 0.18688900 |
| H | 3.74482700 | 1.96035000 | 0.86773200 |
| H | 4.74563500 | 0.57220300 | 0.39274200 |
| H | 3.83927100 | 1.47049100 | -0.84265300 |
| H | 3.45126900 | -0.86470200 | 2.10864300 |
| H | 2.45250900 | 0.55399000 | 2.51148400 |
| H | 1.68935500 | -0.96984800 | 2.00173100 |
| O | 3.19451600 | -2.10970800 | -0.20128100 |
| O | 2.41306300 | -0.75798800 | -1.83423200 |

13

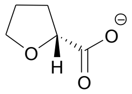


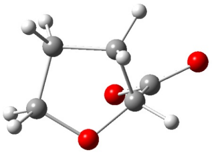

Cartesian Coordinates

| | | | |
|---|---|---|---|
| C | 1.79841500 | -0.75564300 | 0.04302400 |
| O | 0.80671000 | -0.74493400 | -1.00291400 |
| C | -0.15716700 | 0.29041800 | -0.75260400 |
| C | 0.64459100 | 1.36623400 | 0.00249900 |
| C | 1.57469900 | 0.51620600 | 0.88731900 |
| H | 1.68988900 | -1.66787800 | 0.64065300 |
| H | 2.78411500 | -0.76857800 | -0.43848100 |
| H | 1.22764000 | 1.95983300 | -0.71136500 |
| H | 0.00457800 | 2.04486500 | 0.57125900 |
| H | 2.51380300 | 1.02537700 | 1.12412000 |
| H | 1.07961200 | 0.26686300 | 1.83049200 |
| C | -1.41035800 | -0.17275800 | 0.04647900 |
| O | -2.38091900 | 0.64137600 | 0.00371200 |
| O | -1.36333800 | -1.26785900 | 0.66836000 |
| H | -0.50033900 | 0.64410400 | -1.73024200 |

14

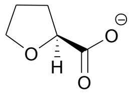


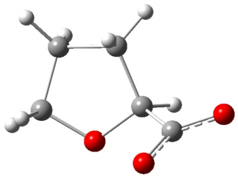

Cartesian Coordinates

| | | | |
|---|---|---|---|
| C | 1.83715500 | -0.83719100 | -0.09988500 |
| O | 0.69559500 | -0.92323000 | 0.76215000 |
| C | -0.14973600 | 0.23343800 | 0.57274200 |
| C | 0.64360600 | 1.19654800 | -0.35075900 |
| C | 2.08266000 | 0.66091900 | -0.27780700 |

| | | | |
|---|---|---|---|
| H | 2.66182800 | -1.37258800 | 0.38090500 |
| H | 1.62121400 | -1.31761800 | -1.06622500 |
| H | -0.30089100 | 0.67843200 | 1.56301000 |
| H | 0.26945700 | 1.12743700 | -1.37839800 |
| H | 0.55031200 | 2.23689900 | -0.02936000 |
| H | 2.67271200 | 0.89244100 | -1.17029000 |
| H | 2.60643500 | 1.06423600 | 0.59670900 |
| C | -1.54442000 | -0.09461800 | -0.02148300 |
| O | -1.67326200 | -1.13099200 | -0.72617100 |
| O | -2.43441500 | 0.77124500 | 0.23487100 |

## 9 NMR Spectra

**3a** – $^{1}$H NMR (400 MHz, $CDCl_3$)

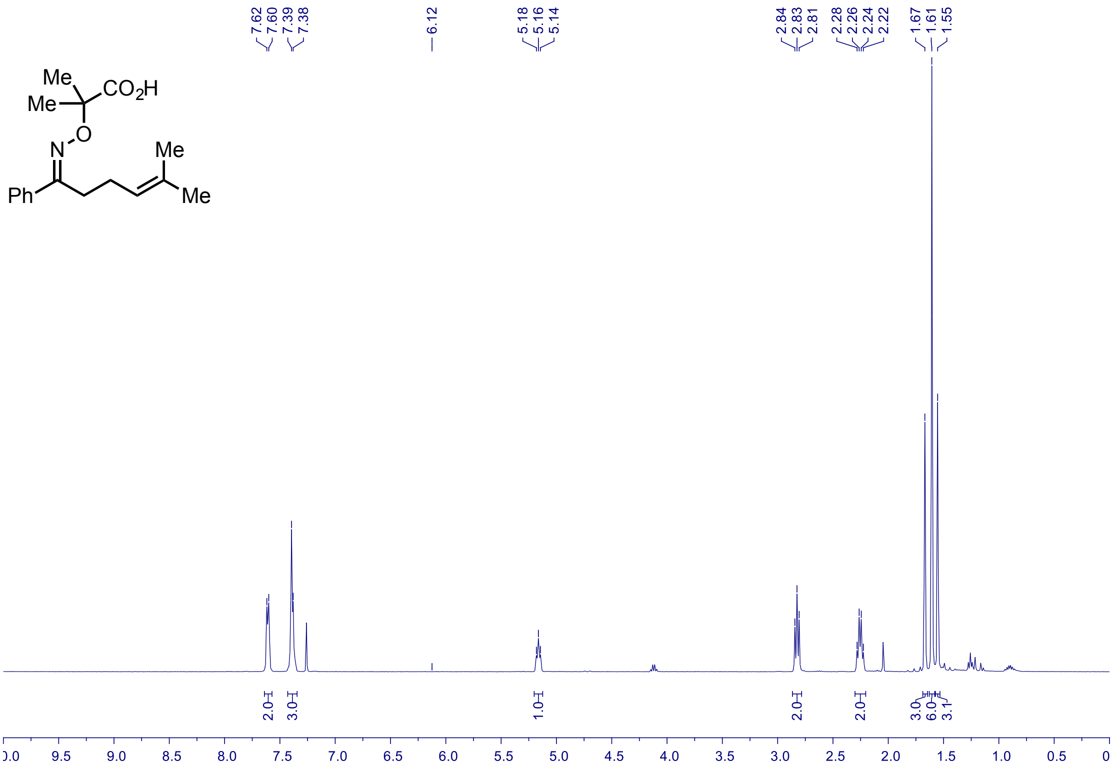

**3a** – $^{13}$C NMR (101 MHz, $CDCl_3$)

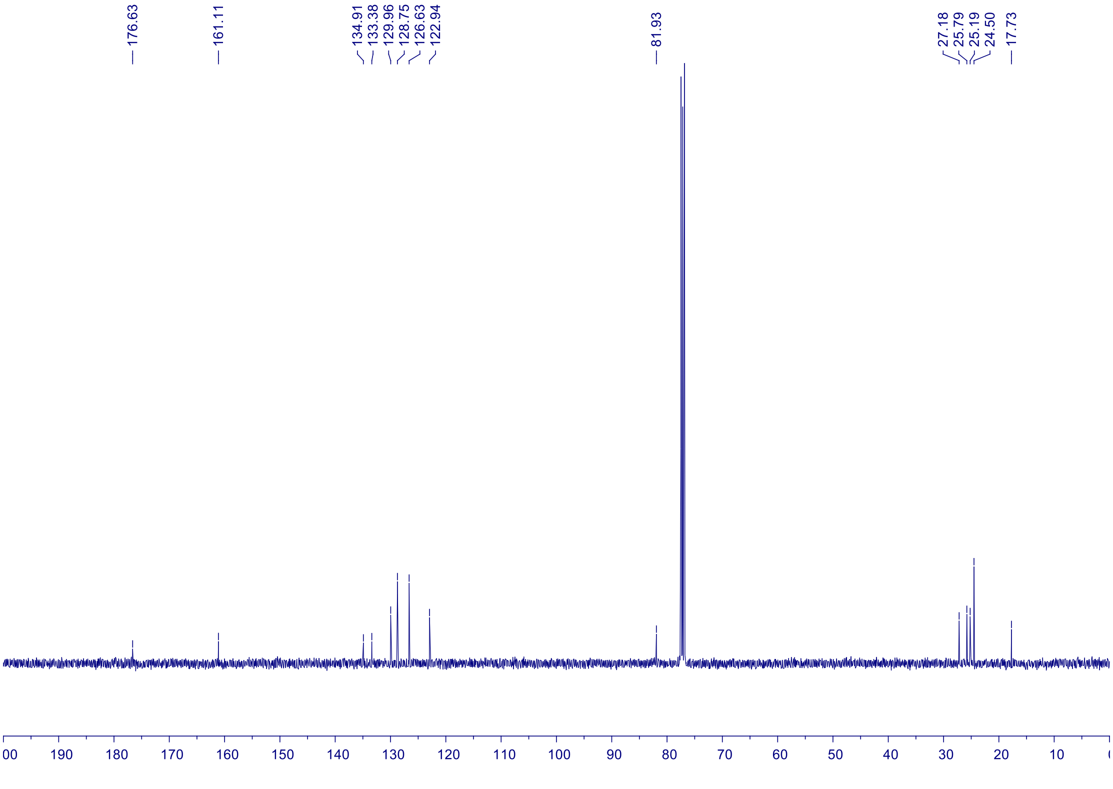

**3b** – $^{1}$H NMR (400 MHz, $CDCl_3$)

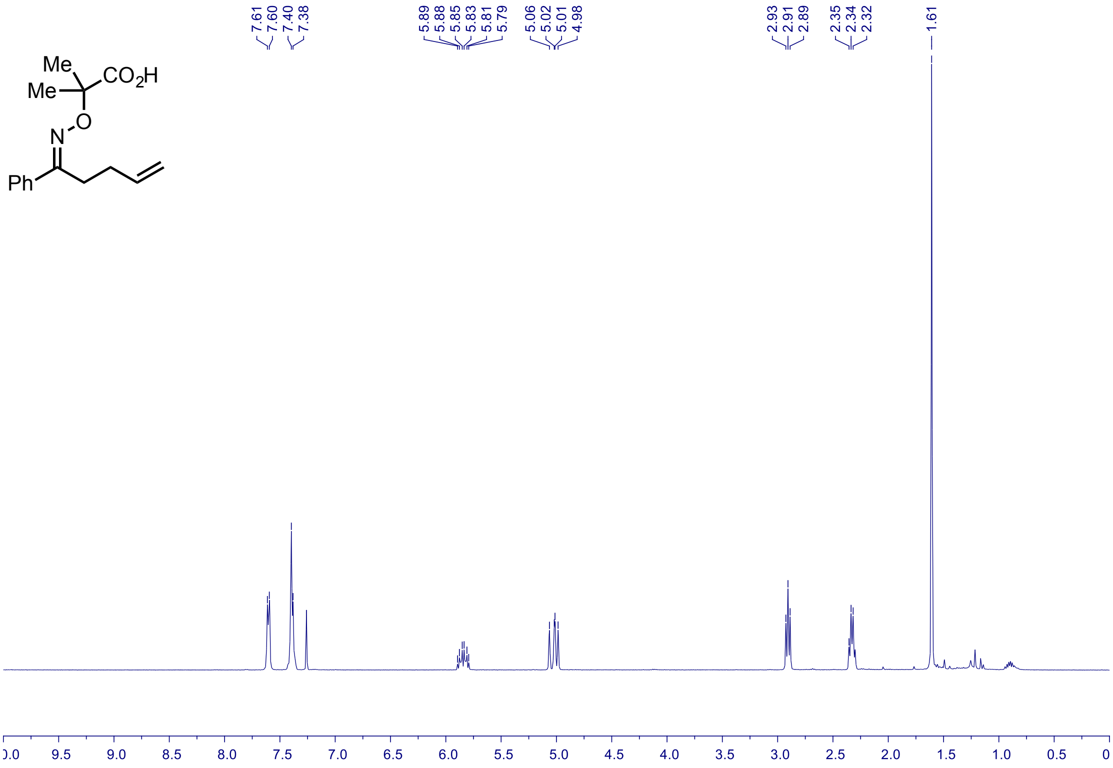


**3b** – $^{13}$C NMR (101 MHz, $CDCl_3$)

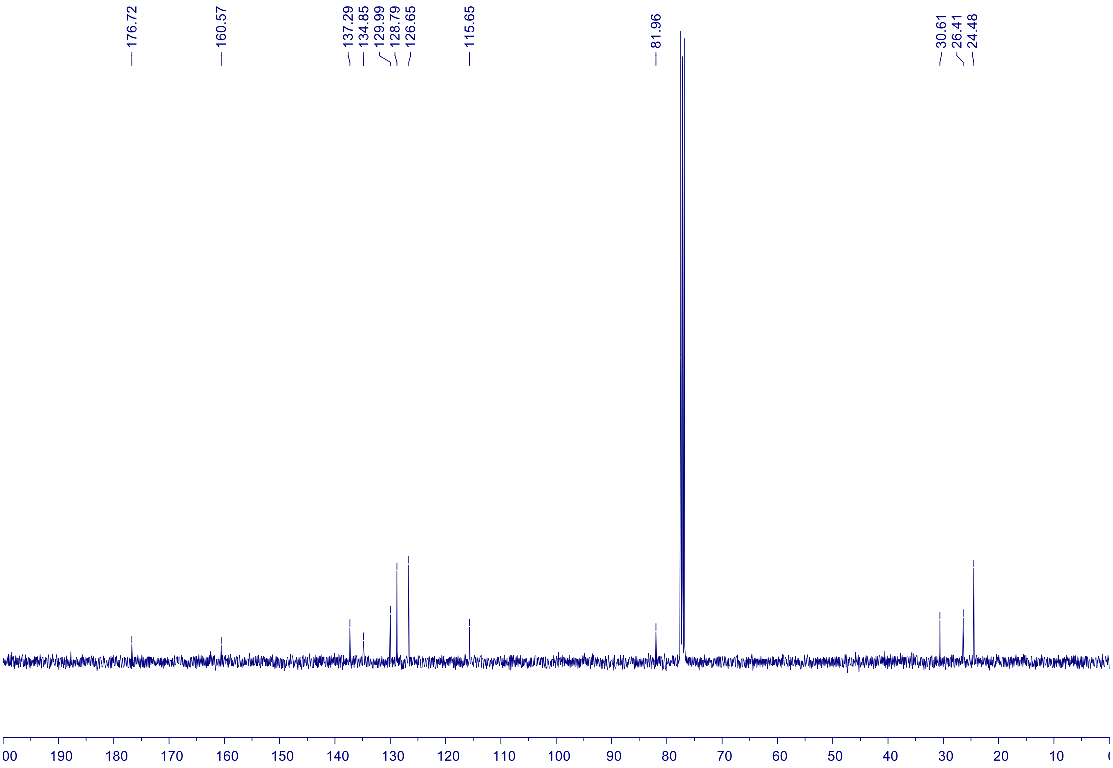

**3c** – $^{1}$H NMR (500 MHz, $CDCl_3$)

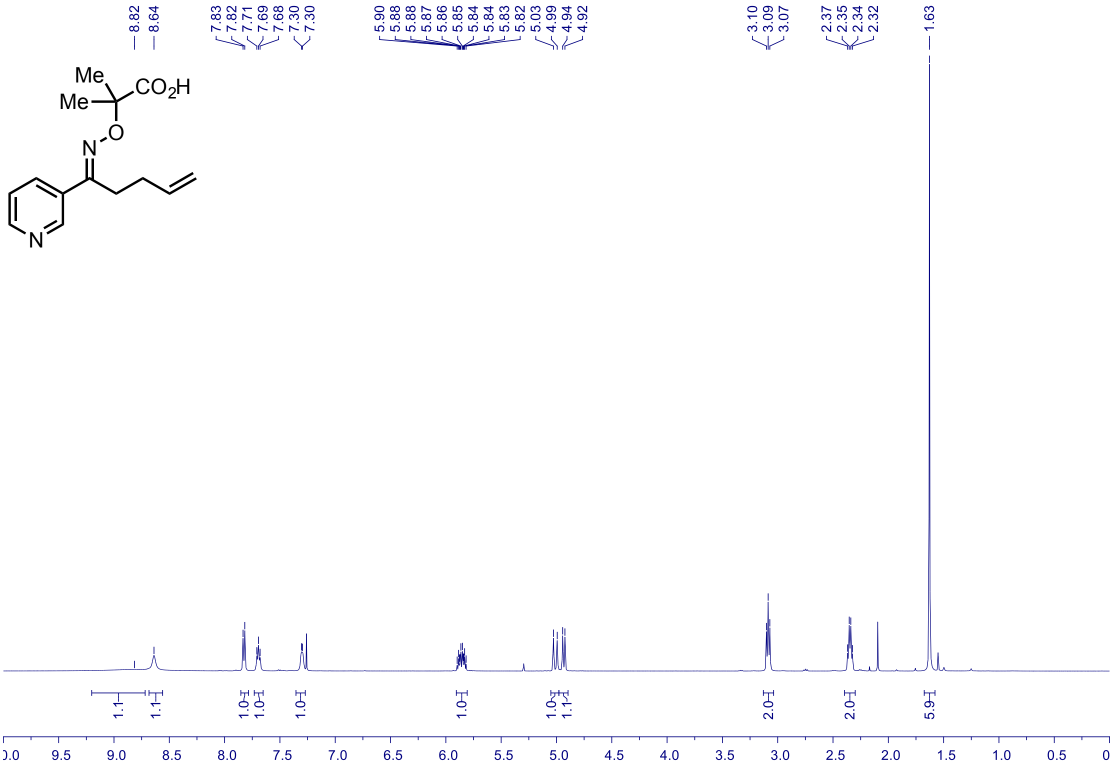


**3c** – $^{13}$C NMR (126 MHz, $CDCl_3$)

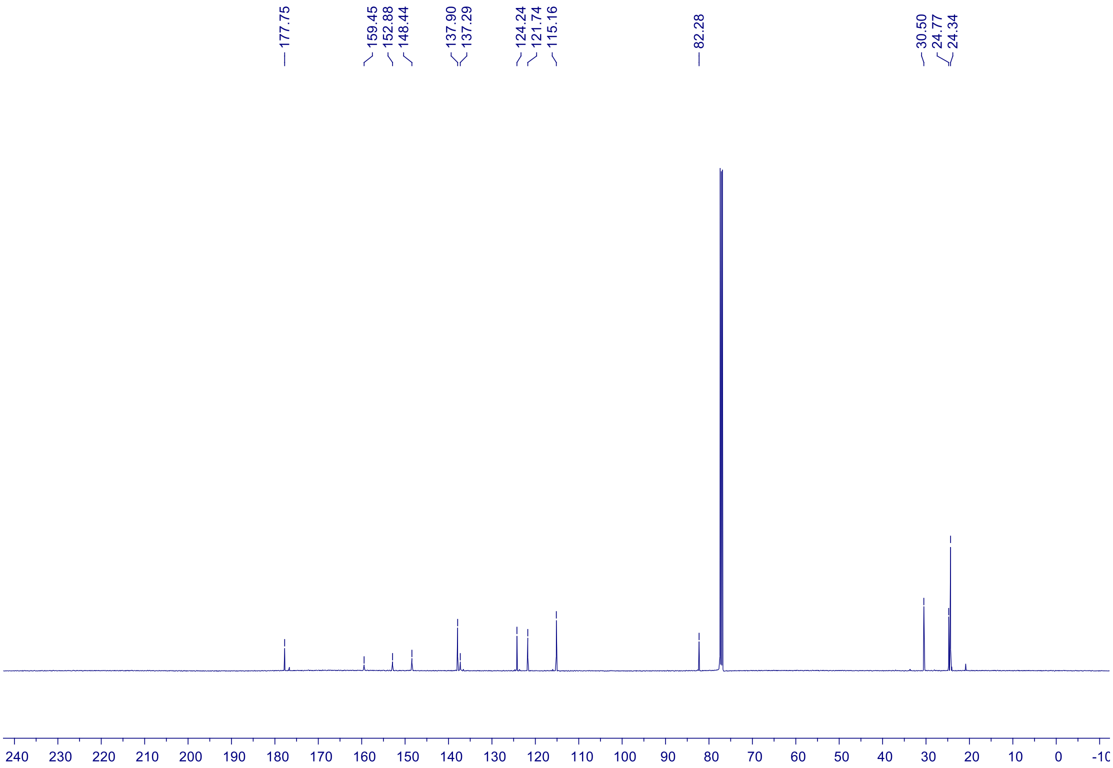

**3d** – $^{1}$H NMR (400 MHz, $CDCl_3$)

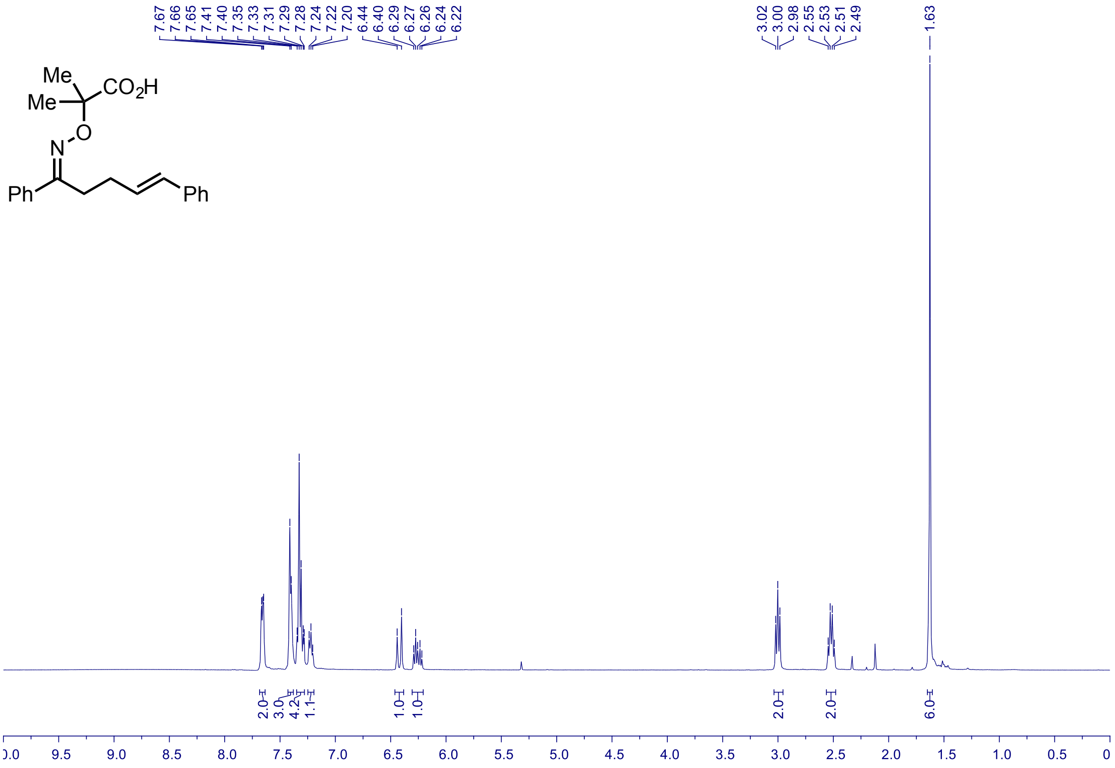


**3d** – $^{13}$C NMR (101 MHz, $CDCl_3$)

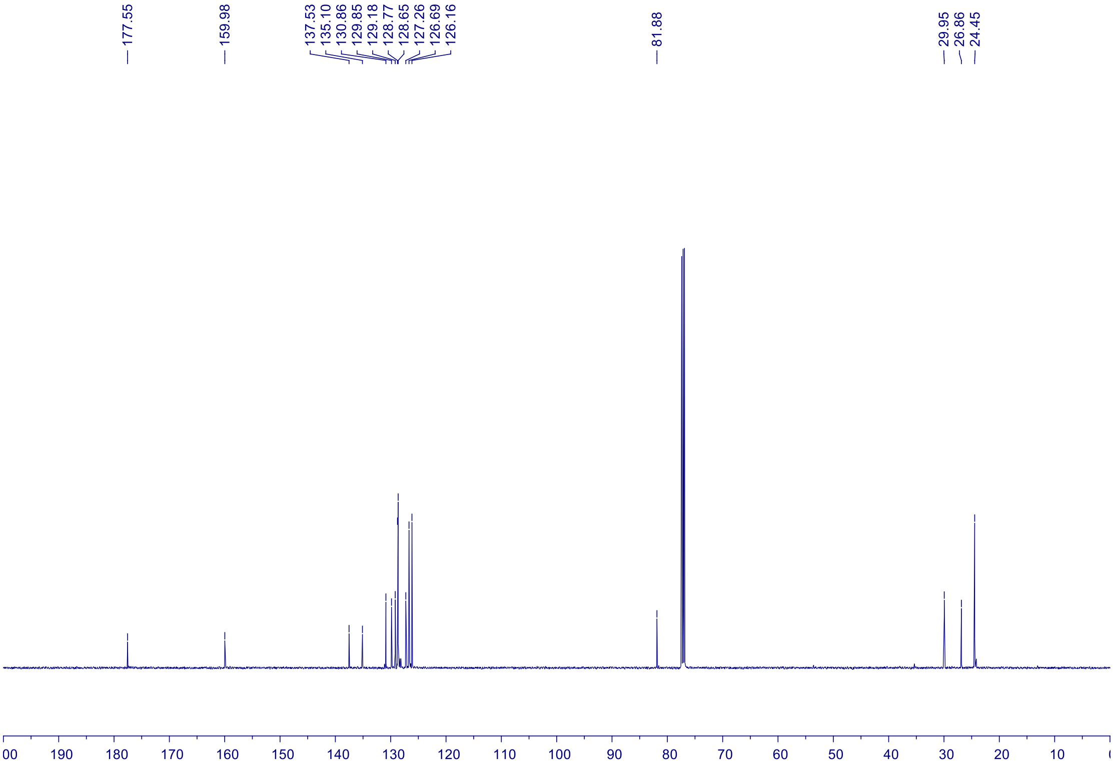

**3e** – $^{1}$H NMR (400 MHz, $CDCl_3$)

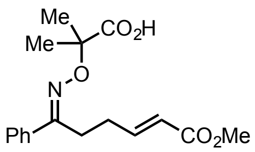

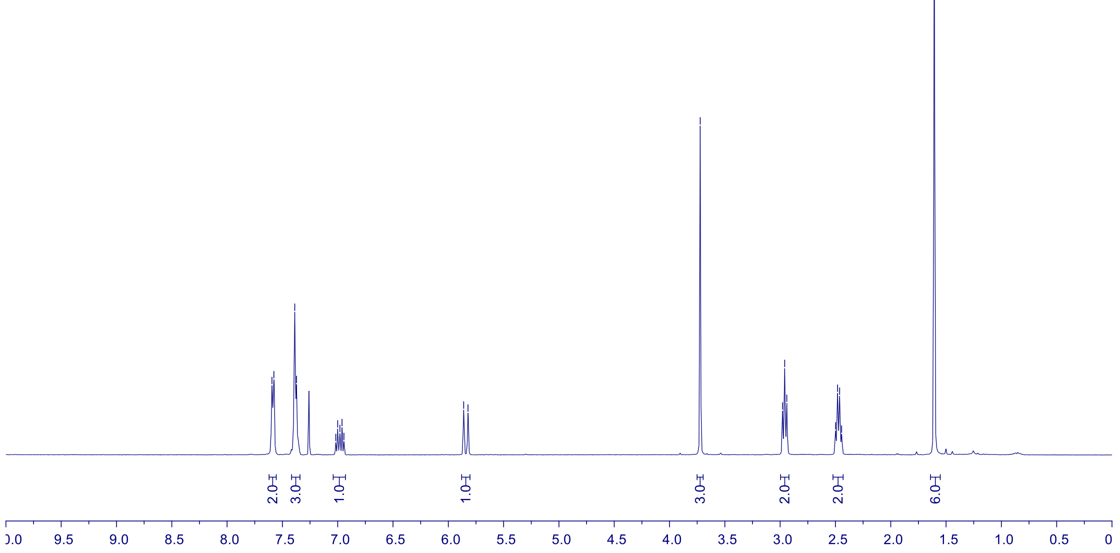


**3e** – $^{13}$C NMR (101 MHz, $CDCl_3$)

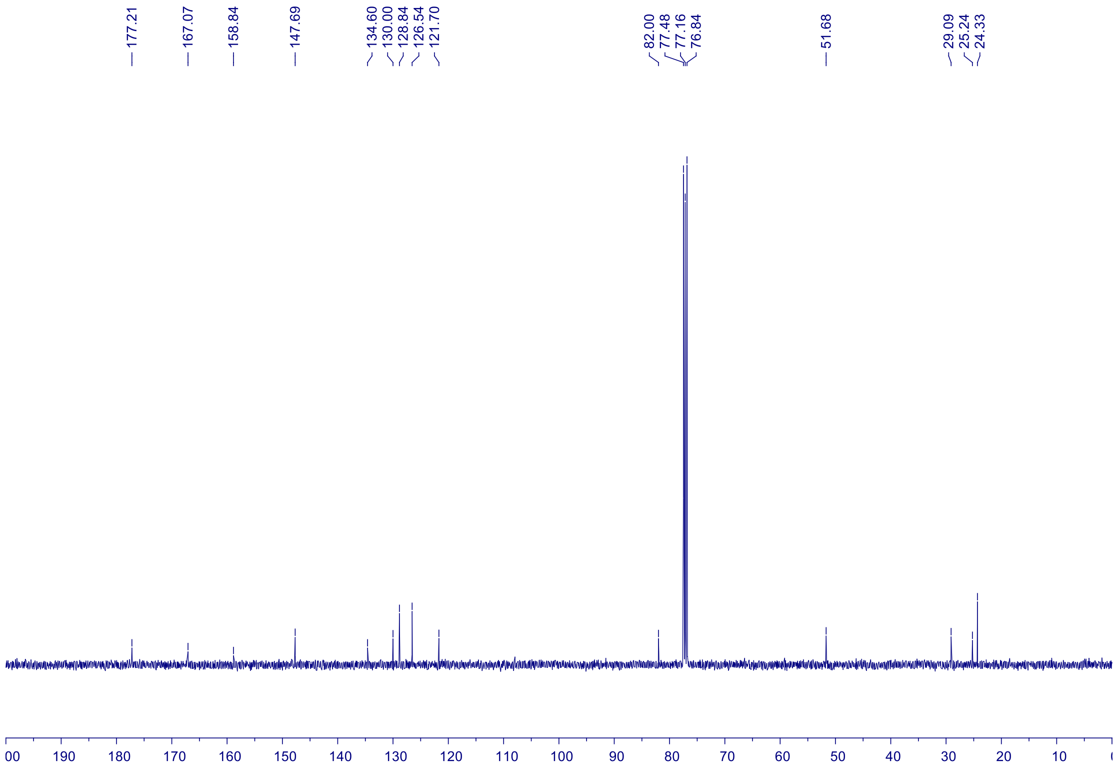

**3f** – $^{1}$H NMR (400 MHz, $CDCl_3$)

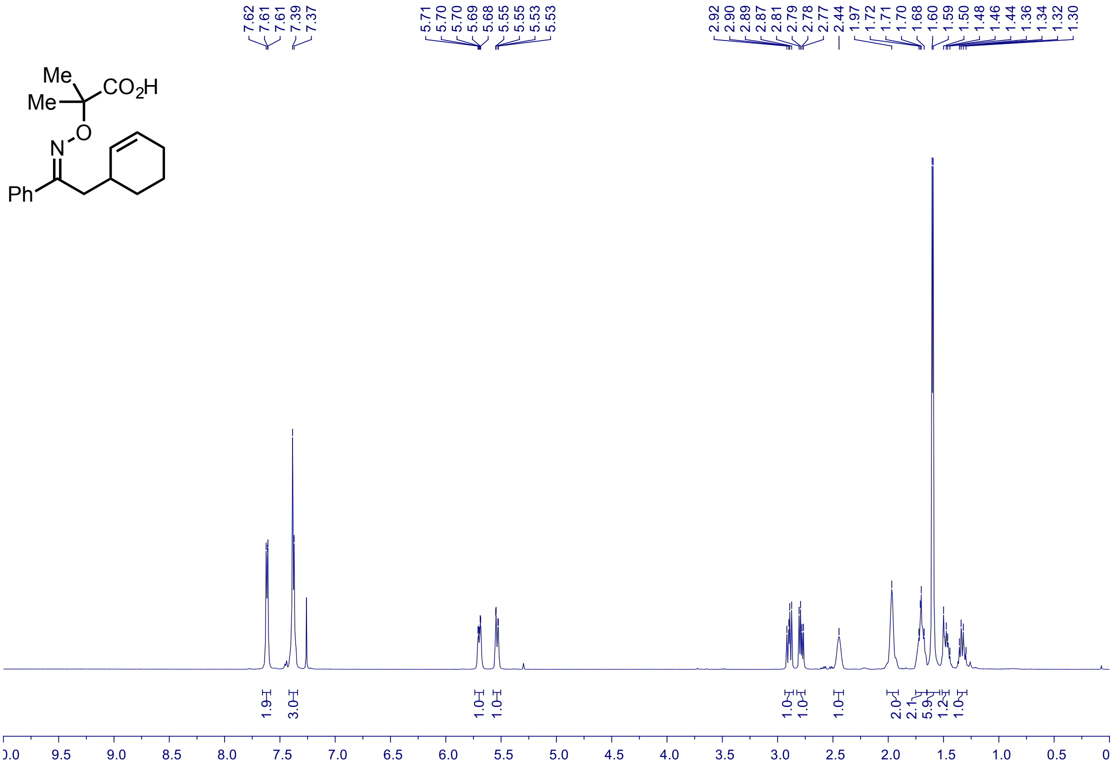


**3f** – $^{13}$C NMR (101 MHz, $CDCl_3$)

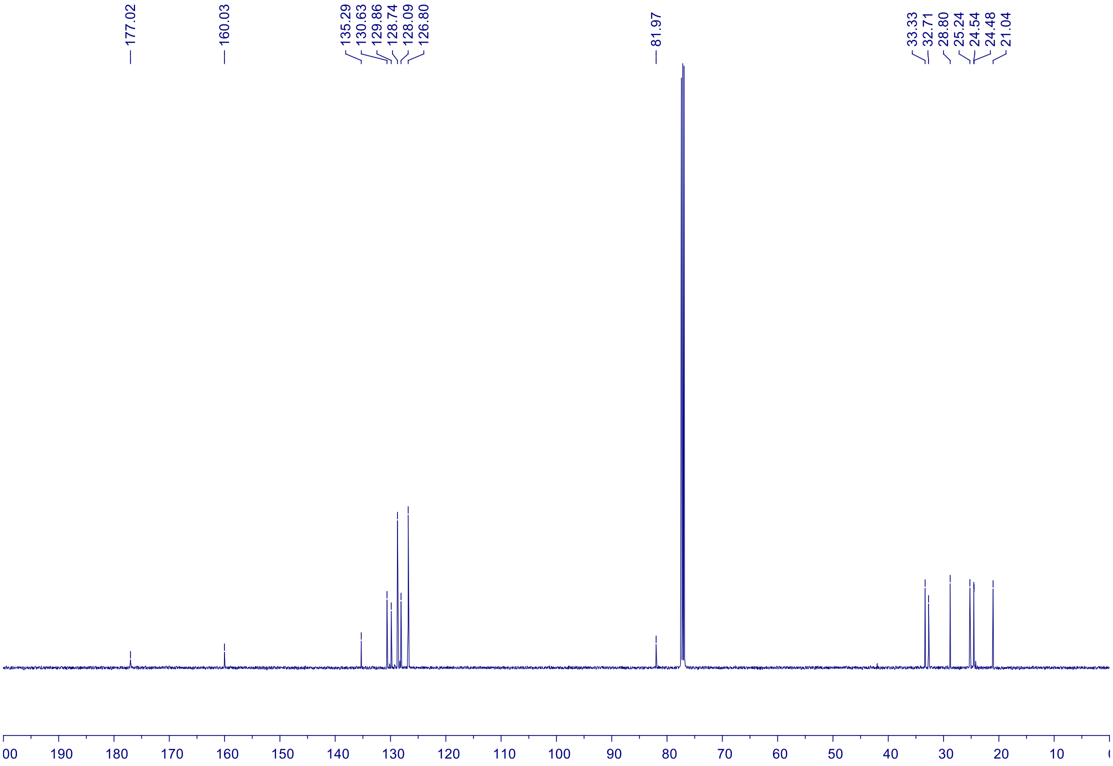

**3g** – $^{1}$H NMR (400 MHz, $CDCl_3$)

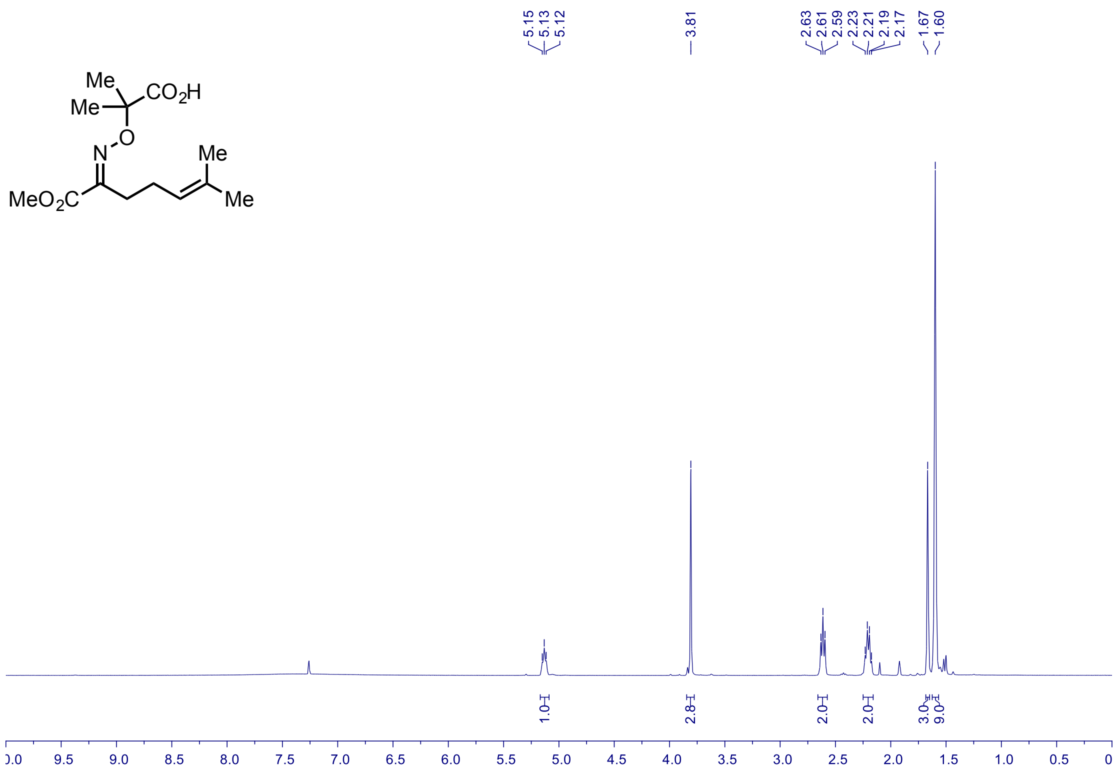


**3g** – $^{13}$C NMR (101 MHz, $CDCl_3$)

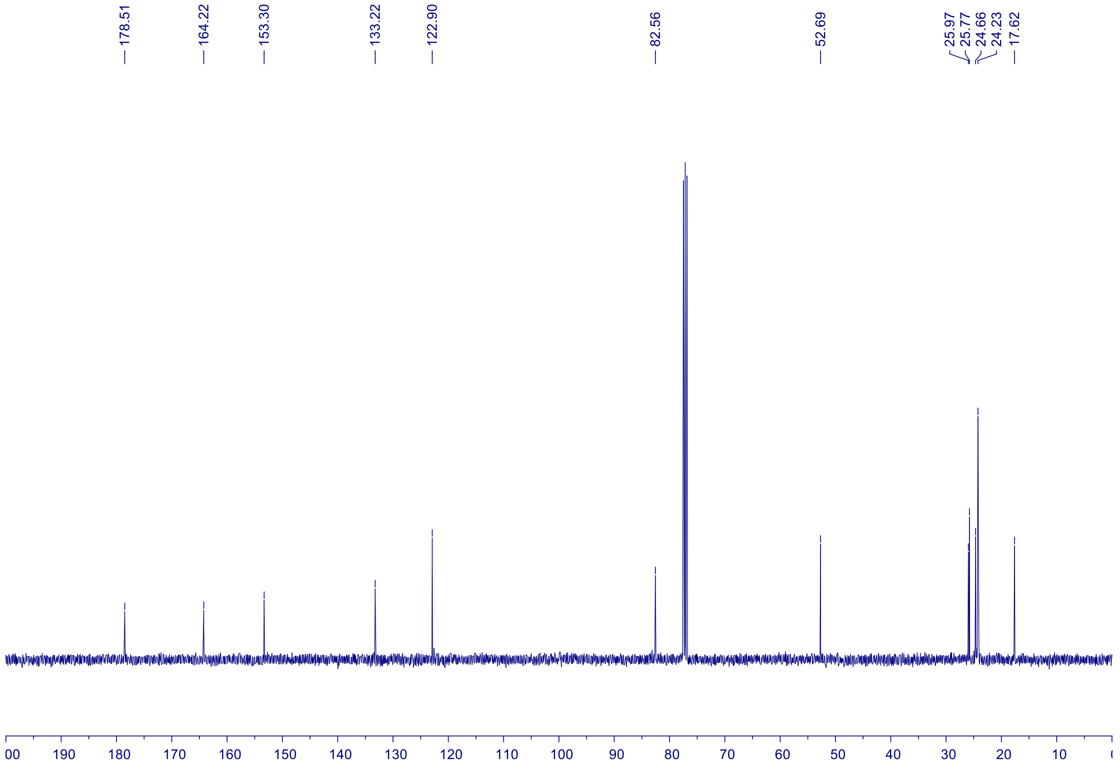

**3h** – $^{1}$H NMR (400 MHz, $CDCl_3$)

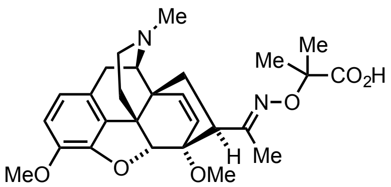


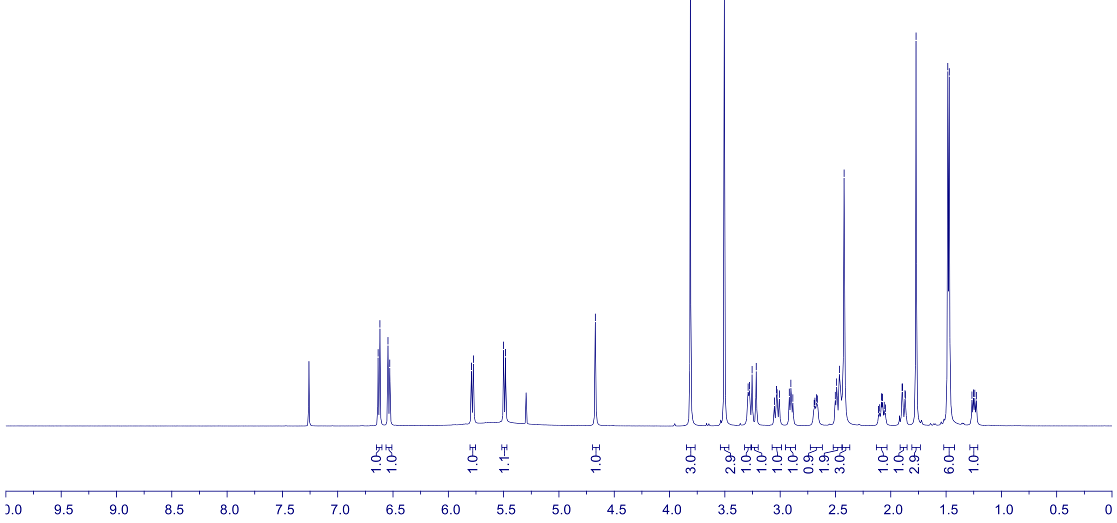


**3h** – $^{13}$C NMR (101 MHz, $CDCl_3$)

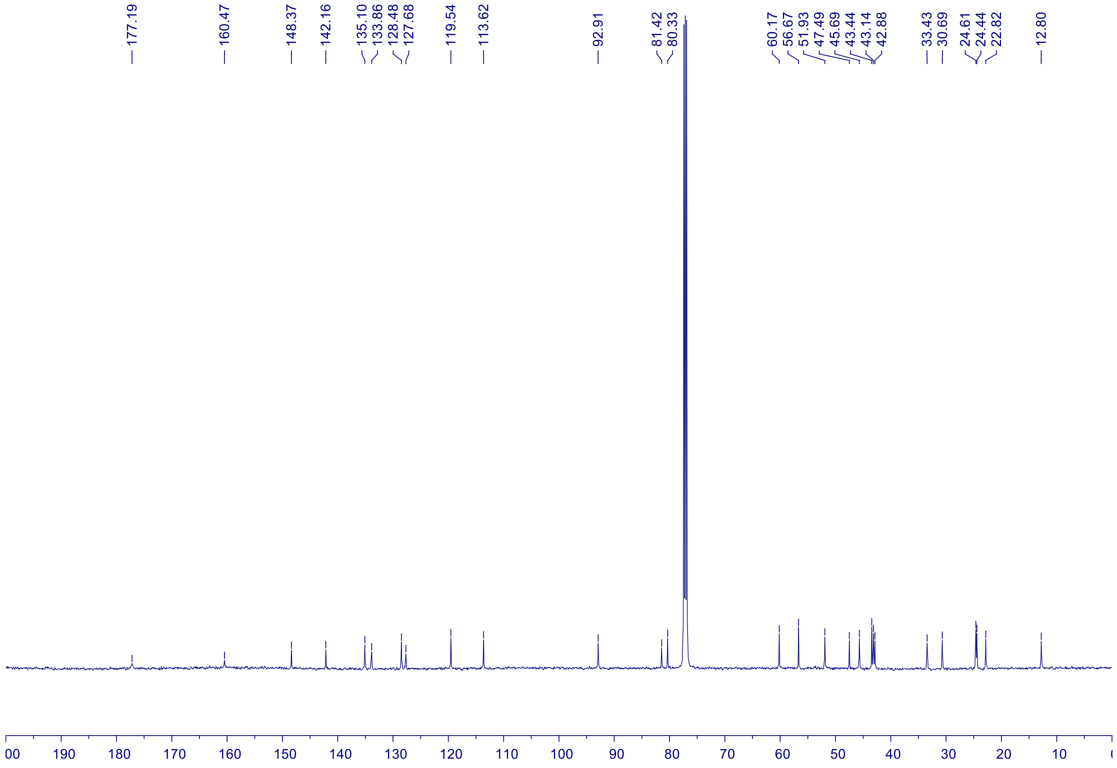

**2b** – $^{1}H$ NMR (400 MHz, $CDCl_3$)

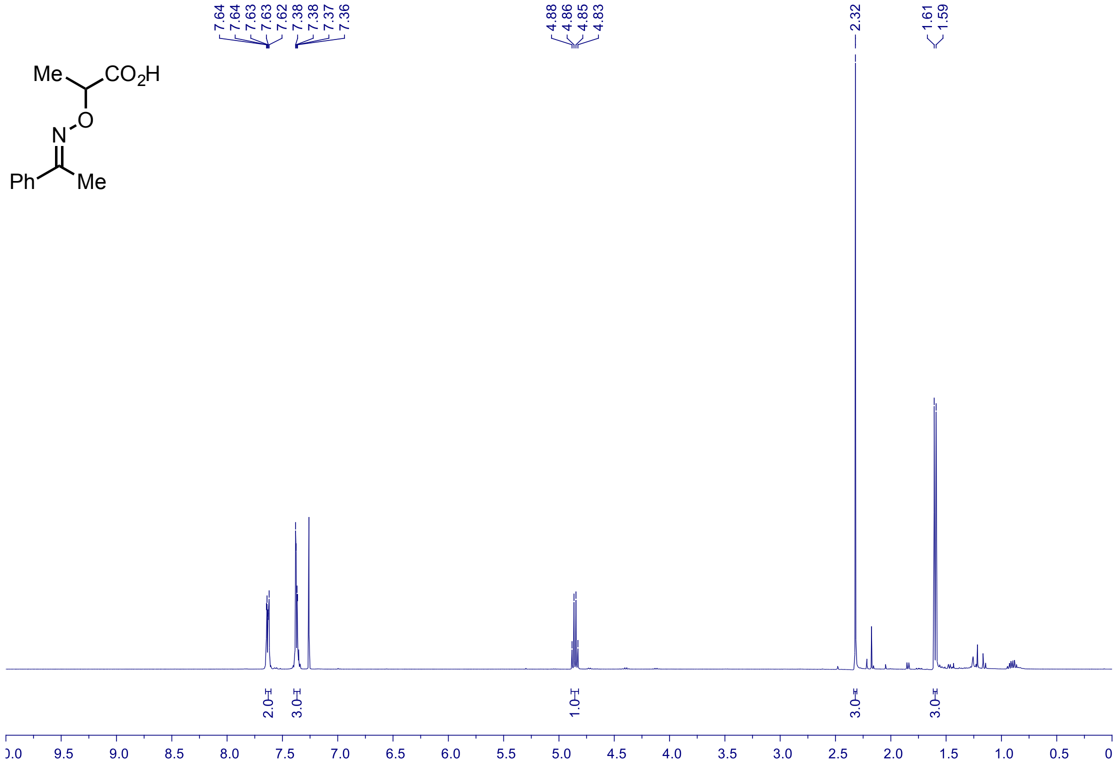


**2b** – $^{13}C$ NMR (101 MHz, $CDCl_3$)

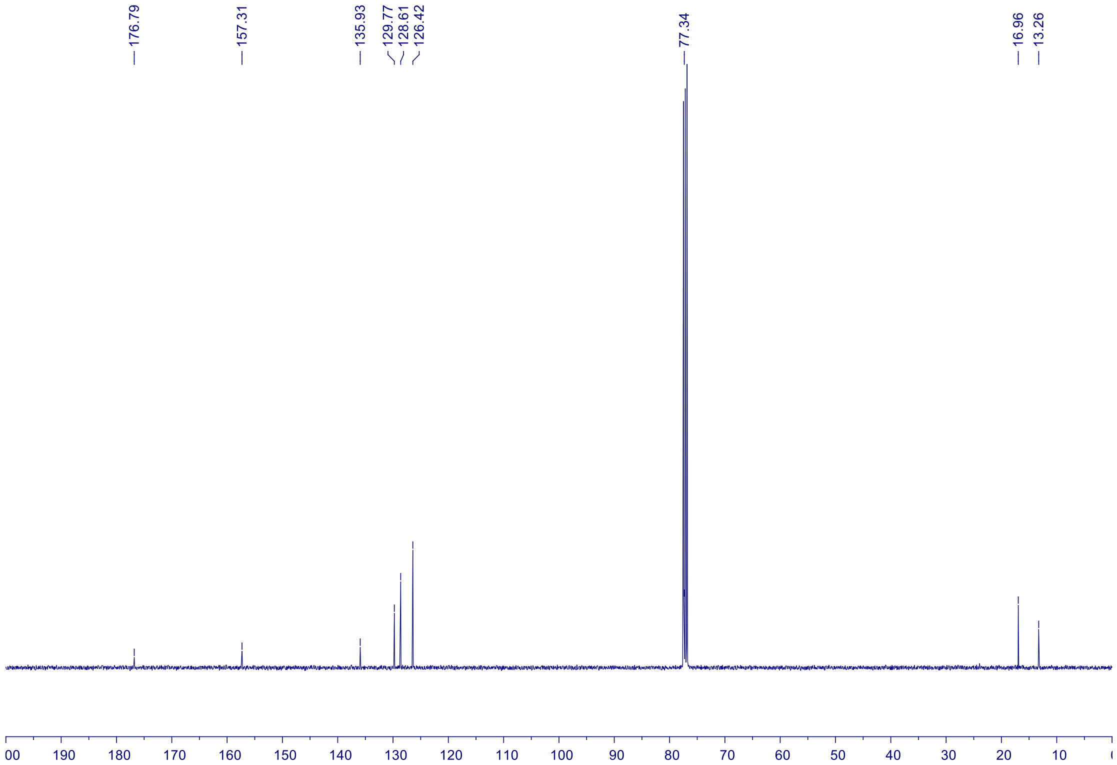

**2c** – $^{1}$H NMR (400 MHz, CDCl$_3$)

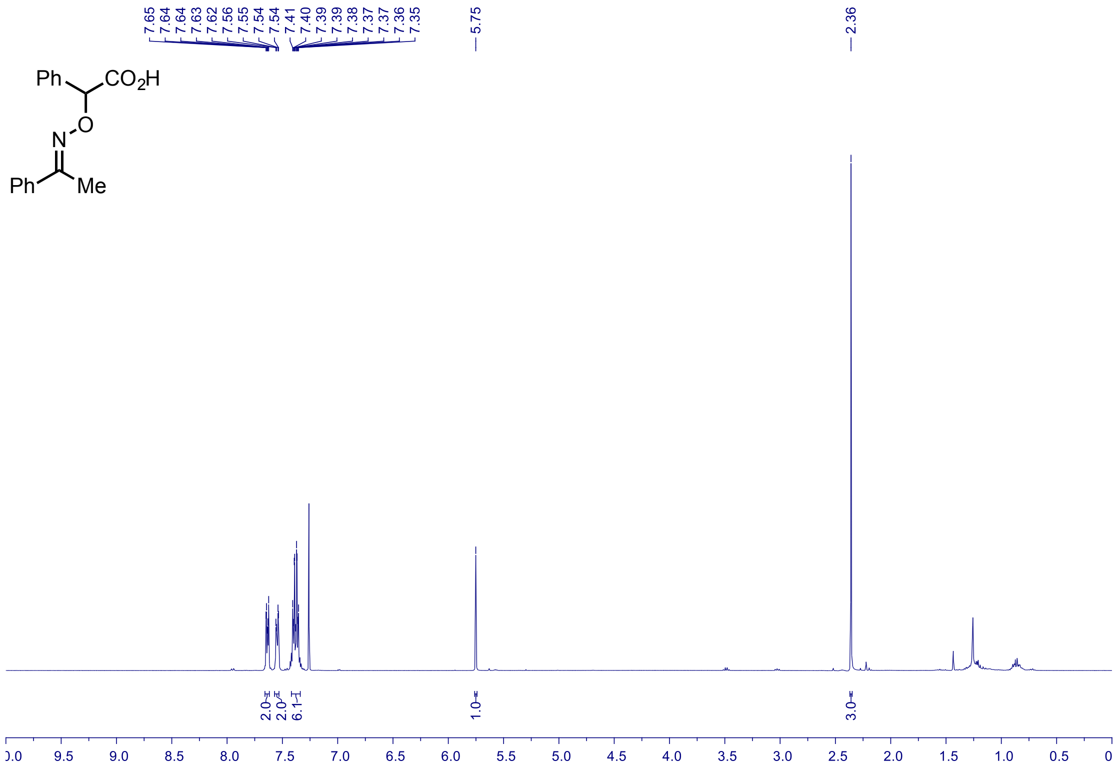

**2c** – $^{13}$C NMR (101 MHz, CDCl$_3$)

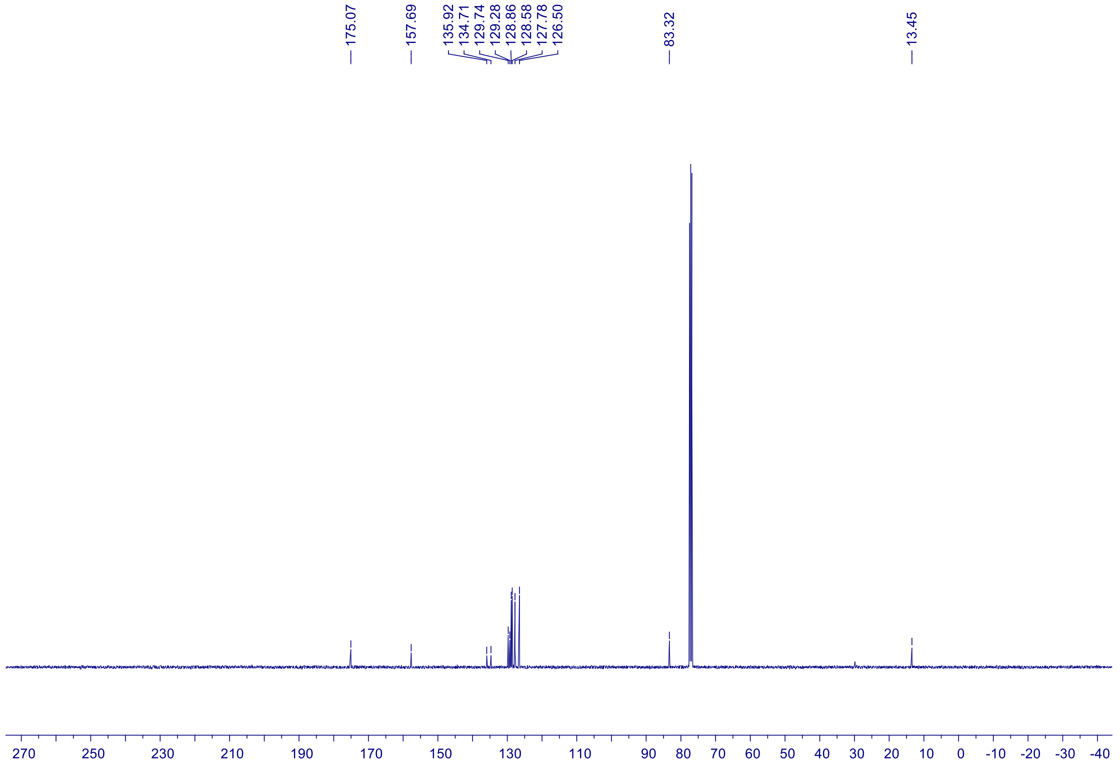

**2d** – $^{1}$H NMR (400 MHz, $CDCl_3$)

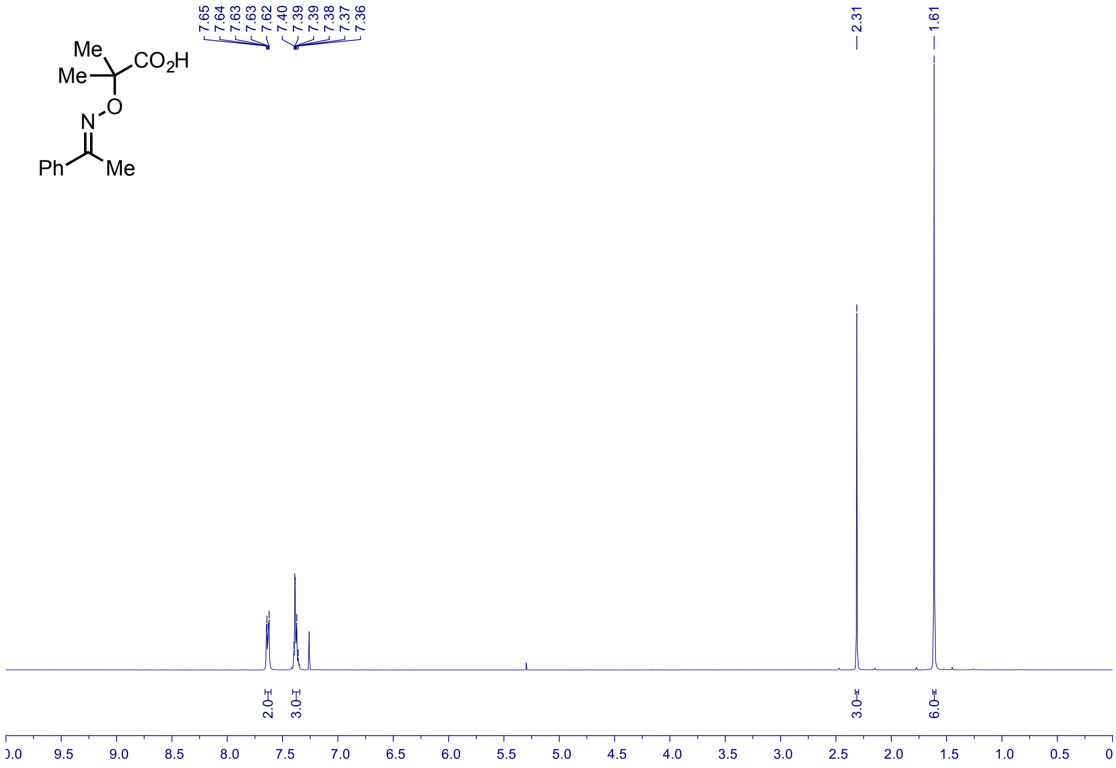


**2d** – $^{13}$C NMR (101 MHz, $CDCl_3$)

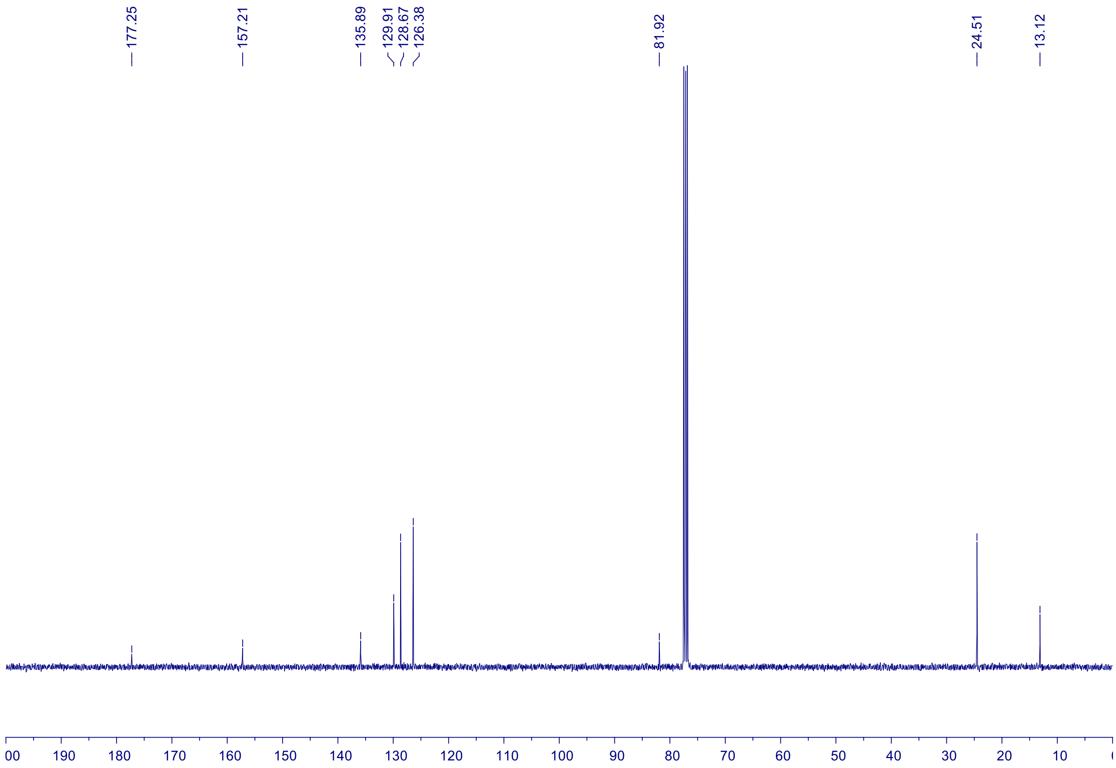

**5a** – $^{1}$H NMR (400 MHz, $CDCl_3$)

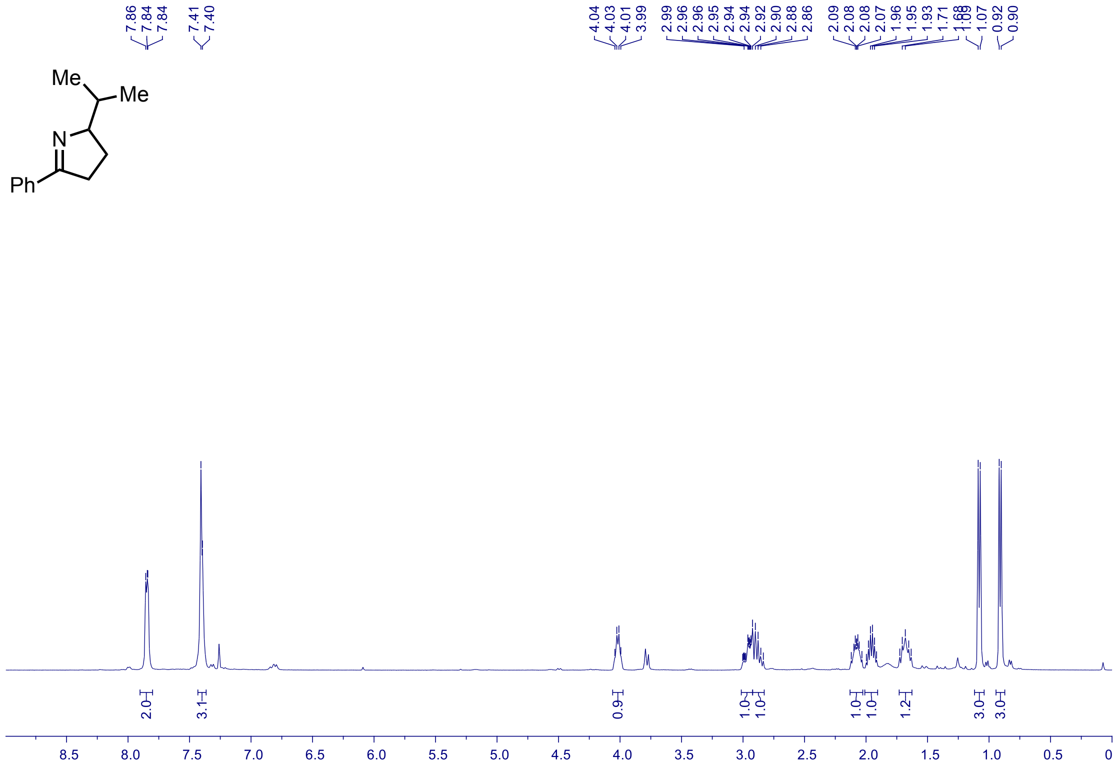


**5a** – $^{13}$C NMR (101 MHz, $CDCl_3$)

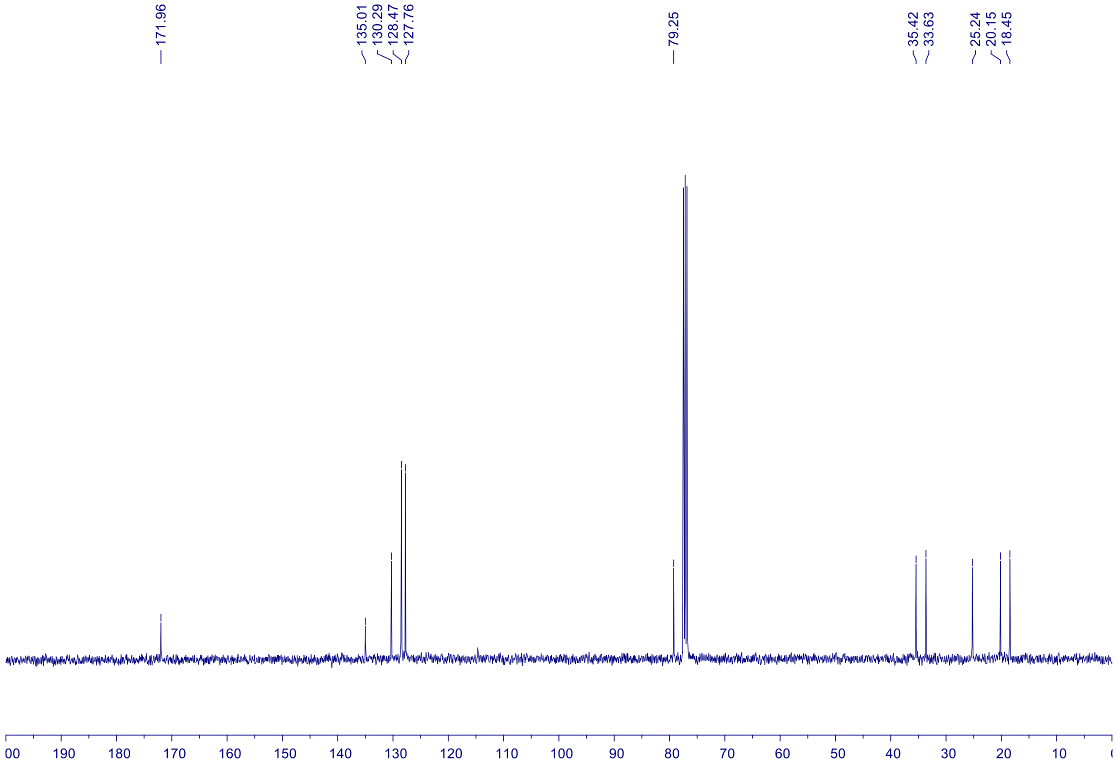

**5b** – $^1$H NMR (400 MHz, $CDCl_3$)

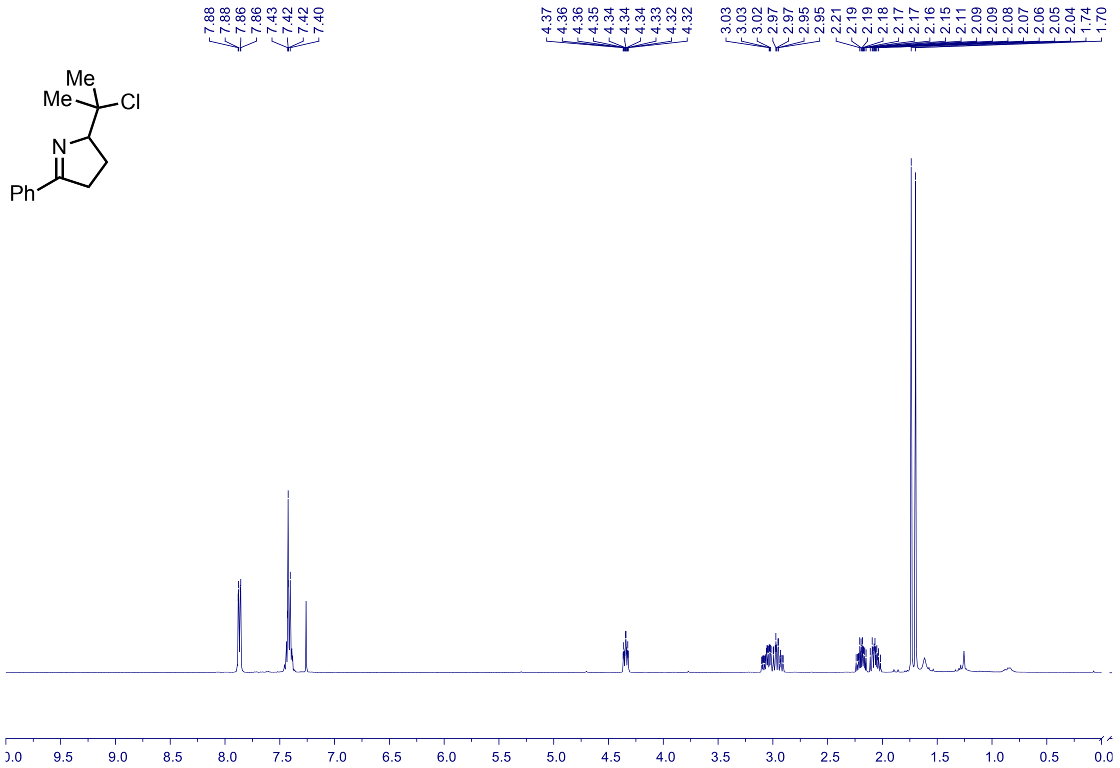


**5b** – $^{13}$C NMR (101 MHz, $CDCl_3$)

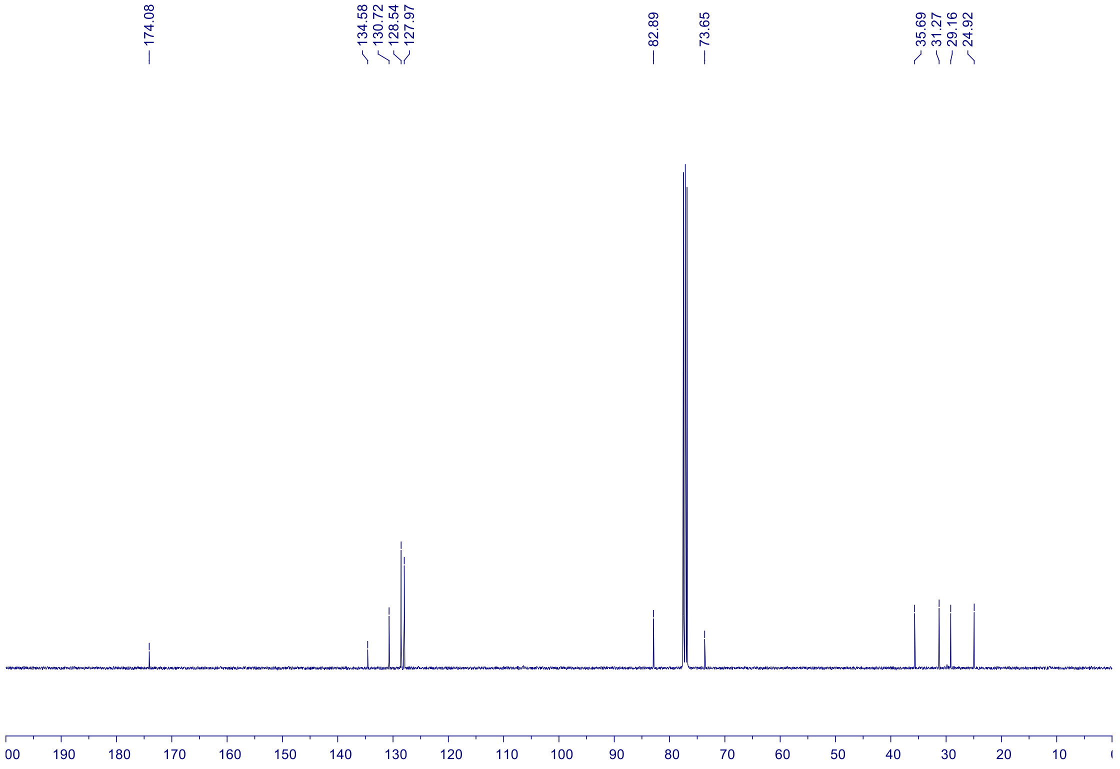

**5c** – $^{1}$H NMR (400 MHz, $CDCl_3$)

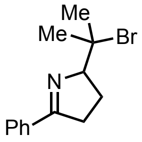

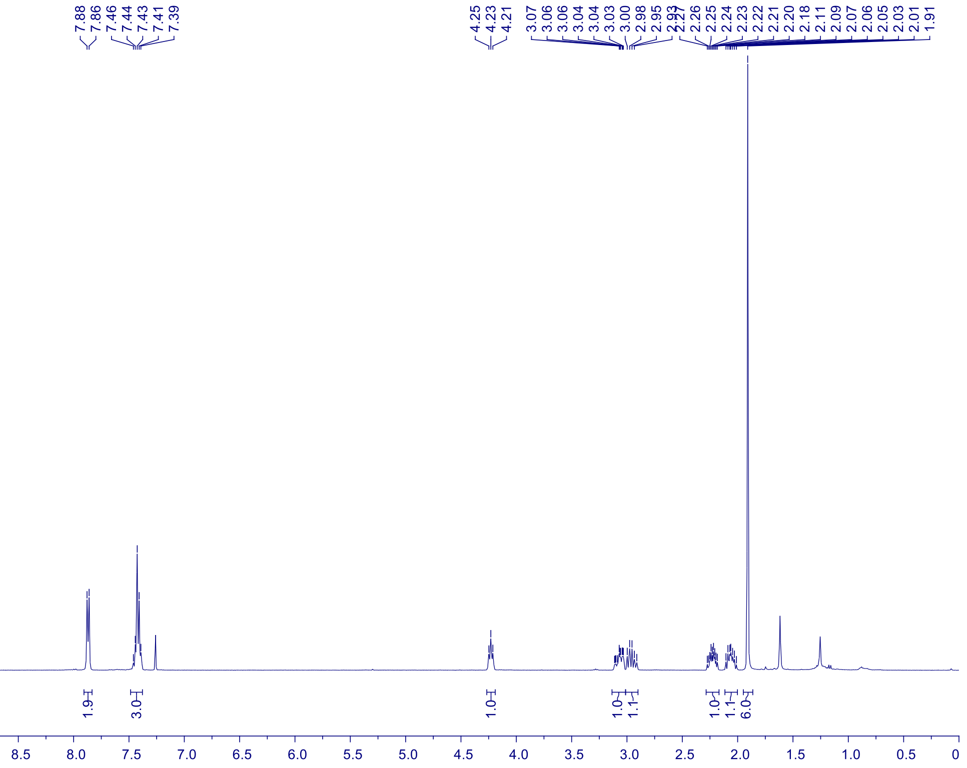

**5c** – $^{13}$C NMR (101 MHz, $CDCl_3$)

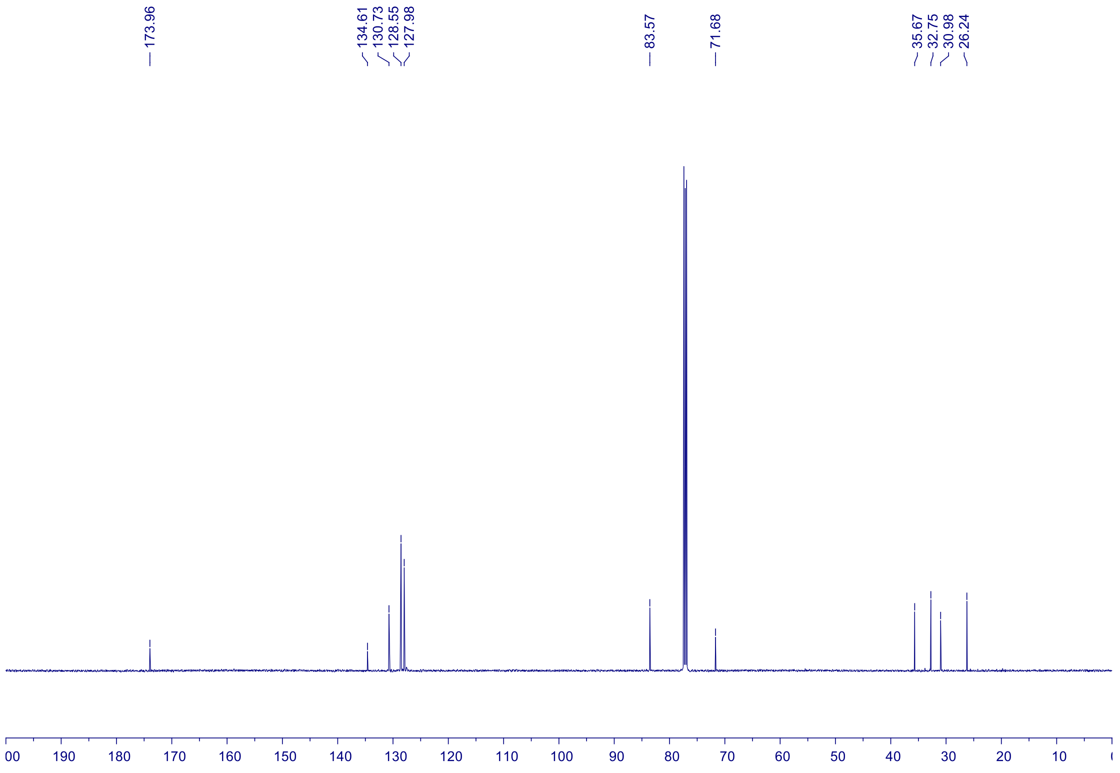

**5d** – $^1$H NMR (400 MHz, $CDCl_3$)

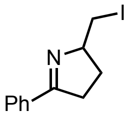

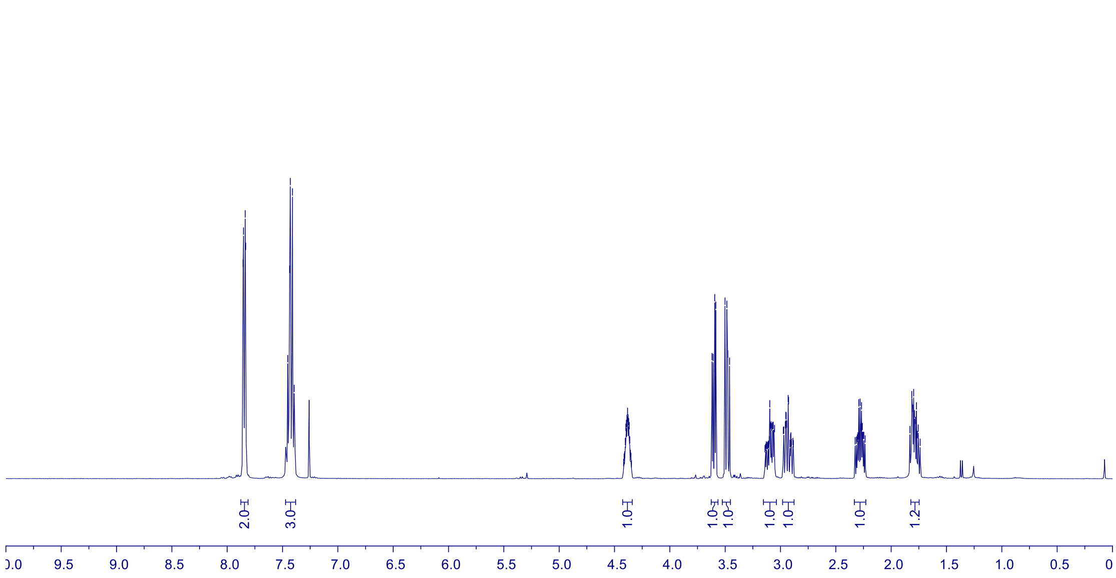

**5d** – $^{13}$C NMR (101 MHz, $CDCl_3$)

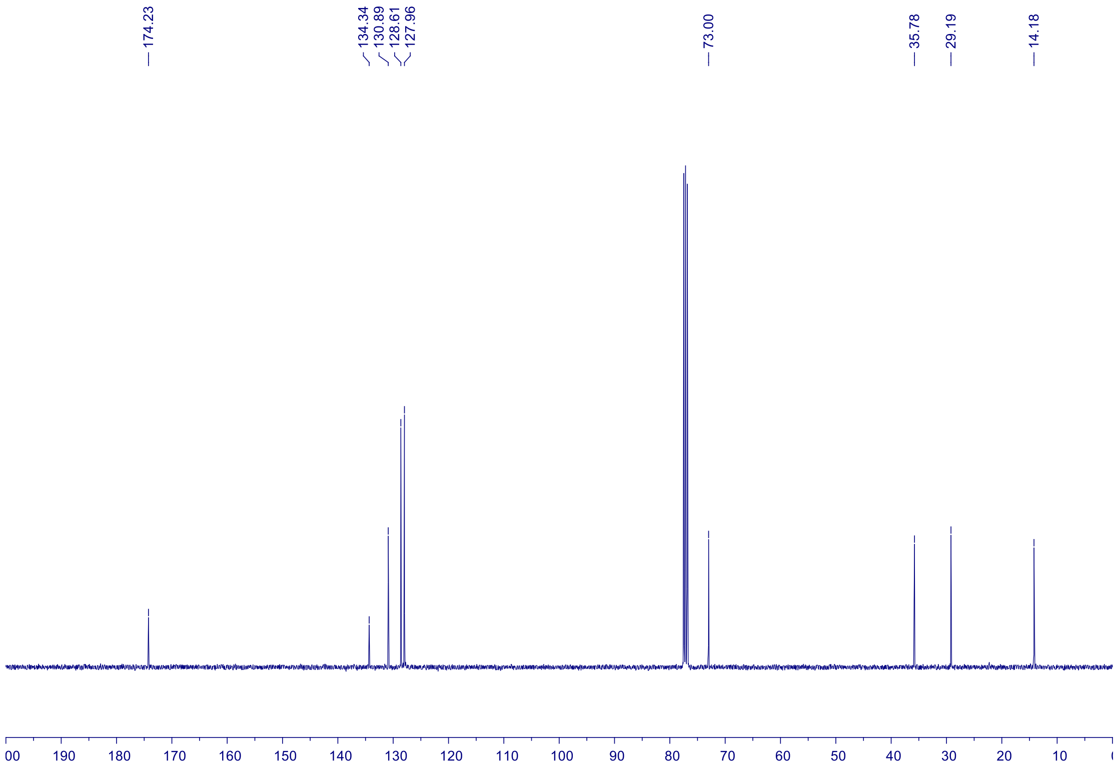

**5e** – $^1$H NMR (400 MHz, $CDCl_3$)

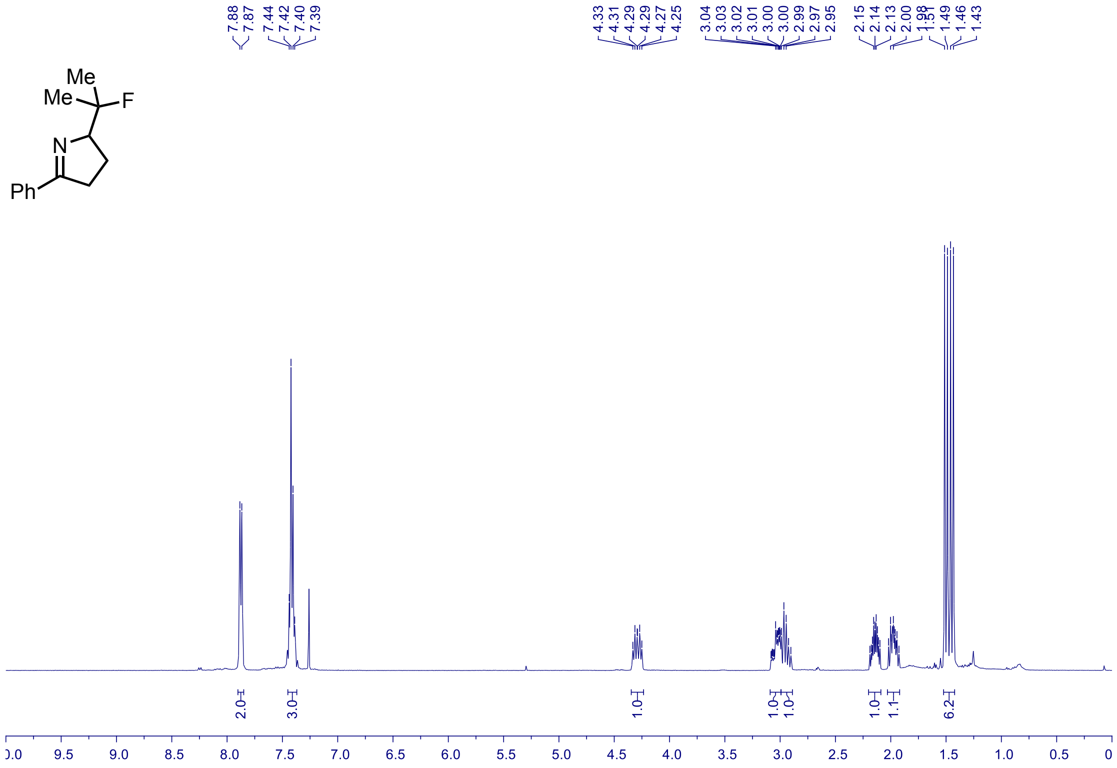


**5e** – $^{13}$C NMR (101 MHz, $CDCl_3$)

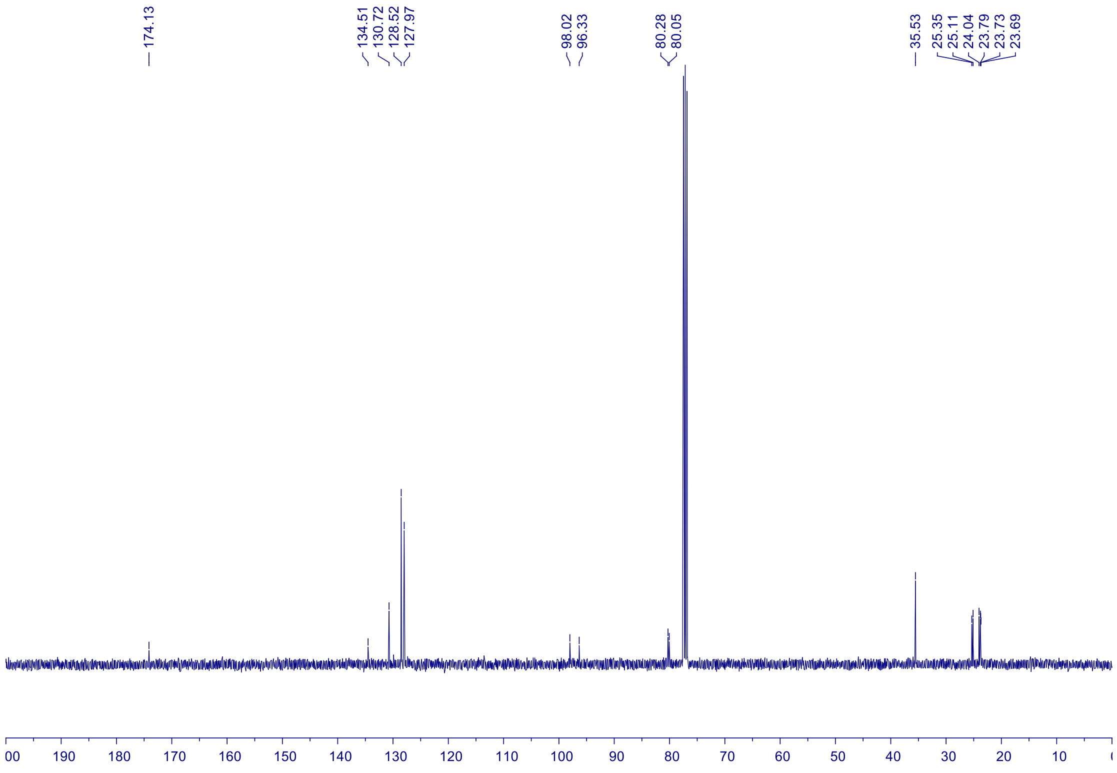

**5e** – $^{19}$F NMR (376 MHz, $CDCl_3$)

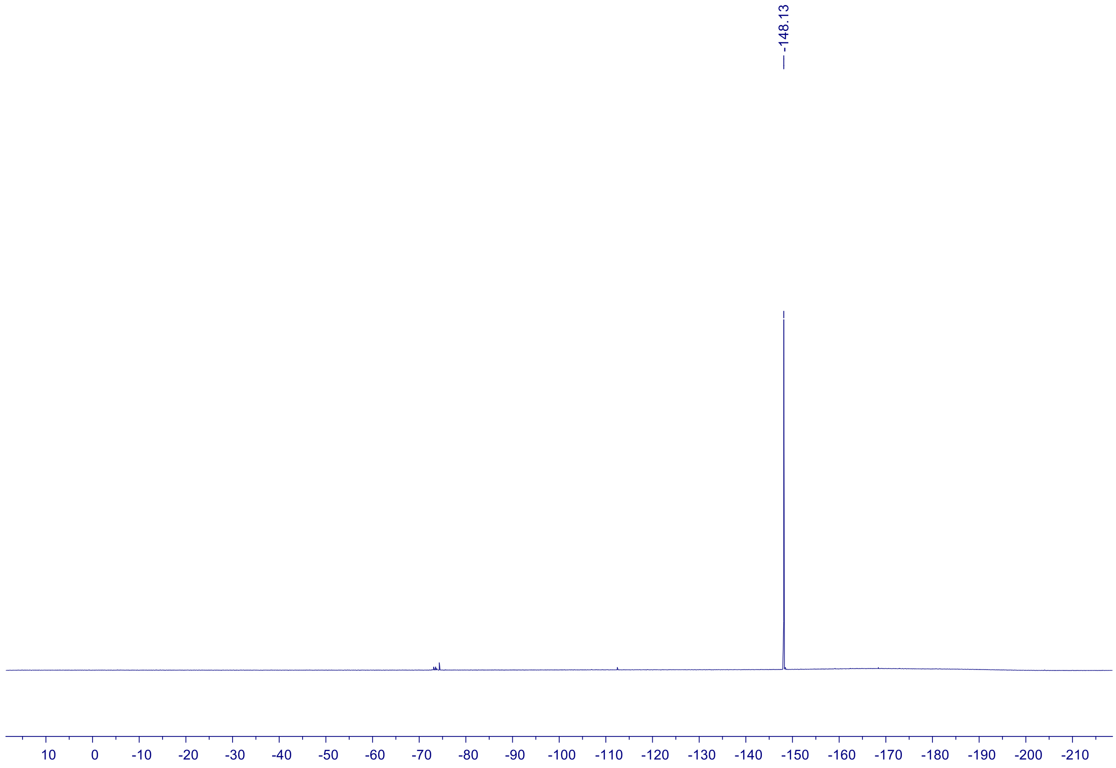

**5g** – $^{1}$H NMR (500 MHz, toluene-$d_9$, T = 100 ºC)

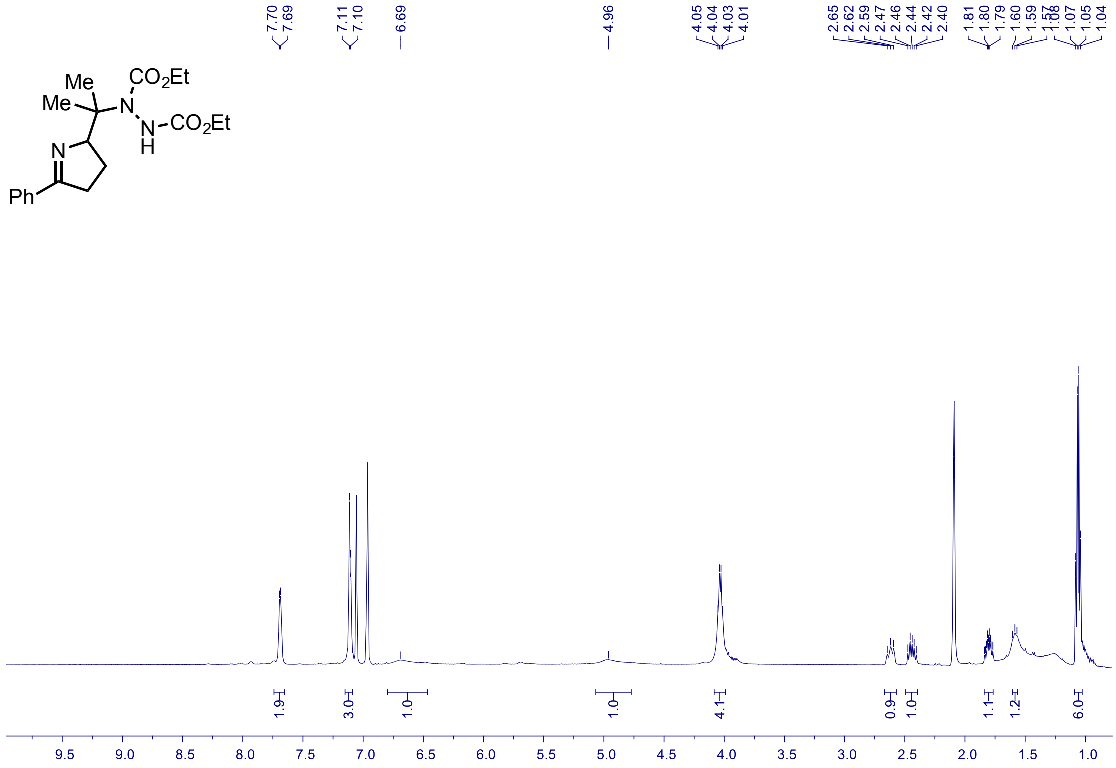


**5g** – $^{13}$C NMR (125 MHz, toluene-$d_9$, T = 100 ºC)

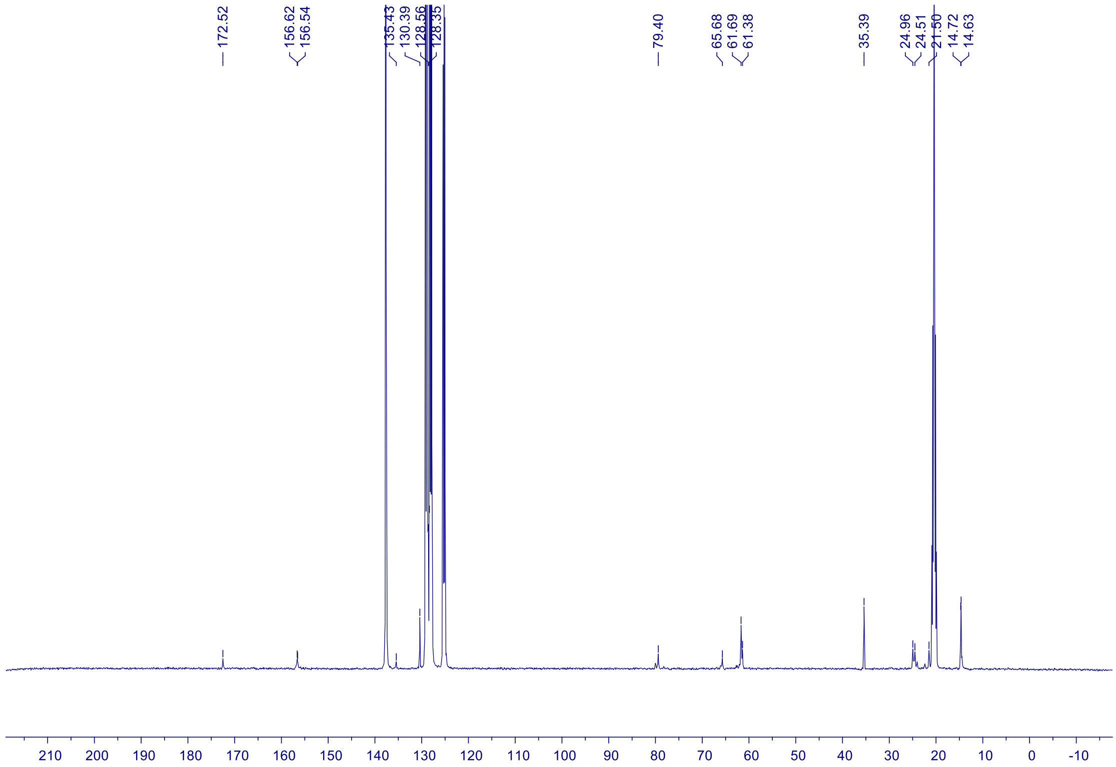

**5h** – $^{1}$H NMR (500 MHz, $CDCl_3$)

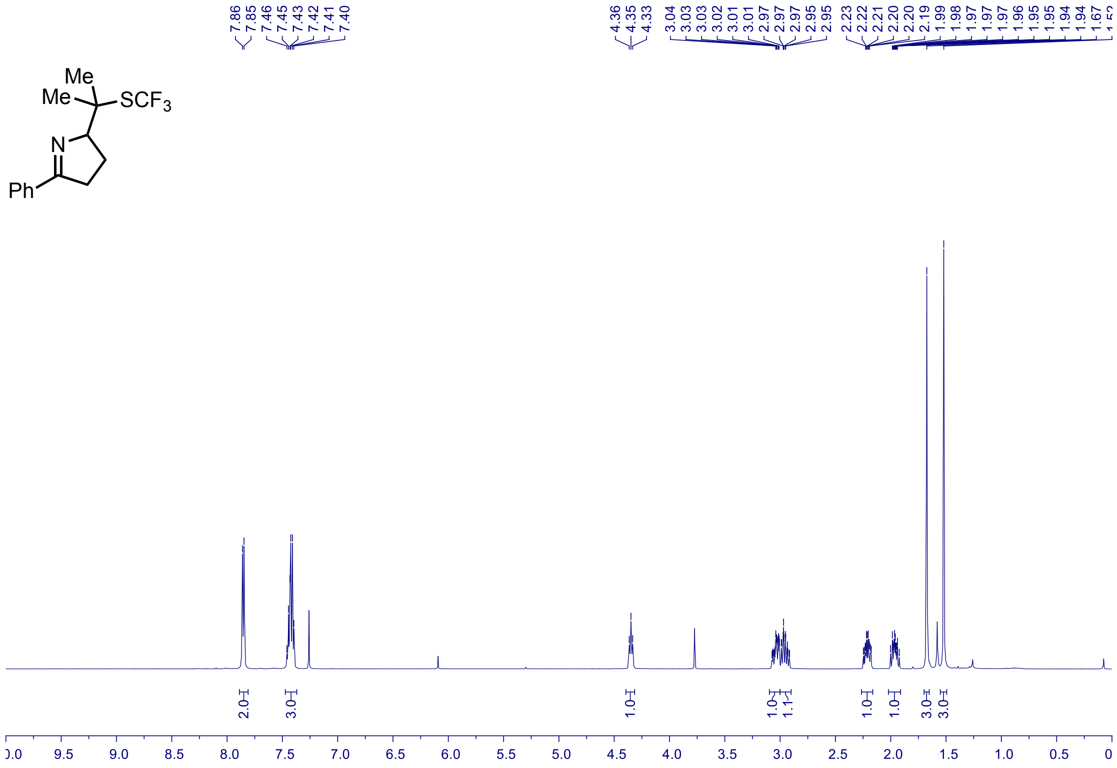


**5h** – $^{13}$C NMR (126 MHz, $CDCl_3$)

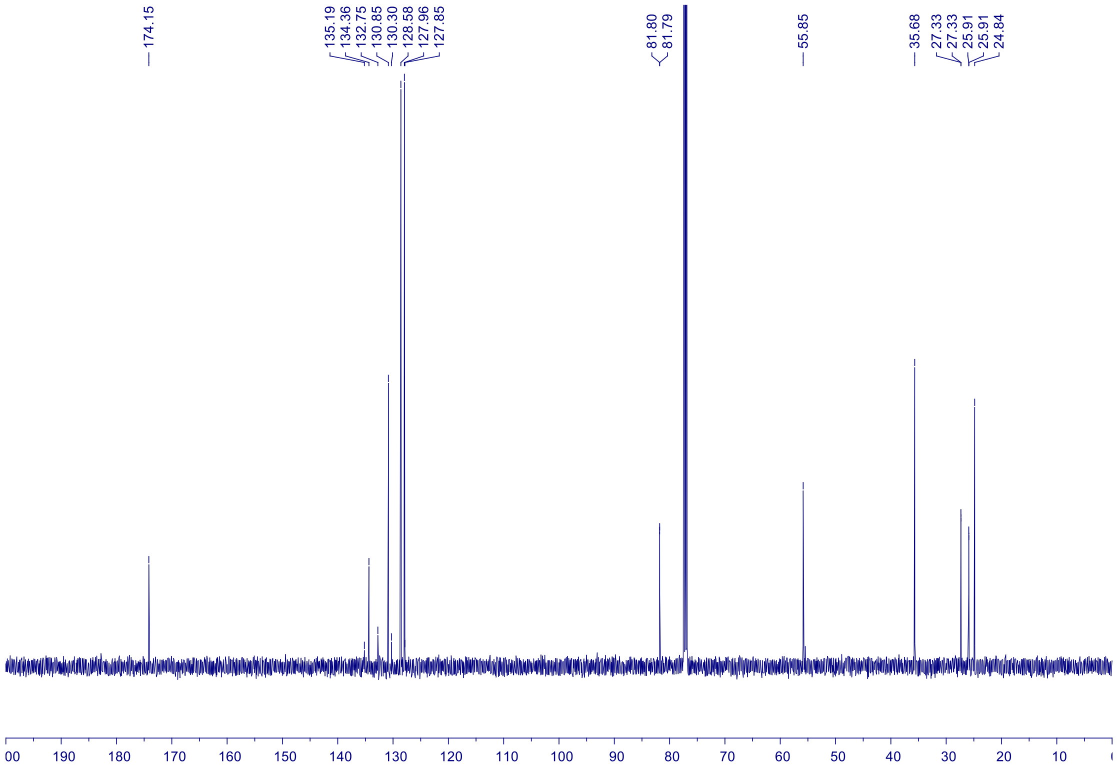

**5h** – $^{19}$F NMR (376 MHz, $CDCl_3$)

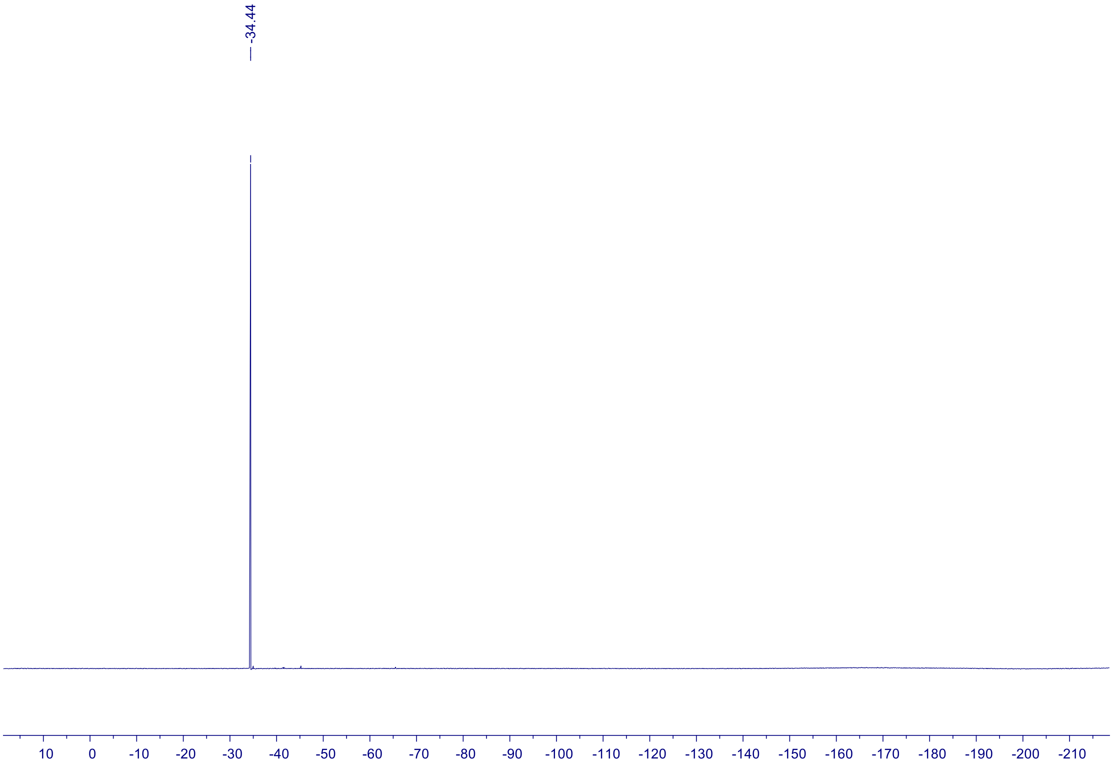

**5i** – $^{1}$H NMR (500 MHz, CDCl$_3$)

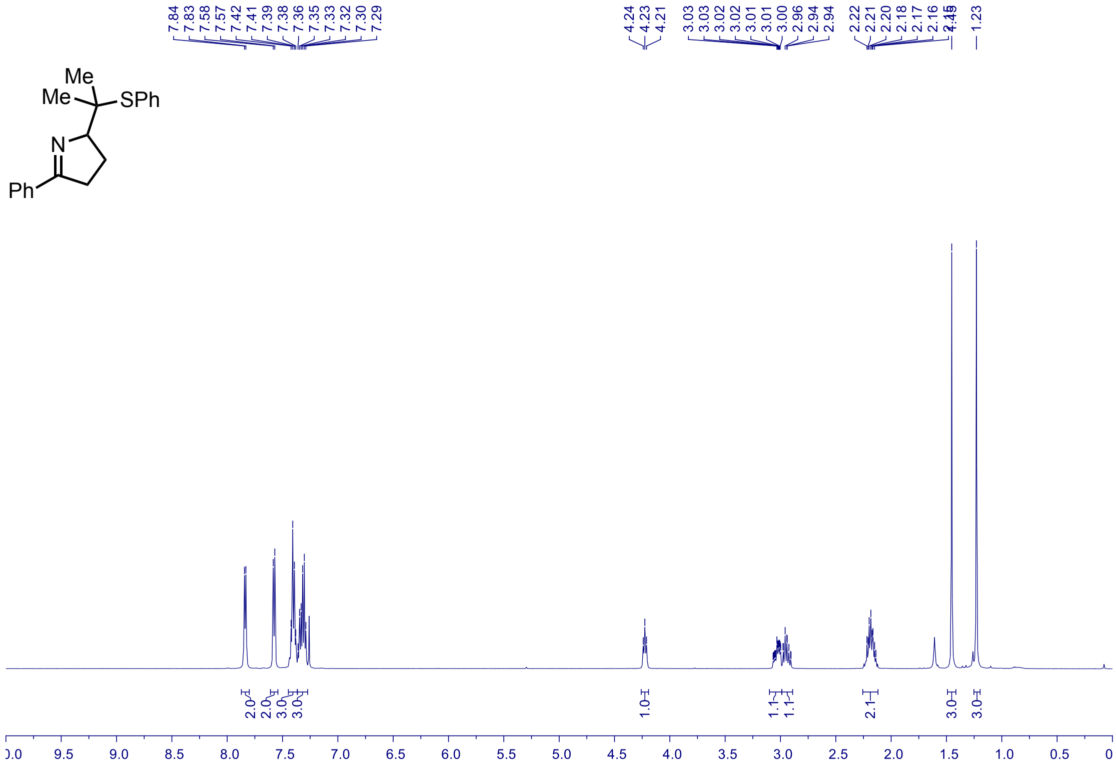


**5i** – $^{13}$C NMR (126 MHz, CDCl$_3$)

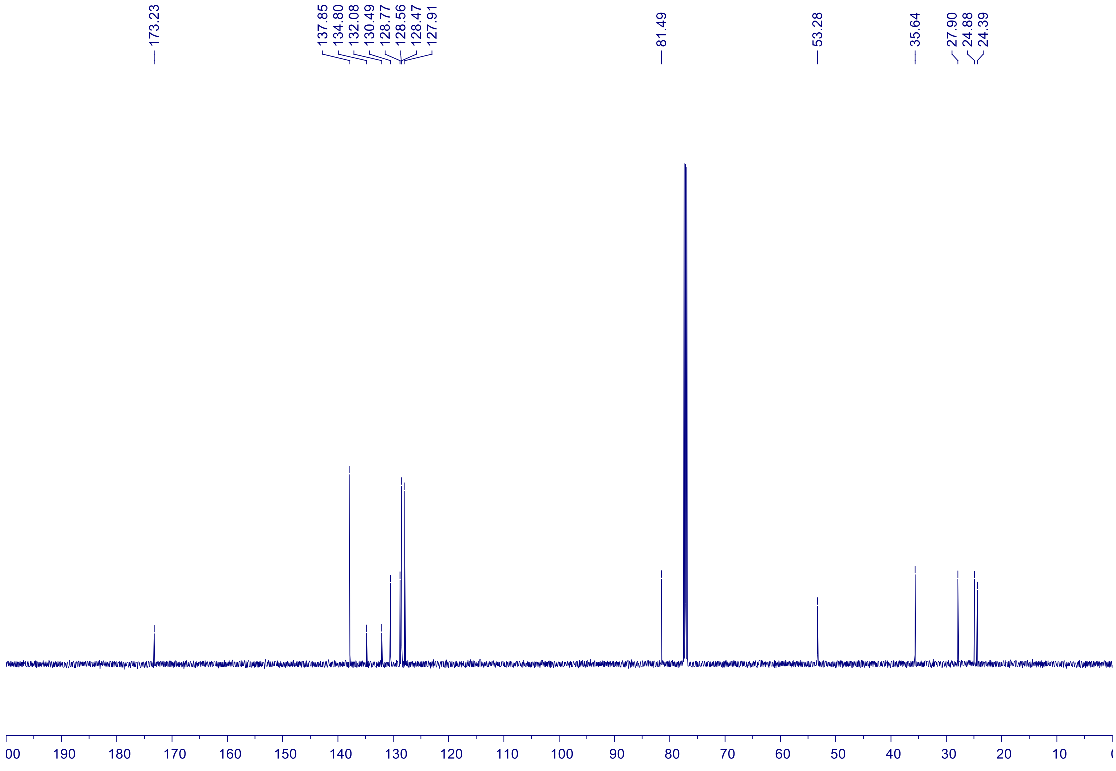

**5j** – $^{1}$H NMR (400 MHz, $CDCl_3$)

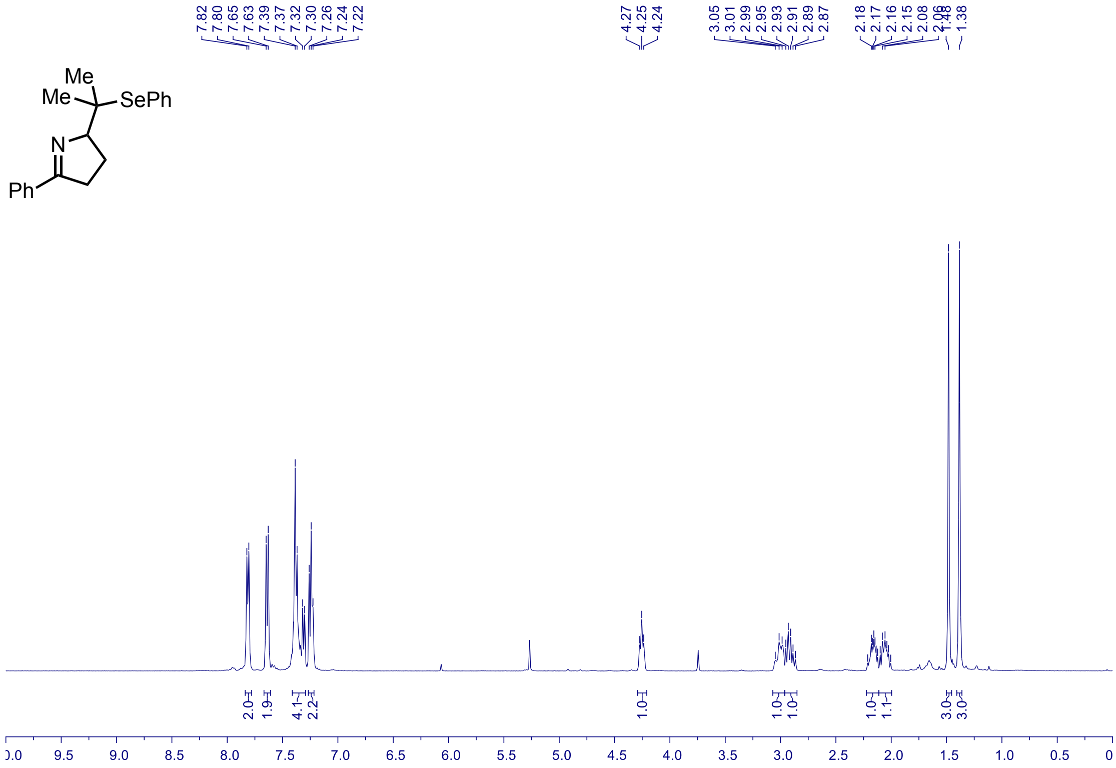


**5j** – $^{13}$C NMR (101 MHz, $CDCl_3$)

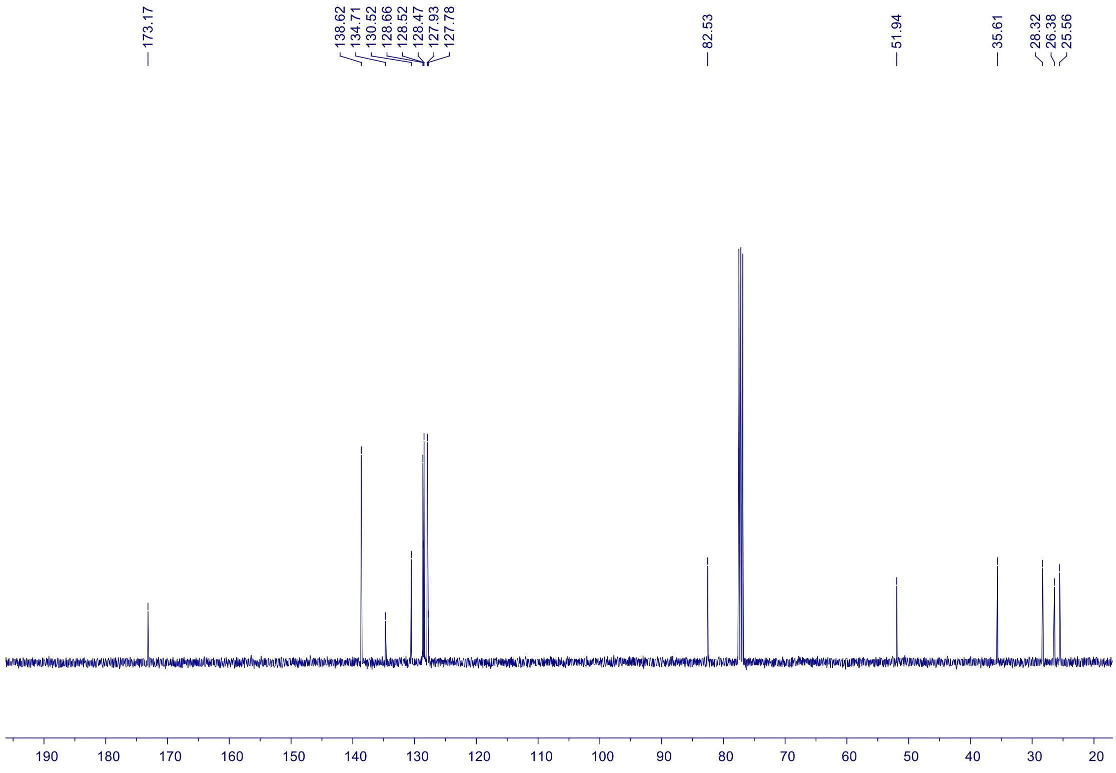

**5k** – $^{1}$H NMR (500 MHz, $CDCl_3$)

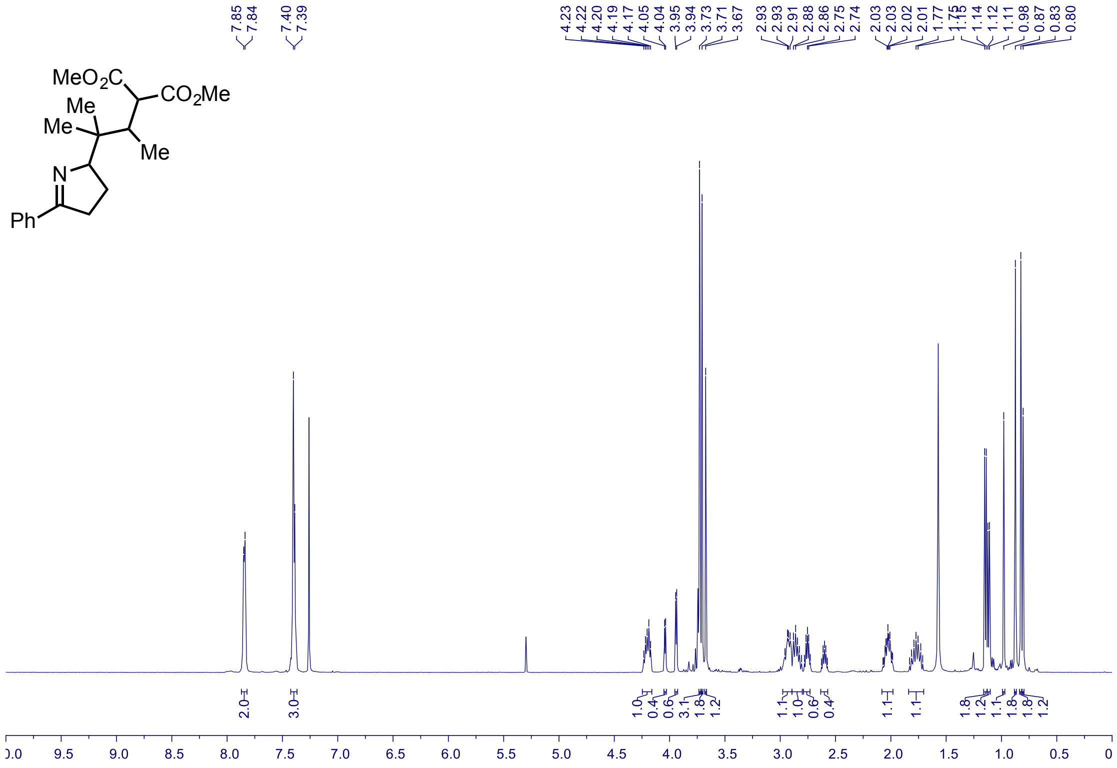


**5k** – $^{13}$C NMR (126 MHz, $CDCl_3$)

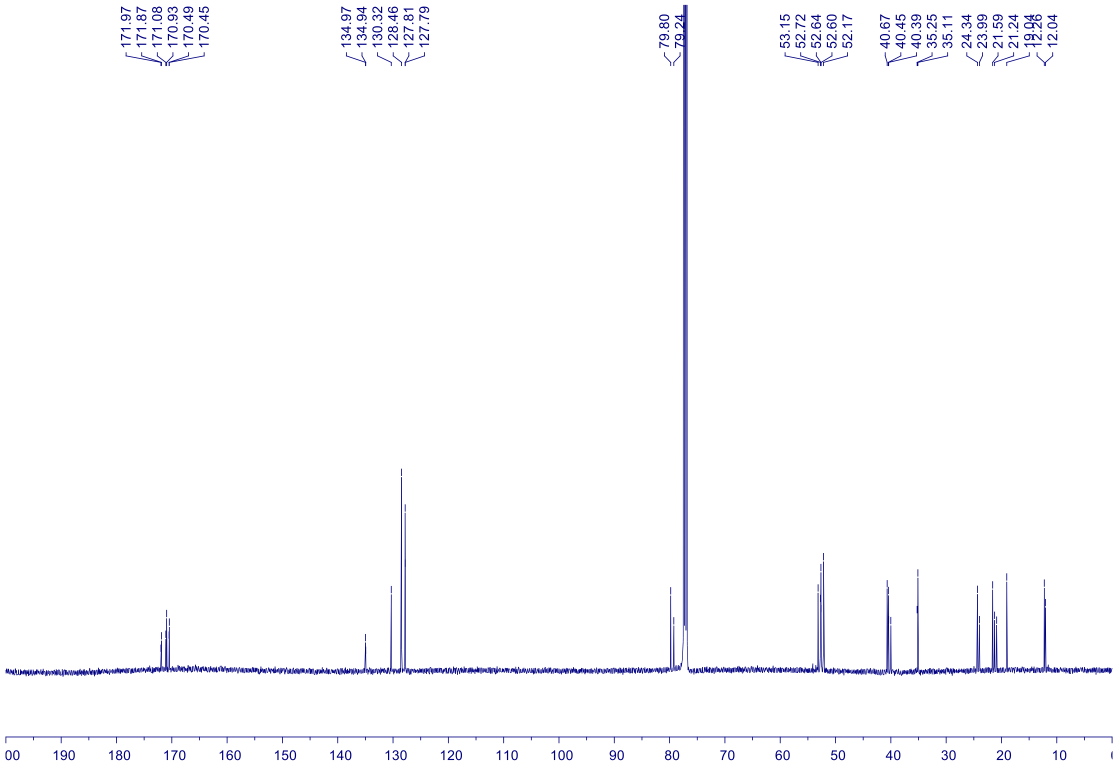

**5l** – $^{1}$H NMR (400 MHz, $CDCl_3$)

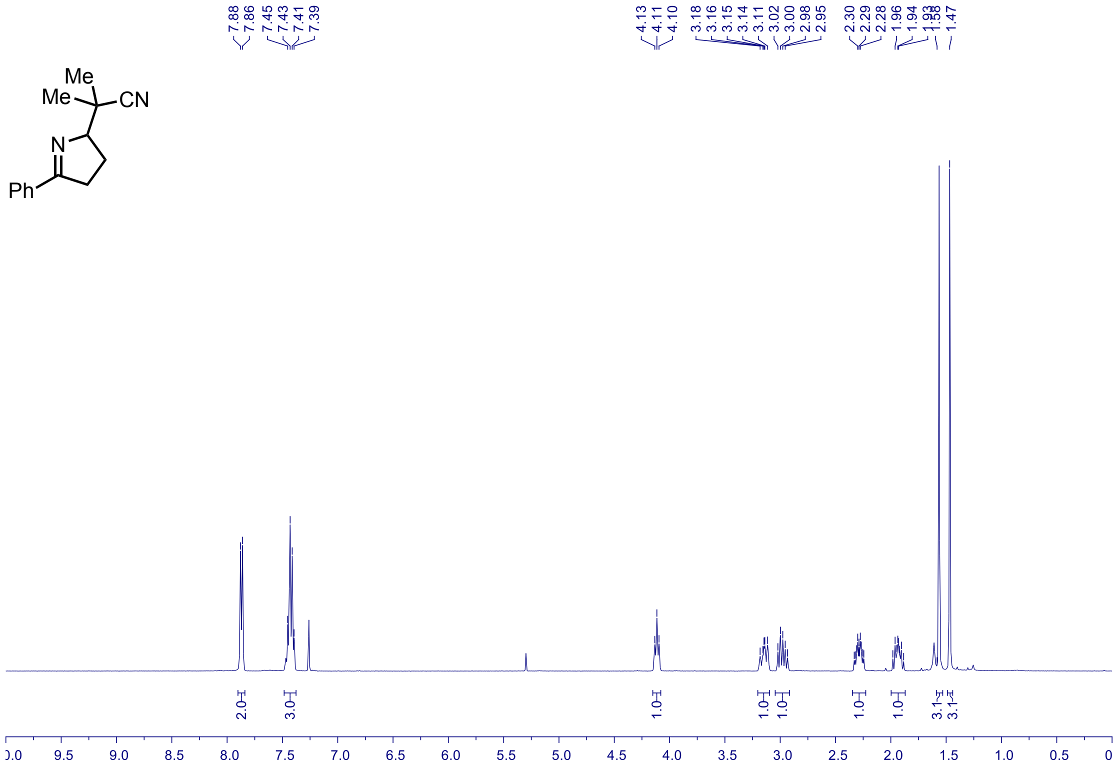


**5l** – $^{13}$C NMR (101 MHz, $CDCl_3$)

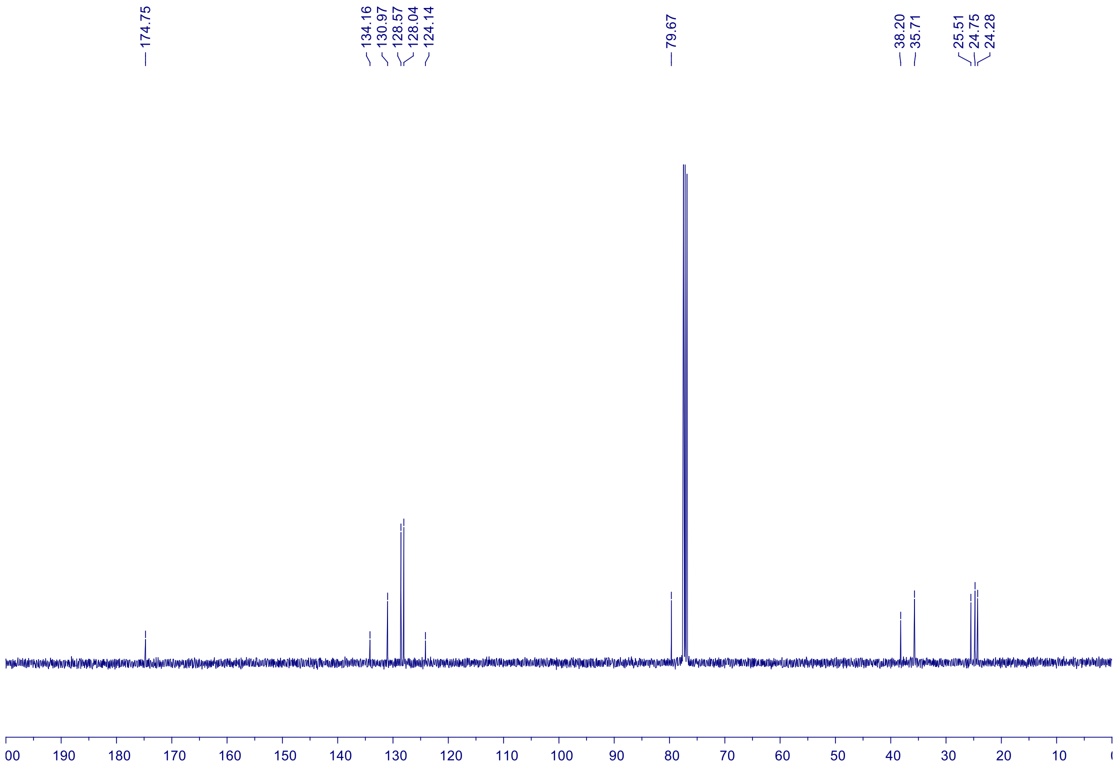

**5m** – $^{1}$H NMR (400 MHz, CDCl$_3$)

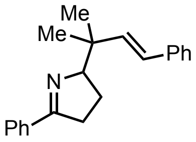


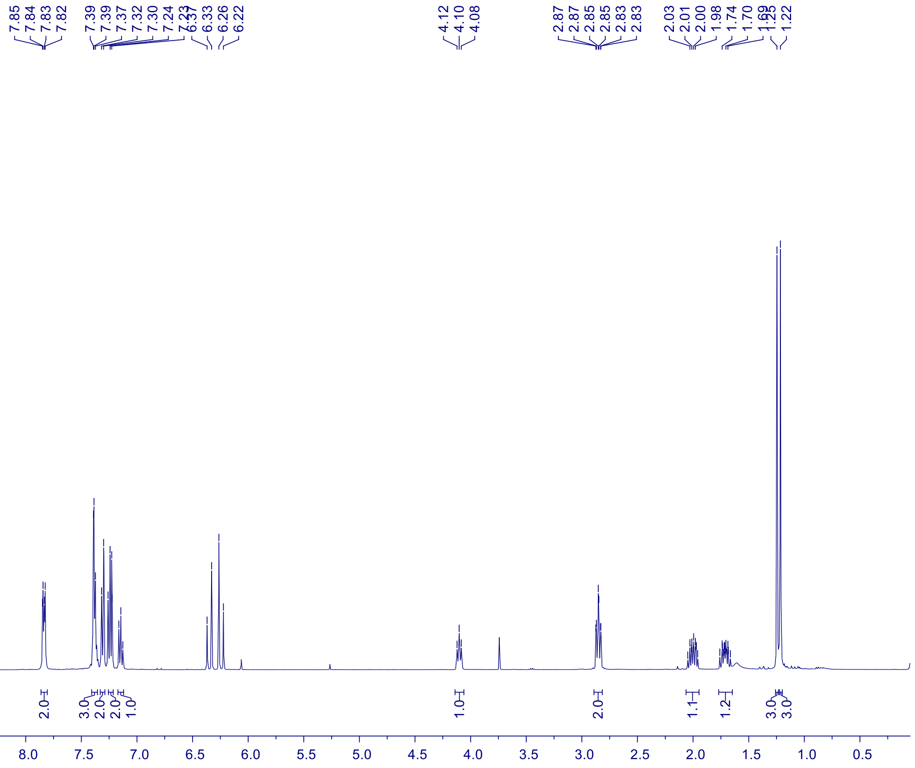


**5m** – $^{13}$C NMR (101 MHz, CDCl$_3$)

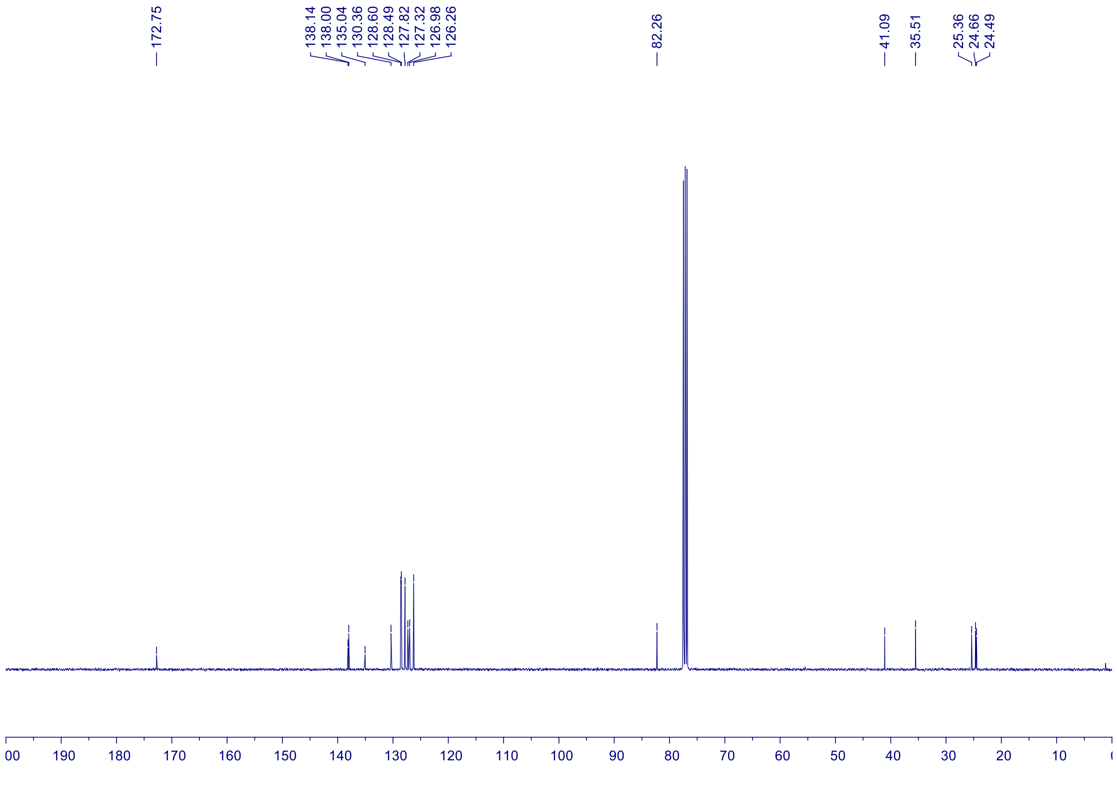

**5n** – $^{1}$H NMR (500 MHz, CDCl$_3$)

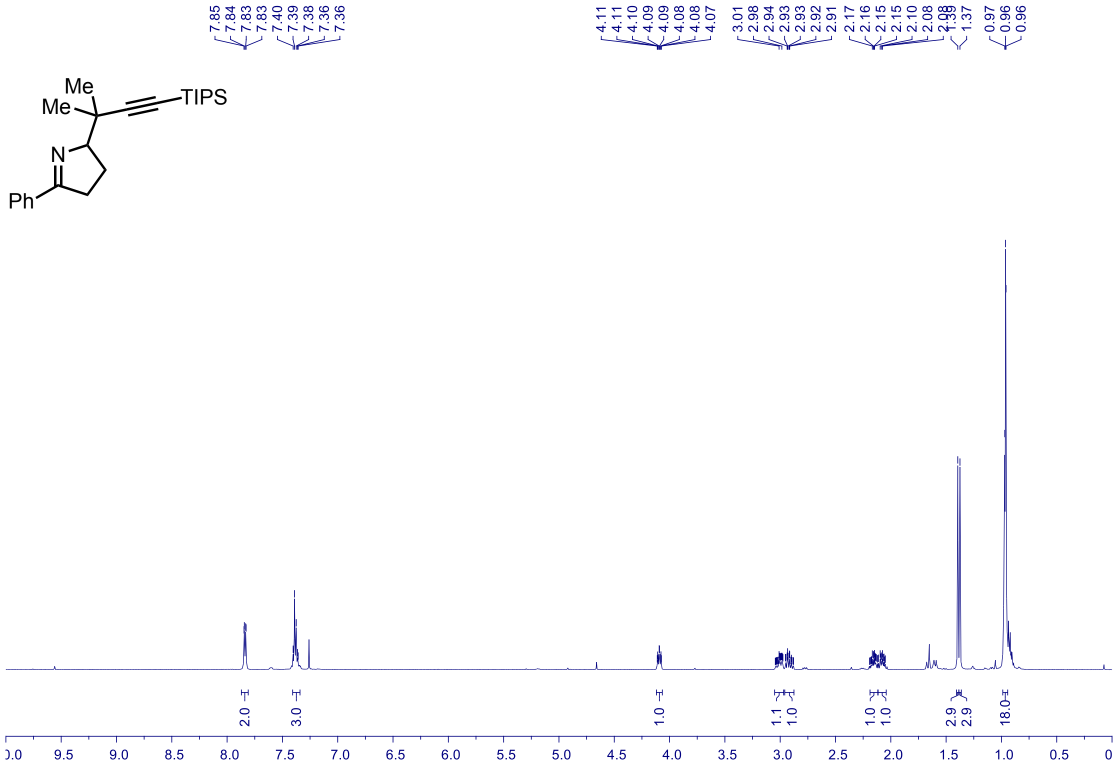


**5n** – $^{13}$C NMR (126 MHz, CDCl$_3$)

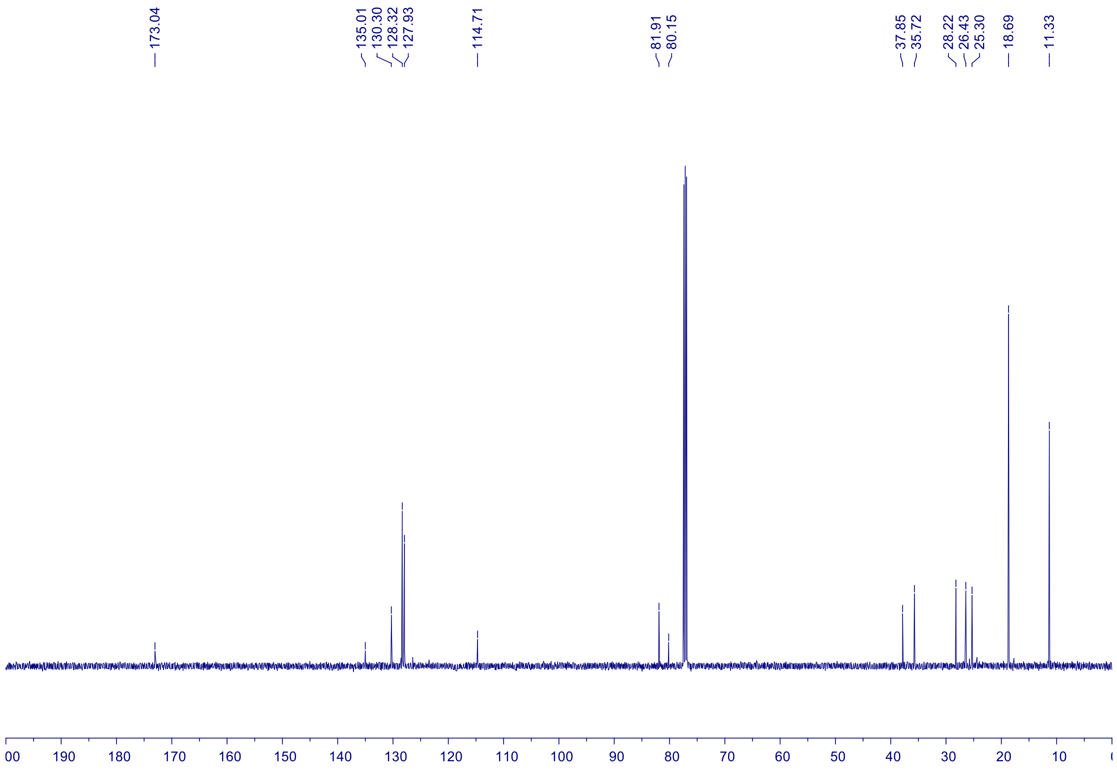

**6d** – $^{1}$H NMR (500 MHz, toluene-$d_9$, T = 100 ºC)

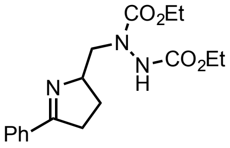

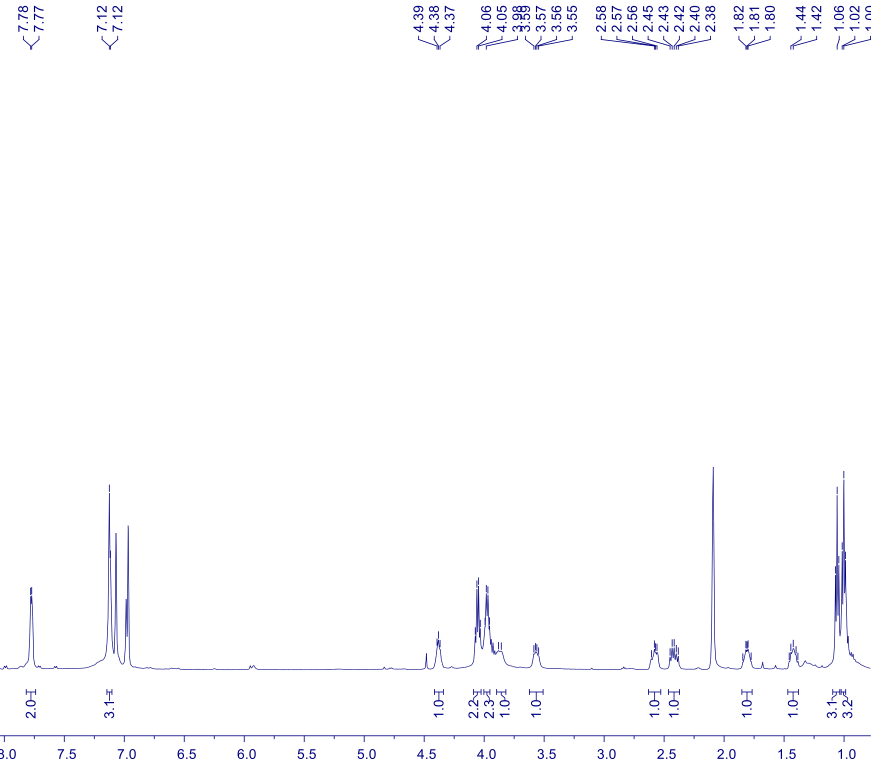

**6d** – $^{13}$C NMR (125 MHz, toluene-$d_9$, T = 100 ºC)

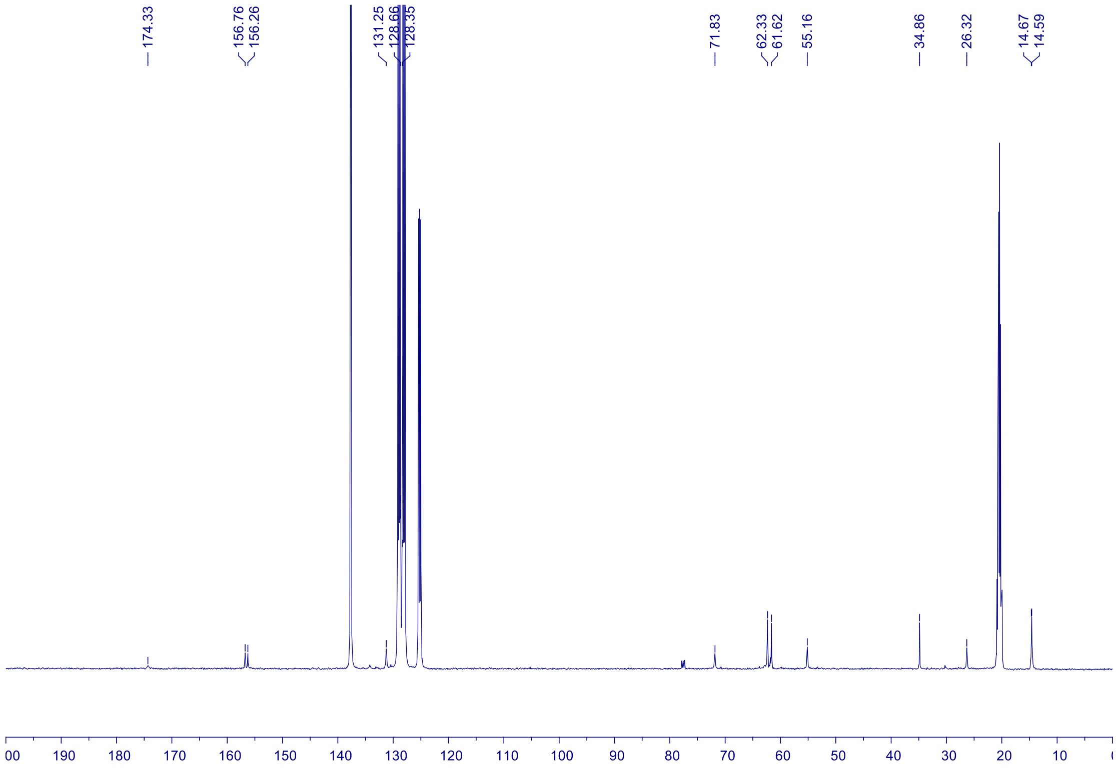

**6g** – $^{1}$H NMR (500 MHz, $CDCl_3$)

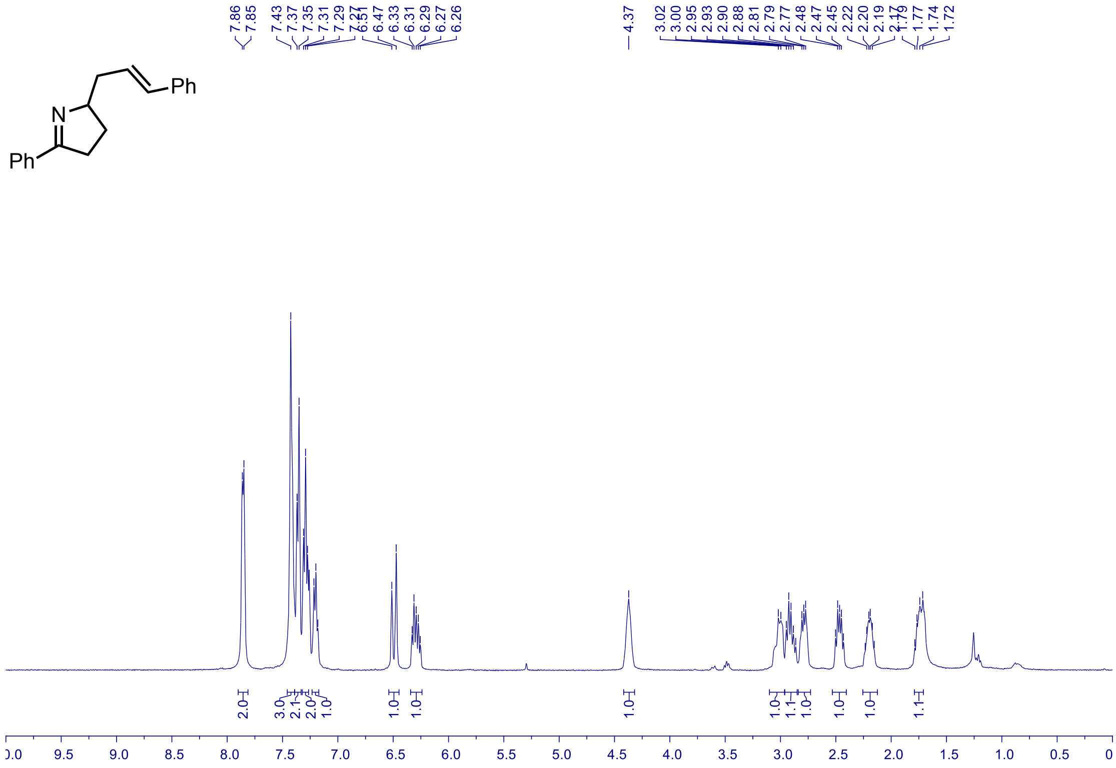

**6g** – $^{13}$C NMR (126 MHz, $CDCl_3$)

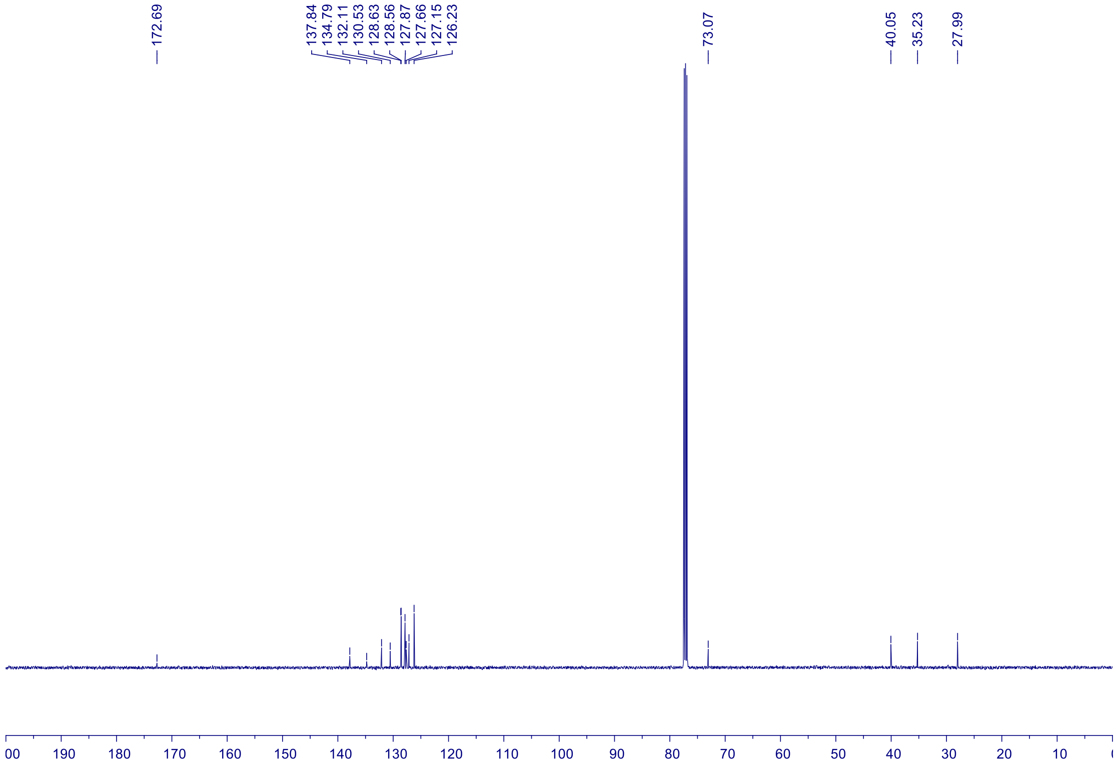

**6h** – $^{1}$H NMR (500 MHz, $CDCl_3$)

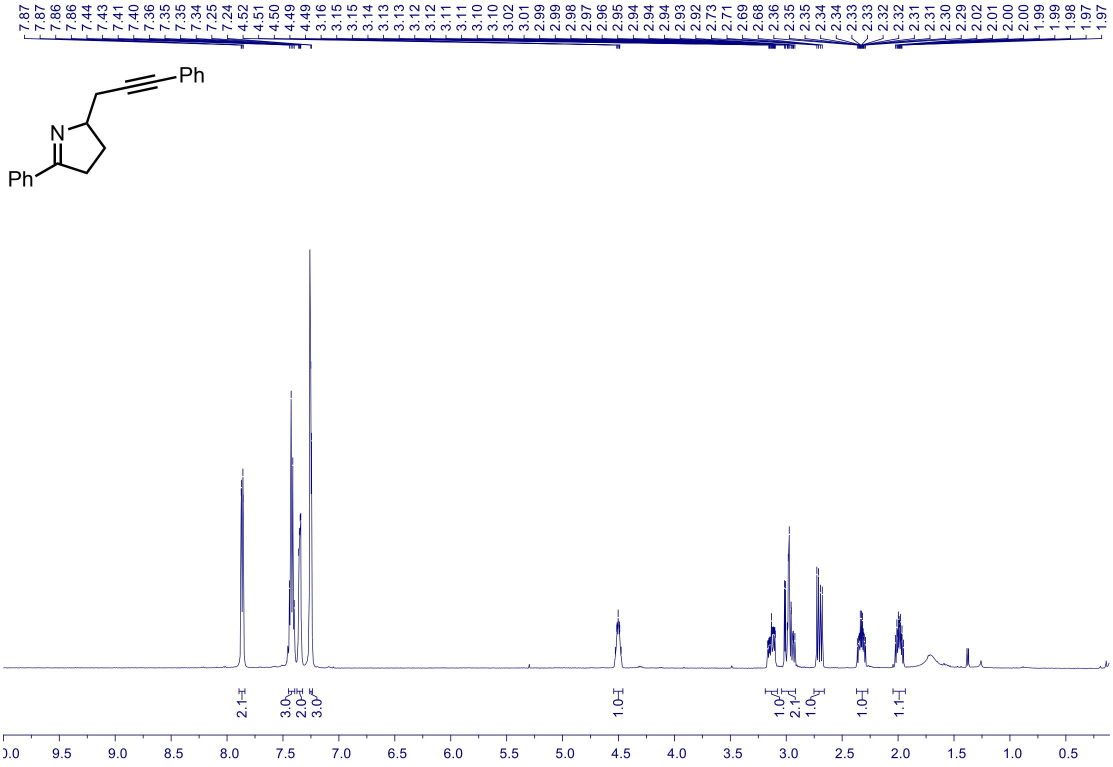


**6h** – $^{13}$C NMR (126 MHz, $CDCl_3$)

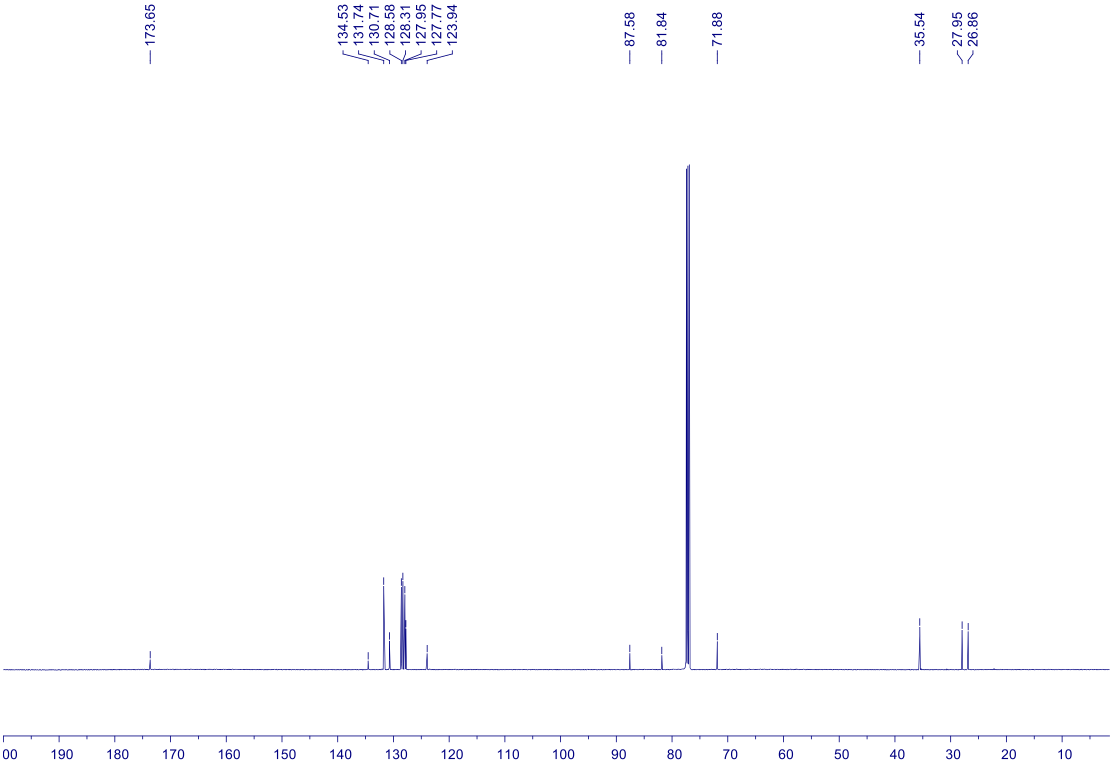

**6i** – $^{1}$H NMR (500 MHz, CDCl$_3$)

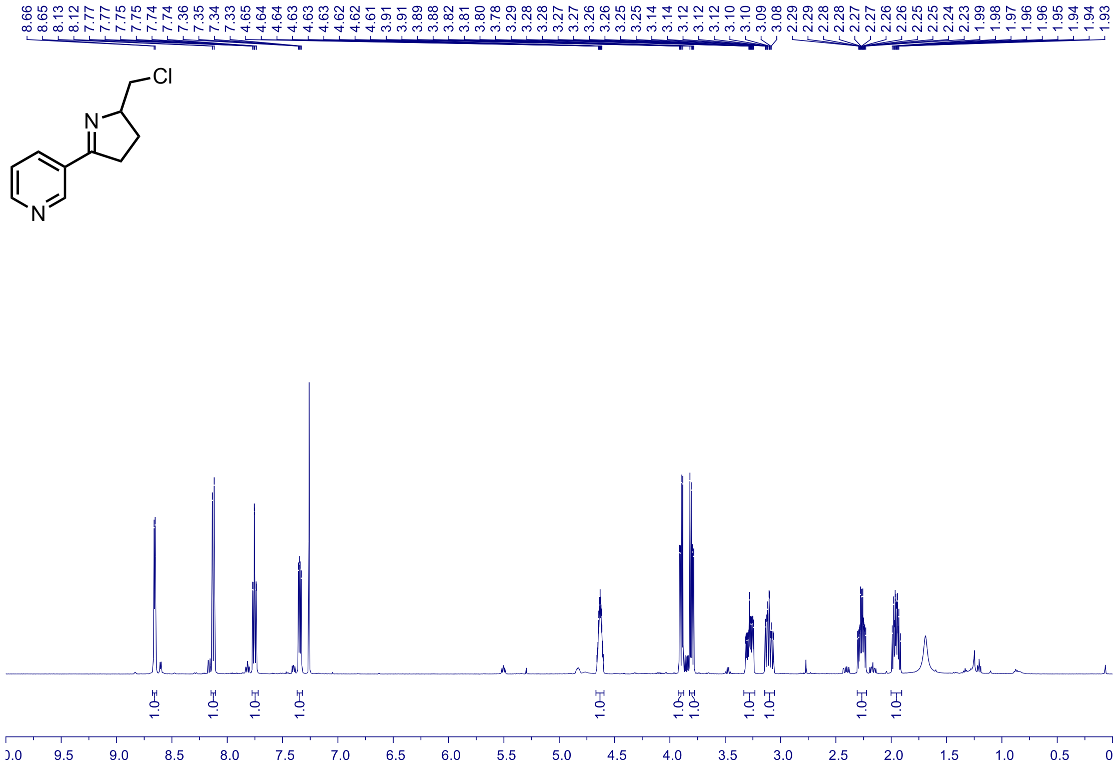


**6i** – $^{13}$C NMR (126 MHz, CDCl$_3$)

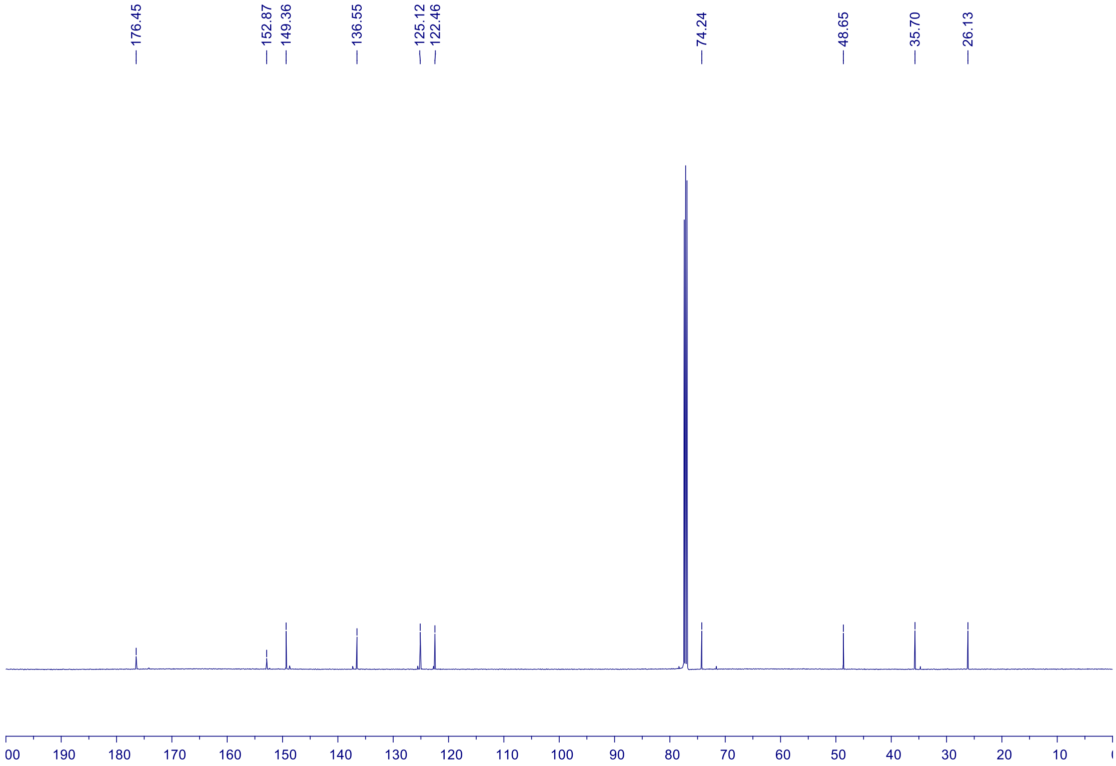

**6j** – $^{1}$H NMR (500 MHz, toluene-$d_9$, T = 100 ºC)

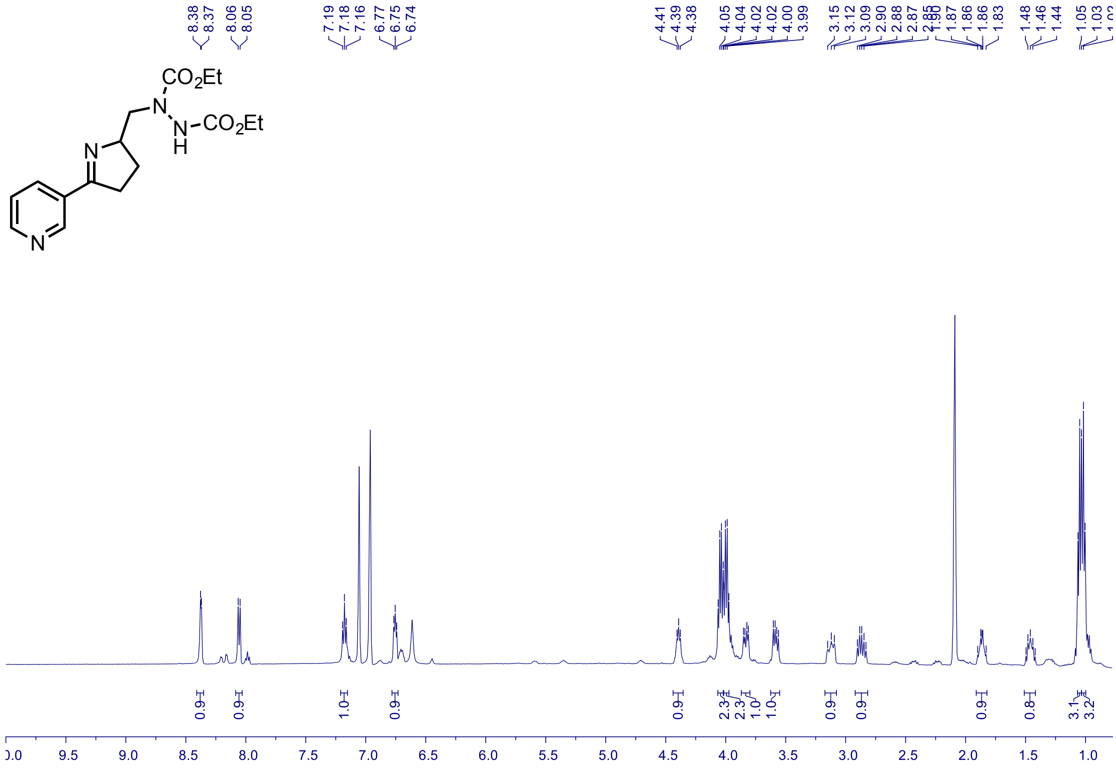


**6j** – $^{13}$C NMR (125 MHz, toluene-$d_9$, T = 100 ºC)

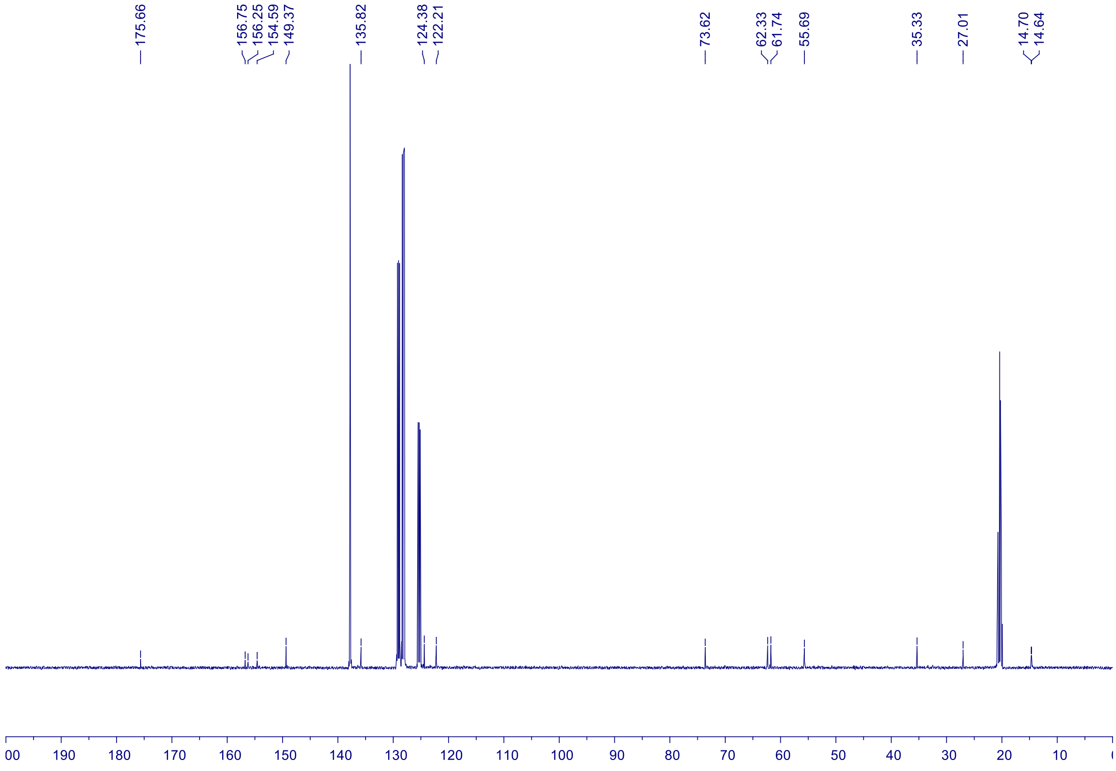

**6k** – $^{1}$H NMR (400 MHz, $CDCl_3$)

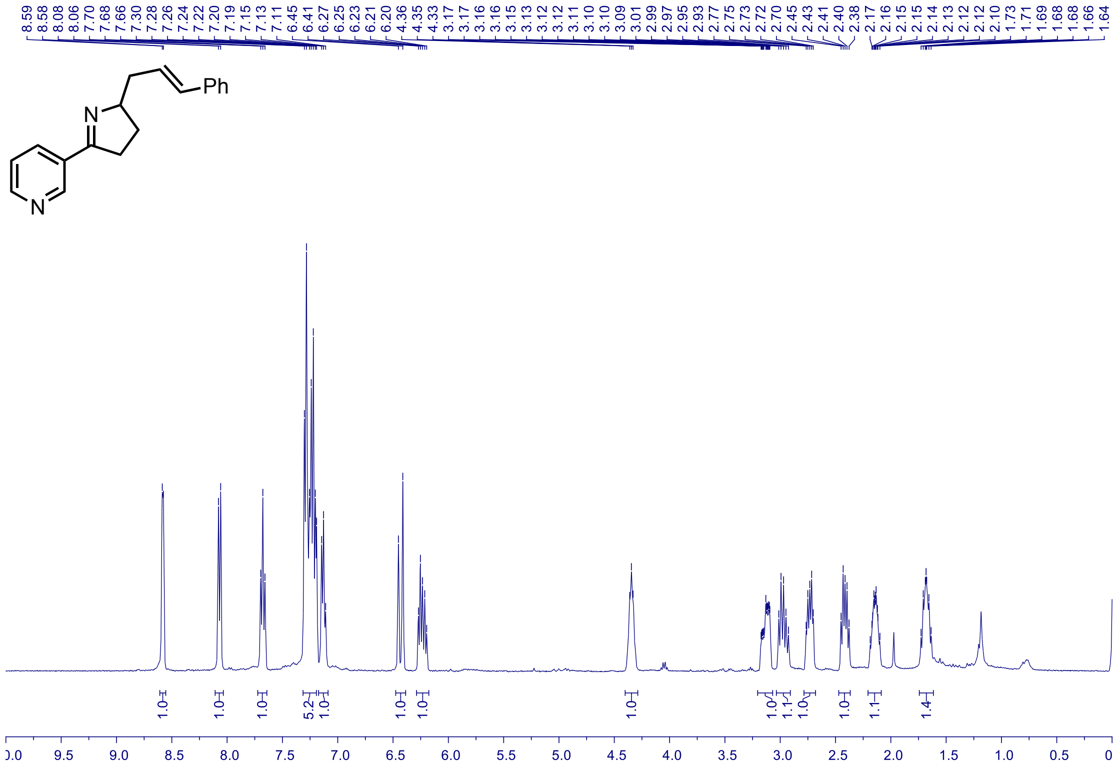


**6k** – $^{13}$C NMR (101 MHz, $CDCl_3$)

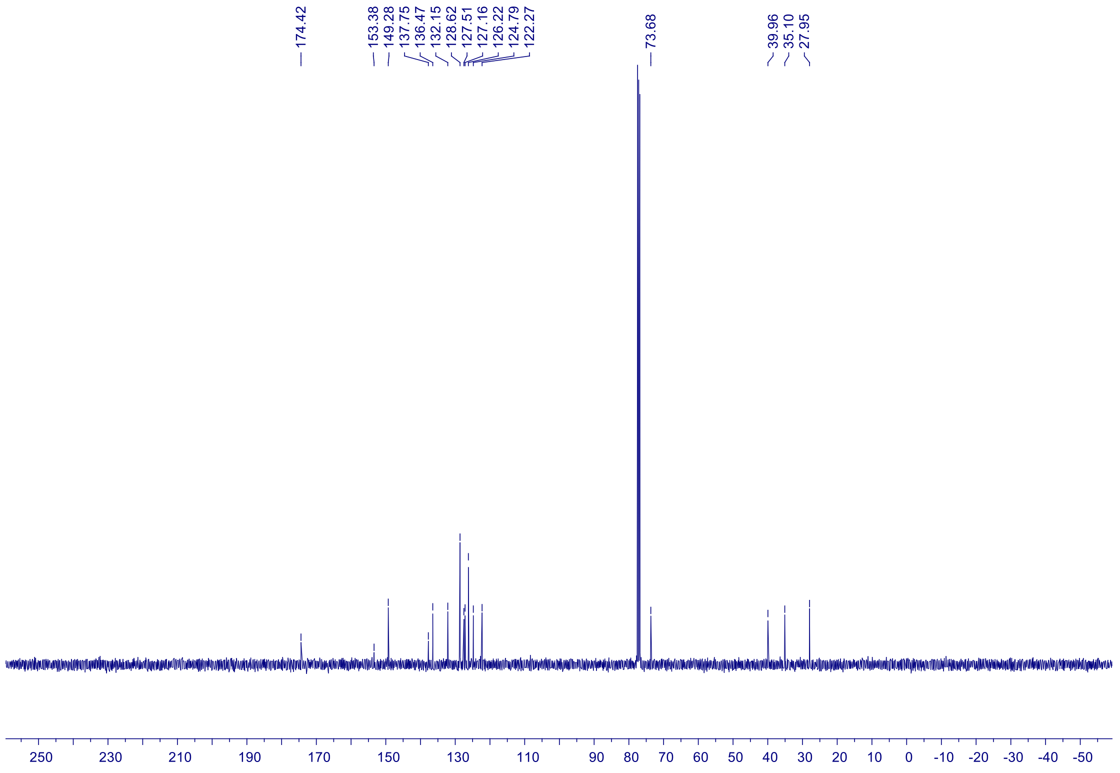

**7b** – $^{1}$H NMR (400 MHz, CDCl$_3$)

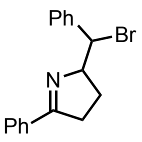

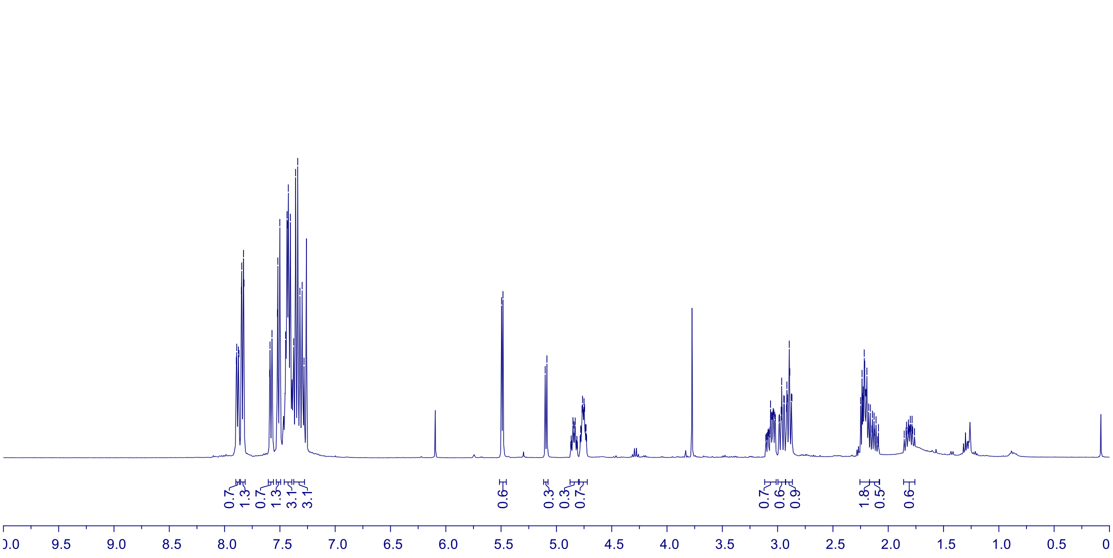

**7b** – $^{13}$C NMR (101 MHz, CDCl$_3$)

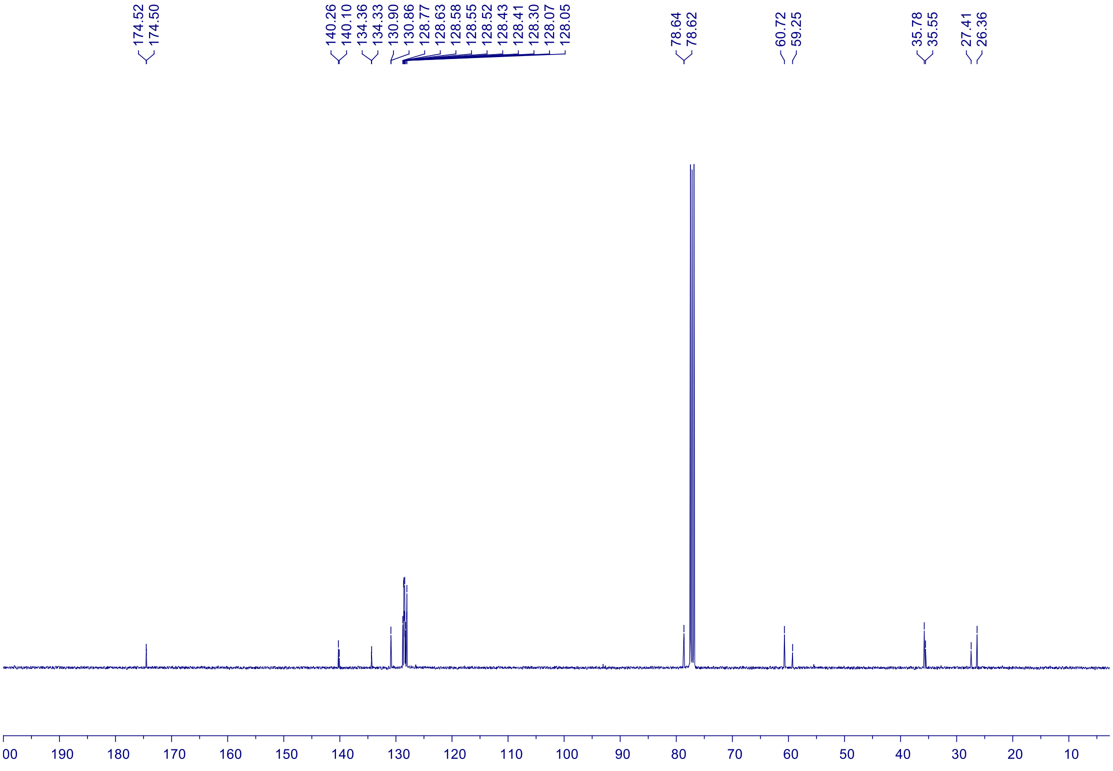

**7c** – $^{1}$H NMR (400 MHz, $CDCl_3$)

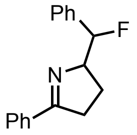


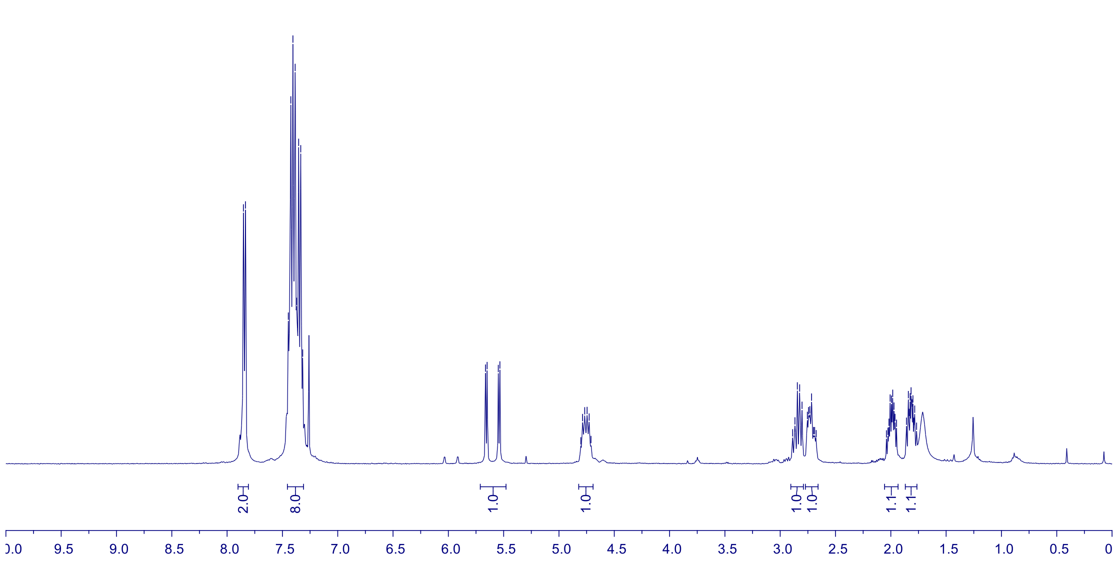


**7c** – $^{13}$C NMR (101 MHz, $CDCl_3$)

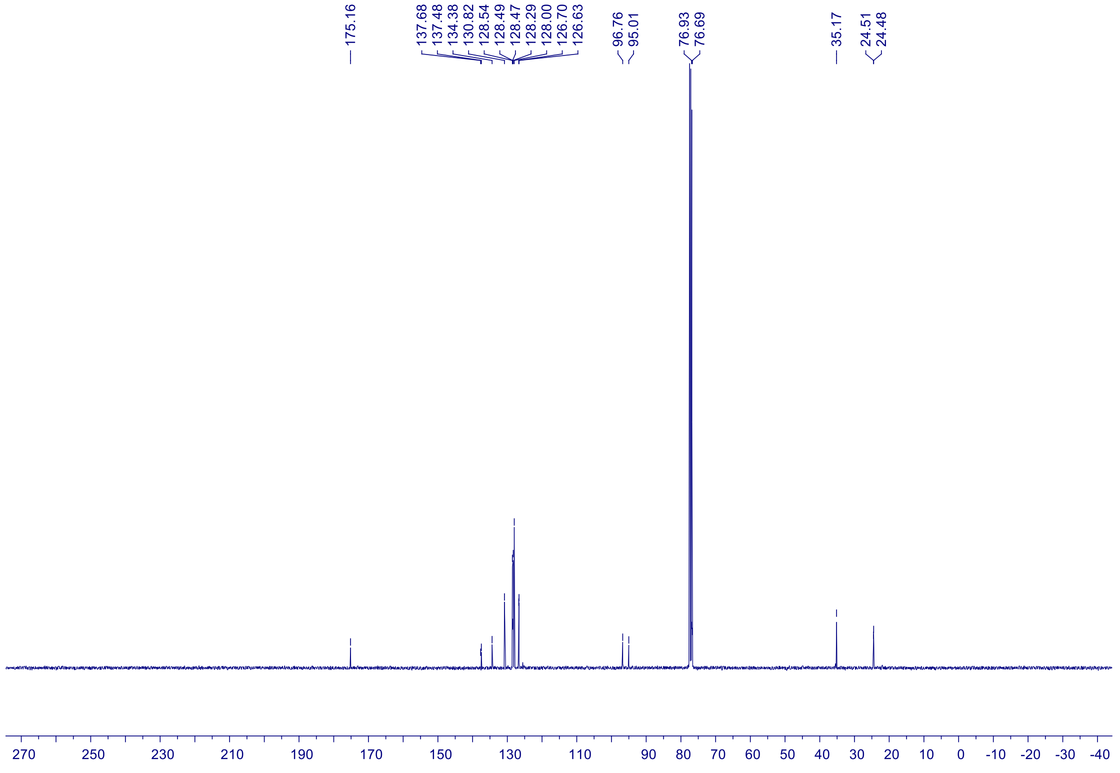

**7c** – $^{19}$F NMR (376 MHz, $CDCl_3$)

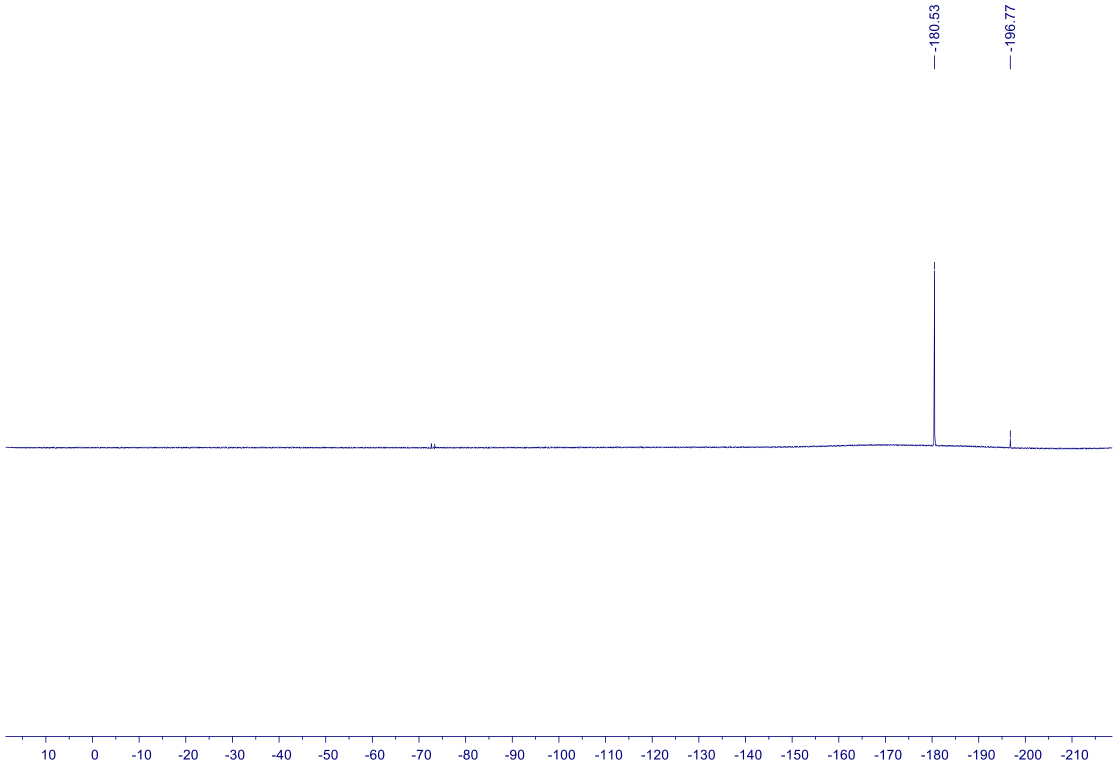

**7d** – $^{1}$H NMR (400 MHz, $C_6D_6$, T = 75 ºC )

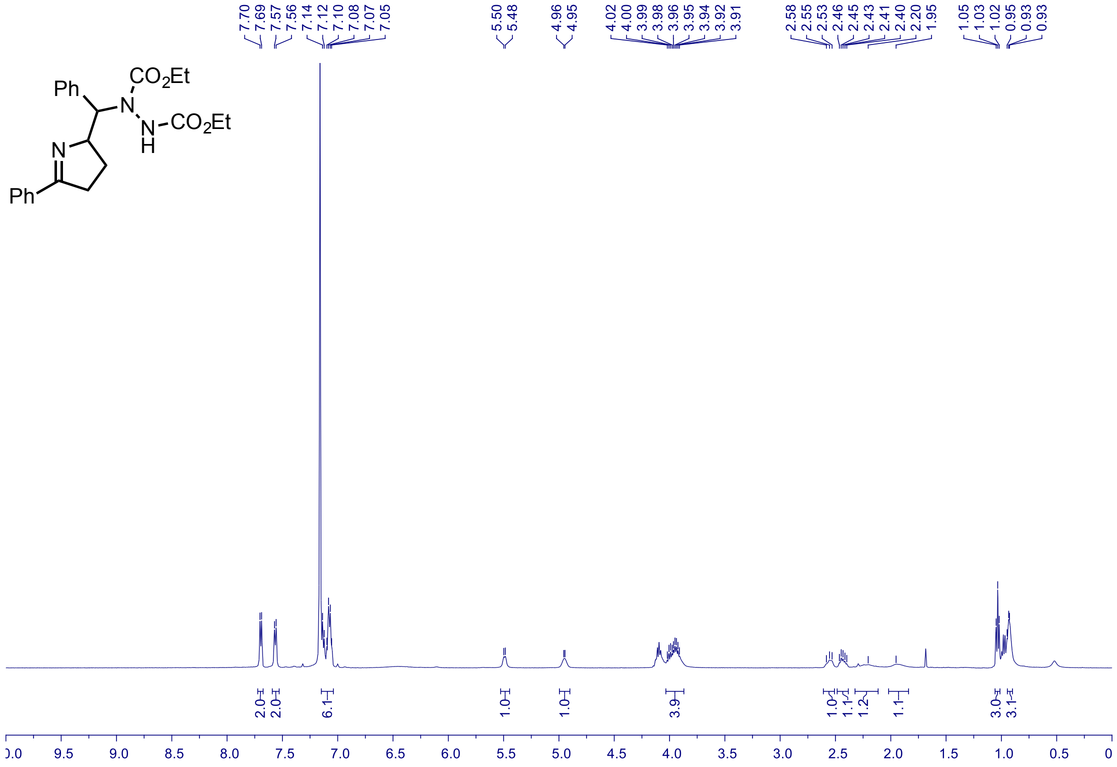


**7d** – $^{13}$C NMR (101 MHz, $C_6D_6$, T = 75 ºC)

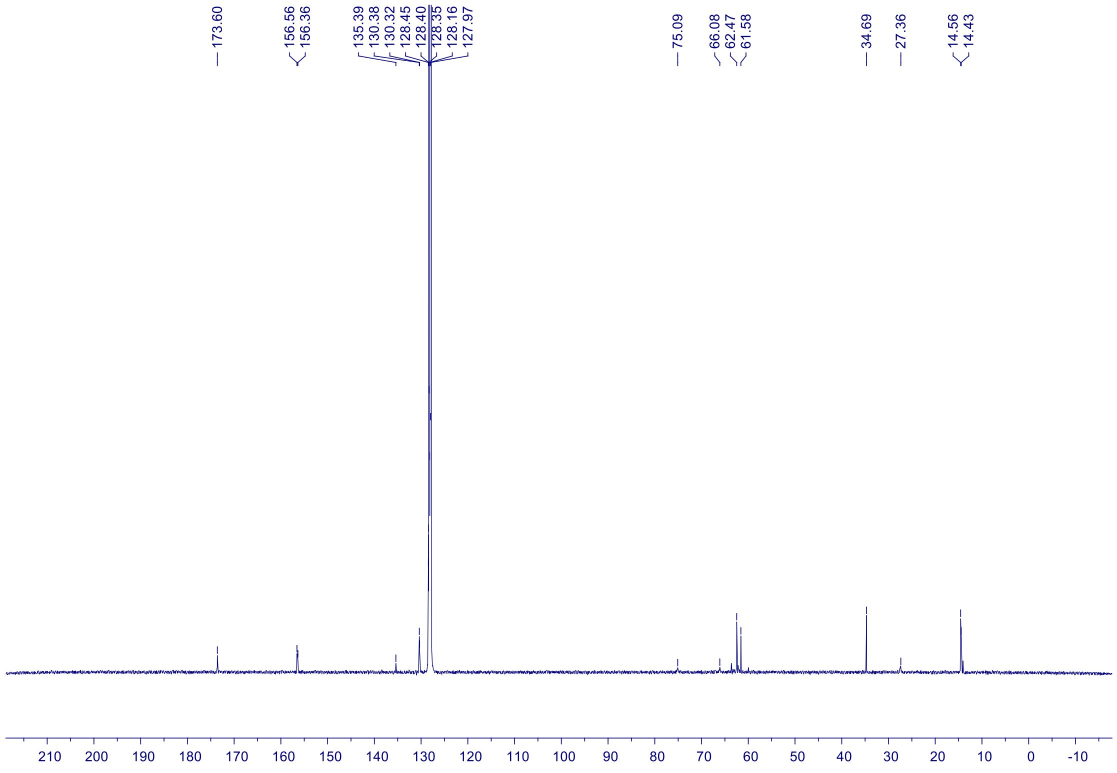

**7e** – $^{1}$H NMR (500 MHz, $CDCl_3$)

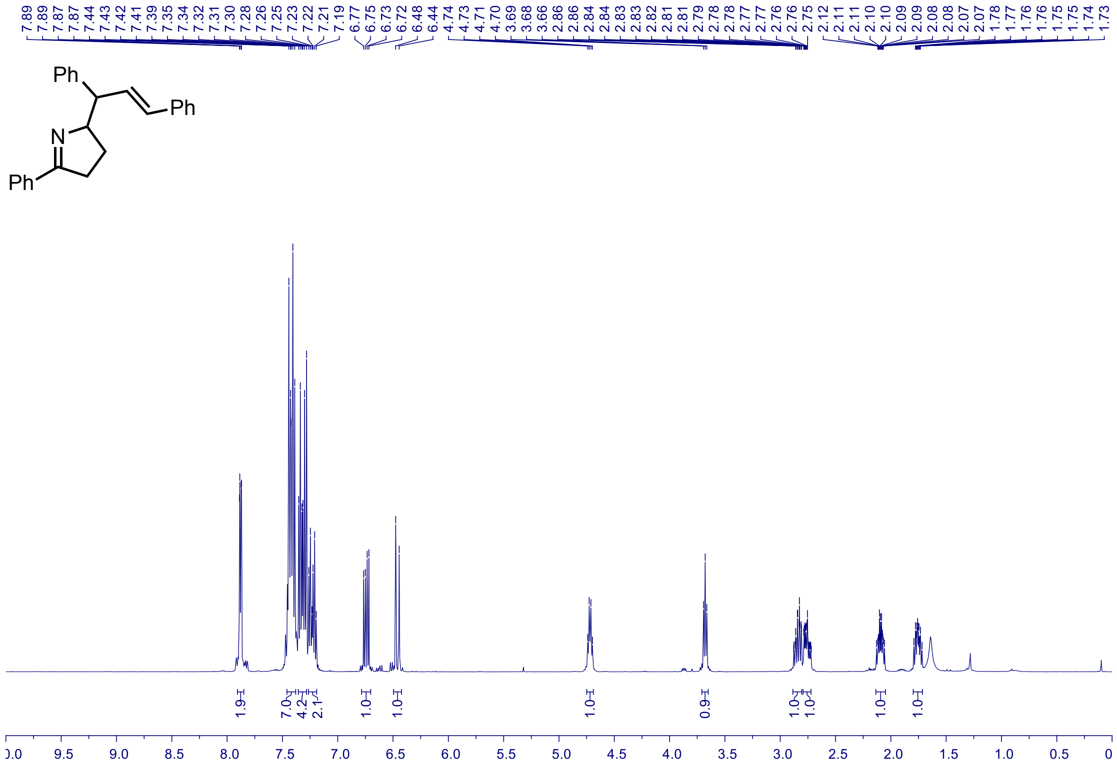

**7e** – $^{13}$C NMR (126 MHz, $CDCl_3$)

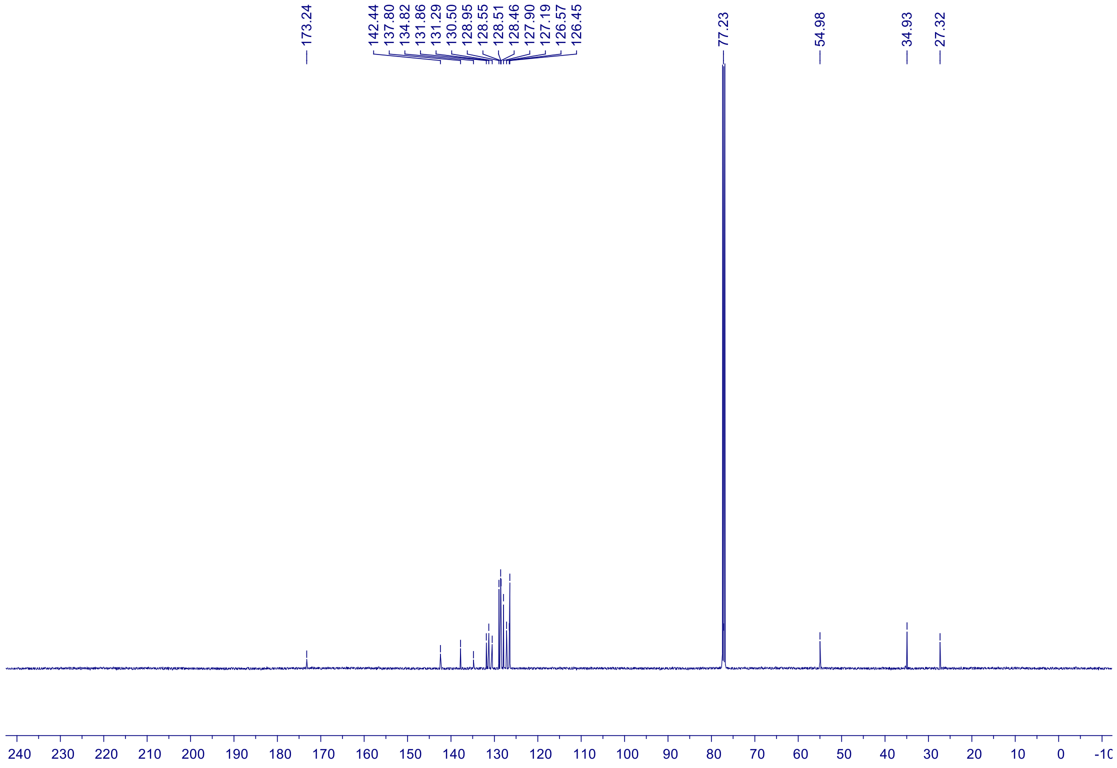

**7f** – $^{1}$H NMR (500 MHz, $CDCl_3$)

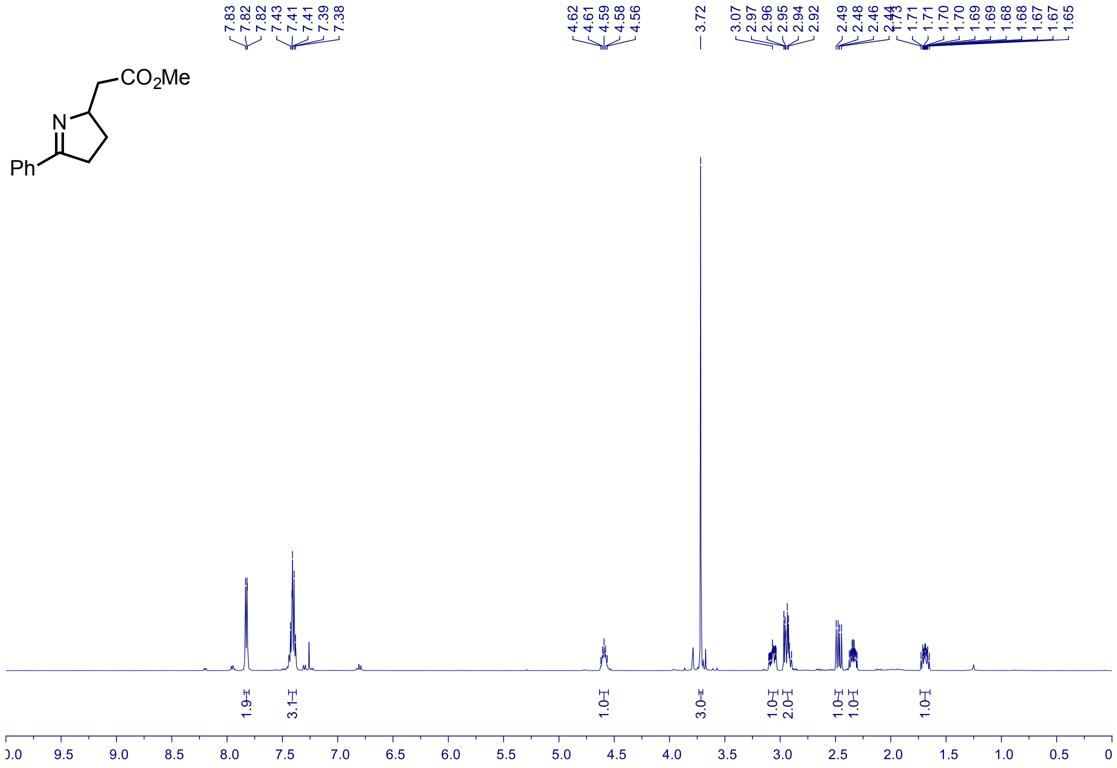

**7f** – $^{13}$C NMR (126 MHz, $CDCl_3$)

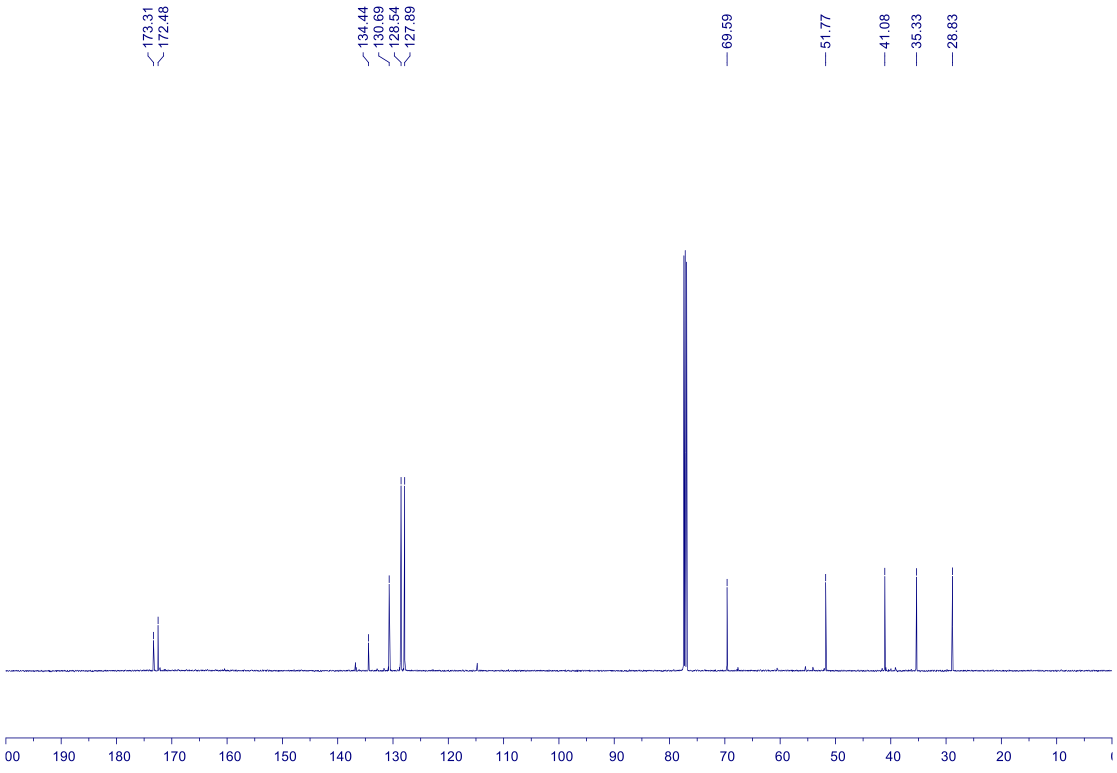

**7g** – $^{1}$H NMR (400 MHz, CDCl$_3$)

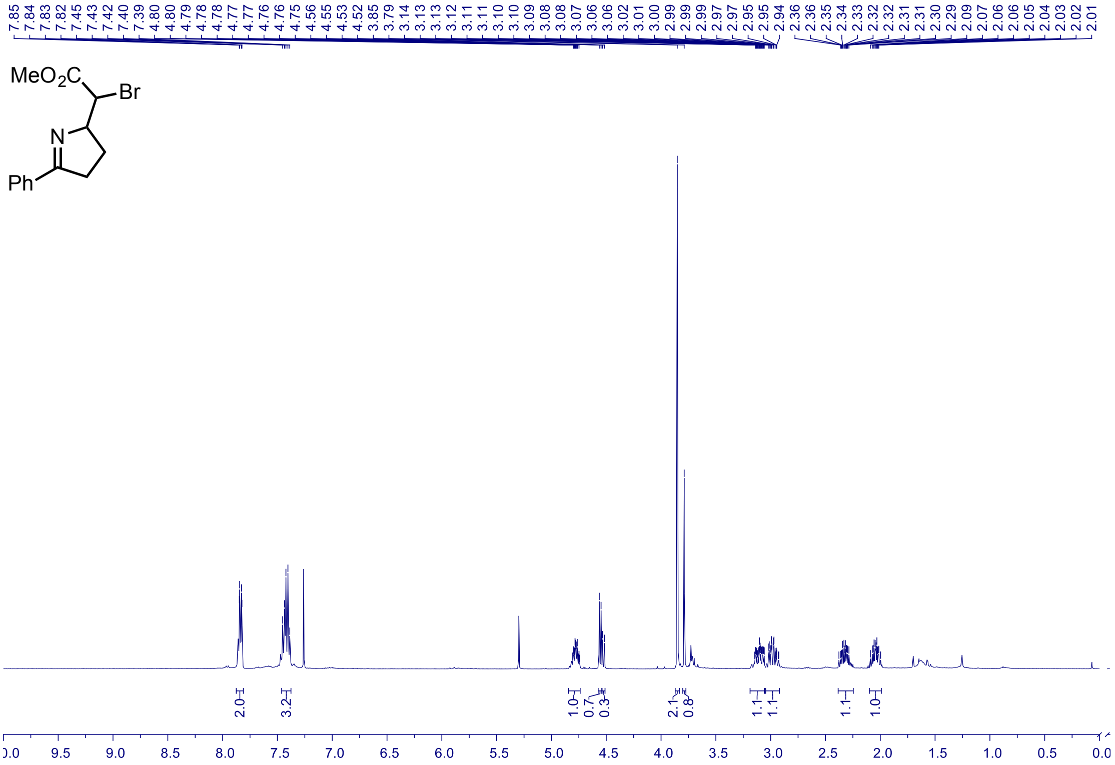


**7g** – $^{13}$C NMR (101 MHz, CDCl$_3$)

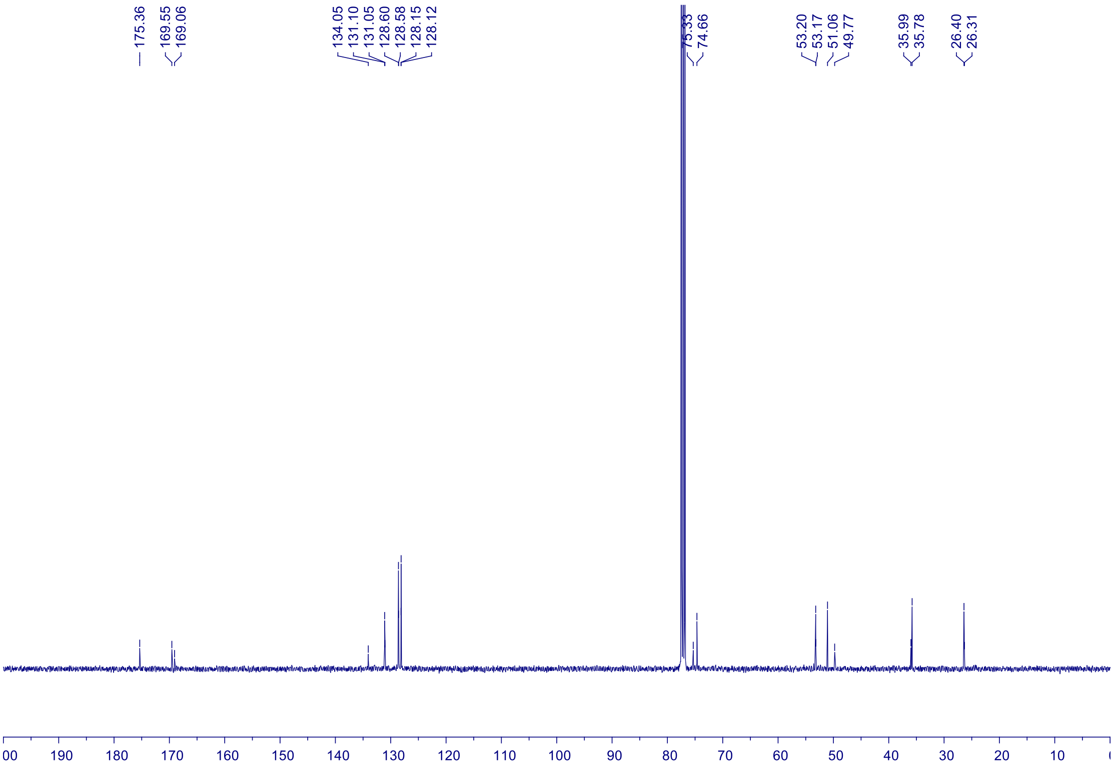

**7h** – $^{1}$H NMR (500 MHz, toluene-$d_9$, T = 100 ºC)

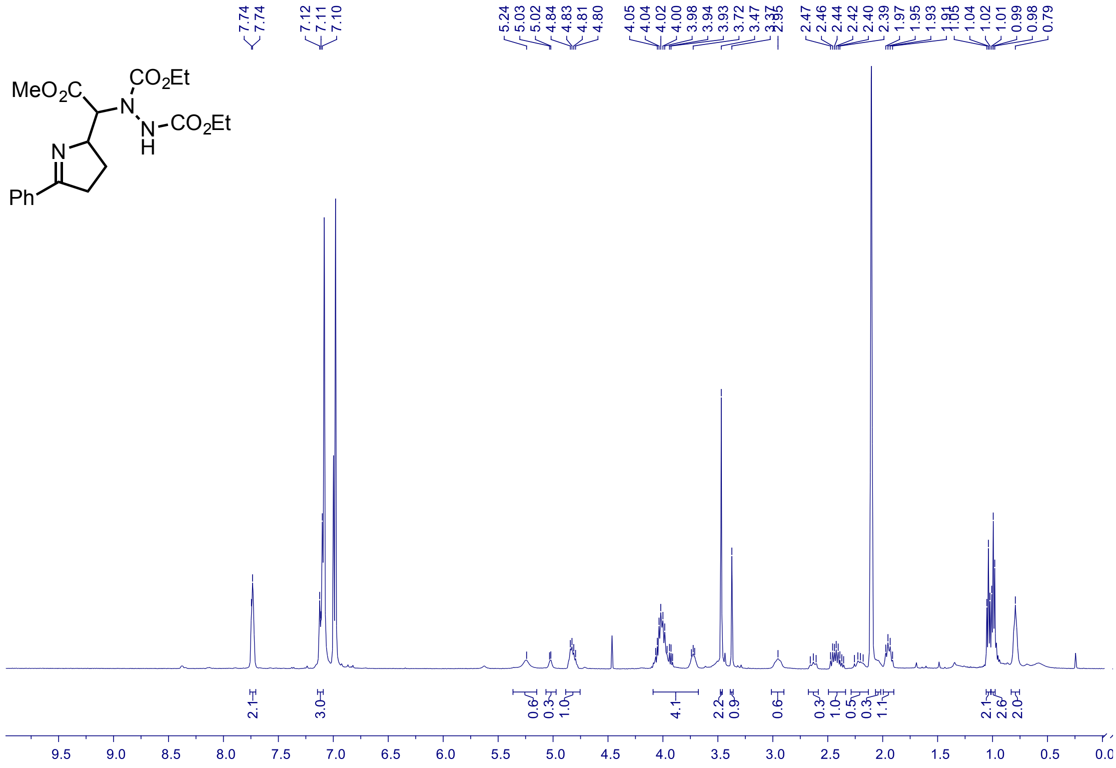

**7h** – $^{13}$C NMR (125 MHz, toluene-$d_9$, T = 100 ºC)

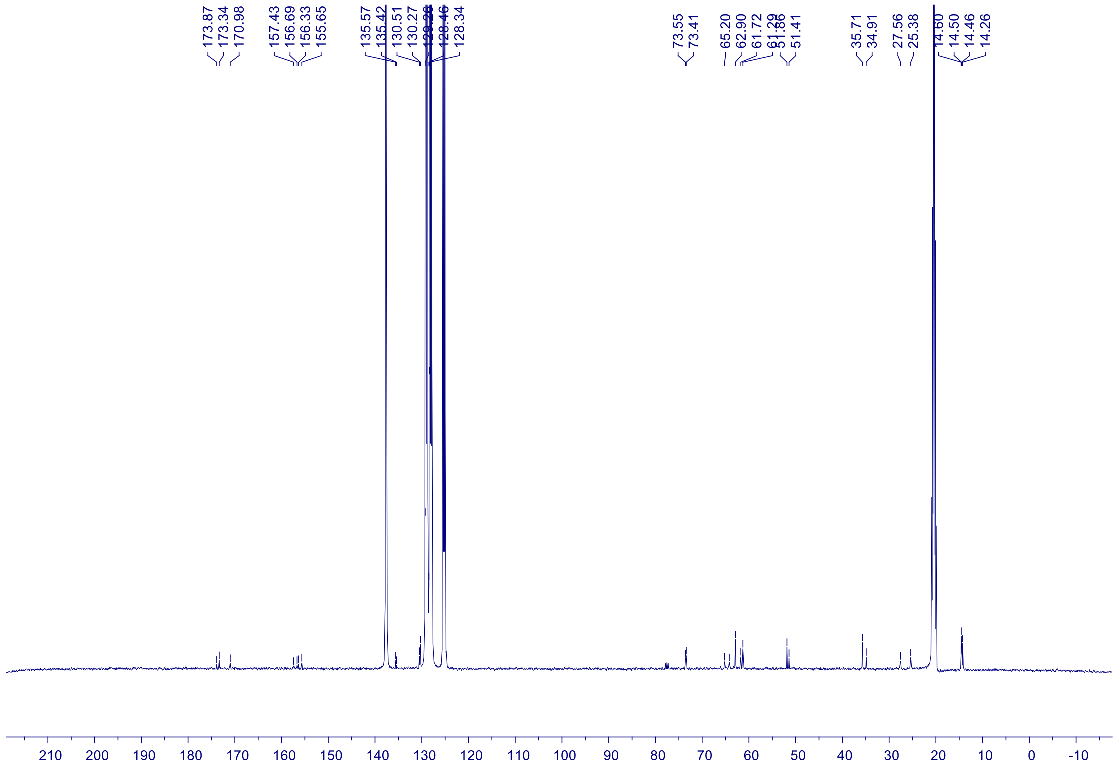

**7i** – $^{1}$H NMR (400 MHz, $CDCl_3$)

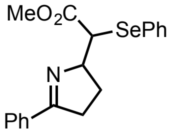


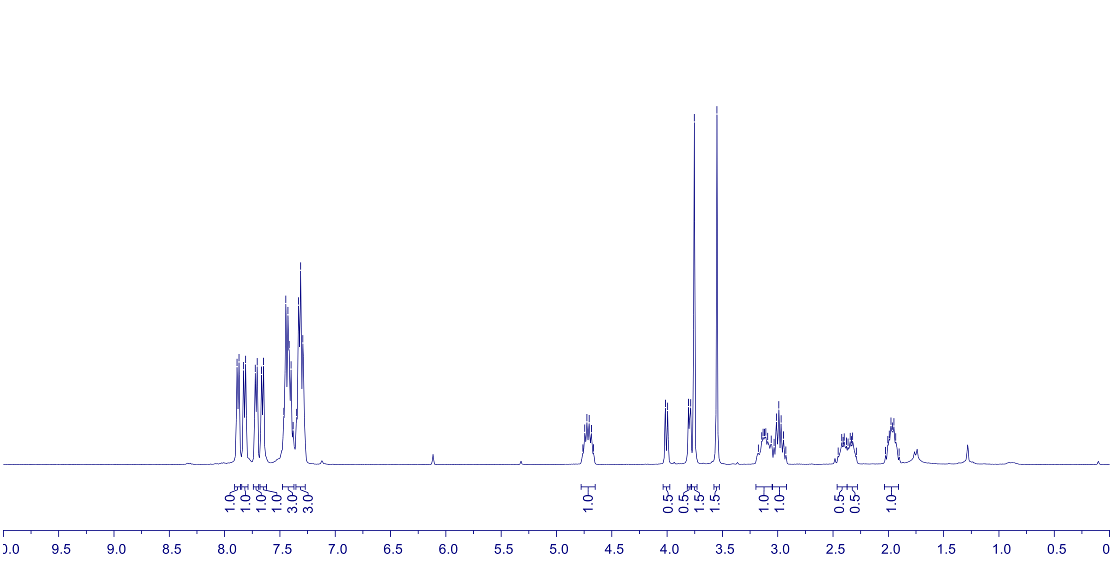


**7i** – $^{13}$C NMR (101 MHz, $CDCl_3$)

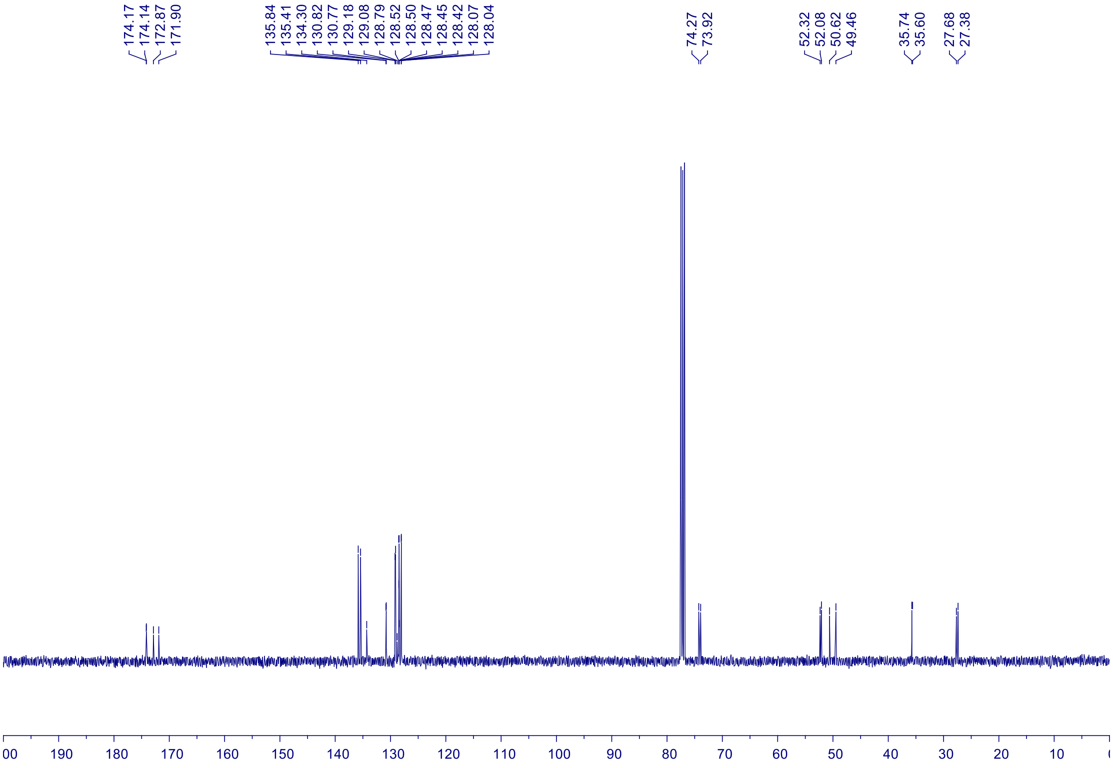

**7j** – $^{1}$H NMR (500 MHz, $CDCl_3$)

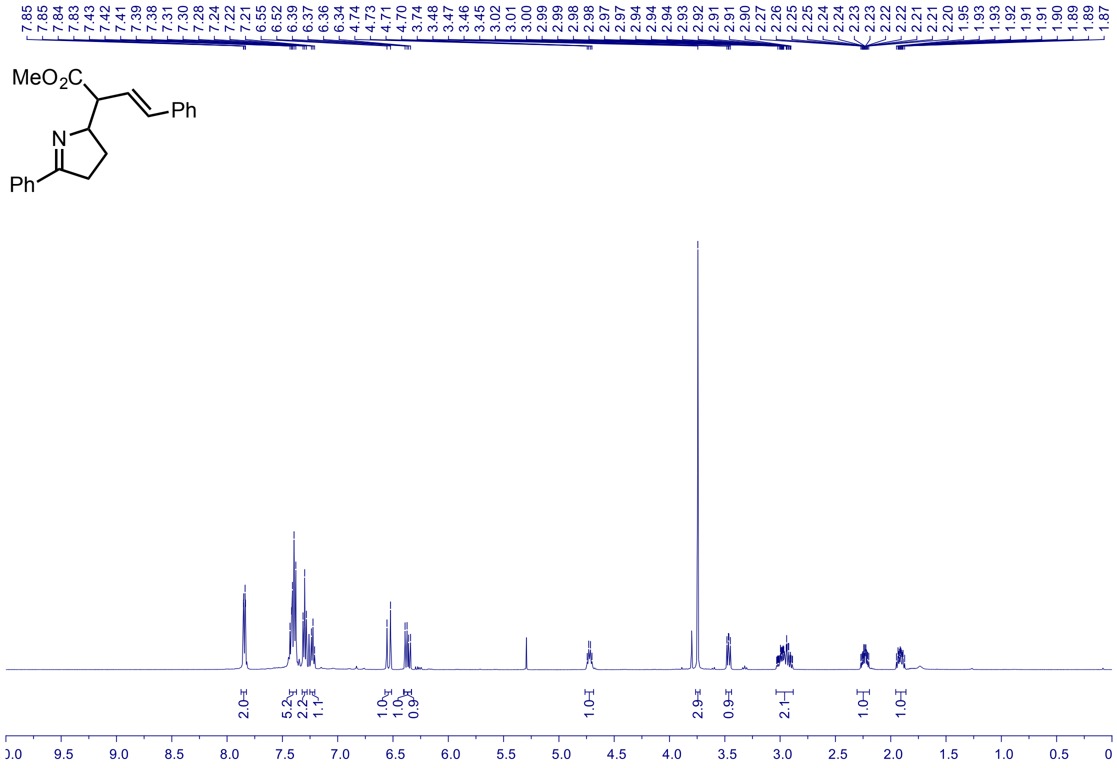


**7j** – $^{13}$C NMR (126 MHz, $CDCl_3$)

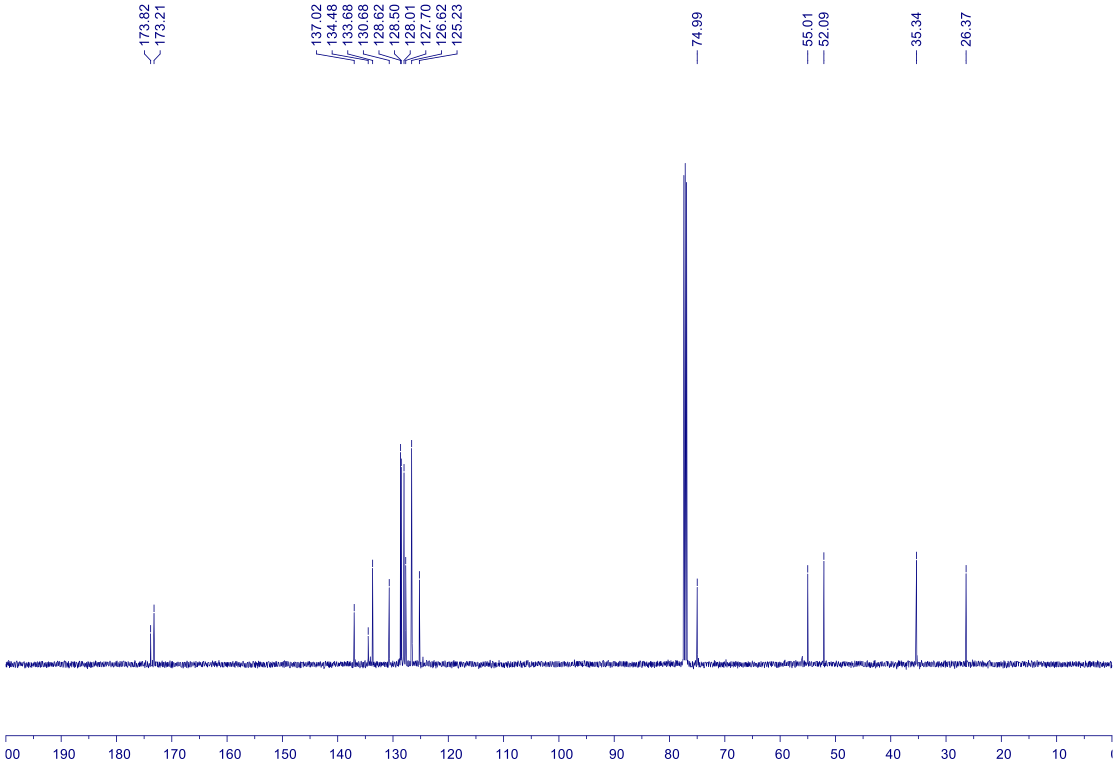

**7k** – ¹H NMR (400 MHz, $CDCl_3$)

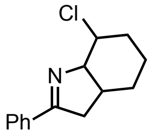


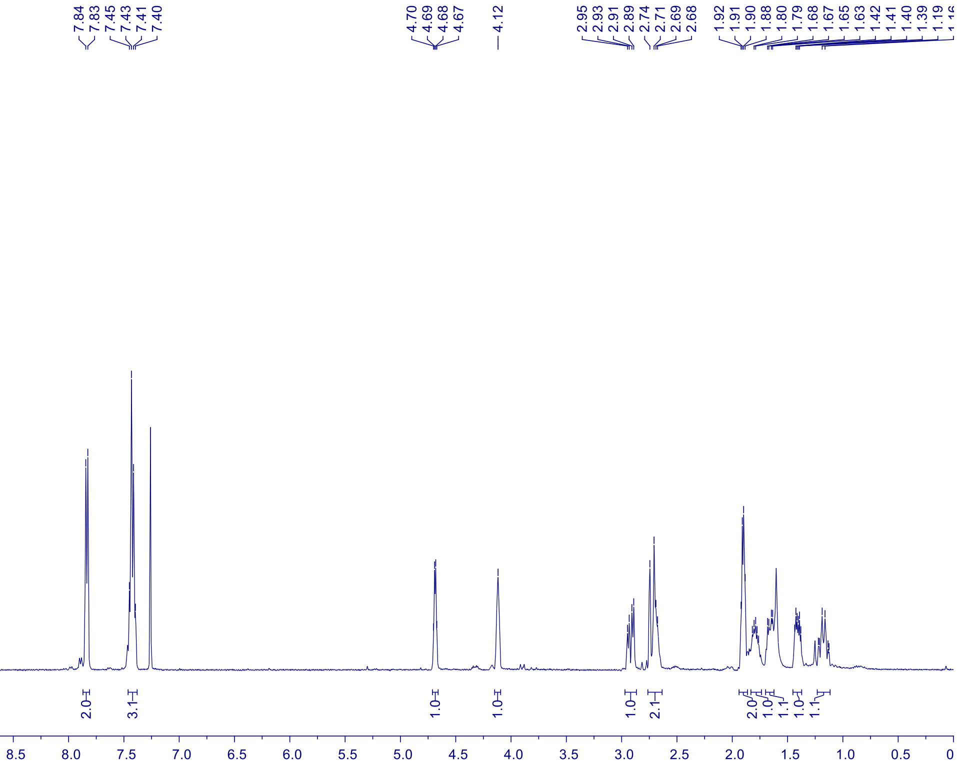


**7k** – ¹³C NMR (101 MHz, $CDCl_3$)

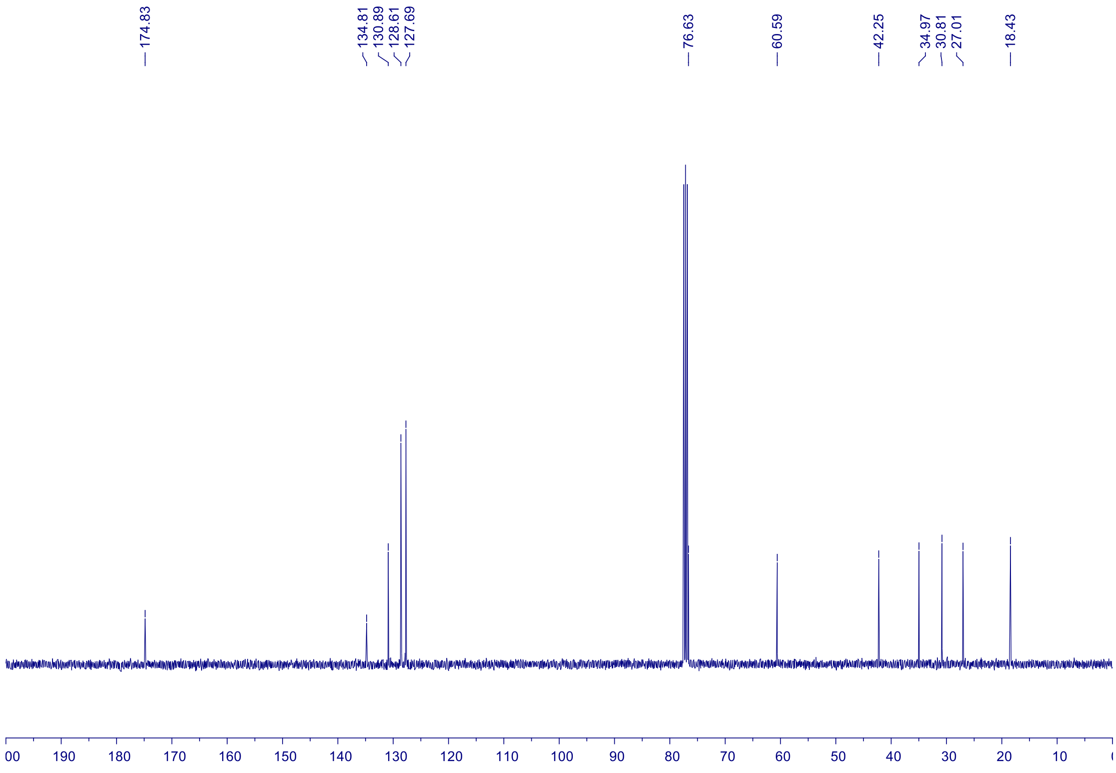

**7l** – $^{1}$H NMR (400 MHz, $CDCl_3$)

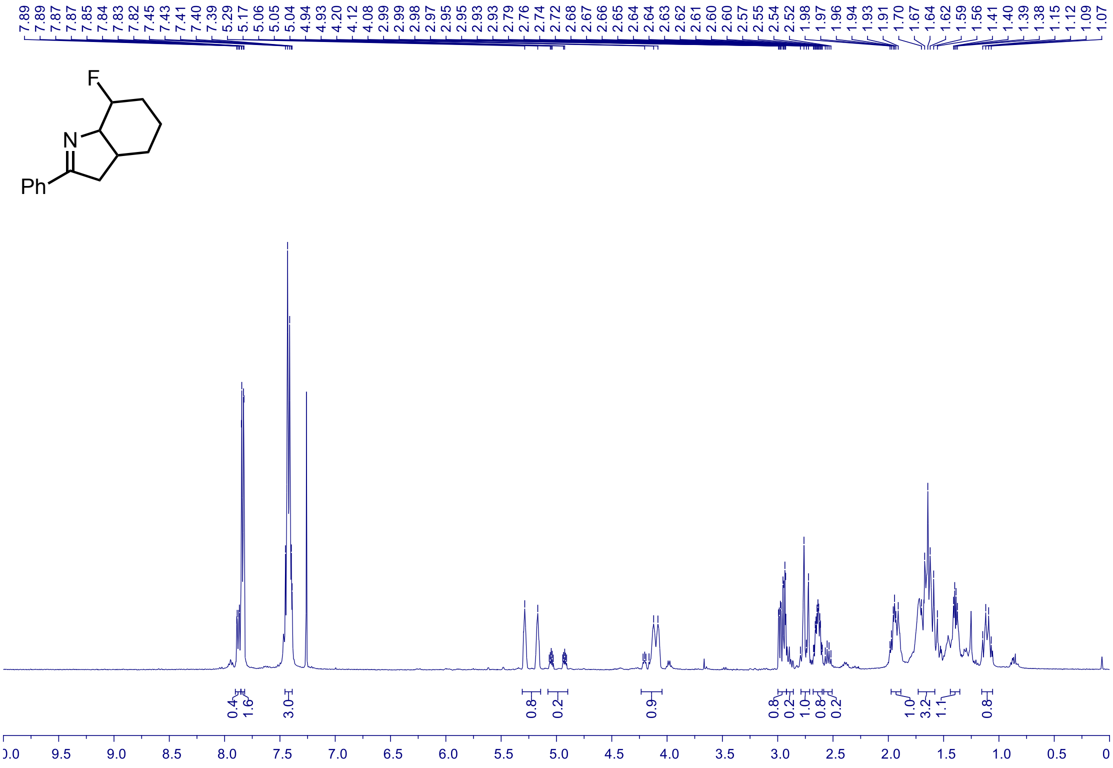


**7l** – $^{13}$C NMR (101 MHz, $CDCl_3$)

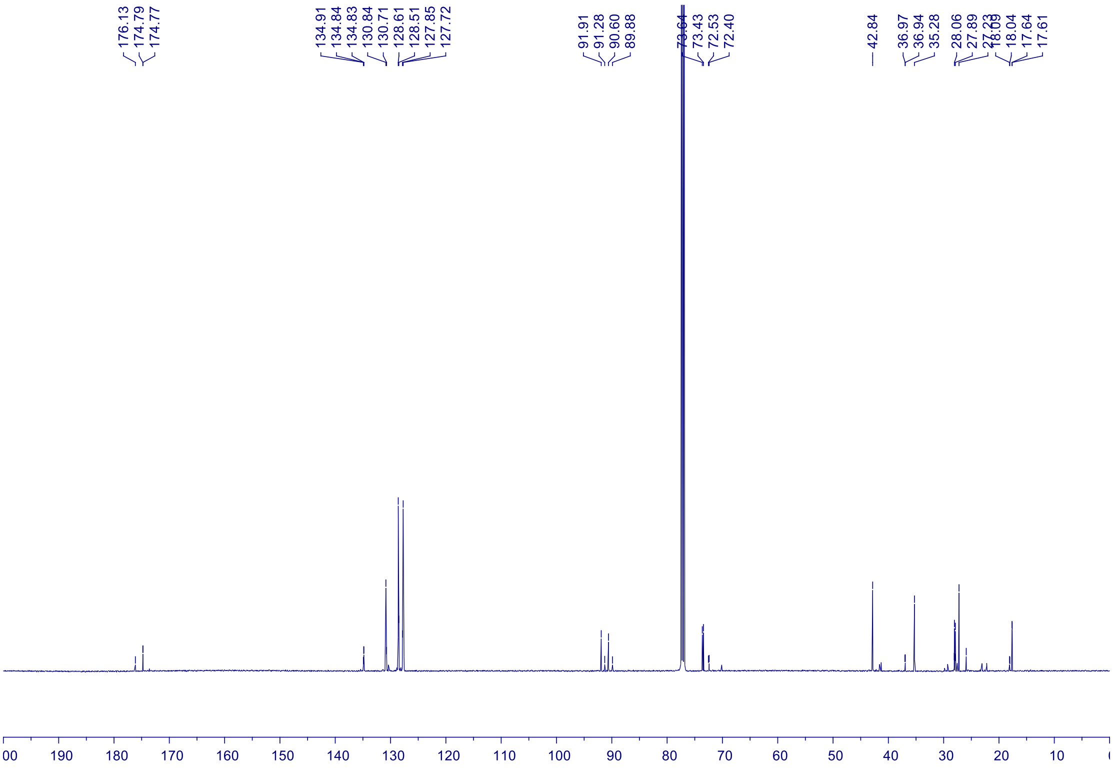

**7l** – $^{19}$F NMR (376 MHz, $CDCl_3$)

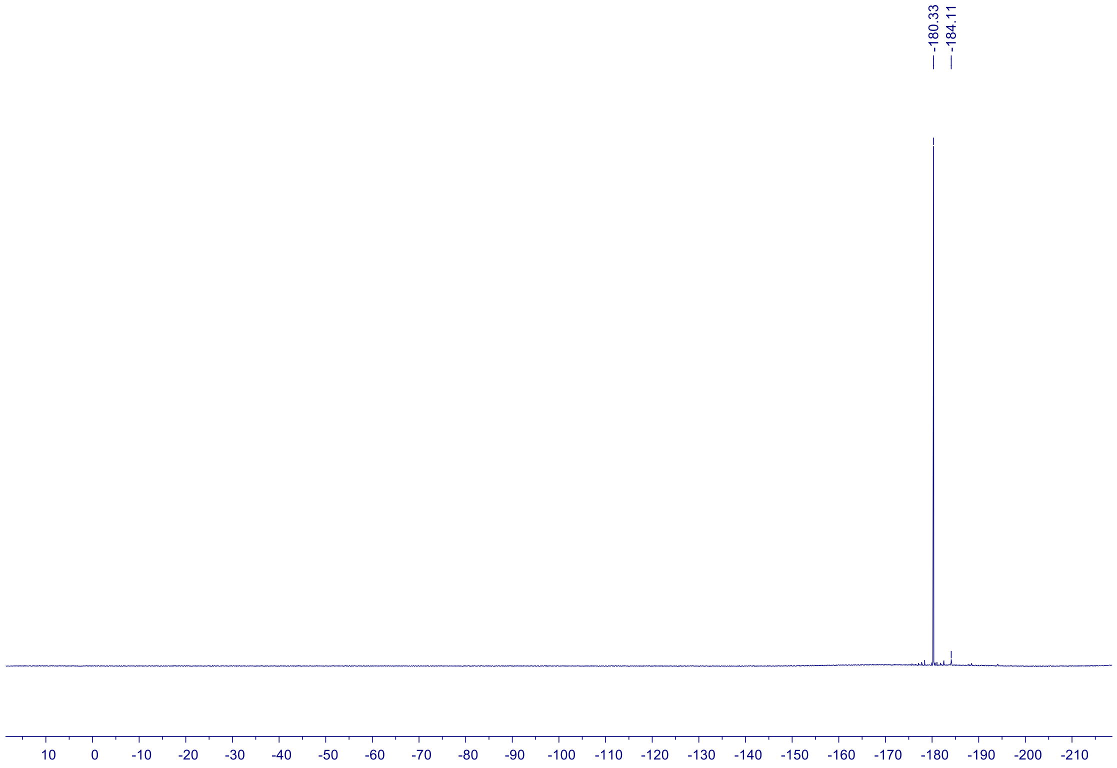

**7m** – $^{1}$H NMR (400 MHz, $CDCl_3$)

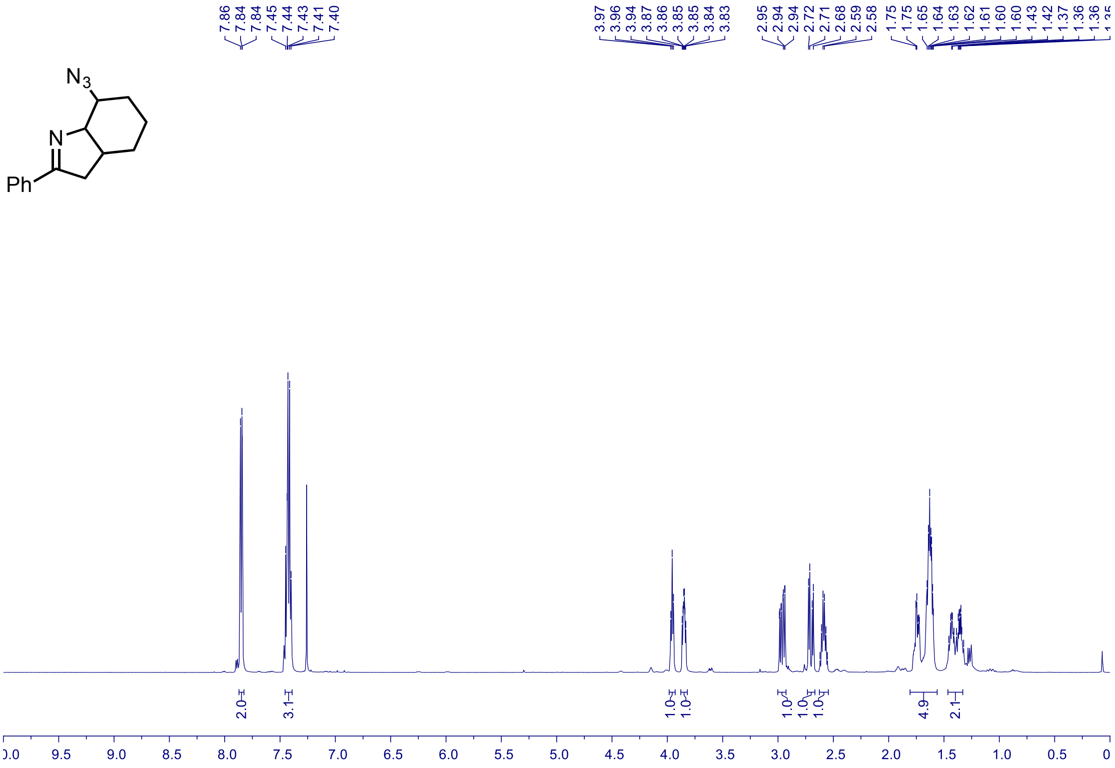


**7m** – $^{13}$C NMR (101 MHz, $CDCl_3$)

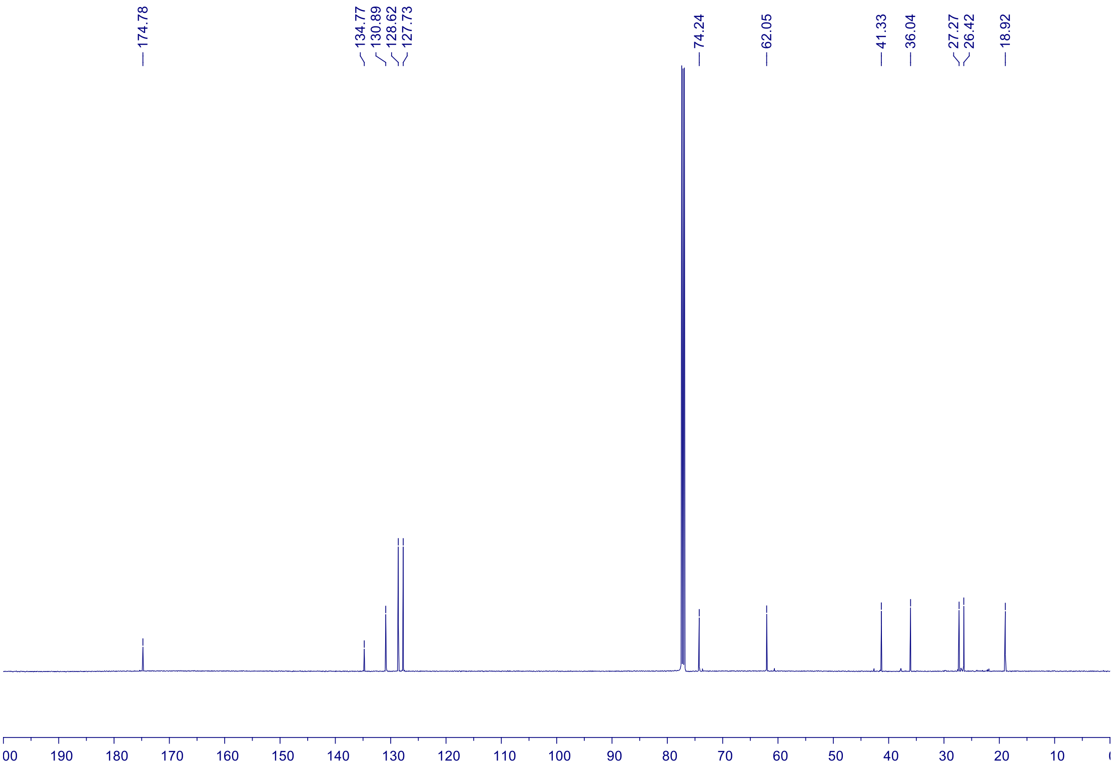

**7n** – $^{1}$H NMR (500 MHz, CDCl$_3$)

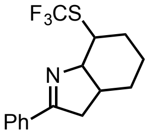

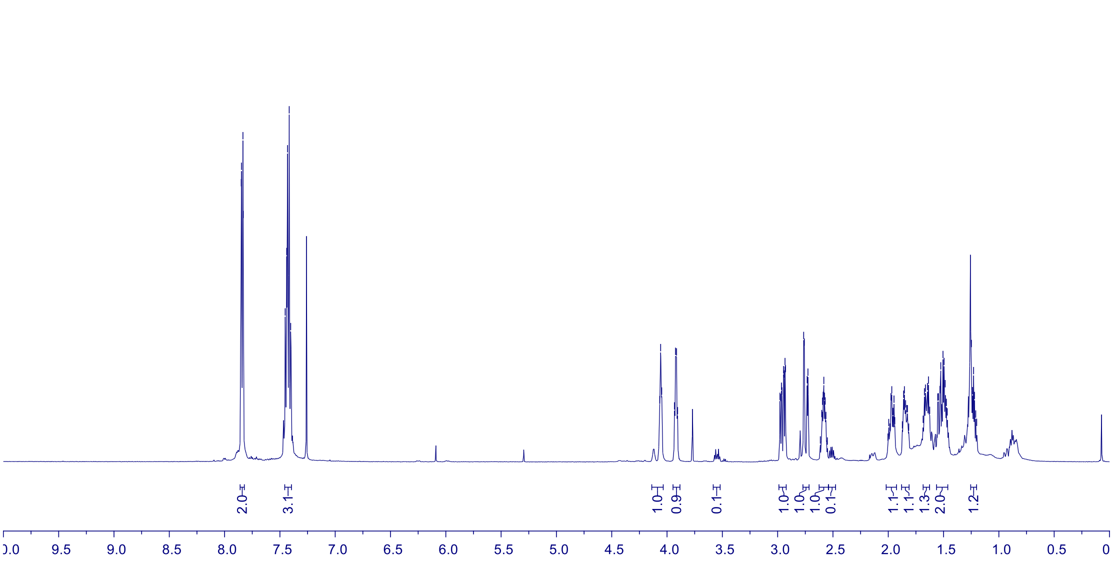

**7n** – $^{13}$C NMR (126 MHz, CDCl$_3$)

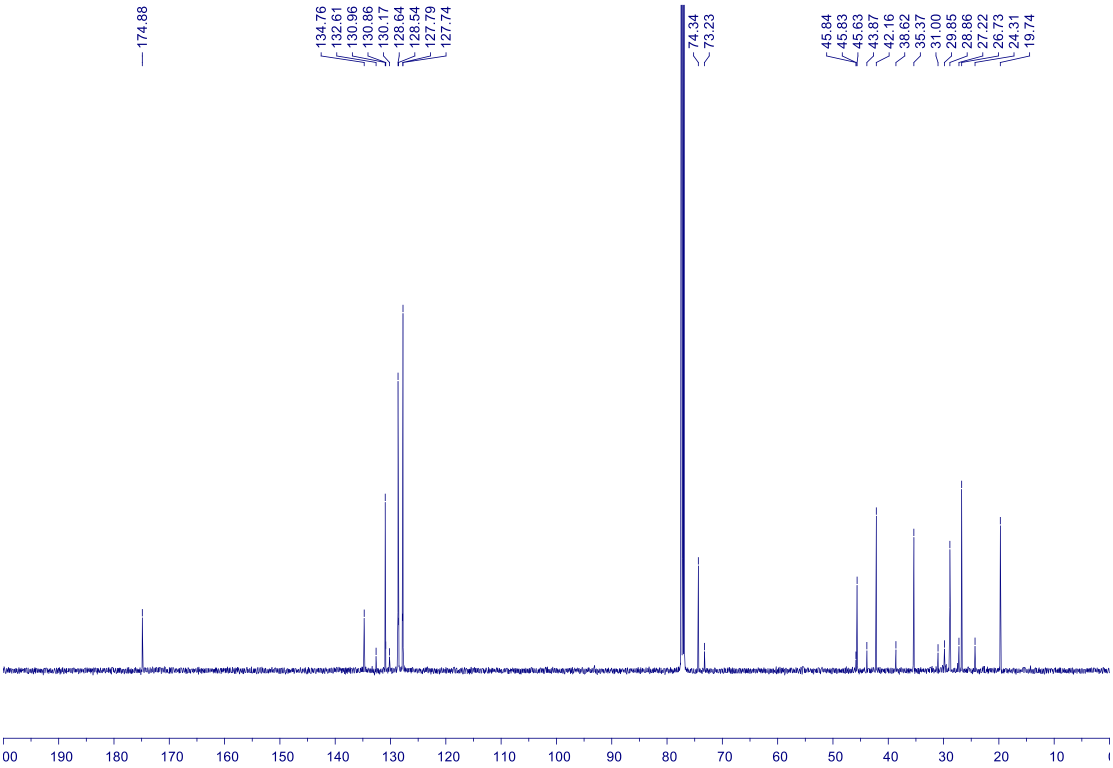

**7n** – $^{19}$F NMR (376 MHz, $CDCl_3$)

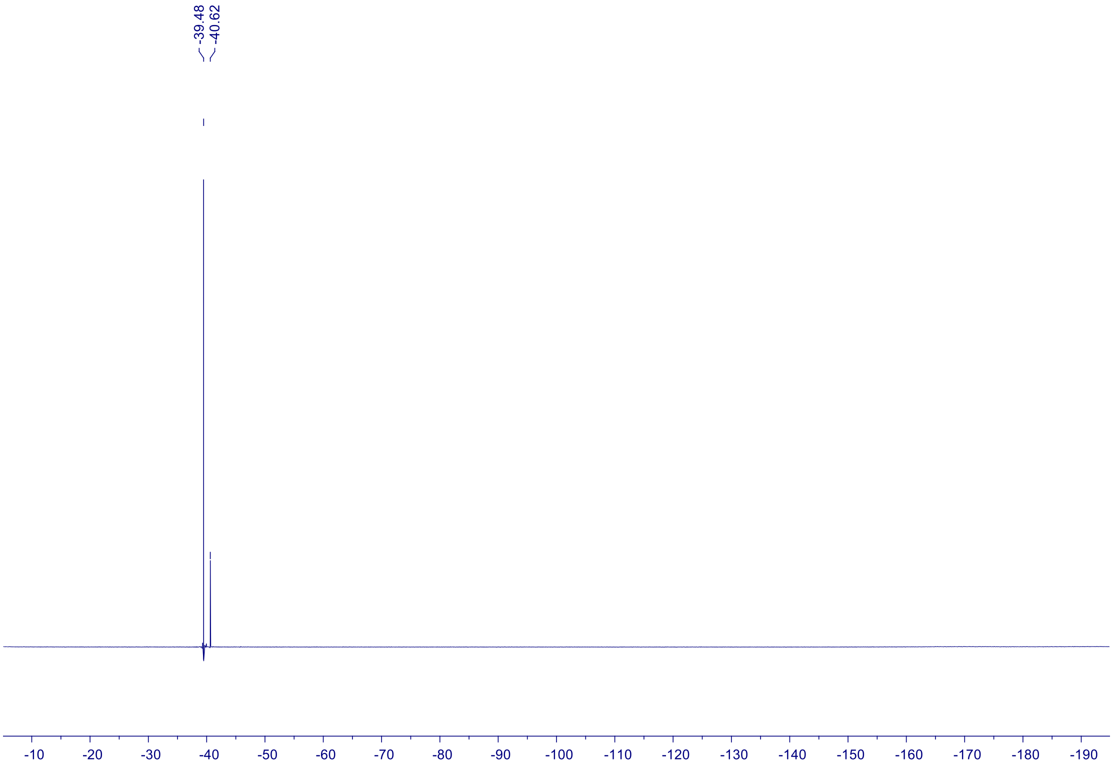

**7o** – $^{1}$H NMR (400 MHz, $CDCl_3$)

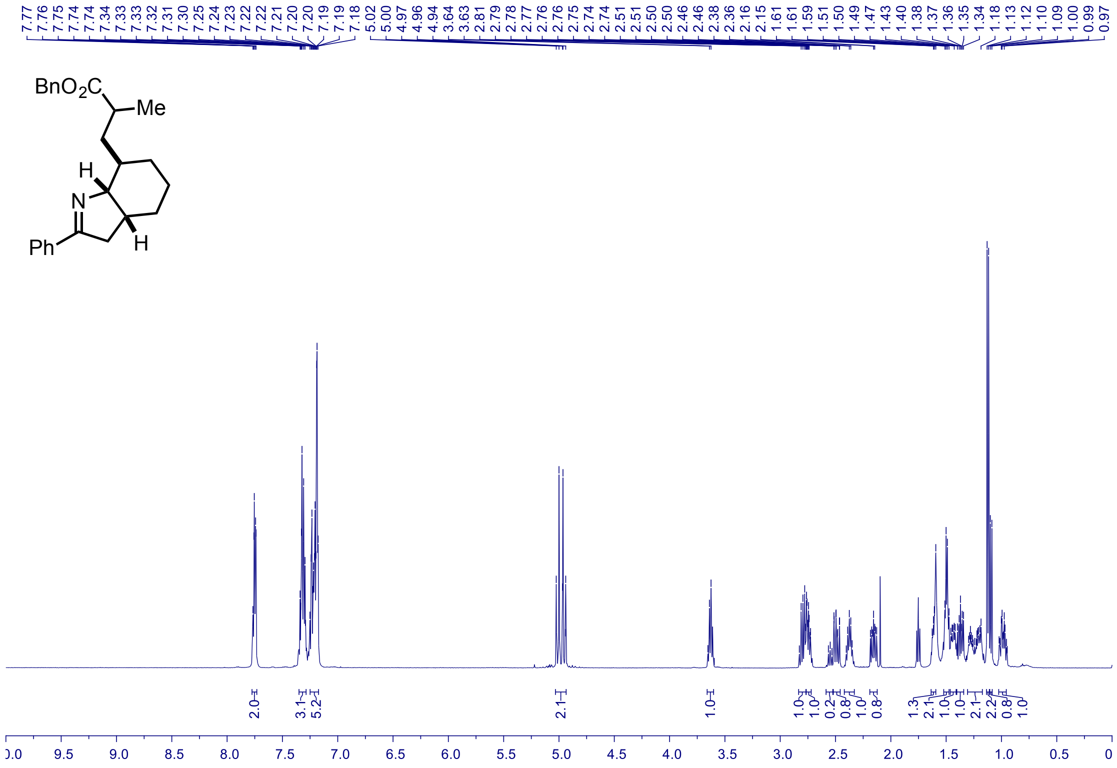


**7o** – $^{13}$C NMR (101 MHz, $CDCl_3$)

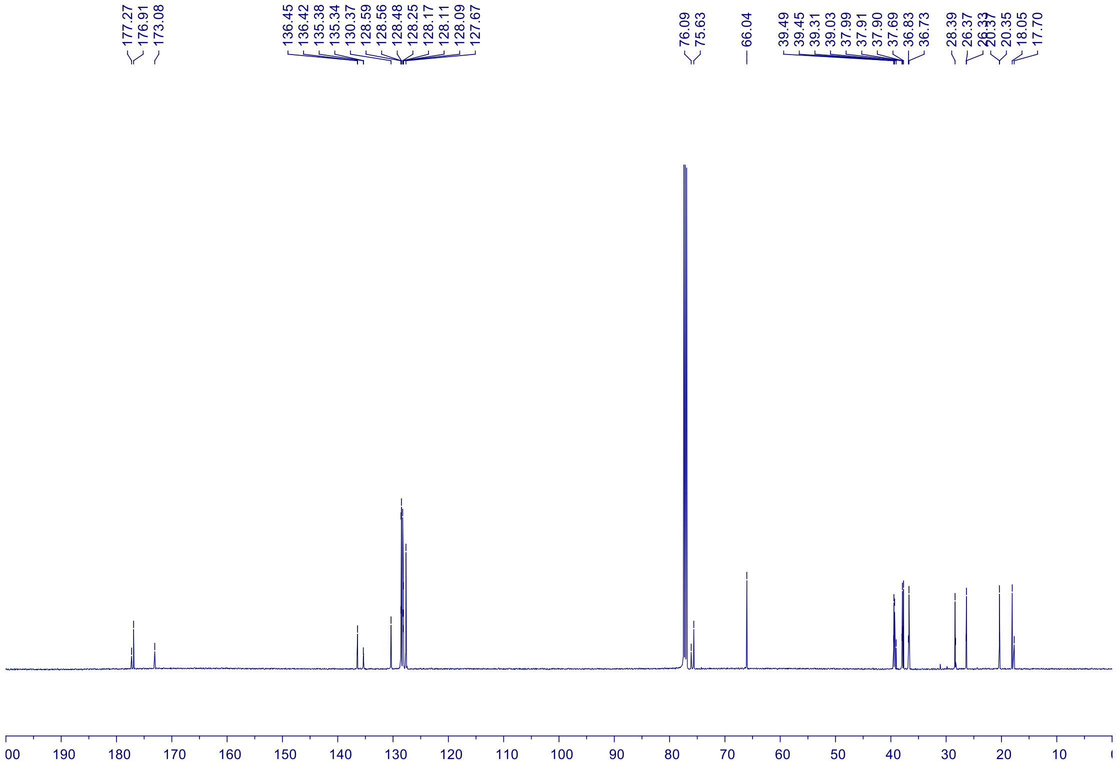

**7p** – $^{1}$H NMR (400 MHz, $CDCl_3$)

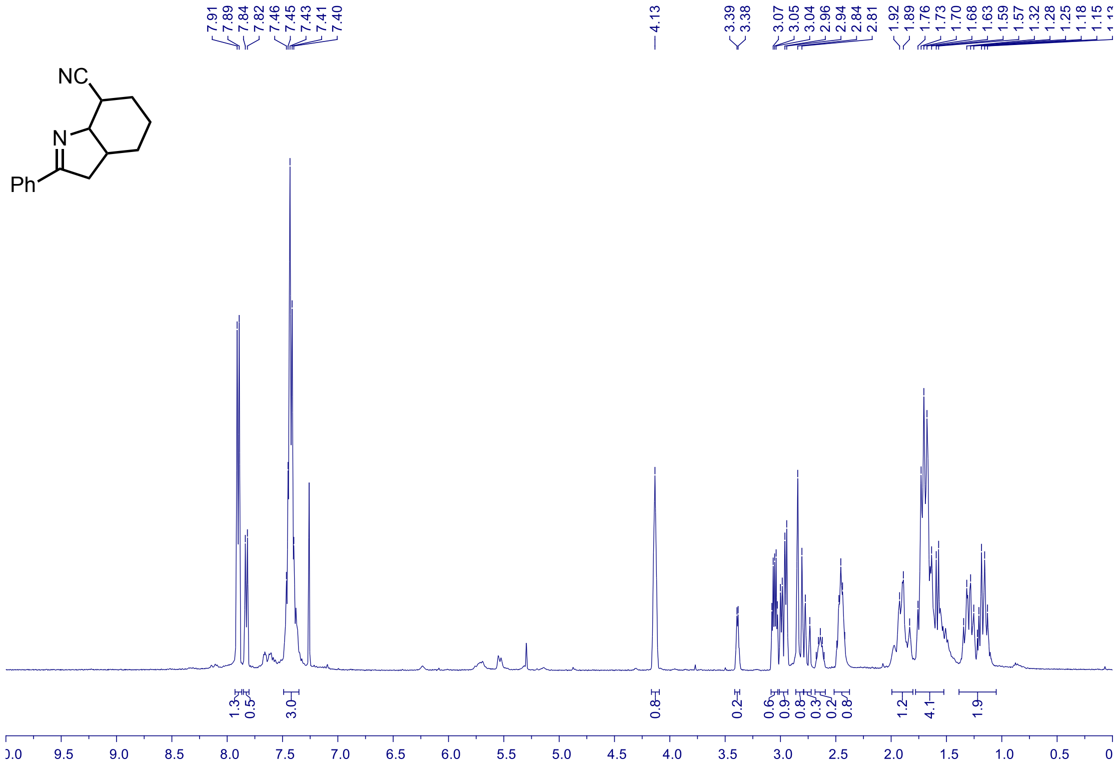

**7p** – $^{13}$C NMR (101 MHz, $CDCl_3$)

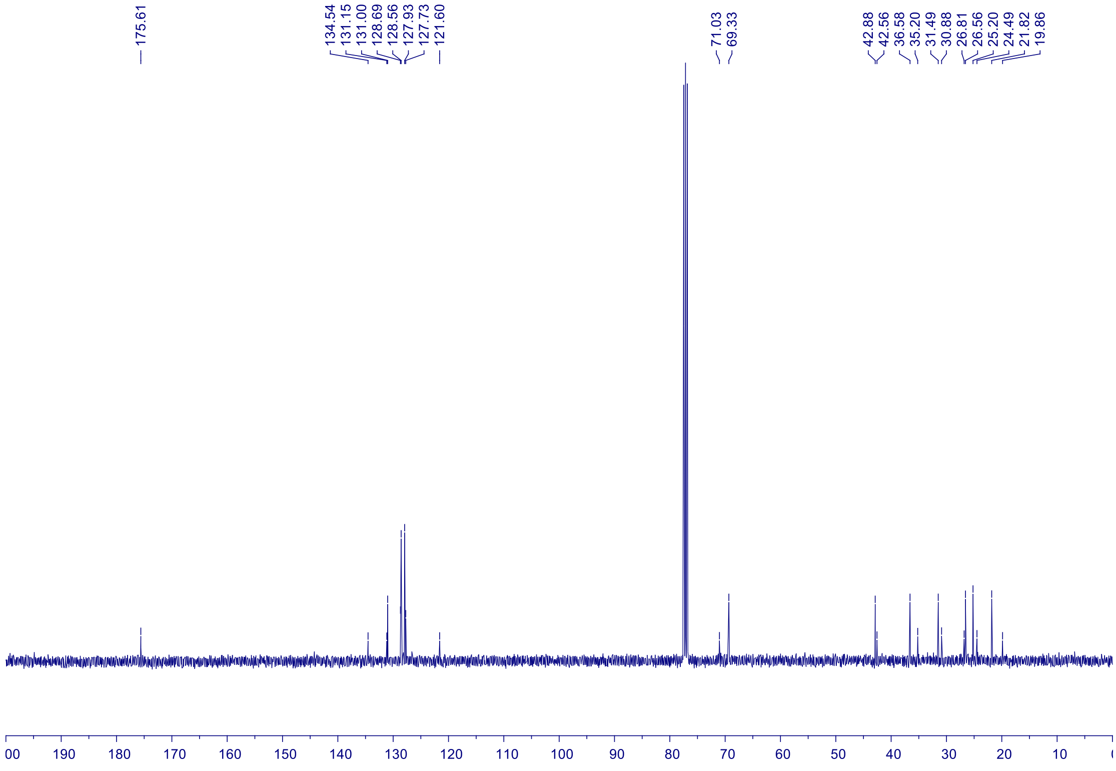

**7q** – $^{1}$H NMR (500 MHz, CDCl$_3$)

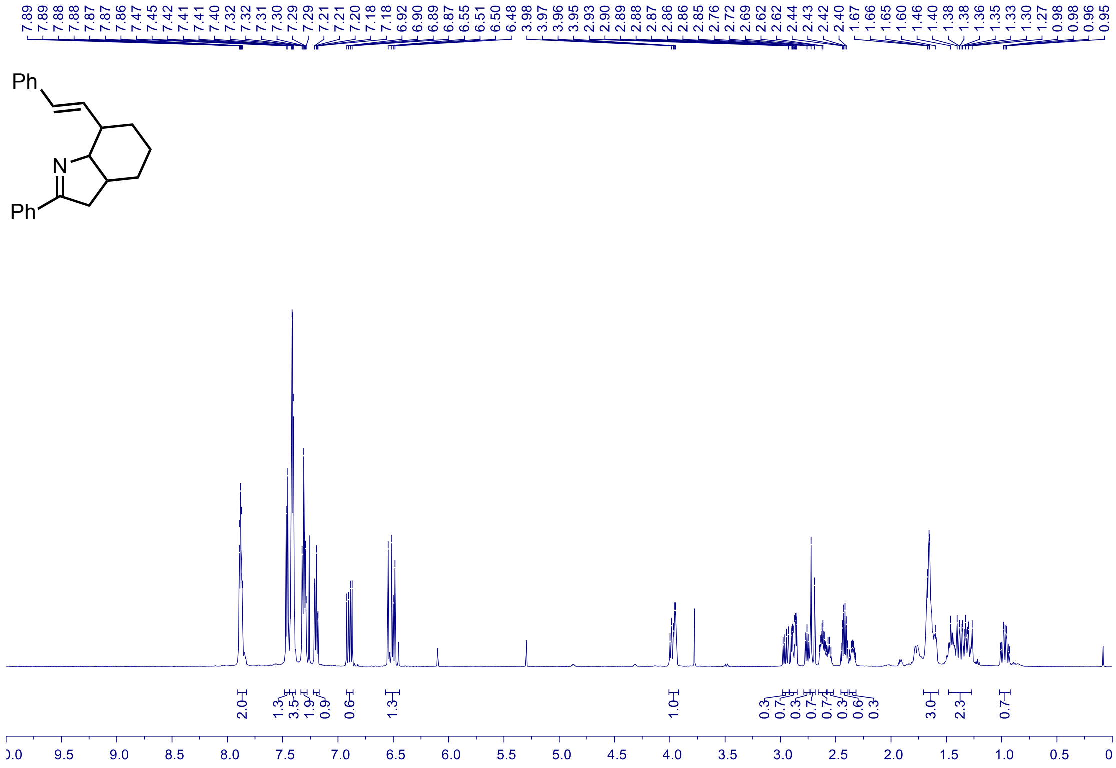

**7q** – $^{13}$C NMR (126 MHz, CDCl$_3$)

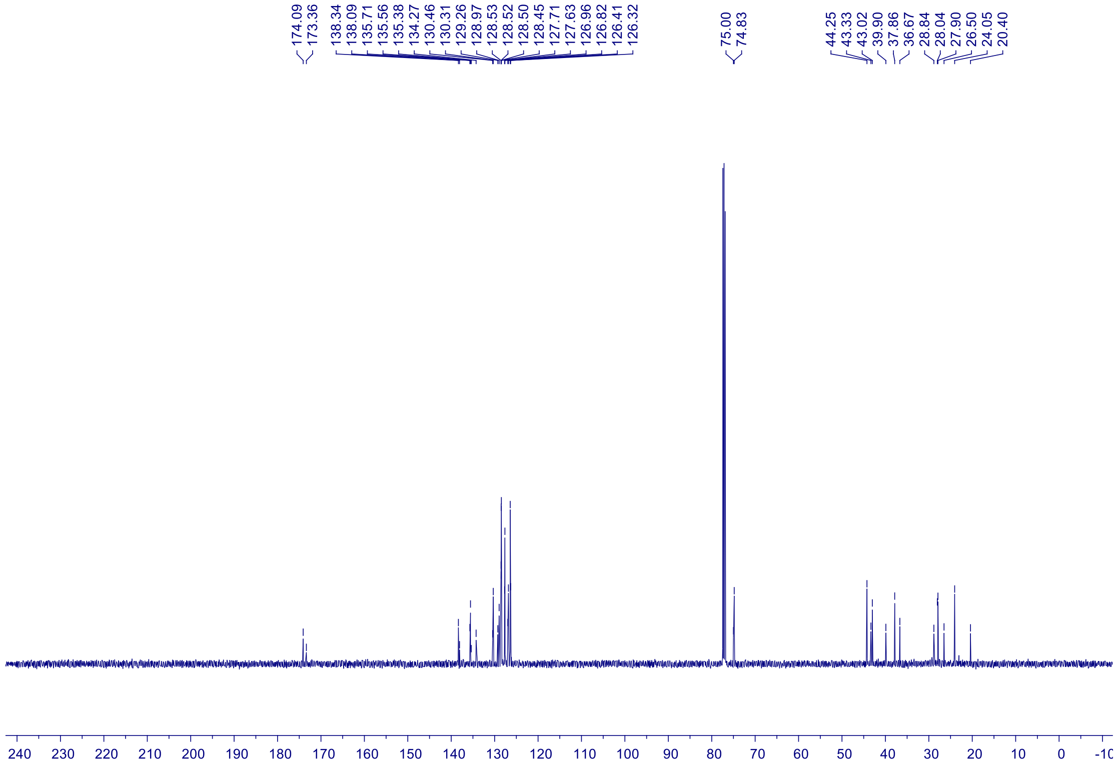

**7r** – $^{1}$H NMR (500 MHz, $CDCl_3$)

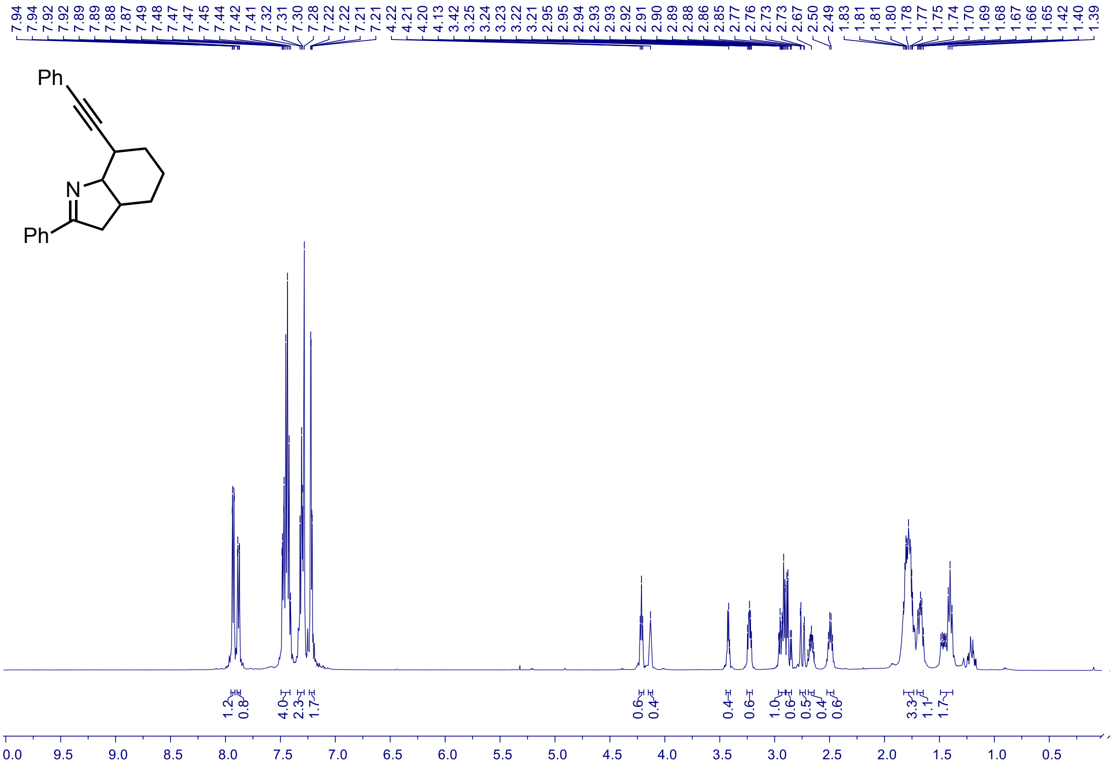


**7r** – $^{13}$C NMR (126 MHz, $CDCl_3$)

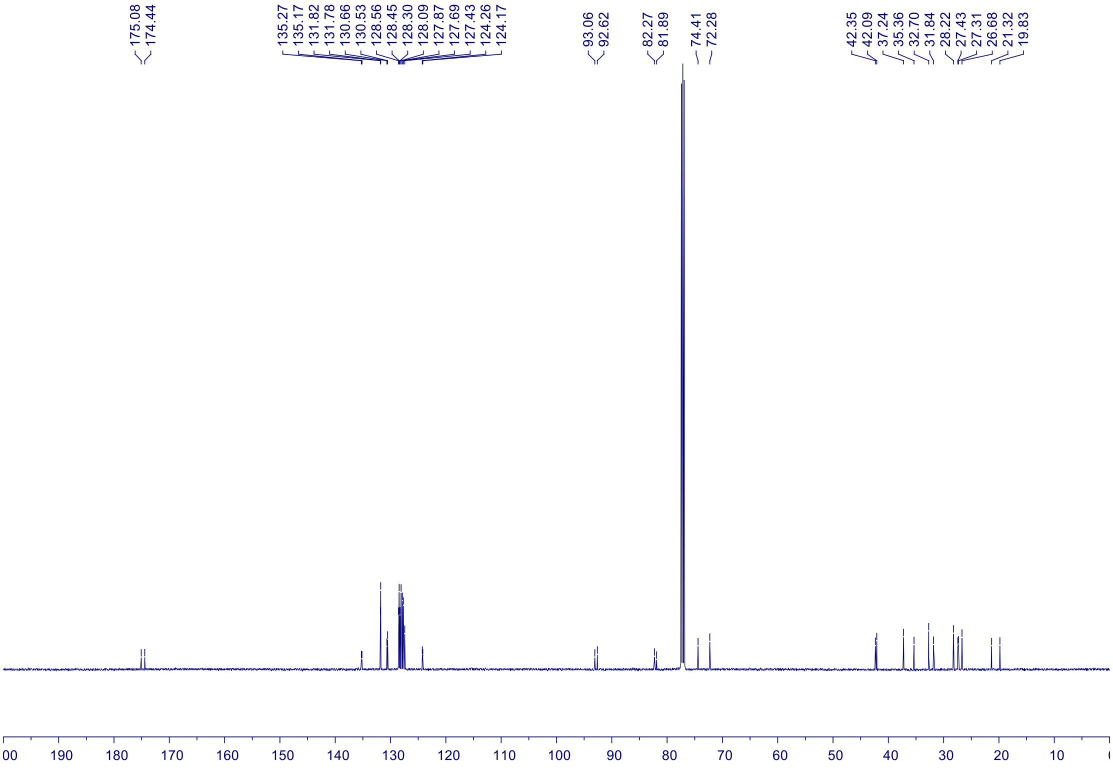

**8a** – $^1$H NMR (400 MHz, CDCl$_3$)

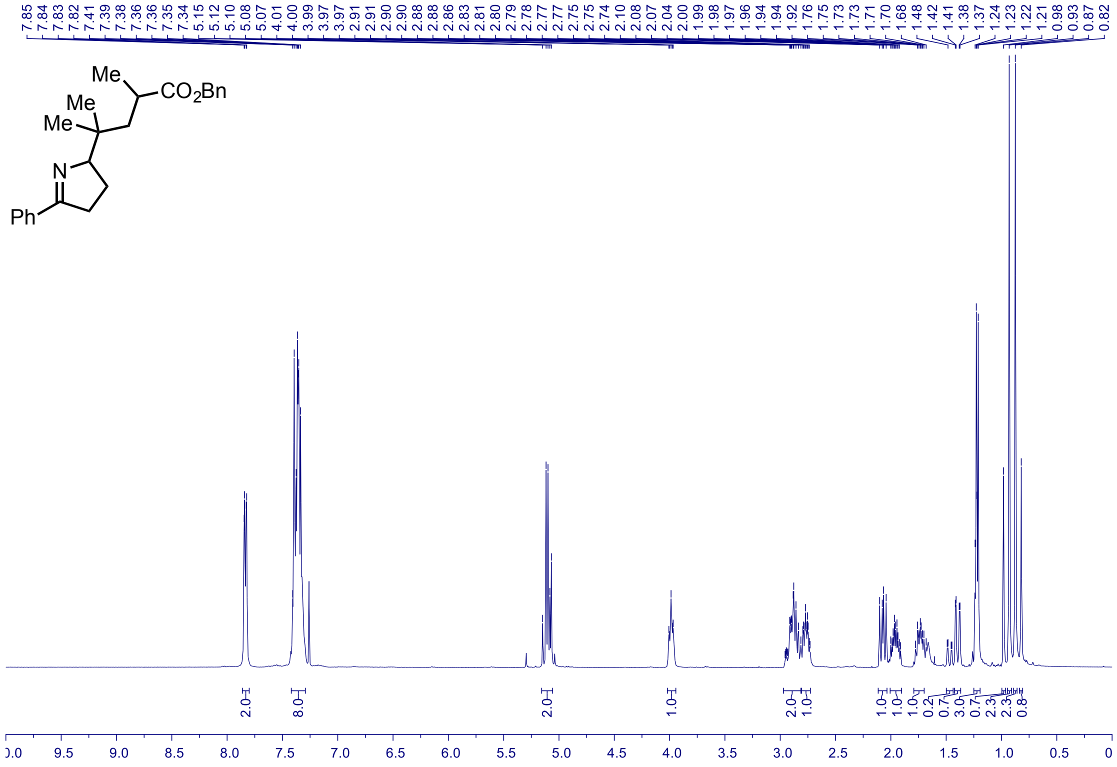


**8a** – $^{13}$C NMR (101 MHz, CDCl$_3$)

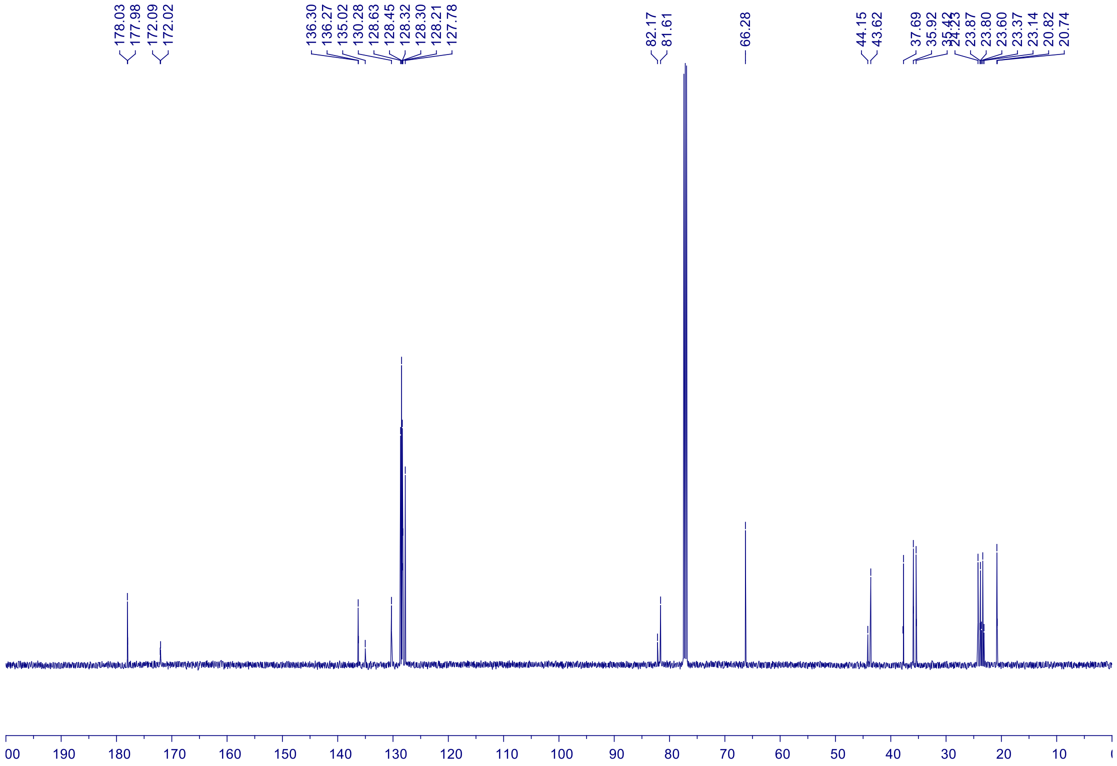

**8b** – $^{1}$H NMR (400 MHz, $CDCl_3$)

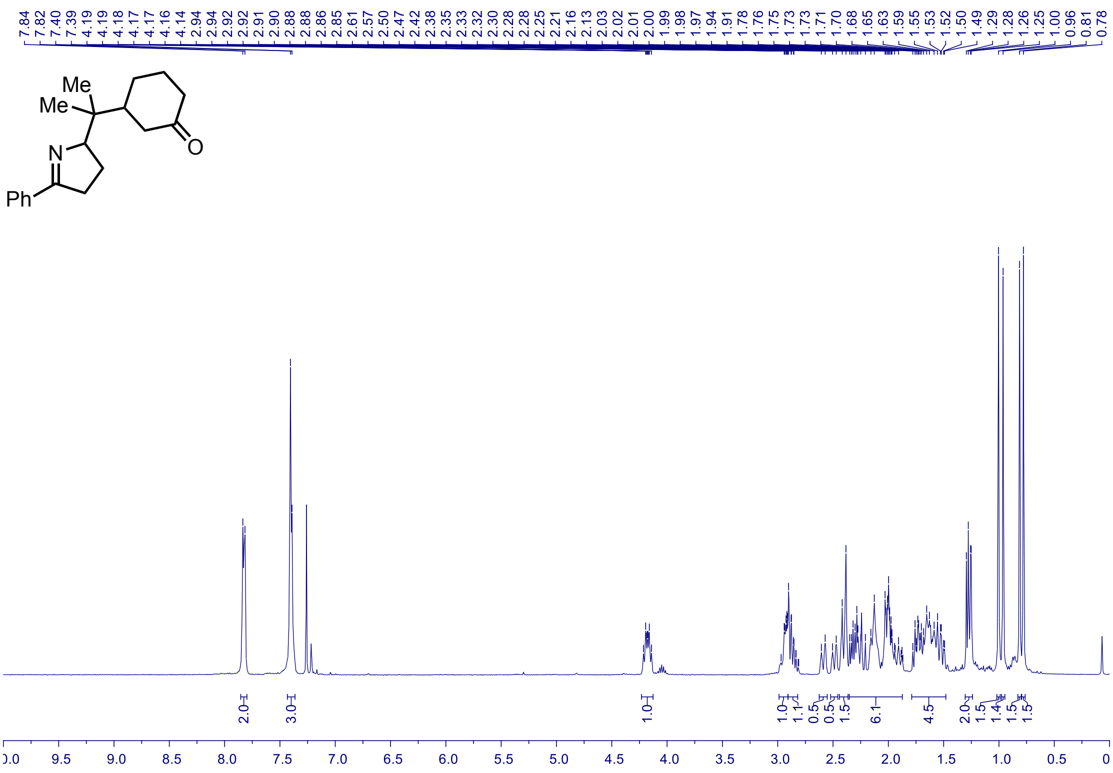

**8b** – $^{13}$C NMR (101 MHz, $CDCl_3$)

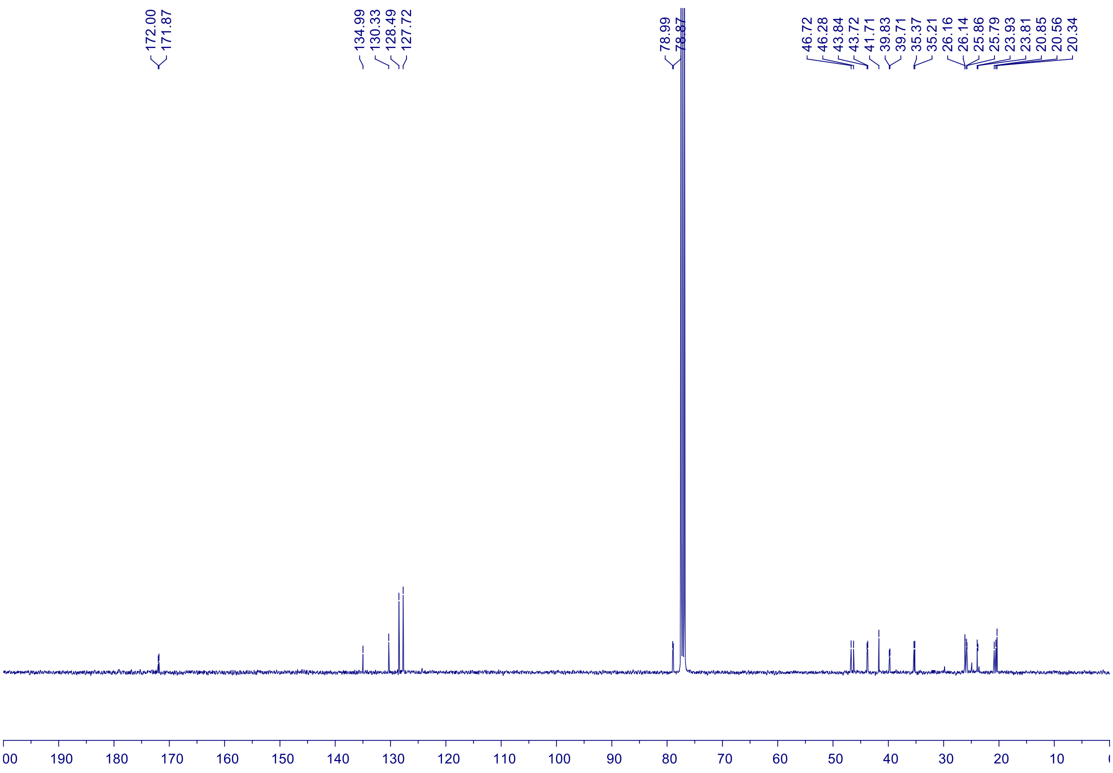

**8c** – $^{1}$H NMR (400 MHz, CDCl$_3$)

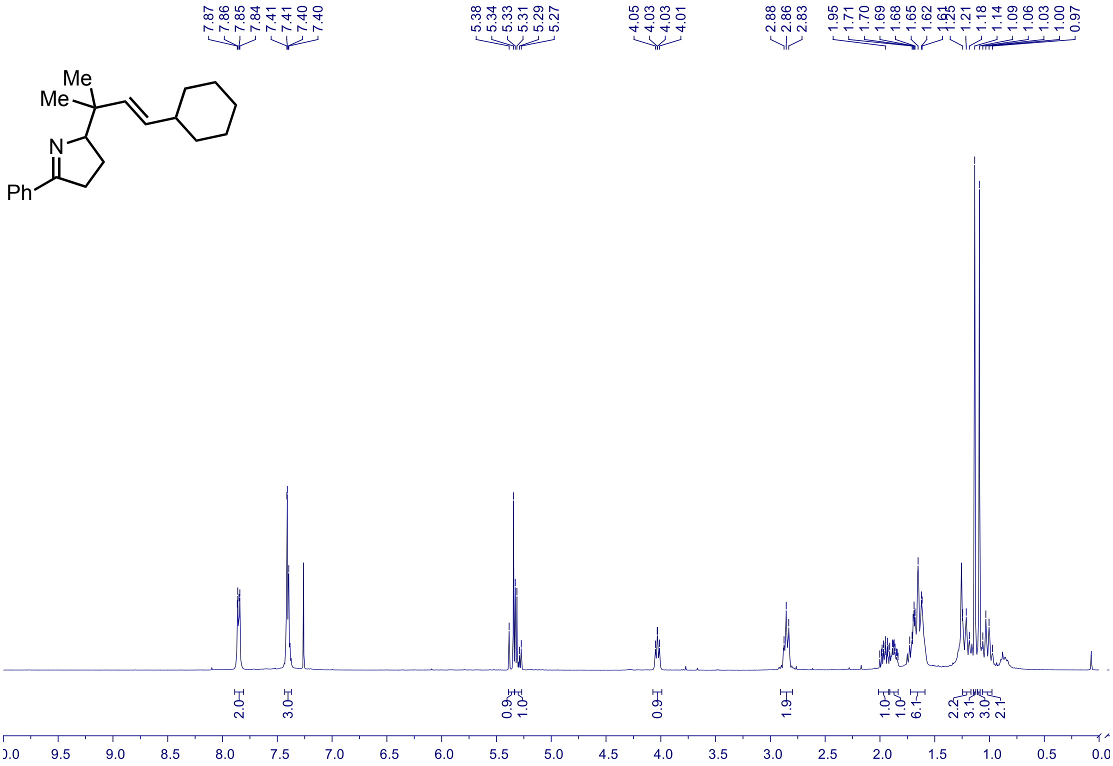


**8c** – $^{13}$C NMR (101 MHz, CDCl$_3$)

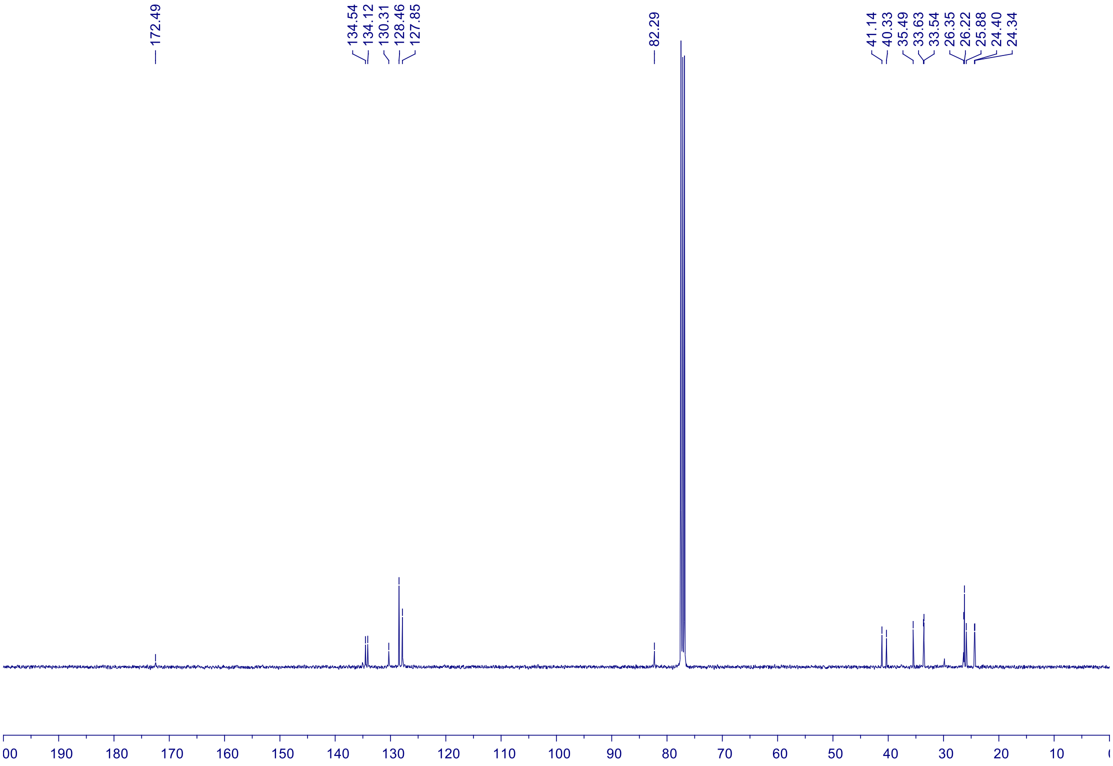

**8d** – $^{1}$H NMR (500 MHz, $CDCl_3$)

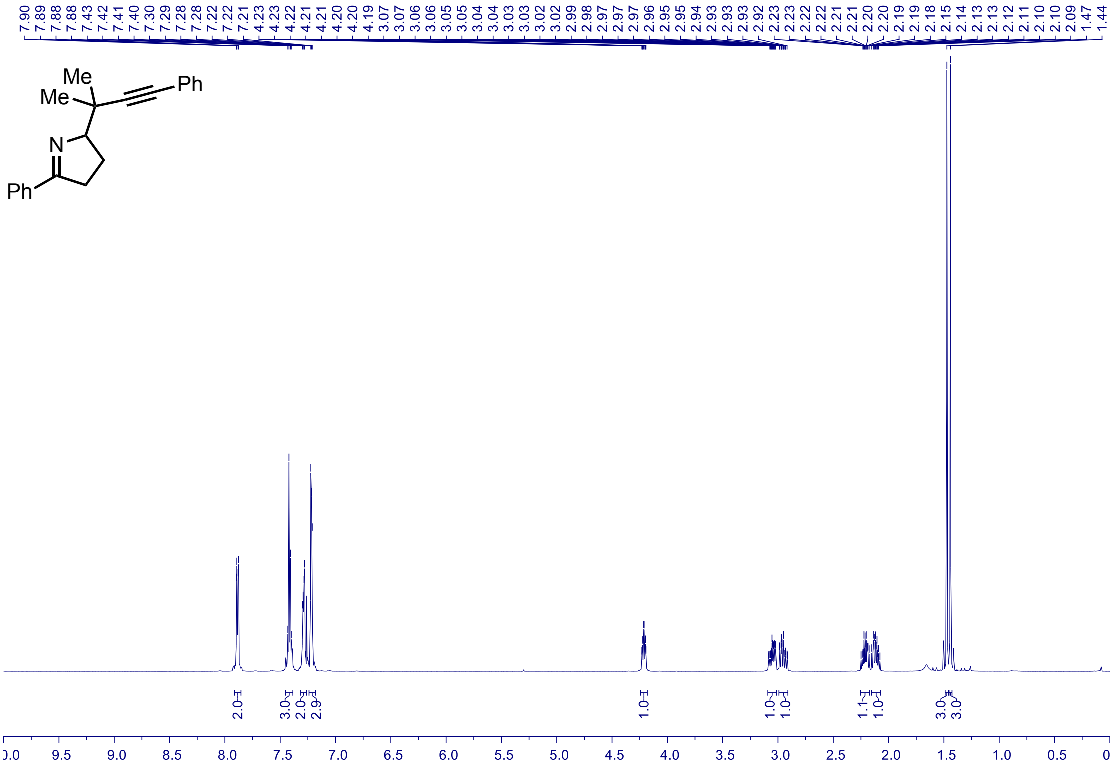


**8d** – $^{13}$C NMR (126 MHz, $CDCl_3$)

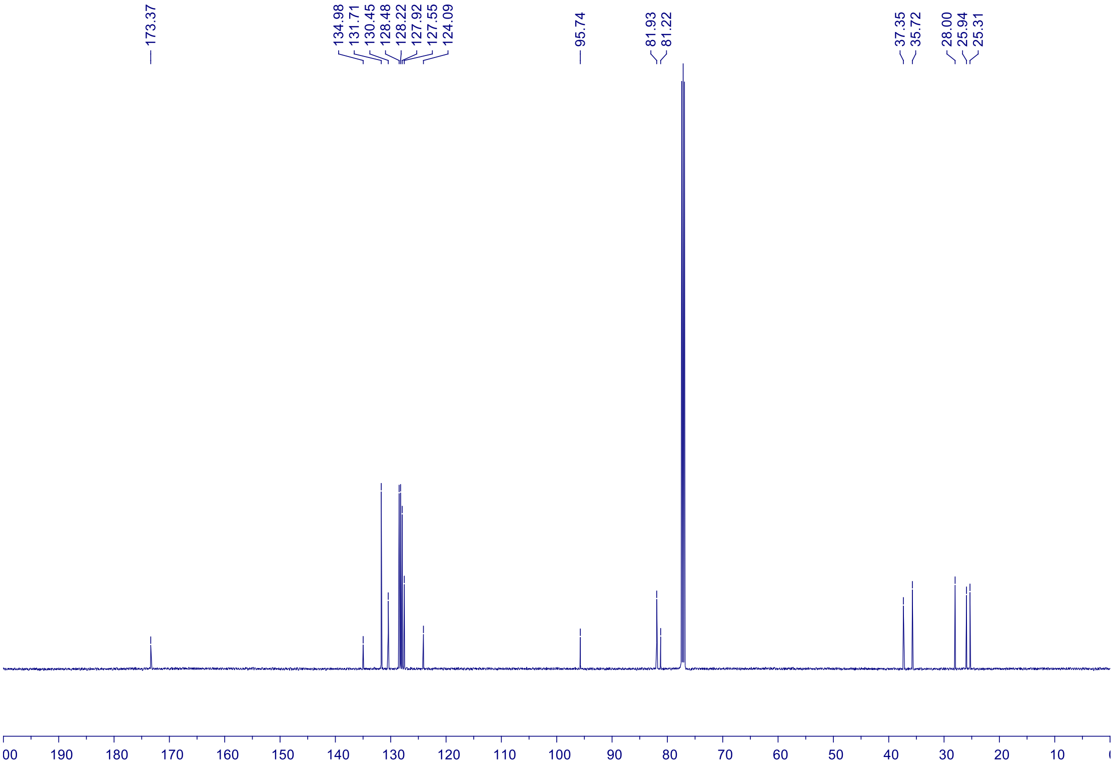

**8e** – $^{1}$H NMR (500 MHz, CDCl$_3$)

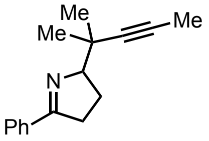


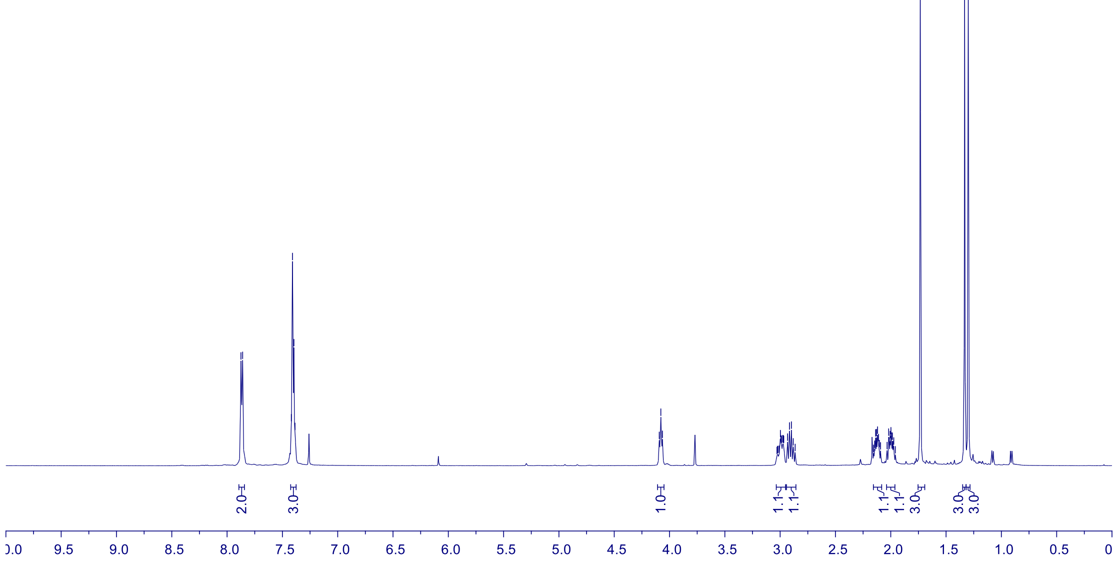


**8e** – $^{13}$C NMR (126 MHz, CDCl$_3$)

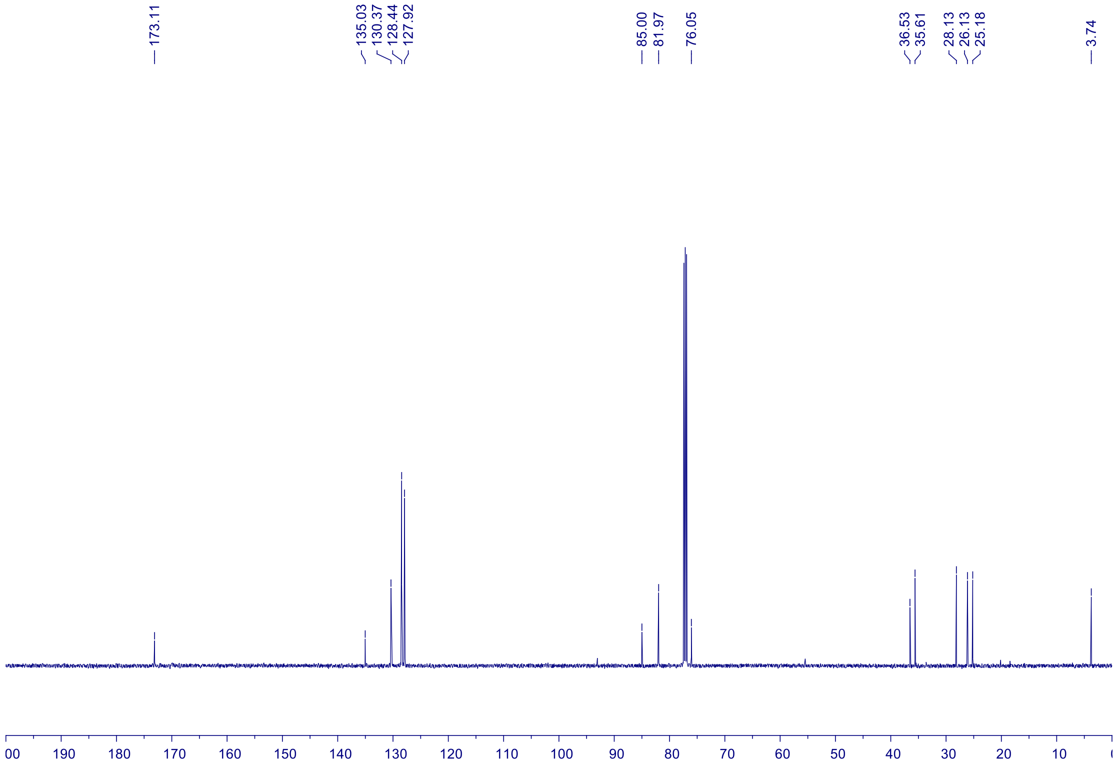

**8f** – $^{1}$H NMR (400 MHz, $CDCl_3$)

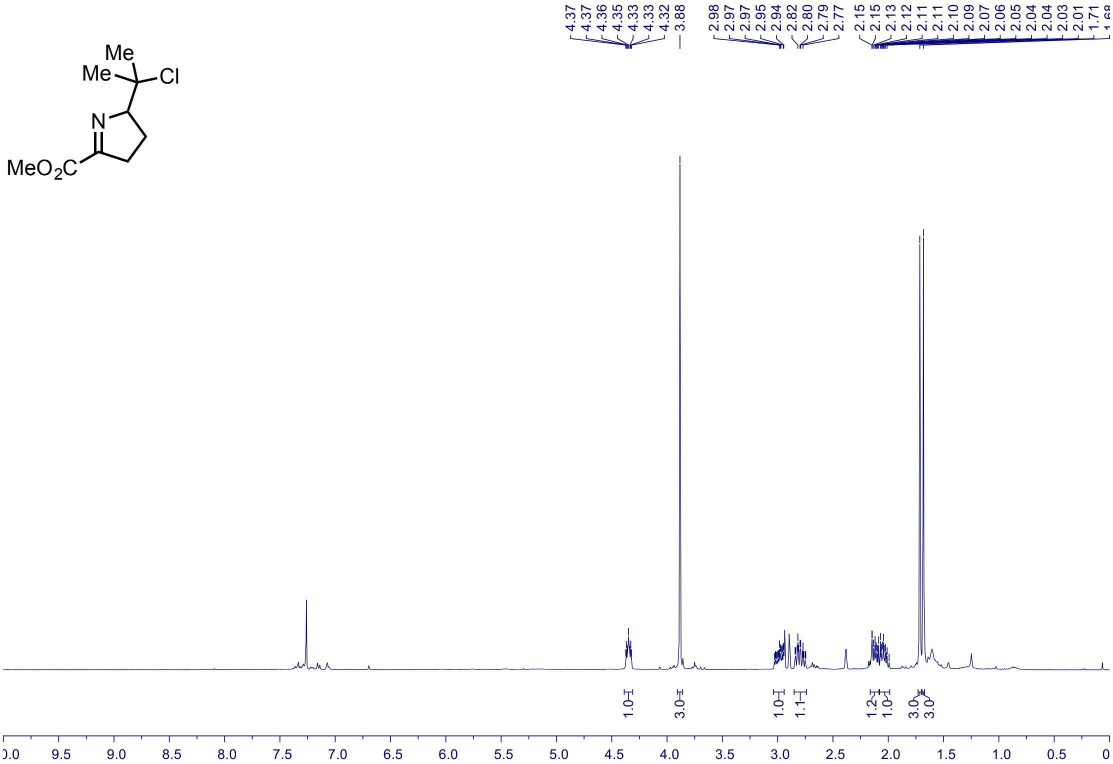


**8f** – $^{13}$C NMR (101 MHz, $CDCl_3$)

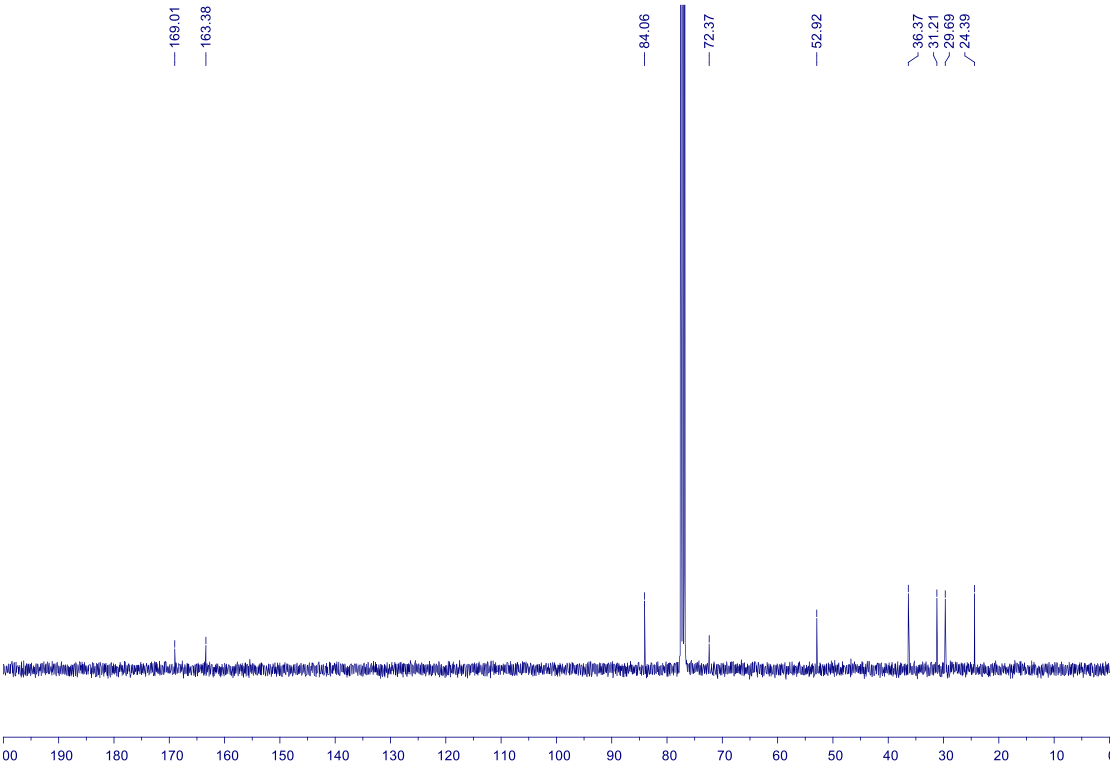

**8g** – $^{1}$H NMR (400 MHz, CDCl$_3$)

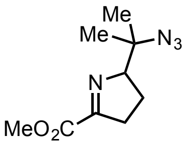


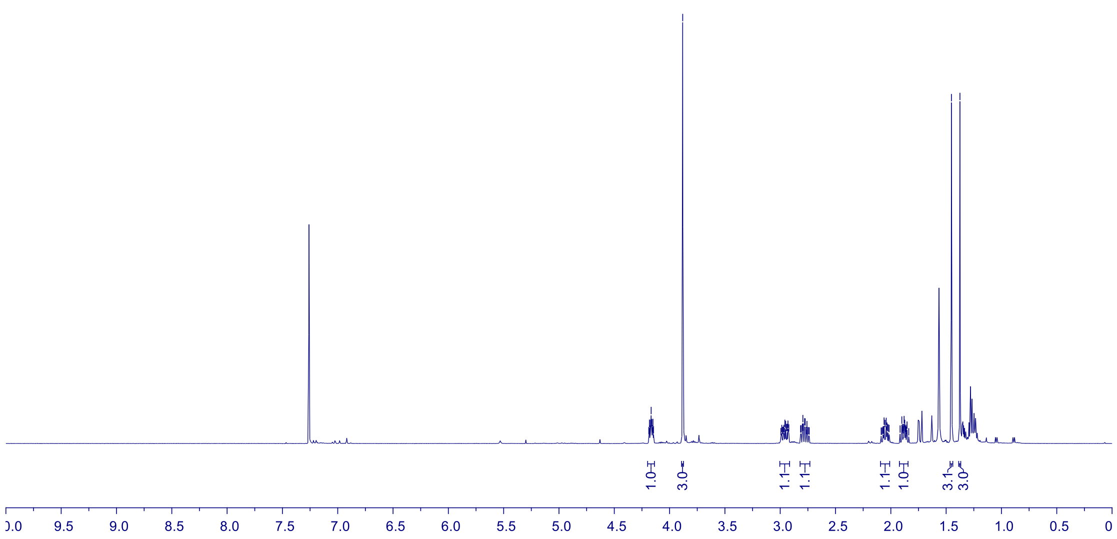


**8g** – $^{13}$C NMR (101 MHz, CDCl$_3$)

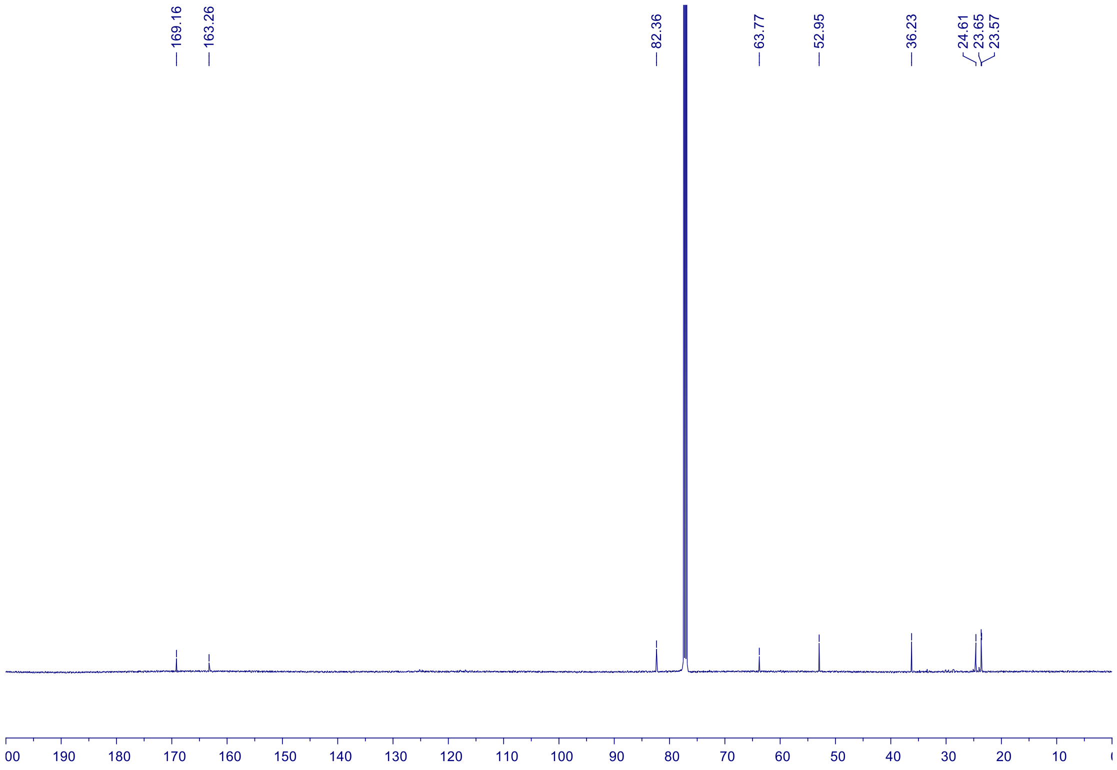

**8h** – $^{1}$H NMR (400 MHz, $CDCl_3$)

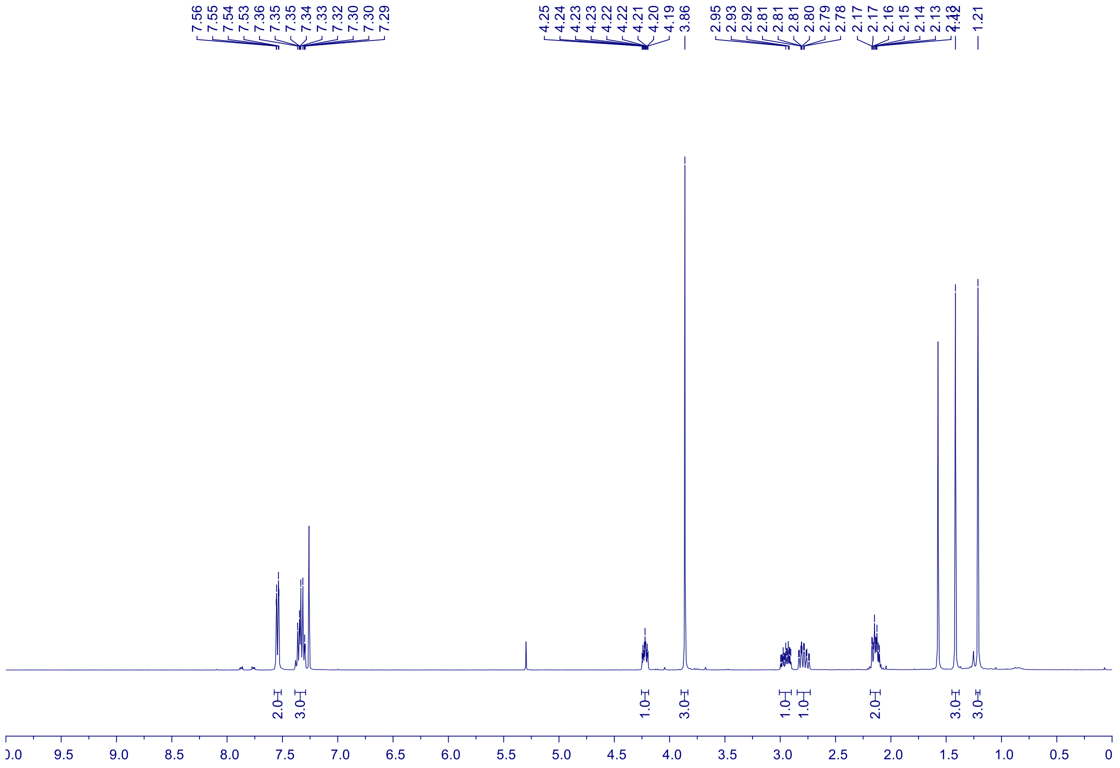


**8h** – $^{13}$C NMR (101 MHz, $CDCl_3$)

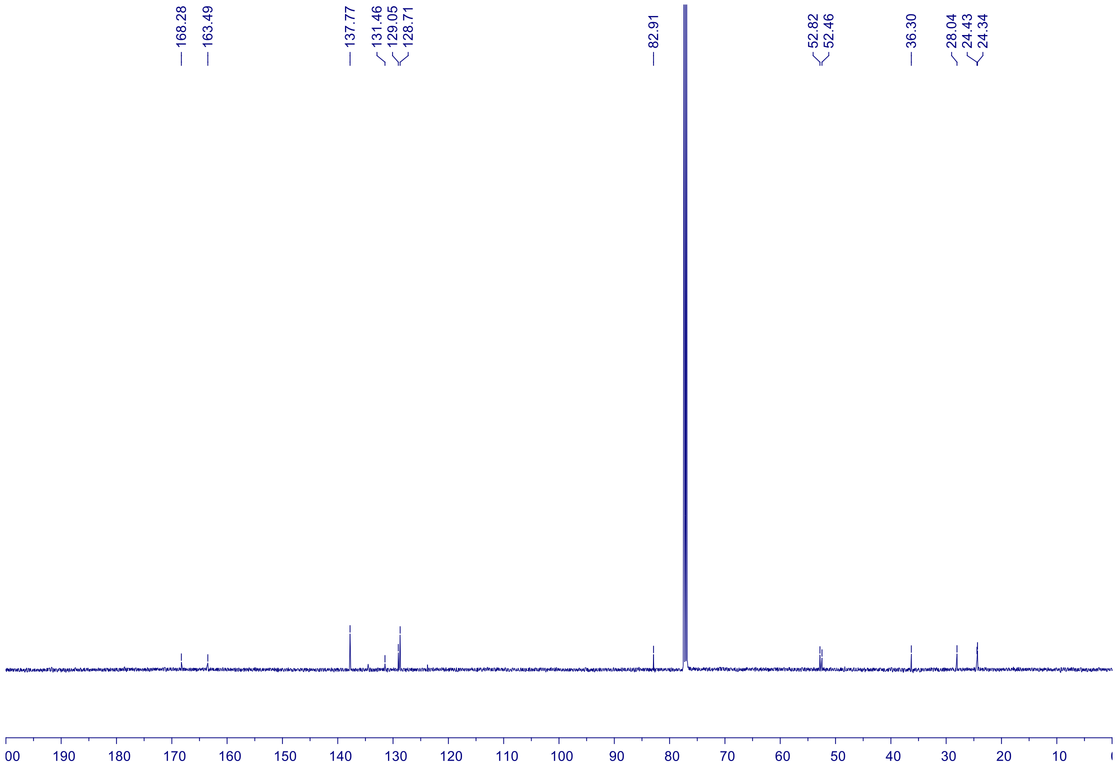

**8i** – $^{1}$H NMR (400 MHz, CDCl$_3$)

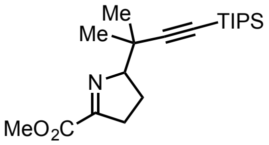


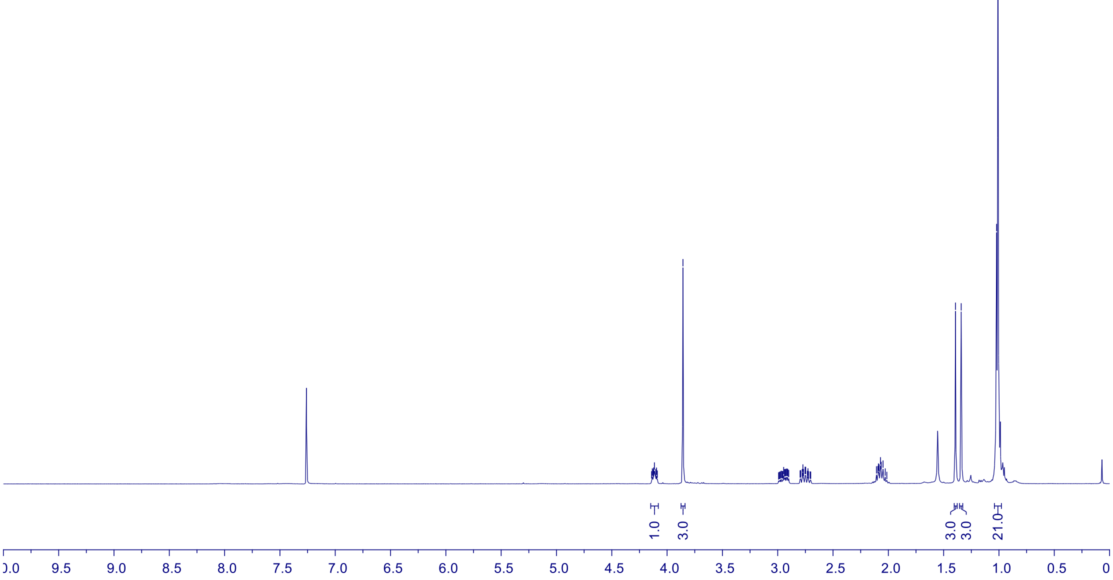


**8i** – $^{13}$C NMR (101 MHz, CDCl$_3$)

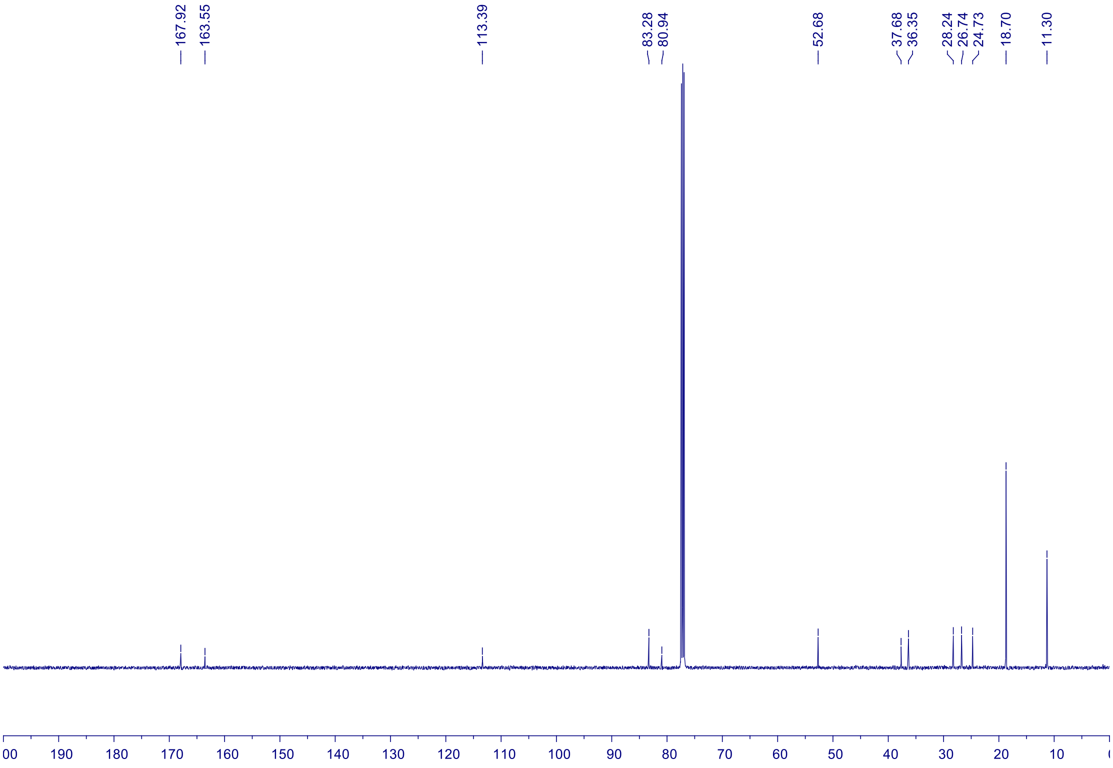

**10a** – $^{1}$H NMR (400 MHz, $CDCl_3$)

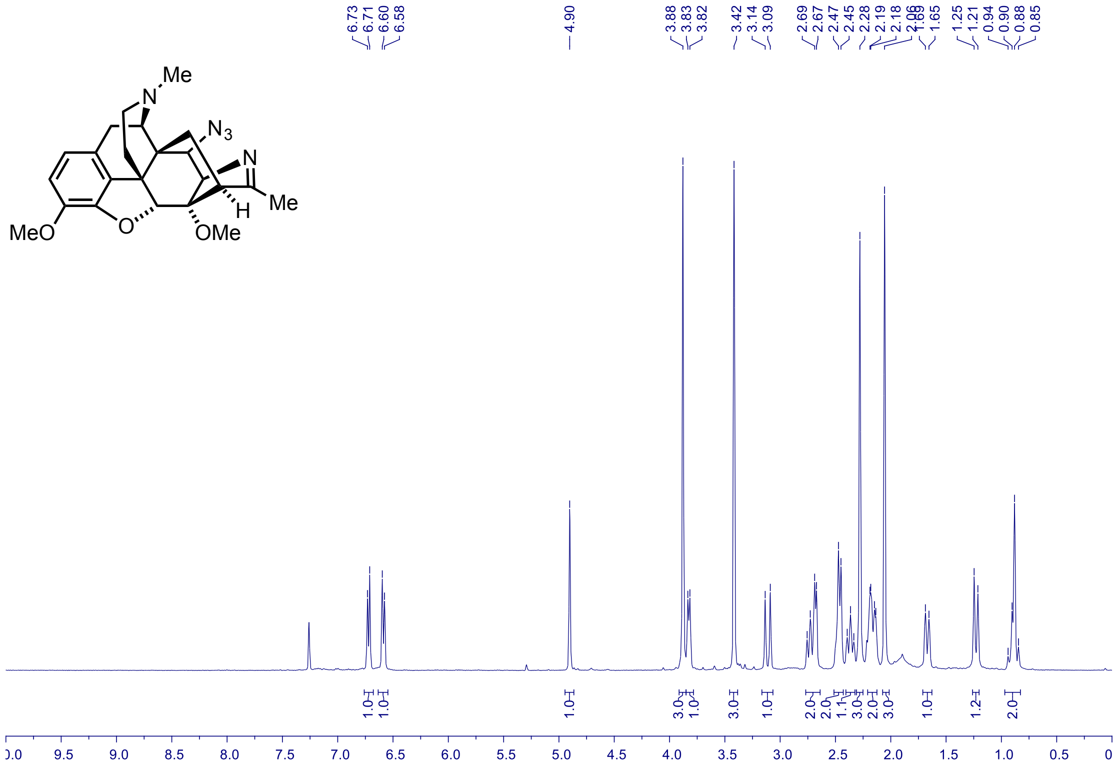

**10a** – $^{13}$C NMR (101 MHz, $CDCl_3$)

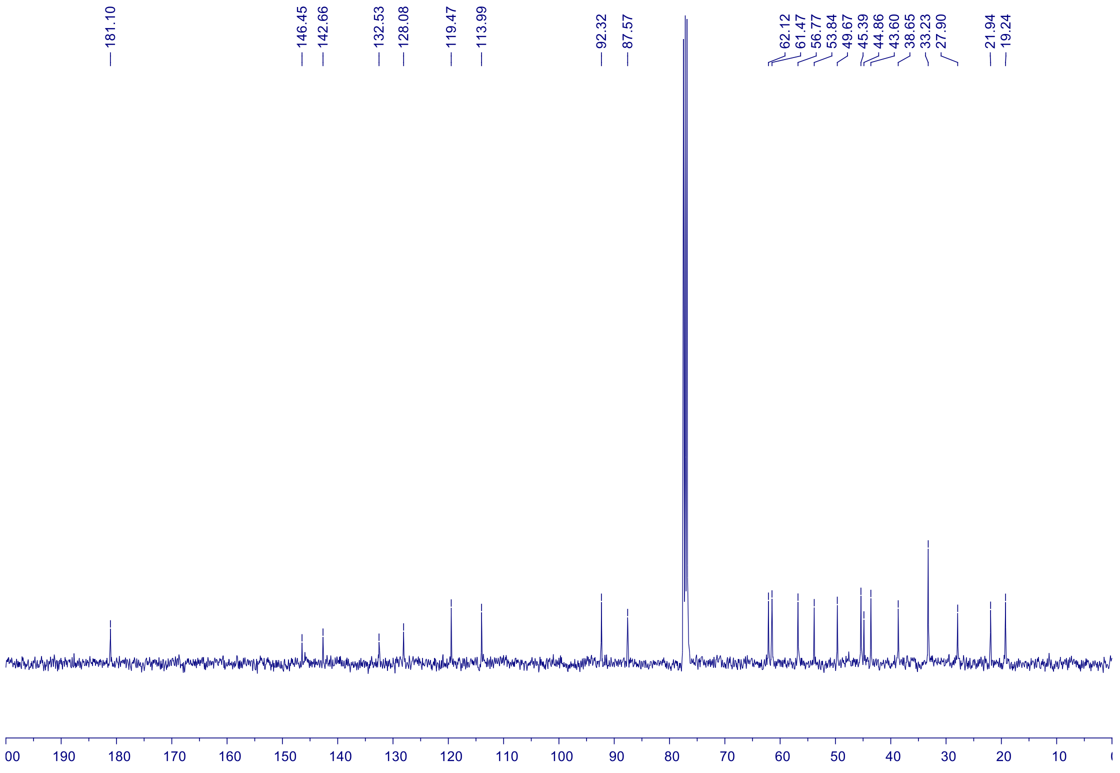

**10b** – $^{1}$H NMR (400 MHz, $CDCl_3$)

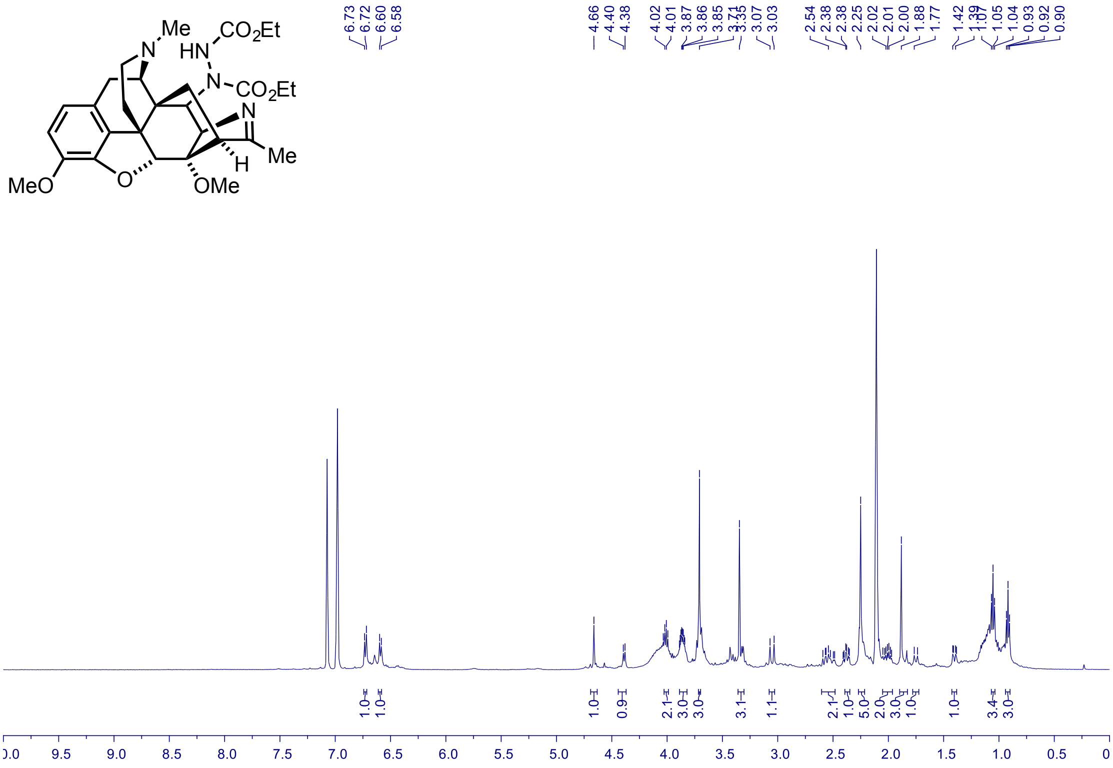


**10b** – $^{13}$C NMR (101 MHz, $CDCl_3$)

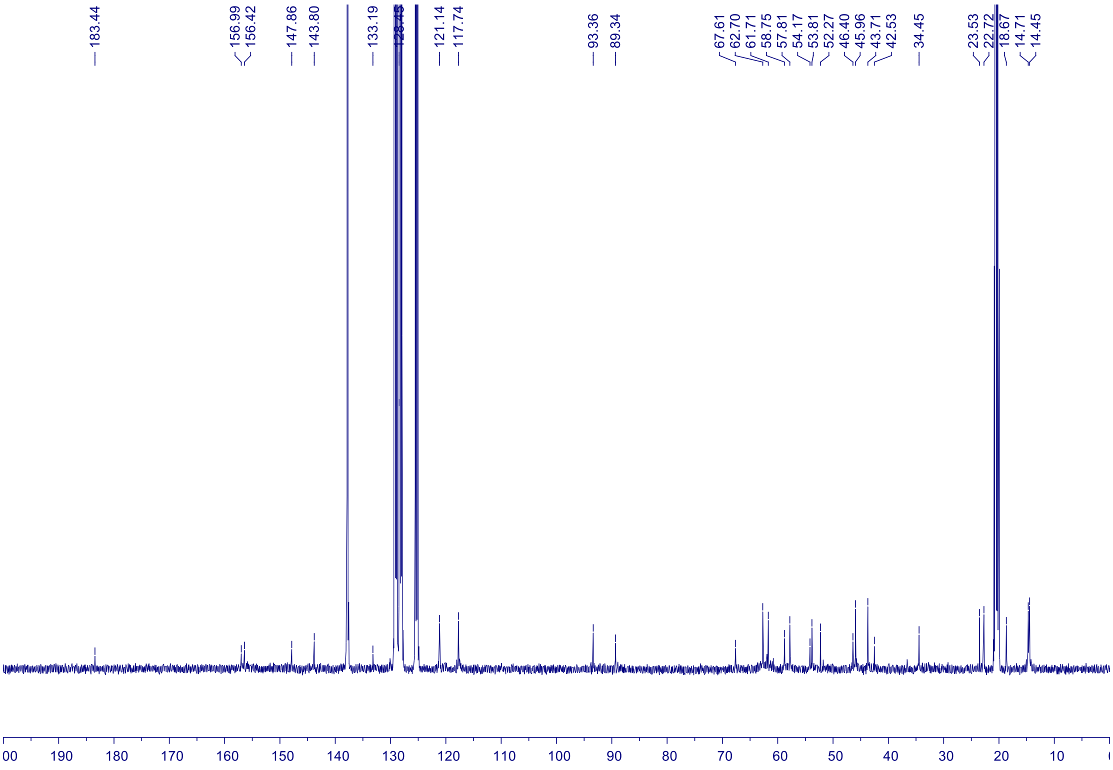

**10c** – $^{1}$H NMR (400 MHz, $CDCl_3$)

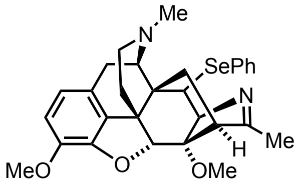


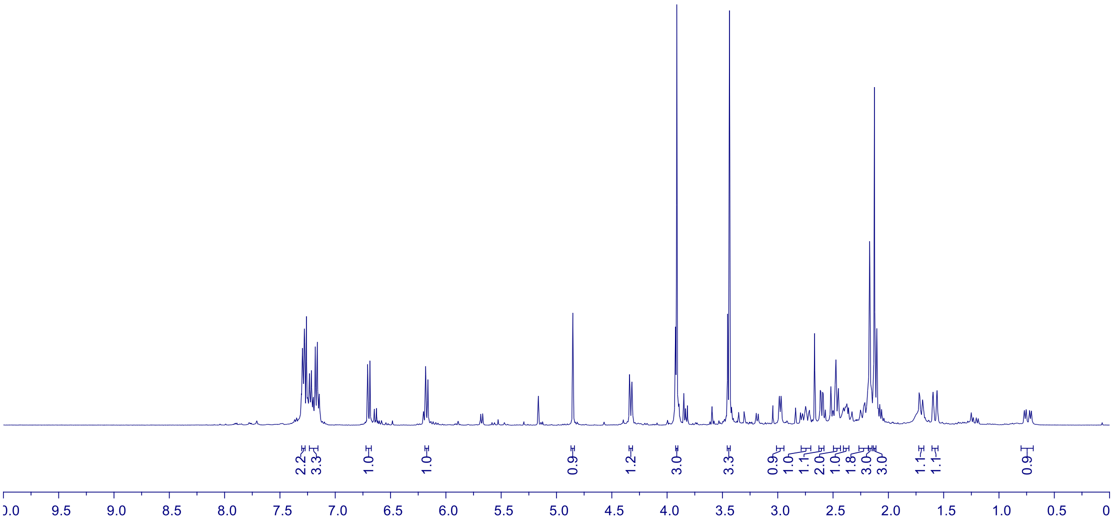


**10c** – $^{13}$C NMR (101 MHz, $CDCl_3$)

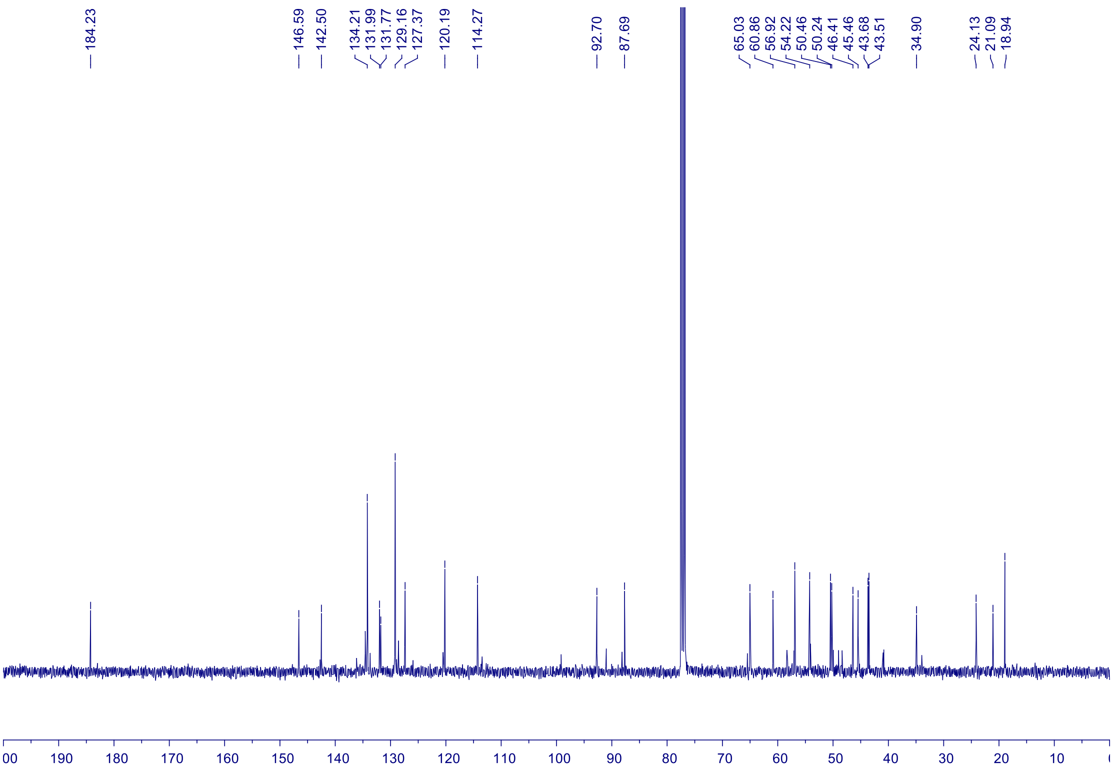

**nor-10c** – $^{1}$H NMR (400 MHz, $CDCl_3$)

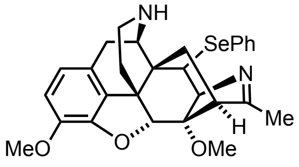

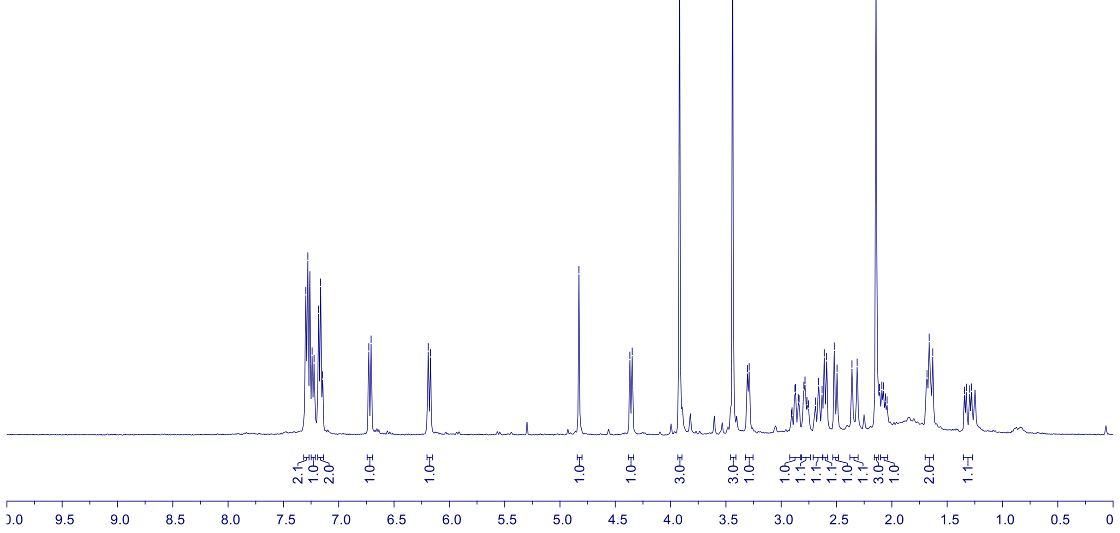


**nor-10c** – $^{13}$C NMR (101 MHz, $CDCl_3$)

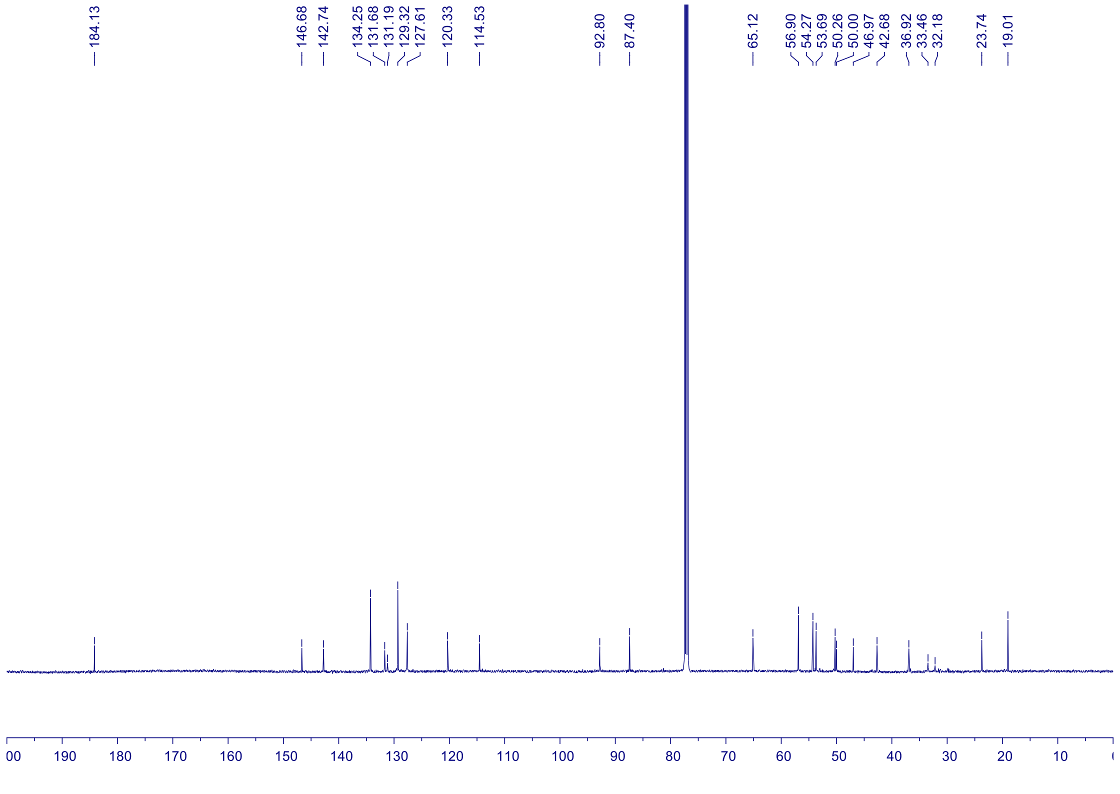

## 10 References


1 Hari, D. P. & Waser, J. *J. Am. Chem. Soc.* **138**, 2190 (2016).

2 Racine, S., Hegedüs, B., Scopelliti, R. & Waser, J. *Chem. Eur. J.* **22**, 11997 (2016).

3 Stridfeldt, E. *et al. Chem. Eur. J.* **22**, 16066 (2016).

4 Waser, J., Gaspar, B., Nambu, H. & Carreira, E. M. *J. Am. Chem. Soc.* **128**, 11693 (2006).

5 Michael, J. P. & Nkwelo, M. M. *Tetrahedron* **46**, 2549 (1990).

6 Patil, N. T., Pahadi, N. K. & Yamamoto, Y. *Synthesis* **13**, 2186 (2004).

7 Davies, J., Booth, S. G., Essafi, S., Dryfe, R. W. A. & Leonori, D. *Angew. Chem. Int. Ed.* **54**, 14017 (2015).

8 Fox, D. J., Pedersen, D. S. & Warren, S. *Chem. Commun.*, 2598 (2004).

9 Yang, C.-F., Wang, J.-Y. & Tian, S.-K. *Chem. Commun.* **47**, 8343 (2011).

10 Trost, B. M., Lehr, K., Michaelis, D. J., Xu, J. & Buckl, A. K. *J. Am. Chem. Soc.* **132**, 8915 (2010).

11 Hansford, K. A., Dettwiler, J. E. & Lubell, W. D. *Org. Lett.* **5**, 4887 (2003).

12 Kawanishi, M., Kotoku, N., Itagaki, S., Horiib, T. & Kobayashi, M. *Bioorg. & Med. Chem.* **12**, 5297 (2004).

13 Bentley, K. W. & Hardy, D. G. *J. Am. Chem. Soc.* **89**, 3267 (1967).

14 Dreyer, D. L., Tabata, S. & Horowitz, R. M. *Tetrahedron* **20**, 2977 (1964).

15 Tsierkezos, N. G. *J. Solution Chem.* **36**, 289 (2007).

16 Bard, A. J. & Faulker, L. R. *Electrochemical Methods: Fundamentals and Applications, 2nd Edition, Wiley, New York.* (2001).

17 Roth, H. G., Romero, N. A. & Nicewicz, D. A. *Synlett* **27**, 714 (2016).

18 Su, H., Li, W., Xuan, Z. & Yu, W. *Adv. Synth. Catal.* **357**, 64 (2014).

19 Wang, D.-S. *et al. J. Am. Chem. Soc.* **133**, 8866 (2011).

20 Verniest, G., Claessens, S. & Kimpe, N. D. *Tetrahedron* **61**, 4631 (2005).

21 Dechoux, L., Jung, L. & Stambach, J.-F. *Synthesis*, 242 (1995).

22 Tiecco, M., L.Testaferri, Tingoli, M., Bagnoli, L. & Marini, F. *J. Chem. Soc., Perkin Trans. 1*, 1989 (1993).

23 Cismenia, M. A. & Yoon, T. P. *Chem. Sci.* **6**, 5426 (2015).

24 Roberts, B. P. & Steel, A. J. *Tetrahedron Lett.* **34**, 5167 (1993).

25 Parr, R. G. *Density-Functional Theory of Atoms and Molecules*, Oxford University Press, Oxford U.K. (1989).

26 Frisch, M. J. *et al. Gaussian 09*, revision D.01; Gaussian, Inc. (2013).

27 Stephens, P. J., Devlin, F. J., Chabalowski, C. F. & Frisch, M. J. *J. Chem. Phys.* **98**, 11623 (1994).

28 Becke, A. D. *J. Chem. Phys.* **98**, 1372 (1993).

29 Becke, A. D. *J. Phys. Chem.* **98**, 5648 (1993).

30 Lee, C., Yang, W. & Parr, R. G. *Phys. Rev. B* **37**, 785 (1988).

31 Vleeschouwer, F. D., Speybroeck, V. V., Waroquier, M., Geerlings, P. & Proft, F. D. *Org. Lett.* **9**, 2721 (2007).

32 Hirshfeld, F. L. *Theoret. Chim. Acta* **44**, 129 (1977).

33 J. Davies, D.Svejstrup, T., Reina, D. F., Sheikh, N. S. & Leonori, D. *J. Am. Chem. Soc.* **138**, 8092 (2016).

34 Gonzalez, C. & Schlegel, H. B. *J. Phys. Chem.* **94**, 5523 (1990).

35 Gonzalez, C. & Schlegel, H. B. *J. Phys. Chem.* **90**, 2154 (1989).

36 Fukui, K. *Acc. Chem. Res.* **14**, 363 (1981).

37 Liu, J., Niwayame, S., You, Y. & Houk, K. N. *J. Org. Chem.* **63**, 1064 (1998).

38 DiLabio, G. A. & Pratt, D. A. *J. Phys. Chem. A* **104**, 1938 (2000).

39 DiLabio, G. A., LoFaro, A. D. & Wright, J. S. *J. Phys. Chem. A* **103**, 1653 (1999).

40 Scot, A. P. & Random, L. *J. Phys. Chem.* **100**, 16502 (1996).
